# Synthesis of 1-(2-Hydroxy-5-methylphenyl)-5-oxopyrrolidine-3-carboxylic Acid Derivatives as a Promising Scaffold Against Disease-Causing Bacteria Relevant to Public Health

**DOI:** 10.3390/molecules30122639

**Published:** 2025-06-18

**Authors:** Karolis Krikštaponis, Jūratė Šiugždaitė, Rita Vaickelionienė, Vytautas Mickevičius, Birutė Grybaitė

**Affiliations:** 1 Department of Organic Chemistry, Kaunas University of Technology, 50254 Kaunas, Lithuania; krikstaponis.karolis@gmail.com (K.K.); rita.vaickelioniene@ktu.lt (R.V.); birute.grybaite@ktu.lt (B.G.); 2 Department of Veterinary Pathobiology, Lithuanian University of Health Sciences, 47181 Kaunas, Lithuania; jurate.siugzdaite@lsmu.lt

**Keywords:** 5-oxopyrrolidine, hydrazone, heterocycles, antibacterial activity, biofilm formation

## Abstract

The increasing number of antibiotic-resistant pathogens forces us to accelerate the search for new antimicrobial agents. Based on this, we chose to synthesize a library of 1-(2-hydroxy-5-methylphenyl)-5-oxopyrrolidine-3-carboxylic acid derivatives and evaluate their antibacterial activity against various pathogens. A series of (2-hydroxy-5-methylphenyl)-5-oxopyrrolidine-3-carboxylic acid and its hydrazide derivatives were prepared and identified by the methods of IR, ^1^H, and ^13^C NMR spectroscopy and a microanalysis technique. The resulting compounds were evaluated in vitro for their efficacy against the Gram-positive *Staphylococcus aureus* (ATCC 9144), *Listeria monocytogenes* (ATCC 7644), and *Bacillus cereus* (ATCC 11778) bacterial strains as well as the Gram-negative *Escherichia coli* (ATCC 8739) bacteria. Oxacillin, ampicillin, and cefuroxime were used as control antibiotics. Among the obtained compounds, hydrazone with a 5-nitrothien-2-yl fragment surpassed the control cefuroxime (7.8 μg/mL) against almost all strains tested. Hydrazone with a 5-nitrofuran-2-yl moiety showed a slightly lower but also potent effect on all bacterial strains. Moreover, hydrazone with a benzylidene moiety demonstrated very strong inhibition of *S. aureus* (3.9 μg/mL) in comparison with the antibacterial drug cefuroxime (7.8 μg/mL). In addition, some of these compounds exhibited remarkable bactericidal properties. In a complete biofilm disruption study, 5-nitrothienylhydrazone showed excellent results in disrupting *S. aureus* and *E. coli* biofilms. The test results show the potential of the newly obtained derivatives as a source of antibacterial agents. Therefore, further studies on the molecular optimization of these compounds are necessary for the development of new antibacterial drugs.

## 1. Introduction

Heterocyclic compounds are the largest and most diverse class of natural or synthetic organic compounds that play a key role in the development and discovery of modern drugs, as they provide a variety of scaffolds and derivatives through robust synthetic methods [1]. These compounds have a unique chemical behavior that allows them to be widely used in drug development. Their stability, diverse functionality, and drug-likeness characterize their significance.

Pharmaceutical medications, agrochemicals, and veterinary are the main areas of application of heterocycles [2]. Their ability to interact with specific biological targets determines their important therapeutic effects [3]. As a result, most approved drugs contain one or more heterocyclic rings. According to the published data, compounds with one or more heterocycles in their structure make up more than 85% of all biologically active compounds [2,4], and they are prevalent in more than 90% of newly approved drugs [5,6].

Heterocyclic rings are found to be abundant in macromolecules such as enzymes, vitamins, natural products, and biologically active chemicals [7,8]. Compounds of natural origin, such as alkaloids, morphine, vinblastine, and reserpine, and antibiotics, such as cephalosporin, penicillin, and others, include a heterocyclic scaffold [9]. Numerous studies have shown them to possess anticancer, antiallergic, anti-inflammatory, antimicrobial, antiviral, antioxidant, anti-HIV, antidiabetic, anticonvulsant, and other biological effects [2,10,11], and they are used in the creation of antibiotics, antidepressants, and anticancer, antiviral, and anti-inflammatory medical preparations [12].

Heterocycles have been recognized as important structural motifs in the development of new drugs [13], particularly in the field of antimicrobial agents [14]. The importance of heterocycles in drug development is demonstrated by their ability to modify the physicochemical properties, biological effects, pharmacokinetics, and toxicological profile of a drug candidate [15]. Much research in the past decade has focused on therapeutic agents whose structures are based on the presence of different heterocycles in their molecular structure [16,17,18,19]. Scientists have successfully synthesized a large number of heterocyclic compounds and developed a number of new antibacterial drugs that can treat infections caused by drug-resistant strains of bacteria [20]. Cefiderocol, cefditoren pivoxil, ceftobiprope, sulbactam/durlobactam, sulopenem, ensifentrine, ceftobiprole medocaril sodium, and others, are antibacterial pharmaceuticals that have been approved in recent decades [21,22]. This is very important because antibiotic resistance in the modern world has become a crucial global problem, posing major challenges to medicine and public health. Over the past few decades, the misuse and overuse of antibiotics has accelerated the development of pathogen resistance, making previously effective treatments virtually ineffective. All of this has led to the emergence of previously controlled diseases and an increase in untreated infections, leading to prolonged illness and increased mortality. Various forms of malignant bacteria have emerged, each with their own level of resistance to medical treatment. *Escherichia coli*, multidrug-resistant tuberculosis [23], methicillin-resistant *Staphylococcus aureus* [24], and others, can cause serious infections. Therefore, urgent and coordinated efforts are necessary to address this important problem. However, the growing understanding of bacterial pathogenesis and cellular communication has revealed many potential drug discovery strategies that can be used to treat bacterial infections [25].

In our previous studies, we revealed some five-membered heterocycle-based compounds with promising bioactivity against various cancer cell lines [26,27,28] and pathogenic strains [29,30]. Our research findings have led to further exploration of this area, and this time, we present our latest research in the search for promising antibacterial agents.

## 2. Results and Discussion

Derivatives obtained from aromatic amines are widely used in the production of various important and useful substances, such as drugs, polymers, dyes, surfactants, cosmetics, corrosion inhibitors, photosensitizers, and agricultural protection agents [31].

For this study, we chose 2-amino-4-methylphenol (**1**) as the starting compound, hoping to obtain a series of its derivatives with promising pharmacological properties. The target acid **2**, in 88% yield, was synthesized by the modification of the amine **1** with itaconic acid using the aza-Michael method [32,33].

**Figure 1:**
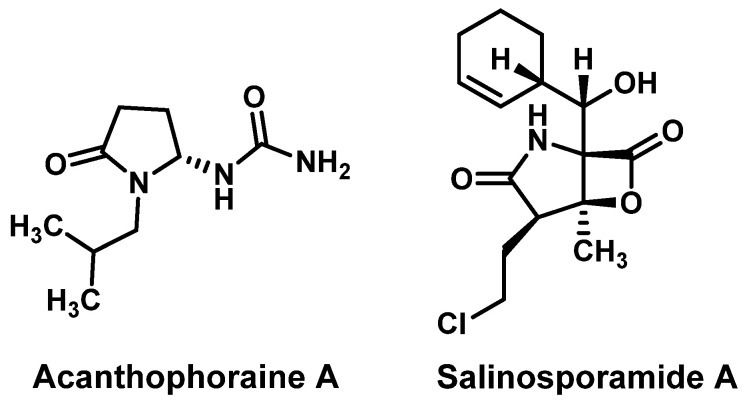
Naturally occurring alkaloids featuring a 2-pyrrolidone core.

Several natural alkaloids containing a 2-pyrrolidone core have been reported to possess notable antibacterial and antimicrobial activities such as Acanthophoraine A and Salinosporamide A (Figure 1), and we were encouraged to design and synthesize novel analogues based on this scaffold with the aim of enhancing their biological efficacy [34,35].

The formation of a 5-oxopyrrolidine-3-carboxylic acid fragment was confirmed by the corresponding signals of COCH_2_, CH, and NCH_2_ groups in the ^1^H NMR spectra as follows: 2.58–2.68, 3.26–3.42, and 3.76–3.93 ppm. A broad singlet at 12.65 ppm confirmed the COOH group present in the molecular structure. The carbon peaks of the corresponding groups in the ^13^C NMR spectrum were observed at 33.74, 36.22, and 50.98 ppm, and the COOH carbon resonance line was found at 174.41 ppm. (Supplementary Files, Figures S1 and S2).

**Scheme 1:**
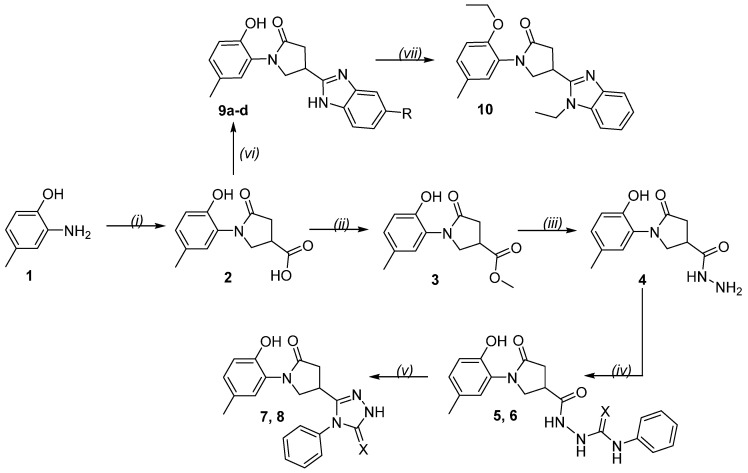
Synthesis of aza-heterocycles. Reagents and conditions: (**i**) itaconic acid, water, Δ, 2 h; (**ii**) MeOH, H_2_SO_4_, Δ, 8 h; (**iii**) N_2_H_4_·H_2_O, i-PrOH, Δ, 8 h; (**iv**) phenyl isothiocyanate (for **5**) or phenyl isocyanate (for **6**), MeOH, Δ, 4 (for **5**) or 6 (for **6**) h; (**v**) aqueous 4% NaOH, Δ, 3 (for **7**) or 4 (for **8**) h; (**vi**) unsubstituted (**9a**) or the corresponding substituted (**9b**–**d**) benzene-1,2-diamine, 4N HCl, Δ, 24 h; (**vii**) EtI, KOH, K_2_CO_3_, DMF, r.t. 6 h. **2**–**10**. **5**, **7** X = S; **6**, **8** X = O; **9a** R = H; **9b** R = F; **9c** R = Cl; **9d** R = Me.

The intended acid hydrazide **4** was prepared by esterification of acid **2** followed by hydrazinolysis of the prepared ester **3** (Supplementary Files, Figures S3 and S4) with hydrazine monohydrate (Scheme 1) (Supplementary Files, Figures S5 and S6). In this work, the structural elucidation was mainly focused on the analysis of the ^1^H and ^13^C NMR spectra. The spectra of **3** and **4** were in excellent agreement with the desired structures.

Further, according to Scheme 1, the preparation of the target thiosemicarbazide **5** and semicarbazide **6** was accomplished by interacting the acid hydrazide **4** with phenyl isothiocyanate or phenyl isocyanate, respectively. The reaction was performed in methanol at reflux for 4 or 6 h. The reactants were used in a 1:1 molar ratio, and the resulting compounds were isolated in 89% and 91% yields. The NMR spectra of thiosemicarbazide **5** showed three singlets at 9.58, 9.86, and 10.17 ppm for the three protons of 3 NH (^1^H) and an intense resonance line at 172.24 ppm for the two C=O carbon atoms, as well as a spectral line for C=S at 181 ppm. In the NMR spectra of semicarbazide **6**, singlets at 8.11, 8.42, 8.79, 8.98, 9.62, and 9.91 ppm proved the presence of three NH groups (^1^H), and the peaks at 172.21, 172.61, and 176.79 ppm indicated three C=O groups (^13^C) (Supplementary Files, Figures S7–S10).

Next, the obtained phenylhydrazine-1-carbothioamide **5** and phenylhydrazine-1-carboxamide **6** were converted to the corresponding triazoles **7** and **8** by the classical technique for the cyclization of the appropriate semicarbazides. The alkaline cyclization of **5** and **6** in aqueous 4% sodium hydroxide for 3 or 4 h, respectively, led to the formation of triazolethione **7** or triazolone derivative **8** (Scheme 1). The desired compounds were isolated in good yields of 83% and 79%. The cyclic structure of both compounds was fully approved by the used structure identification techniques, and the ^1^H and ^13^C NMR data can be found in the Supplementary Files, Figures S11–S14. Analysis of the ^1^H NMR spectrum of triazolethione **7** showed a characteristic singlet at 13.41 ppm for the NH of the 1,2,4-triazole ring, and the ^13^C NMR spectrum also showed carbon peaks of the N=C and thiocarbonyl groups at 168.38 and 182.99 ppm, respectively.

If we analyze the NMR spectrum of compound **8**, there is clear evidence for the formation of the 1,2,4-triazolone ring, i.e., the proton of the NH group resonates at the characteristic field of the ^1^H NMR spectrum, namely at 11.93 ppm, and the ^13^C NMR demonstrates resonance lines at 168.48 and 173.63 ppm for the carbons of the N=C and the carbonyl of the triazole ring.

Starting from the common precursor, i.e., carboxylic acid **2**, a series of benzimidazoles **9a**–**d** were obtained by heating at reflux a mixture of initial acid **2** with the appropriate benzene-1,2-diamine in 4N hydrochloric acid for 24 h, as described in [36]. Depending on the applied benzene-1,2-diamine, unsubstituted benzimidazole **9a**, 5-fluoro-**9b**, 5-chloro-**9c**, and 5-methyl-**9d** benzimidazoles were isolated from the reaction mixtures in the yield range of 54–67%. Based on the elemental analysis and spectral data, the structure of the compounds was fully confirmed. In the ^1^H NMR spectrum of compound **9a**, the singlet at δ 12.57 ppm showed the presence of the NH proton. Five protons of the benzimidazole phenyl ring resonated in the range of 7.11–7.76 ppm. The remaining protons of the molecule resonated in excellent agreement with the proposed structure (Supplementary Files, Figure S15). As for the ^13^C NMR spectrum, the spectral peaks of the carbon atoms of the molecule resonated in the expected areas of the spectrum and confirmed the formation of the desired benzimidazole **9a** (Supplementary Files, Figure S16).

Biological evaluation has demonstrated *N*-alkylated benzimidazoles to be promising compounds in drug design and discovery [37].

In this work, we performed the alkylation of compound **9a** using a large (6-fold) excess of iodoethane to avoid the formation of a mixture of *O*- and *N*-alkylated compounds. The reaction was performed in dimethylformamide as a solvent and in the presence of potassium hydroxide and potassium carbonate in the reaction mixture. After stirring for 6 h at room temperature, the alkylated product **10** was isolated from the mixture. The ^1^H and ^13^C NMR spectra showed *N*- and *O*-alkylation. In the ^1^H NMR spectrum, triplets at 1.19 and 1.36 ppm and four protons in the range of 3.75–4.14 ppm confirmed the presence of two CH_3_CH_2_ fragments in the target compound. The absence of OH and NH proton singlets in the ^1^H NMR spectrum led to the conclusion that *N*- as well as *O*-alkylation occurred. The ^13^C NMR spectrum revealed resonance lines at 14.53, 14.57 (2CH_3_CH_2_), and 29.46, 63.79 (2CH_3_CH_2_) ppm, which were assigned to the carbons of the ethyl fragments (Supplementary Files, Figures S23 and S24).

A library of hydrazones **11a**–**d** and **12a**–**j** based on acid hydrazide **4** were also synthesized (Scheme 2). To achieve this, condensation of compound **4** with heterocyclic (for **11**) and aromatic (for **12**) aldehydes in propan-2-ol was caried out. All obtained structures **11** and **12** were confirmed by IR, ^1^H NMR, ^13^C NMR, and elemental analysis data. Analyzing the ^1^H NMR spectra of hydrazones **11a**–**d**, the presence of two singlets in the ranges of 7.91–8.47 ppm for the CH=N group and 11.50–11.98 ppm for the NH of the hydrazone fragment was found. The ^13^C NMR spectra of hydrazones **11a**–**d** showed additional resonance lines originating from the carbonyl, azomethine, and newly attached aryl ring carbons (Supplementary Files, Figures S25–S32). Compounds **12a**–**j** were also fully confirmed using microanalysis and spectral results. The ^1^H and ^13^C NMR spectra can be found in the Supplementary Files, Figures S33–S50.

Hydrazones derived from acid hydrazides and aromatic aldehydes have mostly linear structures of stereoisomers arising from *Z/E* isomerism with respect to the double C=N bond and restricted rotation around the amide bond [38]. The hydrazones are usually obtained in an E configuration at the imine double bond and as a *Z/E* conformers mixture due to the amide bond [39]. The ^1^H NMR spectra in DMSO-d_6_ showed two sets of signals in a 65:35 intensity ratio (*Z*/*E*) for hydrazones **11a**–**d** and in an intensity ratio of 60:40 (*Z*/*E*) for arylidene hydrazones **12a**–**j**.

**Scheme 2:**
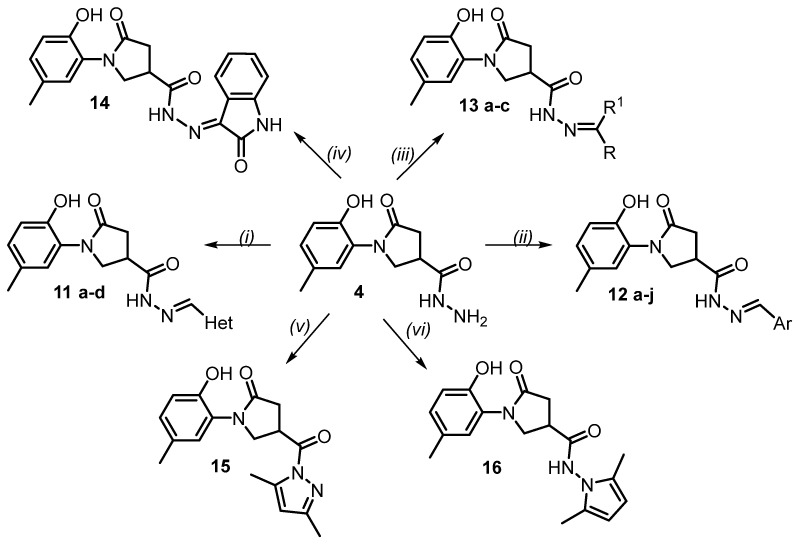
Synthesis of compounds **12**–**16**. Reagents and conditions: (**i**) the corresponding carbaldehyde, i-PrOH, Δ, 2 h; (**ii**) the corresponding aromatic aldehyde, i-PrOH, Δ, 2 (for **12a**–**i**) or 3 (for **12j**) h; (**iii**) for **13a**, **b**, acetone or ethyl methyl ketone, Δ, 4 h; for **13c**, 4-aminoacetophenone, glacial AcOH, i-PrOH, Δ, 20 h; (**iv**) isatin, MeOH, AcOH, Δ, 3 h; (**v**) pentane-2,4-dione, conc. hydrochloric acid, i-PrOH, Δ, 2 h; (**vi**) hexane-2,5-dione, glacial AcOH, i-PrOH, Δ, 4 h. **11a** Het = thien-2-yl; **11b** Het = 5-nitro-2-thienyl; **11c** Het = furan-2-yl; **11d** Het = 5-nitrofuran-2-yl; **12a** Ar = Ph; **12b** Ar = 2,4-F_2_Ph; **12c** Ar = 4-ClPh; **12d** Ar = 4-BrPh; **12e** Ar = 4-O_2_NPh; **12f** Ar = 4-MePh; **12g** Ar = 4-Me_2_NPh; **12h** Ar = 2,3,4-(MeO)_3_Ph; **12i** Ar = 3,4,5-(MeO)_3_Ph; **12j** Ar = 1-naphthyl; **13a** R = R^1^ = Me; **13b** R = Me, R^1^ = Et; **13c** R = Me, R^1^ = 4-H_2_N-Ph.

In the case of ketones (Scheme 2), their reactions with hydrazide **4** gave a set of expected hydrazones **13a**–**c**. Reactions with acetone and ethyl methyl ketone afforded *N*′-propan-2-ylidene-**13a** or *N*′-butan-2-ylidene-**13b** carbohydrazides, respectively. Meanwhile, condensation with aminoacetophenone in propan-2-ol resulted in *N*′-(1-(4-aminophenyl)ethylidene)carbohydrazide derivative **13c**. The NMR spectra of the obtained **13a**–**c** fully confirmed their structures (Supplementary Files, Figures S51–S56). Compounds **13a** and **13b** were found to exist in DMSO-d_6_ solutions as a mixture of *Z*/*E* conformers with an intensity ratio of 57:43, whereas **12c** had a *Z*/*E* rotamer ratio of 65:35.

In an extension of the present study, the reaction of acid hydrazide **4** with isatin was carried out. The reaction was realized in methanol using a molar ratio of reactants of 1 to 1.5, and a catalytic amount of glacial acetic acid was used to accelerate the reaction. Compound **14** was isolated in a good yield of 69%. The ^1^H NMR spectrum of isatin derivative **14** showed a proton signal of the amide group at 11.33 ppm, while the NH signal of the isatin core was visible at 10.81 ppm [40]. In the ^13^C NMR spectrum, the carbons of the isatin, amide, and 5-oxopyrrolidine carbonyls were observed as an intense peak at 172.11 ppm (Supplementary Files, Figures S57 and S58).

To synthesize small molecules suitable as antimicrobial agents, 3,5-dimethylpyrazole **15** and 2,5-dimethylpyrrole **16** derivatives were obtained. The condensation of acid hydrazide **4** with the appropriate diketone, as depicted in Scheme 2, was performed in propan-2-ol by catalytically accelerating the reaction with concentrated hydrochloric acid for **15** or glacial acetic acid for **16**. In the ^1^H NMR spectrum of pyrazole **15**, a proton singlet of a CH_pyr_ was observed at 6.22 ppm, and an additional singlet at 2.19 ppm integrated for six protons indicated the presence of two new methyl groups in the molecule. In the ^13^C NMR spectrum, the carbons of the same groups were found as follows: 111.50, 13.54, and 14.07 ppm (Supplementary Files, Figures S59 and S60).

Examining the ^1^H NMR spectrum of compound **16**, an intense singlet of the protons of two CH groups of the newly formed pyrrole ring was observed at 5.65 ppm, and peaks of two CH_3_ groups were found to resonate at 1.97 and 2.00 ppm. In the ^13^C NMR results, the pyrrole structure was affirmed by the presence of the spectral lines at 10.92, 10.96 (2 CH_3_), and 103.08 (2 CH), as well as at 126.74 and 126.76 (2 C–CH_3_) ppm.

The structures of the obtained compounds and their yields (%) are shown in Table 1.

**Figure 2:**
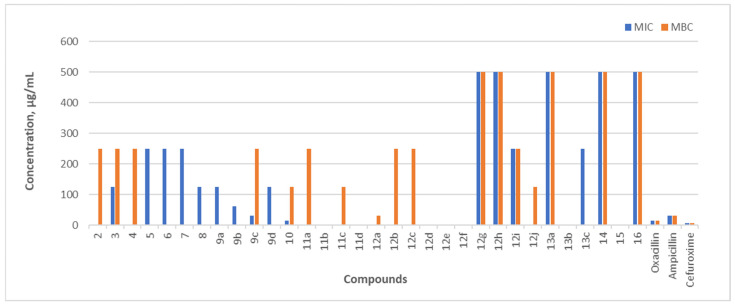
In vitro MIC and MBC of the tested compounds (**2**–**16**) against *Staphylococcus aureus* (ATCC 9144), expressed in µg/mL. The antibacterial activity of the compounds was compared with standard antibiotics: oxacillin, ampicillin, and cefuroxime.

**Figure 3:**
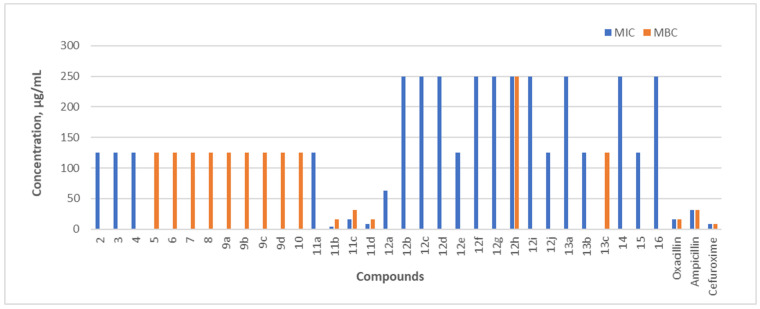
In vitro MIC and MBC of the tested compounds (**2**–**16**) against expressed in *L. monocytogenes* (ATCC 7644) µg/mL. The antibacterial activity of the compounds was compared with standard antibiotics: oxacillin, ampicillin, and cefuroxime.

**Figure 4:**
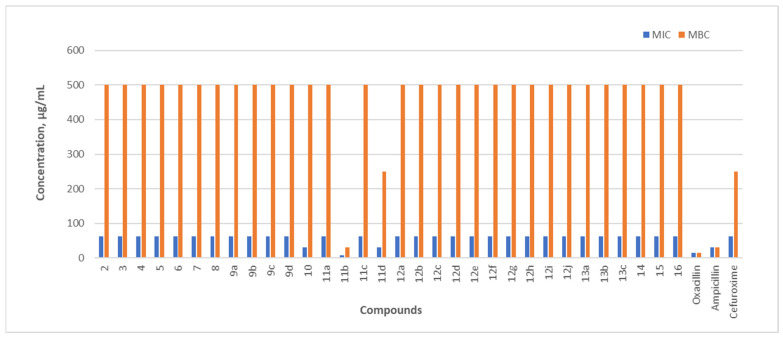
In vitro MIC and MBC of the tested compounds (**2**–**16**) against expressed in *B. cereus* (ATCC 11778) µg/mL. The antibacterial activity of the compounds was compared with standard antibiotics: oxacillin, ampicillin, and cefuroxime.

**Figure 5:**
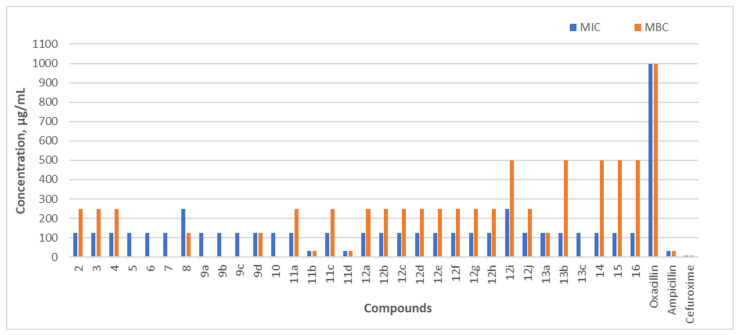
In vitro MIC and MBC of the tested compounds (**2**–**16**) against expressed in *E. coli* (ATCC 8739) µg/mL. The antibacterial activity of the compounds was compared with standard antibiotics: oxacillin, ampicillin, and cefuroxime.

The antibacterial activity of the synthesized compounds was evaluated as described in Section 3.2. The minimum inhibitory concentration (MIC) and minimum bactericidal concentration (MBC) values were determined against three Gram-positive bacterial strains—*Staphylococcus aureus* subsp. *aureus* (ATCC 9144), *Listeria monocytogenes* (ATCC 7644), and *Bacillus cereus* (ATCC 11778)—as well as the Gram-negative strain *Escherichia coli* (ATCC 8739). The results are graphically depicted in Figure 2, Figure 3, Figure 4 and Figure 5.

*Staphylococcus aureus* is a Gram-positive coccus and is a pathogen responsible for hospital-acquired (nosocomial) infections, often causing skin and upper respiratory tract infections. A key factor contributing to its pathogenicity is its ability to form biofilms, which protect it from host immune responses and antimicrobial treatments [41]. Three compounds from the hydrazone class, containing 5-nitro-2-thiophene, 5-nitro-2-furfural, or an additional benzene ring in their structure—**11b**, **11d**, and **12a**—exhibited bacteriostatic activity against *S. aureus.* The MIC value of these compounds against this pathogenic *S. aureus* was 3.90 µg/mL, which is 2 times lower than that of cefuroxime, 4 times lower than that of oxacillin, and 8 times lower than that of ampicillin. Compounds **11b** and **11d** also demonstrated strong bactericidal properties against *S. aureus.* The MBC of compound **11d** was comparable to cefuroxime, which showed the best bactericidal effect among the control antibiotics, while the MBC value for compound **11b** was half as low. The bactericidal and bacteriostatic activities of the other unmentioned compounds were much weaker. Hydrazone **13b**, the pyrazole-containing compound **15**, the compound containing an isatin fragment **14**, hydrazinecarbo(thio)amides **5**, **6**, triazole derivatives **7**, **8**, and benzimidazoles **9a** did not exhibit bactericidal activity against *S. aureus.*

*L. monocytogenes* is a Gram-positive, non-spore-forming, motile, rod-shaped facultative intracellular bacterium. It is one of the most virulent foodborne pathogens [42]. Among all the new compounds synthesized during the study, only those containing thiophene and furfural heterocyclic rings (compounds **11b**–**d**) exhibited strong antibacterial properties. The MIC of compound **11b** (7.8 µg/mL) was equivalent to that of the second-generation cephalosporin antibiotic cefuroxime; however, its MBC (15.23 µg/mL) was two-fold higher. The activity of this compound and others with antibacterial properties may be related to the inhibition of DNA, RNA, cell wall, or protein synthesis. Compounds containing a lactam ring are characterized by an inhibition of the synthesis of the peptidoglycan layer of the cell wall [43]. These compounds interfere with the normal functioning of enzymes such as transpeptidase, transglycosylase, and/or carboxypeptidase, which are responsible for the final stages of peptidoglycan synthesis, leading to bacterial cell death.

*B. cereus* is a Gram-positive, rod-shaped bacterium that forms endospores and produces biofilms [44]. It is commonly found in soil and food. The MIC of compound **11b** (7.8 µg/mL) was two-fold lower (15.23 µg/mL) compared with oxacillin and four-fold lower compared with ampicillin—31.20 µg/mL. The MIC of compound **11d** and the *N*-alkylated benzimidazole fragment-containing compound **10** was equal to the MIC of ampicillin (31.25 µg/mL) and 2 times lower than that of cefuroxime (62.4 µg/mL). The compound with the strongest bactericidal activity against *B. cereus* was the 5-nitrothiophen-2-yl-containing hydrazone **11b**, with a minimum bactericidal concentration (MBC) of 31.25 µg/mL.

During the study, the antibacterial activity of the synthesized compounds was also tested against *E. coli*, a Gram-negative, non-spore-forming, and biofilm-forming intestinal rod [45]. The compounds with the highest antibacterial activity were hydrazones **11b** and **11d**, which contain 5-nitro-2-thiophene and 5-nitro-2-furfural fragments in their structure. The MIC and MBC values of the latter compounds were equal to the MIC and MBC values of ampicillin (31.25 µg/mL) and 4 times higher than those of cefuroxime (7.80 µg/mL). The MIC values of the other compounds were ≤125 µg/mL. The minimum bactericidal concentration of many compounds was 250 µg/mL, or they did not exhibit bactericidal activity against *E. coli.*

The results show that compounds **11b** and **11d** exhibited the widest spectrum of activity. They demonstrated antibacterial activity against all tested bacterial strains, with the strongest bacteriostatic effect observed against Gram-positive *S. aureus* (3.90 µg/mL) and *L. monocytogenes* (3.90 µg/mL for compound **11b** and 7.80 µg/mL for compound **11d**). The most potent antibacterial properties were found in the hydrazones containing 5-nitro-2-thiophene and 5-nitro-2-furfural structures in their composition.

Compounds **11b** and **11d** demonstrated the broadest spectrum of activity in the results. Consequently, these compounds were selected to assess their ability to disrupt biofilms produced by the Gram-positive coccus *S. aureus* and the Gram-negative rod *E. coli*.

Using the tube method, moderate biofilm formation was observed for both *S. aureus* and *E. coli*. After treating the bacterial biofilms with compounds **11b** and **11d** for 1 h, no biofilm was detected, except when compound **11d** was used, as it did not disrupt the biofilm of *S. aureus*.

These findings highlight the importance of identifying compounds that can interfere with the early stages of biofilm formation and slow its progression. Additionally, it is important to note that the concentration of compounds required for biofilm removal is typically higher than that needed to target planktonic bacteria [46,47].

The ability of *S. aureus* and *E. coli* to form biofilms plays a critical role in the persistence and severity of the infections they cause. For example, the extracellular matrix of the biofilm prevents the diffusion of antibiotics, and the bacteria within the biofilm adopt a slower metabolic rate, which reduces the efficacy of antibiotics.

Compound **11b** appears to be more effective in terms of biofilm disruption, which is consistent with findings from similar studies investigating biofilm inhibitors. For instance, a range of small molecules, including natural and synthetic compounds, have been found to disrupt biofilm formation in *S. aureus* and *E. coli* [48].

The failure of compound **11d** to disrupt *S. aureus* biofilms may suggest that it is less effective against the specific mechanisms of biofilm formation in this Gram-positive bacterium. Research has shown that biofilm formation can vary significantly between bacterial species, and different bacteria utilize distinct strategies to form and maintain biofilms. In Gram-positive bacteria like *S. aureus*, factors such as surface protein adhesins, teichoic acids, and the accessory gene regulator (Agr) system are crucial for biofilm formation [49]. Compound **11d** might not sufficiently target these specific mechanisms in *S. aureus*, whereas compound **11b** could be more broadly effective due to its mechanism of action.

Our results also indicate that the concentrations of compounds required to disrupt biofilms are higher than those needed for planktonic bacteria. This is consistent with previous studies showing that biofilms are more resistant to antimicrobial agents than their free-living (planktonic) counterparts [50]. The dense extracellular matrix and altered metabolic state of bacteria within biofilms contribute to their enhanced resistance. In fact, treatment strategies often require significantly higher doses of antibiotics or biofilm-disrupting agents to overcome the protective barrier [51].

For example, a study by Chapman et al. (2024) [52] demonstrated that higher concentrations of vancomycin were required to treat *S. aureus* biofilm infections compared to planktonic *S. aureus* infections. Similarly, a review by Høiby et al. (2011) and Stewart et al. (2001) [50,53] emphasized the need for higher antibiotic concentrations or adjunct therapies, such as biofilm-disrupting enzymes, to effectively treat biofilm-associated infections.

## 3. Materials and Methods

### 3.1. Synthesis

Reagents and solvents were purchased from Sigma-Aldrich (St. Louis, MO, USA) and used without further purification. The reaction course and purity of the synthesized compounds were monitored by TLC using Merc Silica gel 60 F254 aluminum plates (Merck KGaA, Darmstadt, Germany).

NMR spectra were recorded on a Bruker BioSpin GmbH (^1^H 400 MHz, ^13^C 101 MHz), spectrometer (Bruker BioSpin AG, Fällanden, Switzerland). Chemical shifts were reported in *δ* ppm relative to tetramethylsilane (TMS), with the residual solvent as an internal reference (DMSO-*d*_6_, *δ* 2.50 ppm for ^1^H and δ 39.52 ppm for ^13^C). Data are reported as follows: chemical shift, multiplicity, coupling constant, Hz, integration, and assignment.

IR spectra (ν, cm^−1^) were recorded on a Perkin–Elmer Spectrum BX FT–IR spectrometer (Perkin–Elmer Inc., Waltham, MA, USA) using KBr pellets.

The C, H, and N elemental analysis was conducted on an Elemental Analyzer CE-440 (Exeter Analytical, Inc., Chelmsford, MA, USA). The results were found to be in good agreement (±0.3%) with the calculated values.

Melting points were determined in an opened capillary tube with an APA1 melting point analyzer and were uncorrected.

*1-(2-Hydroxy-5-methylphenyl)-5-oxopyrrolidine-3-carboxylic acid (* **2** *)*

A mixture of itaconic acid (12.36 g, 95 mmol) and 2-amino-4-methylphenol (**1**) (10 g, 81 mmol) was refluxed in water (40 mL) for 2 h and then cooled down; the formed crystalline precipitate was filtered off, washed with water, and purified by dissolving it in aqueous 5% sodium hydroxide solution (60 mL), filtering and acidifying the filtrate with hydrochloric acid to pH 2 to give the title compound **2** (greyish solid, yield 16.75 g, 88%, m. p. 172–173 °C).

^1^H NMR (400 MHz, DMSO-*d_6_*) δ 2.18 (s, 3H, CH_3_), 2.58–2.68 (m, 2H, COCH_2_), 3.26–3.42 (m, 1H, CH, overlaps with the peak of the H_2_O), 3.76–3.93 (m, 2H, NCH_2_), 6.79 (d, *J* = 8.7 Hz, 1H, H_Ar_), 6.72 (s, 1H, H_Ar_), 6.74 (s, 1H, H_Ar_), 9.31 (br s, 1H, OH), 12.65 (br s, 1H, COOH) ppm.

^13^C NMR (101 MHz, DMSO-*d_6_*) δ 19.96 (CH_3_), 33.74, 36.22, 50.98 (COCH_2_, CH, NCH_2_), 116.65, 125.15, 127.88, 128.31, 128.71, 150.26 (C_Ar_). 172.19, 174.41 (2 CO) ppm.

IR (KBr), ν 3302 (2 OH); 1699, 1641 (2 C=O) cm^−1^.

Calcd for C_12_H_13_NO_4_, %: C 61.27; H 5.57; N 5.95. Found, %: C 61.20; H 5.54; N 5.92.

*Methyl-1-(2-hydroxy-5-methylphenyl)-5-oxopyrrolidine-3-carboxylate (* **3** *)*

To a solution of carboxylic acid **2** (8 g, 340 mol) in methanol (130 mL), a catalytic amount of concentrated sulfuric acid (2.5 mL) was added dropwise, and the mixture was heated at reflux for 8 h. The solvent was then evaporated under reduced pressure, and the residue was neutralized with 5% sodium carbonate solution to pH 8–9. After cooling, the obtained solid was filtered off, washed with plenty of water and hexane, and recrystallized from 2-propanol (25 mL) to give the title compound **3** (greenish solid, yield 8.26 g, 97%, m. p. 116–117 °C).

^1^H NMR (400 MHz, DMSO-*d_6_*) δ 2.19 (s, 3H, CH_3_), 2.59–2.72 (m, 2H, COCH_2_), 3.43–3.52 (m, 1H, CH_2_CH), 3.68 (s, 3H, OCH_3_), 3.79–3.92 (m, 2H, NCH_2_), 6.76–6.81 (m, 1H, H_Ar_), 6.93 (s, 2H, H_Ar_), 9.31 (s, 1H, OH) ppm.

^13^C NMR (101 MHz, DMSO-*d_6_*) δ 19.94 (CH_3_), 33.60 (COCH_2_), 36.00 (CH_2_CH), 50.73 (NCH_2_), 52.13 (OCH_3_), 116.61, 125.01, 127.86, 128.34, 128.75, 150.26 (C_Ar_), 171.86, 173.30 (2 C=O) ppm.

IR (KBr), ν 3287 (OH); 1732, 1682 (2 C=O); 1213 (COOCH_3_) cm^−1^.

Calcd for C_13_H_15_NO_4_, %: C 62.64; H 6.07; N 5.62. Found, %: C 62.70; H 6.02; N 5.68.

*1-(2-Hydroxy-5-methylphenyl)-5-oxopyrrolidine-3-carbohydrazide (* **4** *)*

To a cooled solution of methyl ester **3** (2.85 g, 12 mmol) in 2-propanol (10 mL), hydrazine monohydrate (1.2 g, 24 mmol) was added, and the mixture was heated at reflux for 8 h. After completion of the reaction (TLC), the mixture was cooled to room temperature, and the obtained precipitate was filtered off, washed with propan-2-ol, and recrystallized from 2-propanol to give the title compound **4** (brownish solid, yield 1.98 g, 67%, m. p. 190–191 °C).

^1^H NMR (400 MHz, DMSO-*d_6_*) δ 2.18 (s, 3H, CH_3_), 2.45–2.63 (m, 2H, COCH_2_), 3.14–3.26 (m, 1H, CH), 3.62–3.71 (m, 1H, NCH_2_), 3.74–3.84 (m, 1H, NCH_2_), 4.31 (br s, 2H, NH_2_), 6.72–6.82 (m, 1H, H_Ar_), 6.86–7.00 (m, 2H, H_Ar_), 8.59 (s, 1H, NH), 9.29 (s, 1H, OH) ppm.

^13^C NMR (101 MHz, DMSO-*d_6_*) δ 19.96 (CH_3_), 34.42, 35.56, 51.65 (COCH_2_, CH, NCH_2_), 116.62, 125.13, 127.89, 128.39, 128.78, 150.36 (C_Ar_), 171.82, 172.43 (2 C=O) ppm.

IR (KBr), ν 3345, 3295, 3144 (OH, NH_2_, NH); 1687, 1646 (2 C=O) cm^−1^.

Calcd for C_12_H_15_N_3_O_3_, %: C 57.82; H 6.07; N 16.86. Found, %: C 55.98; H 5.93; N 16.63.

*General procedure for the preparation of compounds* **5** *and* **6**

To a solution of hydrazide **4** (0.55 g, 2.2 mmol) in methanol (10 mL), phenyl isothiocyanate (0.30 g, 2.2 mmol, 5) or phenyl isocyanate (0.26 g, 2.2 mmol, 6) was added, and the mixture was heated at reflux for 4 or 6 h, respectively. After completion of the reaction, mixture **5** was diluted with methanol (20 mL), and water was added drop-wise while stirring the mixture. The obtained oily mass was separated and dissolved in methanol and water was added dropwise. After cooling, the obtained crystalline solid was filtered off, washed with methanol, and recrystallized from 1,4-dioxane.

For mixture **6**, part of the solvent was evaporated under reduced pressure, and the obtained solid was filtered off, washed with methanol, and recrystallized from 1,4-dioxane.

*2-(1-(2-Hydroxy-5-methylphenyl)-5-oxopyrrolidine-3-carbonyl)-N-phenylhydrazine-1-carbothioamide (* **5** *)*

Brownish solid, yield 0.22 g, 69%, mp 165–166 °C.

^1^H NMR (400 MHz, DMSO-*d_6_*) δ 2.19 (s, 3H, CH_3_), 2.54–2.79 (m, 2H, COCH_2_), 3.32–3.37 (m, 1H, CH), 3.70–4.02 (m, 2H, NCH_2_), 6.80 (d, *J* = 8.1 Hz, 1H, H_Ar_), 6.68–6.73 (m, 2H, H_Ar_), 7.17 (t, *J* = 6.9 Hz, 1H, H_Ar_), 7.34 (t, *J* = 7.4 Hz, 2H, H_Ar_), 7.38–7.51 (m, 2H, H_Ar_), 9.31 (s, 1H, OH), 9.58, 9.86, 10.17 (3s, 3H, 3 NH) ppm.

^13^C NMR (101 MHz, DMSO-*d_6_*) δ 19.97 (CH_3_), 34.07, 35.44, 48.65, 51.35 (COCH_2_, CH, NCH_2_), 116.63, 125.14, 126.07, 126.26, 127.91, 128.20, 128.29, 128.35, 128.73, 139.08, 150.26 (C_Ar_), 172.24 (2 C=O), 181.04 (C=S) ppm.

IR (KBr), ν 3470, 3281, 3224, 3031 (OH, NH); 1664, 1622 (2 C=O) cm^−1^. Calcd for C_19_H_20_N_4_O_3_S, %: C 59.36; H 5.24; N, 14.57. Found, %: C 59.30; H 5.28; N 14.52.

*2-(1-(2-Hydroxy-5-methylphenyl)-5-oxopyrrolidine-3-carbonyl)-N-phenylhydrazine-1-carboxamide (* **6** *)*

White, solid, yield 1.07 g, 91%, mp 98–99 °C.

^1^H NMR (400 MHz, DMSO-*d_6_*) δ 2.19 (s, 3H, CH_3_), 2.54–2.74 (m, 2H, COCH_2_), 3.31–3.36 (m, 1H, CH), 3.70–3.96 (m, 2H, NCH_2_), 6.80 (d, *J* = 8.3 Hz, 1H, H_Ar_), 6.92–6.97 (m, 2H, H_Ar_), 7.16–7.31 (m, 2H, H_Ar_), 7.44 (d, *J* = 7.8 Hz, 2H, H_Ar_); 8.11, 8.42, 8.79, 8.98, 9.62, 9.91 (3s, 3H, 3 NH), 9.24, 9.30 (s, 1H, OH) ppm.

^13^C NMR (101 MHz, DMSO-*d_6_*) δ 19.94 (CH_3_), 34.07, 35.18, 51.43, 51.63 (COCH_2_, CH, NCH_2_), 116.61, 118.18, 118.51, 121.98, 122.38, 125.12, 127.88, 128.34, 128.69, 128.76, 139.16, 139.58, 148.33, 150.27, 154.02, 155.27 (C_Ar_), 172.21, 172.61, 172.79 (3 C=O) ppm.

IR (KBr), ν 3299, 3040, 3027, 2923 (OH, NH); 1686, 1642, 1602 (3 C=O) cm^−1^.

Calcd for C_19_H_20_N_4_O_4_, %: C 61.95; H 5.47; N 15.21. Found, %: C 61.90; H 5.48; N 15.18.

*General procedure for the preparation of compounds* **7** *and* **8**

A mixture of thiosemicarbazide **5** (0.45 g, 1.2 mmol) or semicarbazide **6** (0.3 g, 0.81 mmol) and aqueous 4% sodium hydroxide solution (11 mL) was refluxed for 3 or 4 h, respectively. The reaction mixture was cooled and neutralized with dropwise glacial acetic acid to pH 6 while cooling in an ice bath. The formed solid was filtered off (**7**) or the solvent was decanted and the remaining solid (**8**) washed with water and recrystallized from propan-2-ol to give the title compounds **7** or **8**.

*1-(2-Hydroxy-5-methylphenyl)-4-(4-phenyl-5-thioxo-4,5-dihydro-1H-1,2,4-triazol-3-yl)pyrrolidin-2-one (* **7** *)*

Brownish solid, yield 0.36 g, 83%, mp 261–262 °C.

^1^H NMR (400 MHz, DMSO-*d_6_*) δ 2.16 (s, 3H, CH_3_), 2.40–2.47 (m, 1H, COCH_2_), 2.65–2.74 (m, 1H, COCH_2_), 3.51–3.98 (m, 3H, CH, NCH_2_), 6.75 (d, *J* = 8.1 Hz, 1H, H_Ar_), 6.81–6.99 (m, 2H, H_Ar_), 7.20–7.70 (m, 5H, H_Ar_), 9.51 (br s, 1H, OH), 13.41 (br s, 1H, NH) ppm.

^13^C NMR (101 MHz, DMSO-*d_6_*) δ 19.90 (CH_3_), 29.03, 34.59, 51.39 (COCH_2_, CH, NCH_2_), 116.54, 124.73, 126.17, 127.68, 128.38, 128.56, 128.76, 129.55, 129.63, 133.77, 150.33, 152.72 (C_Ar_), 168.38 (NC), 171.45 (C=O), 182.99 (C=S) ppm.

IR (KBr), ν 3385, 3139 (OH, NH); 1667 (C=O); 1519 (C=N) cm^−1^.

Calcd for C_19_H_18_N_4_O_2_S, %: C 62.28; H 4.95; N 15.29. Found, %: C 62.35; H 4.97; N 15.32.

*5-(1-(2-Hydroxy-5-methylphenyl)-5-oxopyrrolidin-3-yl)-4-phenyl-2,4-dihydro-3H-1,2,4-triazol-3-one (* **8** *)*

Brownish solid, yield 0.11 g, 38%, mp 217–218 °C.

^1^H NMR (400 MHz, DMSO-*d_6_*) δ 2.15 (s, 3H, CH_3_), 2.40–2.47 (m, 1H, COCH_2_), 2.60–2.76 (m, 1H, COCH_2_), 3.48–3.83 (m, 3H, NCH_2_, CH), 6.77 (d, *J* = 8.1 Hz, 1H, H_Ar_), 6.81–6.94 (m, 2H, H_Ar_), 7.38–7.63 (m, 5H, H_Ar_), 11.93 (br s, 2H, OH, NH) ppm.

^13^C NMR (101 MHz, DMSO-*d_6_*) δ 19.90 (CH_3_), 29.02 (COCH_2_), 33.96 (CH_2_CH), 51.00 (NCH_2_), 116.58, 124.80, 127.30, 127.82, 128.34, 128.64, 128.96, 129.60, 132.77, 147.33, 150.63, 154.70, 168.48 (N=C, C_Ar_), 171.57, 173.63 (2 C=O) ppm.

IR (KBr), ν 3157, 3025 (OH, NH); 1697, 1663 (2 C=O); 1519 (C=N) cm^−1^.

Calcd for C_19_H_18_N_4_O_3_, %: C 65.13; H 5.18; N 15.99. Found, %: C 65.20; H 5.15; N 15.94.

*General procedure for the preparation of benzimidazoles* **9a** *–* **d**

To a cooled solution of carboxylic acid **2** (0.7 g, 3 mmol) and 4N hydrochloric acid (14 mL), the corresponding benzene-1,2-diamine (6 mmol) was added, and the mixture was heated at reflux for 24 h. Afterwards, the mixture was cooled and neutralized with 5% sodium carbonate to pH 8 in an ice bath. The formed precipitate was filtered off and washed with 5% sodium carbonate. Purification was carried out by dissolving it in 5% sodium hydroxide (15 mL), filtering and acidifying the filtrate with glacial acetic acid to pH 6, and then the obtained crystalline solid was recrystallized from propan-2-ol.

For **9b** and **9c**, purification from 5% sodium carbonate was repeated twice, and then recrystallization from propan-2-ol was performed.

*4-(1H-benzo[d]imidazol-2-yl)-1-(2-hydroxy-5-methylphenyl)pyrrolidin-2-one (* **9a** *)*

Brownish solid, yield 0.35 g, 45%, mp 239–240 °C.

^1^H NMR (400 MHz, DMSO-*d_6_*) δ 2.20 (s, 3H, CH_3_), 2.64–2.77, 2.90–3.04 (2m, 2H, COCH_2_), 3.93–4.04 (m, 2H, NCH_2_), 4.06–4.15 (m, 1H, CH), 6.83 (m, *J* = 8.2 Hz, 1H, H_Ar_), 6.90–7.03 (m, 2H, H_Ar_), 7.11–7.26 (m, 2H, H_Ar_), 7.35–7.76 (br s, 2H, H_Ar_), 10.19 (s, 1H, OH), 12.57 (s, 1H, NH) ppm.

^13^C NMR (101 MHz, DMSO-*d_6_*) δ 19.96 (CH_3_); 32.12, 36.72, 53.31 (COCH_2_, CH, NCH_2_), 111.29, 116.80, 118.17, 121.57, 122.18, 124.89, 127.76, 128.58, 129.07, 134.19, 142.29, 150.93 (C_Ar_), 172.29 (C=O) ppm.

IR (KBr), ν 3183, 3152, 3061 (OH, NH); 1663 (C=O); 1506 (C=N) cm^−1^.

Calcd for C_18_H_17_N_3_O_2_, %: C 70.34; H 5.58; N 13.67. Found, %: C 70.28; H 5.54; N 13.62.

*4-(6-Fluoro-1H-benzo[d]imidazol-2-yl)-1-(2-hydroxy-5-methylphenyl)pyrrolidin-2-one (* **9b** *)*

Brownish solid, yield 0.10 g, 11%, mp 246–247 °C.

^1^H NMR (400 MHz, DMSO-*d_6_*) δ 2.20 (s, 3H, CH_3_), 2.69–2.82, 2.88–3.03 (2m, 2H, COCH_2_), 3.92–4.03 (m, 2H, NCH_2_), 4.05–4.16 (m, 1H, CH), 6.82 (d, *J* = 8.0 Hz, 1H, H_Ar_), 6.90–7.12 (m, 3H, H_Ar_), 7.34 (d, *J* = 9.2 Hz, 1H, H_Ar_), 7.46–7.61 (m, 1H, H_Ar_), 10.10 (br s, 1H, OH), 12.48 (s, 1H, NH) ppm.

^13^C NMR (101 MHz, DMSO-*d_6_*) δ 19.93 (CH_3_); 32.04, 36.42, 53.17 (COCH_2_, CH, NCH_2_); 109.61, 109.86, 116.71, 120.67, 124.94, 127.75, 128.46, 128.92, 150.69, 157.18, 157.72, 157.26 (C_Ar_), 172.20 (C=O) ppm.

IR (KBr), ν 3159, 3137 (OH, NH); 1660 (C=O); 1507 (C=N) cm^−1^.

Calcd for C_18_H_16_FN_3_O_2_, %: C 66.45; H 4.96; N 12.92. Found, %: C 66.52; H 4.94; N 12.89.

*4-(6-Chloro-1H-benzo[d]imidazol-2-yl)-1-(2-hydroxy-5-methylphenyl)pyrrolidin-2-one (* **9c** *)*

Brownish solid, yield 0.16 g, 15%, mp 239–240 °C.

^1^H NMR (400 MHz, DMSO-*d_6_*) δ 2.20 (s, 3H, CH_3_), 2.64–2.84, 2.88–3.05 (2m, 2H, COCH_2_), 3.90–4.04 (m, 2H, NCH_2_), 4.05–4.22 (m, 1H, CH), 6.81 (d, *J* = 8.0 Hz, 1H, H_Ar_); 6.88–7.04 (m, 2H, H_Ar_), 7.18 (d, *J* = 8.5 Hz, 1H, H_Ar_), 7.53 (d, *J* = 8.5 Hz, 1H, H_Ar_), 7.59 (s, 1H, H_Ar_), 11.27 (br s, 2H, OH, NH) ppm.

^13^C NMR (101 MHz, DMSO-*d_6_*) δ 19.93 (CH_3_); 32.11, 36.46, 53.17 (COCH_2_, CH, NCH_2_); 114.60, 115.84, 116.76, 121.83, 124.96, 125.98, 127.54, 128.47, 128.91, 137.11, 139.71, 150.95 (C_Ar_), 172.18 (C=O) ppm.

IR (KBr), ν 3227, 3115 (OH, NH); 1670 (C=O); 1521 (C=N) cm^−1^.

Calcd for C_18_H_16_ClN_3_O_2_, %: C 63.25; H 4.72; N 12.29. Found, %: C 63.31; H 4.72; N 12.26.

*1-(2-Hydroxy-5-methylphenyl)-4-(6-methyl-1H-benzo[d]imidazol-2-yl)pyrrolidin-2-one (* **9d** *)*

Brown solid, yield 1.38 g, 67%, mp 247–248 °C.

^1^H NMR (400 MHz, DMSO-*d_6_*) δ 2.20 (s, 3H, CH_3_), 2.47 (s, 3H, CH_3_), 3.00 (d, *J* = 8.8 Hz, 2H, COCH_2_); 4.09 (t, *J* = 8.3 Hz, 1H, NCH_2_), 4.17 (t, *J* = 8.9 Hz, 1H, NCH_2_), 4.24–4.40 (m, 1H, CH), 6.85 (d, *J* = 8.2 Hz, 1H, H_Ar_), 6.96 (d, *J* = 8.2 Hz, 1H, H_Ar_), 7.03 (s, 1H, H_Ar_), 7.28 (d, *J* = 8.3 Hz, 1H, H_Ar_); 7.53 (s, 1H, H_Ar_), 7.62 (d, *J* = 8.3 Hz, 1H, H_Ar_), 12.43 (br s, 2H, OH, NH) ppm.

^13^C NMR (101 MHz, DMSO-*d_6_*) δ 19.96 (CH_3_), 21.15 (CH_3_), 30.65, 35.62, 52.38 (COCH_2_, CH, NCH_2_), 113.46, 113.70, 116.65, 124.65, 126.22, 127.73, 128.53, 128.99, 130.77, 132.79, 134.58, 150.61, 153.84 (C_Ar_), 171.27 (C=O) ppm.

IR (KBr), ν 3051, 2987 (OH, NH); 1654 (C=O); 1519 (C=N) cm^−1^.

Calcd for C_19_H_19_N_3_O_2_, %: C 71.01; H 5.96; N 13.08. Found, %: C 71.11; H 5.95; N 13.05.

*1-(2-Ethoxy-5-methylphenyl)-4-(1-ethyl-1H-benzo[d]imidazol-2-yl)pyrrolidin-2-one (* **10** *)*

To a solution of benzimidazole **9a** (0.15 g, 0.5 mmol) in DMF (3 mL), ground potassium hydroxide (0.06 g, 1.05 mmol) and potassium carbonate (0.05 g, 0.35 mmol) were added, and the mixture was stirred at room temperature for 15 min. Then, ethyl iodide (0.47 g, 3 mmol) was added dropwise, and the reaction was performed at room temperature for 6 h. After completion of the reaction (TLC), inorganic precipitate was filtered off, ethyl iodide was evaporated under reduced pressure, and the residue was diluted with water (10 mL) while keeping the flask in an ice bath. The formed crystalline solid was filtered off and recrystallized from the mixture of propan-2-ol and water (2:1) to give the title compound **10** (brown solid, yield 61 mg, 34%, mp 210–211 °C).

^1^H NMR (400 MHz, DMSO-*d_6_*) δ 1.19 (t, *J* = 7.1 Hz, 3H, CH_2_CH_3_), 1.36 (t, *J* = 6.9 Hz, 3H, CH_2_CH_3_), 2.24 (s, 3H, CH_3_), 2.87–3.04 (m, 2H, COCH_2_), 3.75–4.44 (m, 7H, NCH_2_, 2 CH_2_CH_3_, CH_2_CH), 6.81–7.12 (m, 3H, H_Ar_), 7.21–7.39 (m, 2H, H_Ar_), 7.57–7.74 (m, 2H, H_Ar_) ppm.

^13^C NMR (101 MHz, DMSO-*d_6_*) δ 14.53, 14.57 (2 CH_2_CH_3_), 19.93 (CH_3_), 29.46, 35.77, 36.60, 53.01, 63.79 (CH, COCH_2_, 2CH_2_CH_3_, NCH_2_), 110.76, 113.42, 116.71, 122.61, 122.85, 123.55, 124.67, 126.69, 128.70, 129.01, 129.28, 151.58, 154.31 (C_Ar_); 171.79 (C=O) ppm.

IR (KBr), ν 1692 (C=O); 1510 (C=N) cm^−1^.

Calcd for C_22_H_25_N_3_O_2_, %: C 72.70; H 6.93; N 11.56. Found, %: C 72.78; H 6.94; N 11.51.

*General procedure for the synthesis of hydrazones* **11a** *–* **d**

To a cooled solution of hydrazide **4** (0.3 g, 1.2 mmol) in propan-2-ol (13 mL), the corresponding carbaldehyde was added, and the reaction mixture was heated at reflux for 2 h. Then, the mixture was cooled, and the formed solids 11a, b, and d were filtered off, washed with 2-propanol, and recrystallized from 1,4-dioxane to give the title compounds **11a**, **b**, and **d**. For **11c**, the cooled reaction mixture was diluted with hexane (5 mL) to isolate the target compound, and then the separation procedure of **11a**, **b**, and **d** was repeated.

*1-(2-Hydroxy-5-methylphenyl)-5-oxo-N′-(thien-2-ylmethylene)pyrrolidine-3-carbohydrazide (* **11a** *)*

White solid, yield 0.30 g, 73%, mp 203–204 °C.

^1^H NMR (400 MHz, DMSO-*d_6_*) δ (*Z*/*E* isomers mixture, 65/35) 2.19 (s, 3H, CH_3_), 2.58–2.79 (m, 2H, COCH_2_), 3.33–3.38, 3.96–3.99 (2m, 1H, CH), 3.70–3.96 (s, 2H, NCH_2_), 6.70–6.84 (m, 1H, H_Ar_), 6.86–7.02 (m, 2H, H_Ar_), 7.04–7.17 (m, 1H, H_Ar_), 7.36–7.50 (m, 1H, H_Ar_), 7.53–7.70 (m, 1H, H_Ar_), 8.19, 8.42 (2s, 1H, N=CH), 9.30, 9.31 (2s, 1H, OH), 11.53, 11.55 (2s, 1H, NH) ppm.

^13^C NMR (101 MHz, DMSO-*d_6_*) δ 19.92 (CH_3_); 33.16, 34.15, 34.17, 36.17, 51.05, 51.44 (COCH_2_, CH, NCH_2_), 116.62, 125.18, 127.83, 127.97, 128.37, 128.37, 128.43, 128.64, 129.00, 130.37, 138.55, 138.88, 138.93, 142.18, 150.24, 168.61 (C_Ar_, N=CH), 172.13, 172.29, 173.27 (2 C=O) ppm.

IR (KBr), ν 3274, 3110 (OH, NH); 1692, 1653 (2x C=O); 1509 (C=N) cm^−1^.

Calcd for C_17_H_17_N_3_O_3_S, %: C 59.46; H 4.99; N 12.24. Found, %: C 59.53; H 5.03; N 12.20.

*1-(2-Hydroxy-5-methylphenyl)-N′-((5-nitrothien-2-yl)methylene)-5-oxopyrrolidine-3-carbohydrazide (* **11b** *)*

Yellowish solid, yield 0.34 g, 73%, mp 210–211 °C.

^1^H NMR (400 MHz, DMSO-*d_6_*) δ (*Z*/*E* isomers mixture, 65/35) 2.19 (s, 3H, CH_3_); 2.60–2.80 (m, 2H, COCH_2_), 3.35–3.44, 3.75–3.81 (2m, 1H, CH), 3.87–4.09 (m, 2H, NCH_2_), 6.74–6.83 (m, 1H, H_Ar_). 6.89–6.99 (m, 2H, H_Ar_). 7.53, 7.56 (2d, *J* = 4.2 Hz, 1H, H_Ar_). 8.06–8.14 (m, 1H, H_Ar_). 8.19, 8.47 (2s, 1H, N=CH), 9.30, 9.33 (2s, 1H, OH), 11.92, 11.96 (2s, 1H, NH) ppm.

^13^C NMR (101 MHz, DMSO-*d_6_*) δ 19.92 (CH_3_); 33.18, 34.04, 36.23, 50.94, 51.26 (COCH_2_, CH, NCH_2_), 116.63, 125.11, 127.85, 128.36, 128.68, 129.20, 129.77, 130.65, 136.82, 140.55, 146.57, 150.26, 150.52, 150.87, 169.27 (N=CH, C_Ar_), 172.01, 172.17, 173.93 (2 C=O) ppm.

IR (KBr), ν 3324, 3191 (OH, NH); 1703, 1675 (2 C=O); 1509 (C=N) cm^−1^.

Calcd for C_17_H_16_N_4_O_5_S, %: C 52.57; H 4.15; N 14.43. Found, %: C 52.50, H 4.17; N 14.39.

*N′-(furan-2-ylmethylene)-1-(2-hydroxy-5-methylphenyl)-5-oxopyrrolidine-3-carbohydrazide (* **11c** *)*

Brownish solid, yield 0.22 g, 57%, mp 153–154 °C.

^1^H NMR (400 MHz, DMSO-*d_6_*) δ (*Z*/*E* isomers mixture, 65/35) 2.19 (s, 3H, CH_3_), 2.58–2.79 (m, 2H, COCH_2_), 3.30–3.35, 3.98–4.07 (2m, 2H, CH), 3.72–4.06 (m, 2H, NCH_2_), 6.53–6.99 (m, 5H, H_Ar_), 7.80, 7.83 (2s, 1H, H_Ar_), 7.91, 8.10 (2s, 1H, N=CH), 9.28, 9.32 (2s, 1H, OH), 11.50, 11.54 (2s, 1H, NH) ppm.

^13^C NMR (101 MHz, DMSO-*d_6_*) δ 19.93 (CH_3_), 34.04, 34.15, 36.21, 51.12, 51.45 (COCH_2_, CH, NCH_2_), 112.13, 112.19, 113.42, 113.71, 116.59, 116.64, 125.09, 125.20, 127.86, 128.28, 128.37, 128.65, 128.70, 133.70, 136.88, 144.98, 145.98, 145.25, 149.14, 149.24, 150.23, 150.23, 150.27, 168.72 (N=CH, C_Ar_), 172.17, 172.34, 173.57 (2 C=O) ppm.

IR (KBr), ν 3263, 3153 (OH, NH); 1702, 1655 (2 C=O); 1509 (C=N) cm^−1^.

Calcd for C_17_H_17_N_3_O_4_, %: C 62.38; H 5.23; N 12.84. Found, %: C 62.43; H 5.21; N 12.88.

*1-(2-Hydroxy-5-methylphenyl)-N′-((5-nitrofuran-2-yl)methylene)-5-oxopyrrolidine-3-carbohydrazide (* **11d** *)*

Yellow solid, yield 0.28 g, 63%, mp 116–117 °C.

^1^H NMR (400 MHz, DMSO-*d_6_*) δ (*Z*/*E* isomers mixture, 65/35) 2.19 (s, 3H, CH_3_), 2.58–2.80 (m, 2H, COCH_2_), 3.38–3.45, 4.00–4.12 (2m, 1H, CH), 3.75–3.97 (m, 2H, NCH_2_), 6.2–6.84 (m, 1H, H_Ar_), 6.87–7.03 (m, 2H, H_Ar_), 7.19–7.32 (m, 1H, H_Ar_); 7.78 (d, *J* = 2.5 Hz, 1H, H_Ar_), 7.97, 8.18 (2s, 1H, N=CH), 9.29, 9.33 (2s, 1H, OH), 11.94, 11.98 (2s, 1H, NH) ppm.

^13^C NMR (101 MHz, DMSO-*d_6_*) δ 19.92 (CH_3_), 33.25, 33.94, 33.98, 36.26, 50.96, 51.27 (COCH, CH, NCH_2_), 114.60, 114.73, 115.45, 116.57, 116.62, 125.03, 125.12, 127.86, 128.34, 128.34, 128.69, 131.72, 134.90, 150.27, 151.57, 151.76, 169.33 (N=CH, C_Ar_), 172.00, 172.17, 174.16 (2 C=O) ppm.

IR (KBr), ν 3351, 3204 (OH, NH); 1673 (2 C=O); 1510 (C=N) cm^−1^.

Calcd for C_17_H_16_N_4_O_6_, %: C 54.84; H 4.33; N 15.05. Found, %: C 54.89; H 4.31; N 15.01.

*General procedure for the preparation of hydrazones* **12a** *–* **j**

To a cooled solution of hydrazide **4** (0.3 g, 1.2 mmol) in propan-2-ol (15 mL), the corresponding aromatic aldehyde (1.4 mmol) was added, and the mixture was heated at reflux for 2 (a–i) or 3 (j) h. Then, the mixture (a–i) was cooled, and the formed solid was filtered off, washed with 2-propanol, and recrystallized from the mixture of methanol and water (2:1) to give the title compounds **12a**–**i**. For **12j**, the cooled reaction mixture was diluted with hexane (20 mL), and then the obtained solid was recrystallized from the mixture of methanol and water (2:1) to give the title compound **12j**.

*N′-benzylidene-1-(2-hydroxy-5-methylphenyl)-5-oxpyrrolidine-3-carbohydrazide (* **12a** *)*

White solid, yield 0.25 g, 69%, mp 223–224 °C.

^1^H NMR (400 MHz, DMSO-*d_6_*) δ (mixture of the *Z*/*E* isomers, 60/40) 2.19 (s, 3H, CH_3_), 2.60–2.83 (m, 2H, COCH_2_), 3.36–3.43, 4.06–4.16 (m, 1H, CH), 3.73–4.05 (m, 2H, NCH_2_), 6.80 (dd, *J* = 7.6, 3.8 Hz, 1H, H_Ar_), 6.87–7.03 (m, 2H, H_Ar_), 7.33–7.55 (m, 3H, H_Ar_), 7.57–7.80 (m, 2H, H_Ar_), 8.03, 8.21 (2s, 1H, N=CH), 9.31, 9.33 (2s, 1H, OH), 11.56, 11.61 (2s, 1H, NH) ppm.

^13^C NMR (101 MHz, DMSO-*d_6_*) δ 19.94 (CH_3_), 34.15, 34.19, 36.20, 51.11, 51.46 (COCH_2_, CH, NCH_2_), 116.59, 116.65, 125.11, 125.,21, 126.82, 127.10, 127.86, 128.29, 128.37, 128.70, 128.83, 128.87, 129.90, 130.13, 134.14, 143.57, 147.02, 150.21, 150.28, 168.79, 172.19, 172.39, 173.69 (N=CH, C_Ar_, 2 C=O) ppm.

IR (KBr), ν 3374, 3227 (OH, NH); 1694, 1655 (2 C=O); 1519 (C=N) cm^−1^.

Calcd for C_19_H_19_N_3_O_3_, %: C 67.64; H 5.68; N 12.46. Found, %: C 67.69; H 5.66; N 12.42.

*N′-(2,4-difluorobenzylidene)-1-(2-hydroxy-5-methylphenyl)-5-oxopyrrolidine-3-carbohydrazide (* **12b** *)*

White solid, yield 0.26 g, 66%, mp 258–259 °C.

^1^H NMR (400 MHz, DMSO-*d_6_*) δ (mixture of the *Z*/*E* isomers, 60/40) 2.19 (s, 3H, CH_3_), 2.60–2.80 (m, 2H, COCH_2_), 3.34–3.41, 4.05–4.15 (m, 1H, CH), 3.77–4.01 (m, 2H, NCH_2_), 6.79 (dd, *J* = 7.9, 3.9 Hz, 1H, H_Ar_), 6.84–7.02 (m, 2H, H_Ar_), 7.06–7.23 (m, 1H, H_Ar_), 7.25–7.41 (m, 1H, H_Ar_), 7.83–8.01 (m, 1H, H_Ar_), 8.17, 8.38 (2s, 1H, N=CH), 9.30, 9.32 (2s, 1H, OH), 11.65, 11.74 (2s, 1H, NH) ppm.

^13^C NMR (101 MHz, DMSO-*d_6_*) δ 19.93 (CH_3_), 33.27, 34.07, 36.23, 51.00, 51.36 (COCH_2_, CH, NCH_2_), 104.26, 104.51, 104.77, 112.51, 112,54, 112.72, 112.76, 116.58, 116.64, 118.51, 118.54, 118.61, 118.64, 125.08, 125.19, 127.84, 127.89, 127.93, 127.98, 128.28, 128.36, 128.62, 128.69, 135.77, 139.04, 150.19, 150.26, 160.85 (*^1^J* = 253 Hz, C–F), 163.04 (*^1^J* = 252.5 Hz, C–F), 168.83, 172.11, 172.31, 173.77 (N=CH, C_Ar_, 2 C=O) ppm.

IR (KBr), ν 3186, 3072 (OH, NH); 1671, 1613 (2 C=O); 1520 (C=N) cm^−1^. Calcd for C_19_H_17_F_2_N_3_O_3_, %: C 61.12; H 4.59; N 11.25. Found, %: C 61.14; H 4.57; N 11.28.

*N′-(4-chlorobenzylidene)-1-(2-hydroxy-5-methylphenyl)-5-oxpyrrolidine-3-carbohydrazide (* **12c** *)*

White solid, yield 0.39 g, 87%, mp 246–247 °C.

^1^H NMR (400 MHz, DMSO-*d_6_*) δ (mixture of the *Z*/*E* isomers, 60/40) 2.19 (s, 3H, CH_3_); 2.59–2.81 (m, 2H, COCH_2_); 3.36–3.46, 4.07–4.16 (m, 1H, CH); 3.75–4.03 (m, 2H, NCH_2_); 6.80 (dd, *J* = 8.4, 3.4 Hz, 1H, H_Ar_); 6.85–7.05 (m, 2H, H_Ar_), 7.40–7.57 (m, 2H, H_Ar_); 7.64–7.82 (m, 2H, H_Ar_); 8.01, 8.20 (2s, 1H, NCH); 9.30, 9.32 (2s, 1H, OH); 11.61, 11.67 (2s, 1H, NH) ppm.

^13^C NMR (101 MHz, DMSO-*d_6_*) δ 19.93 (CH_3_), 33.32, 34.11, 36.19, 51.05, 51.42 (COCH_2_, CH, NCH_2_), 116.59, 116.64, 125.09, 125.19, 127.85, 128.27, 128.36, 128.47, 128.61, 128.72, 128.93, 133.10, 134.30, 134.56, 142.28, 145.69, 150.18, 150.27, 168.86, 172.15, 172.34, 173.75 (N=CH, C_Ar_, 2 C=O) ppm.

IR (KBr), ν 3199, 3068 (OH, NH); 1671 (2x C=O); 1520 (C=N) cm^−1^.

Calcd for C_19_H_18_ClN_3_O_3_, %: C 61.38; H 4.88; N 11.30. Found, %: C 61.42; H 4.87; N 11.26.

*N′-(4-brombenzylidene)-1-(2-hydroxy-5-methylphenyl)-5-oxpyrrolidine-3-carbohydrazide (* **12d** *)*

White solid, yield 0.45 g, 92%, mp 244–245 °C.

^1^H NMR (400 MHz, DMSO-*d_6_*) δ (mixture of the *Z*/*E* isomers, 60/40) 2.19 (s, 3H, CH_3_); 2.59–2.81 (m, 2H, COCH_2_); 3.38–3.43, 4.06–4.15 (m, 1H, CH); 3.76–4.03 (m, 2H, NCH_2_); 6.79 (dd, *J* = 7.8, 2.7 Hz, 1H, H_Ar_); 6.85–7.02 (m, 2H, H_Ar_); 7.57–7.68 (m, 4H, H_Ar_); 8.00, 8.18 (2s, 1H, N=CH); 9.31, 9.33 (2s, 1H, OH); 11.61, 11.68 (2s, 1H, NH) ppm.

^13^C NMR (101 MHz, DMSO-*d_6_*) δ 19.95 (CH_3_), 33.32, 34.13, 36.19, 51.06, 51.43 (COCH_2_, CH, NCH_2_), 116.59, 116.65, 123.09, 123.23, 125.10, 125.19, 127.87, 128.29, 128.72, 128.97, 130.24, 131.86, 132.05, 133.45, 142.41, 145.80, 150.19, 150.28, 160.78, 168.89, 172.17, 172.36, 173.77 (N=CH, C_Ar_, 2 C=O) ppm.

IR (KBr), ν 3198, 3078 (OH, NH); 1671 (2x C=O); 1519 (C=N) cm^−1^.

Calcd for C_19_H_18_BrN_3_O_3_, %: C 54.82; H 4.36; N 10.09. Found, %: C 54.90; H 4.36; N 10.13.

*1-(2-Hydroxy-5-methylphenyl)-N′-(4-nitrobenzylidene)-5-oxopyrrolidine-3-carbohydrazide (* **12e** *)*

White solid, yield 0.46 g, 98%, mp 261–262 °C.

^1^H NMR (400 MHz, DMSO-*d_6_*) δ (mixture of the *Z*/*E* isomers, 60/40) 2.19 (s, 3H, CH_3_), 2.60–2.88 (m, 2H, COCH_2_), 3.39–3.46, 4.11–4.19 (m, 1H, CH), 3.77–4.05 (m, 2H, NCH_2_), 6.70–6.86 (m, 1H, H_Ar_), 6.87–7.09 (m, 2H, H_Ar_), 7.86–8.04 (m, 2H, H_Ar_), 8.09–8.34 (m, 3H, N=CH, H_Ar_), 9.35 (s, 1H, OH), 11.84, 11.91 (2s, 1H, NH) ppm.

^13^C NMR (101 MHz, DMSO-*d_6_*) δ 20.39 (CH_3_), 33.73, 34.54, 34.61, 36.67, 51.40, 51.81 (COCH_2_, CH, NCH_2_), 117.07, 124.53, 125.51, 125.59, 128.24, 128.31, 128.49, 128.77, 128.84, 129.10, 129.18, 140.90, 140.95, 141.71, 145.02, 148.17, 148.35, 150.63, 150.72, 169.69, 172.56, 172.74, 174.54 (N=CH, C_Ar_, 2 C=O) ppm.

IR (KBr), ν 3207, 3083 (OH, NH); 1671 (2 C=O); 1520 (C=N); 1342, 1317 (NO_2_) cm^−1^.

Calcd for C_19_H_18_N_4_O_5_, %: C 59.68; H 4.75; N 14.65. Found, %: C 59.59; H 4.73; N 14.71.

*1-(2-Hydroxy-5-methylphenyl)-N′-(4-methylbenzylidene)-5-oxopyrrolidine-3-carbohydrazide (* **12f** *)*

Greyish solid, yield 0.37 g, 87%, mp 263–264 °C.

^1^H NMR (400 MHz, DMSO-*d_6_*) δ (mixture of the *Z*/*E* isomers, 60/40) 2.19 (s, 3H, NCCHCCH_3_), 2.32 (s, 3H, CH_3_), 2.58–2.82 (m, 2H, COCH_2_), 3.35–3.42, 4.06–4.16 (m, 1H, CH), 3.72–4.04 (m, 2H, NCH_2_), 6.79 (dd, *J* = 8.0, 3.6 Hz, 1H, H_Ar_), 6.84–7.10 (m, 2H, H_Ar_), 7.11–7.35 (m, 2H, H_Ar_), 7.39–7.77 (m, 2H, H_Ar_), 7.99, 8.17 (2s, 1H, N=CH), 9.30, 9.32 (2s, 1H, OH), 11.49, 11.54 (2s, 1H, NH) ppm. ^13^C NMR (101 MHz, DMSO-*d_6_*) δ 19.93 (CH_3_); 21.02 (CH_3_); 33.33, 34.12, 34.20, 36.19, 51.11, 51.47 (COCH_2_, CH, NCH_2_), 116.64, 125.11, 125.21, 126.79, 127.08, 127.84, 128.27, 128.36, 128.61, 128.69, 129.46, 131.44, 139.67, 139.95, 147.04, 150.20, 150.27, 168.65 172.19, 172.39, 173.57 (N=CH, C_Ar_, 2 C=O) ppm.

IR (KBr), ν 3210, 3083 (OH, NH); 1671 (2x C=O); 1520 (C=N) cm^−1^.

Calcd for C_20_H_21_N_3_O_3_, %: C 68.36; H 6.02; N 11.96. Found, %: C 68.42; H 6.05; N 11.91.

*N′-(4-(dimethylamino)benzylidene)-1-(2-hydroxy-5-methylphenyl)-5-oxopyrrolidine-3-carbohydrazide (* **12g** *)*

Brownish solid, yield 0.38 g, 83%, mp 262–263 °C.

^1^H NMR (400 MHz, DMSO-*d_6_*) δ (mixture of the *Z*/*E* isomers, 60/40) 2.19 (s, 3H, CH_3_), 2.58–2.83 (m, 2H, COCH_2_), 2.95, 2.96 (2s, 6H, N(CH_3_)_2_), 3.29–3.34, 4.02–4.16 (2m, 1H, CH), 3.72–4.01 (m, 2H, NCH_2_), 6.73 (t, *J* = 7.5 Hz, 2H, H_Ar_), 6.80 (dd, J = 8.2, 2.6 Hz, 1H, H_Ar_), 6.82–7.05 (m, 2H, H_Ar_), 7.34–7.67 (m, 2H, H_Ar_), 7.89, 8.06 (2s, 1H, N=CH), 9.30, 9.32 (2s, 1H, OH), 11.27, 11.31 (2s, 1H, NH) ppm.

^13^C NMR (101 MHz, DMSO-*d_6_*) δ 19.93 (CH_3_), 33.31, 34.18 36.18 (COCH_2_, CH), 39.76 (N(CH_3_)_2_), 51.12, 51.58 (NCH_2_), 111.77, 111.84, 115.58, 116.65, 121.35, 121.51, 125.12, 125.24, 127.85, 128.06, 128.26, 128.36, 128.36, 128.43, 128.60, 128.69, 144.36, 147.84, 150.20, 150.28, 151.36, 151.54 168.17, 172.27, 172.49, 173.06 (NCH, C_Ar_, 2 C=O) ppm.

IR (KBr), ν 3207, 3099 (OH, NH); 1672, 1660 (2 C=O); 1520 (C=N) cm^−1^.

Calcd for C_21_H_24_N_4_O_3_, %: C 66.30; H 6.36; N 14.73. Found, %: C 66.22; H 6.36; N 14.76.

*1-(2-Hydroxy-5-methylphenyl)-5-oxo-N′-(2,3,4-trimethoxybenzylidene)pyrrolidine-3-carbohydrazide (* **12h** *)*

White solid, yield 0.45 g, 88%, mp 226–227 °C.

^1^H NMR (400 MHz, DMSO-*d_6_*) δ (mixture of the *Z*/*E* isomers, 60/40) 2.19 (s, 3H, CH_3_), 2.58–2.81 (m, 2H, COCH_2_), 3.31–3.38, 4.03–4.14 (m, 1H, CH), 3.68–3.84 (m, 9H, 3OCH_3_), 3.84–4.02 (m, 2H, NCH_2_), 6.80 (dd, *J* = 8.0, 3.6 Hz, 1H, H_Ar_), 6.84–7.03 (m, 4H, H_Ar_), 7.56 (dd, *J* = 8.4, 6.7 Hz, 1H, H_Ar_), 8.21, 8.37 (2s, 1H, N=CH), 9.30, 9.32 (2s, 1H, OH), 11.42, 11.55 (2s, 1H, NH) ppm.

^13^C NMR (101 MHz, DMSO-*d_6_*) δ 19.93 (CH_3_), 33.29, 34.19, 36.21, 51.17, 51.49 (COCH_2_, CH, NCH_2_), 56.00, 60.48, 61.73 (3 OCH_3_), 108.74, 108.77, 116.60, 116.65, 120.10, 120.31, 120.37, 120.53, 125.12, 125.22, 127.86, 128.28, 128.34, 128.62, 128.69, 139.45, 141.53, 141.60, 142.50, 150.20, 150.27, 152.47, 152.57, 154.98, 155.21, 168.43, 172.21, 172.42, 173.34 (C_Ar_, NCH, 2 C=O) ppm.

IR (KBr), ν 3325, 3059 (OH, NH); 1700 (2 C=O); 1519 (C=N) cm^−1^.

Calcd for C_22_H_25_N_3_O_6_, %: C 61.82; H 5.90; N 9.83. Found, %: C 61.76; H 5.91; N 9.85.

*1-(2-Hydroxy-5-methylphenyl)-5-oxo-N′-(3,4,5-trimethoxybenzylidene)pyrrolidine-3-carbohydrazide (* **12i** *)*

White solid, yield 0.39 g, 75%, mp 185–186 °C.

^1^H NMR (400 MHz, DMSO-*d_6_*) δ (mixture of the *Z*/*E* isomers, 60/40) 2.18, 2.20 (2s, 3H, CH_3_), 2.62–2.85 (m, 2H, COCH_2_); 3.35–3.43 (m, 0.4H, CH), 3.68. 3.70 (2s, 3H, OCH_3_), 3.72–4.13 (m, 2H, NCH_2_ + 2s, 6H, OCH_3_ + 0.6H, CH), 6.80 (t, *J* = 6.7 Hz, 1H, H_Ar_), 6.84–7.14 (m, 4H, H_Ar_), 7.93, 8.13 (2s, 1H, N=CH), 9.28, 9.33 (2s, 1H, OH); 11.58, 11.59 (2s, 1H, NH) ppm.

^13^C NMR (101 MHz, DMSO-*d_6_*) δ 19.91 (CH_3_), 33.19, 34.22, 36.17, 51.28, 51.50 (COCH_2_, CH, NCH_2_); 55.91, 55.95, 60.11 (3 OCH_3_); 104.03, 104.32, 116.57, 116.65, 125.10, 125.17 127.86, 128.37, 128.67, 128.69, 129.65, 129.68, 139.02, 139.23, 143.27, 147.27, 147.00, 150.27, 153.19 168.77, 172.18, 172.46, 173.68 (N=CH, C_Ar_, 2 C=O) ppm.

IR (KBr), ν 3400, 3181 (OH, NH); 1663 (2 C=O); 1505 (C=N) cm^−1^.

Calcd for C_22_H_25_N_3_O_6_, %: C 61.82; H 5.90; N 9.83. Found, %: C 61.91; H 5.90; N 9.86.

*1-(2-Hydroxy-5-methylphenyl)-N′-(naphthalen-1-ylmethylene)-5-oxopyrrolidine-3-carbohydrazide (* **12j** *)*

White solid, yield 0.23 g, 50%, mp 196–197 °C.

^1^H NMR (400 MHz, DMSO-*d_6_*) δ (*Z*/*E* isomers mixture, 60/40) 2.18 (s, 3H, CH_3_), 2.68–2.86 (m, 2H, COCH_2_), 3.39–3.49, 4.15–4.24 (2m, 1H, CH), 3.80–4.11 (m, 2H, NCH_2_), 6.74–7.00 (m, 3H, H_Ar_), 7.55–7.70 (m, 3H, H_Ar_), 7.86–8.04 (m, 3H, H_Ar_), 8.59, 8.87 (2d, *J* = 8.5 Hz, 1H, H_Ar_), 8.73, 8.82 (2s, 1H, N=CH), 9.32, 9.35 (2s, 1H, OH), 11.61, 11.72 (2s, 1H, NH) ppm.

^13^C NMR (101 MHz, DMSO-*d_6_*) δ 19.93 (CH_3_), 33.31, 34.24, 36.25, 51.14, 51.48 (COCH_2_, CH, NCH_2_), 116.63, 123.48, 124.38, 125.13, 125.17, 125.54, 125.62, 126.29, 126.32, 126.98, 127.39, 127.85, 128.27, 128.34, 128.65, 128.70, 128.80, 128.89, 129.34, 129.37, 130.10, 130.37, 130.65, 133.51, 133.55, 143.30, 147.11, 150.25, 150.28, 168.81, 172.20, 172.40, 173.62 (N=CH, C_Ar_, 2 C=O) ppm.

IR (KBr), ν 3190, 3057 (OH, NH); 1663 (2 C=O); 1509 (C=N) cm^−1^.

Calcd for C_23_H_21_N_3_O_3_, %: C 71.30; H 5.46; N 10.85. Found, %: C 71.20; H 5.44; N 10.82.

*General procedure for the preparation of hydrazones* **13a** *,* **b**

A solution of hydrazide **4** (0.3 g, 1.2 mmol) in acetone (6 mL) or ethylmethyl ketone (6 mL) was heated at reflux for 4 h for both reactions. Then, the mixture was cooled, and the formed solid was filtered off and washed with acetone and recrystallized from the mixture propan-2-ol and water (1:1) to give the title compounds **13a** (white solid, 0.3 g, 86%, mp 199–200 °C) or **13b** (white solid, yield 0.28 g, 77%, mp 172–173 °C).

*1-(2-Hydroxy-5-methylpnenyl)-5-oxo-N′-(propan-2-ylidene)pyrrolidine-3-carbohydrazide (* **13a** *)*

^1^H NMR (400 MHz, DMSO-*d_6_*) δ (*Z*/*E* isomers mixture, 57/43) 1.86, 1.87 (2s, 3H, CH_3_), 1.91, 1.93 (2s, 3H, CH_3_), 2.19 (s, 3H, CH_3_), 2.53–2.76 (m, 2H, COCH_2_), 3.40–4.04 (m, 3H, NCH_2_, CH), 6.79 (dd, *J* = 8.1, 3.4 Hz, 1H, H_Ar_), 6.92 (s, 1H, H_Ar_), 6.94 (s, 1H, H_Ar_), 9.26, 9.30 (2s, 1H, OH), 10.20, 10.29 (2s, 1H, NH) ppm.

^13^C NMR (101 MHz, DMSO-*d_6_*) δ 17.11, 17.63 (CH_3_), 19.93 (CH_3_), 24.98, 25.25 (CH_3_), 34.26, 34.38 (COCH_2_), 35.76 (CH), 51.31, 51.80 (NCH_2_), 116.57, 116.63, 125.15, 125.25, 127.85, 128.24, 128.35, 128.61, 128.67, 150.21, 150.28, 151.23, 156.34, 168.74, 172.37, 172.50, 173.82 (C_Ar_, N=C, 2 C=O) ppm.

IR (KBr), ν 3184, 3051 (OH, NH); 1682, 1660 (2x C=O); 1522 (C=N) cm^−1^. Calcd for C_15_H_19_N_3_O_3_, %: C 62.27; H 6.62; N 14.52. Found, %: C 62.18; H 6.64; N 14.50.

*N′-(butan-2-ylidene)-1-(2-hydroxy-5-methylphenyl)-5-oxopyrrolidine-3-carbohydrazide (* **13b** *)*

^1^H NMR (400 MHz, DMSO-*d_6_*) δ (*Z*/*E* isomers mixture, 57/43) 0.9, 0.97, 1.02 (3t, *J* = 7.3 Hz, 3H, CH_2_CH_3_), 1.84, 1.86 (2s, 3H, CH_3_), 2.09–2.36 (m, 5H, CH_3_, CH_2_CH_3_), 2.54–2.81 (m, 2H, COCH_2_), 3.40–4.03 (m, 3H, NCH_2_, CH), 6.79 (dd, *J* = 8.1, 3.5 Hz, 1H, H_Ar_), 6.92 (s, 1H, H_Ar_), 6.94 (s, 1H, H_Ar_), 9.27, 9.31 (2s, 1H, OH), 10.17, 10.24 10.32, 10.40 (4s, 1H, NH) ppm.

^13^C NMR (101 MHz, DMSO-*d_6_*) δ 9.78, 9.81, 10.35, 10.77 (CH_2_CH_3_), 16.05, 16.11 (CH_3_), 19.92 (CH_3_), 31.43, 31.56 (CH_2_CH_3_), 33.25, 34.42 (COCH_2_), 35.81 (CH), 51.19, 51.79 (NCH_2_), 116.57, 116.62, 125.16, 125.23, 127.84, 128.26, 128.35, 128.60, 128.66, 150.21, 150.27, 154.48, 155.35, 159.82, 160.16, 168.80, 172.37, 172.51, 173.91 (C_Ar_, N=C, 2 C=O) ppm.

IR (KBr), ν 3200, 3055 (OH, NH); 1682, 1659 (2x C=O); 1522 (C=N) cm^−1^. Calcd for C_16_H_21_N_3_O_3_, %: C 63.35; H 6.98; N 13.85. Found, %: C 63.27; H 6.97; N 13.81.

*N′-(1-(4-aminophenyl)ethylidene)-1-(2-hydroxy-5-methylphenyl)-5-oxopyrrolidine-3-carbohydrazide (* **13c** *)*

To a solution of hydrazide **4** (0.30 g, 1.2 mmol) in propan-2-ol (14 mL), 4-aminoacetophenone (0.18 g, 1.3 mmol) and a catalytic amount of glacial acetic acid (3 drops) were added, and the mixture was heated at reflux for 20 h. Then, the reaction mixture was cooled, and the obtained solid was filtered off, washed with diethyl ether, and recrystallized from 1,4-dioxane.

White solid, yield 0.24 g, 55%, mp 178–179 °C.

^1^H NMR (400 MHz, DMSO-*d_6_*) δ (*Z*/*E* isomers mixture, 65/35) 2.15, 2.18, 2.19 (3s, 6H, 2 CH_3_), 2.56–2.83 (m, 2H, COCH_2_), 3.48–3.62, 4.05–4.20 (m, 1H, CH), 3.70–4.04 (m, 2H, NCH_2_), 5.43, 5.46 (2s, 2H, NH_2_), 6.56 (d, *J* = 8.2 Hz, 2H, H_Ar_), 6.79 (t, *J* = 6.9 Hz, 1H, H_Ar_), 6.93 (d, *J* = 7.1 Hz, 1H, H_Ar_), 6.96 (s, 1H, H_Ar_), 7.51 (t, *J* = 8.6 Hz, 2H, H_Ar_), 9.29, 9.33 (2s, 1H, OH), 10.34, 10.47 (2s, 1H, NH) ppm.

^13^C NMR (101 MHz, DMSO-*d_6_*) δ 13.24, 13.81 (CH_3_), 19.94 (CH_3_), 34.42, 34.53, 35.99, 51.32, 51.88 (COCH_2_, CH, NCH_2_), 112.46, 113.13, 113.28, 116.58, 116.66, 125.05, 125.18, 125.24, 127.21, 127.62, 127.86, 128.26, 128.38, 128.61, 128.68, 130.58, 149.00, 149.98, 150.03, 150.20, 150.24, 150.29, 153.63, 153.79, 168.87 (N=C, C_Ar_), 172.42, 172.58, 174.05 (2 C=O).

IR (KBr), ν 3461, 3360, 3176 (OH, NH_2_, NH); 1669 (2 C=O); 1519 (C=N) cm^−1^. Calcd for C_20_H_22_N_4_O_3_, %: C 65.56; H 6.05; N 15.29. Found, %: C 65.61; H 6.07; N 15.21.

*1-(2-Hydroxy-5-methylphenyl)-5-oxo-N′-(2-oxoindolin-3-ylidene)pyrrolidine-3-carbohydrazide (* **14** *)*

To a cooled solution of hydrazide **4** (0.5 g, 2 mmol) in methanol (10 mL), isatin (0.44 g, 3 mmol) was added, followed by the dripping of a catalytic amount of glacial acetic acid (3 drops), and the reaction mixture was heated at reflux for 3 h. Then, the mixture was cooled, and the obtained solid was filtered off, washed with propan-2-ol, and recrystallized from 2-propanol to give the title compound **14** (yellow solid, yield 0.36 g, 48%, mp 210–211 °C).

^1^H NMR (400 MHz, DMSO-*d_6_*) δ 2.19 (s, 3H, CH_3_), 2.61–2.84 (m, 2H, COCH_2_), 3.80–4.02 (m, 2H, NCH_2_), 4.16 (br s, 1H, CH), 6.79 (d, *J* = 8.1 Hz, 1H, H_Ar_), 6.86–6.95 (m, 2H, H_Ar_), 6.96 (s, 1H, H_Ar_), 7.05 (t, *J* = 7.0 Hz, 1H, H_Ar_), 7.38 (t, *J* = 7.6 Hz, 1H, H_Ar_), 8.12 (s, 1H, H_Ar_), 9.33 (s, 1H, OH), 10.81, 11.33 (2s, 2H, 2 NH) ppm.

^13^C NMR (101 MHz, DMSO-*d_6_*) δ 19.94 (CH_3_), 33.70, 34.71, 51.27 (COCH_2_, CH, NCH_2_); 110.63, 115.23, 116.62, 121.71, 125.12, 126.28, 127.87, 128.39, 128.72, 132.67, 143.84, 150.31, 164.60 (N=C, C_Ar_), 172.11 (3 C=O) ppm.

IR (KBr), ν 3208, 3199, 3139 (OH, NH); 1732, 1717, 1691 (3 C=O); 1510 (C=N) cm^−1^.

Calcd for C_20_H_18_N_4_O_4_, %: C 63.49; H, 4.80; N 14.81. Found, %: C 63.41; H 4.82; N 14.79.

*4-(3,5-Dimethyl-1H-pyrazol-1-yl)-1-(2-hydroxy-5-methylphenyl)pyrrolidin-2-one (* **15** *)*

To a cooled solution of hydrazide **4** (0.5 g, 2 mmol) in propan-2-ol (15 mL), pentane-2,4-dione (0.24 g, 2.4 mmol) and a catalytic amount of concentrated hydrochloric acid (3 drops) were added, and the mixture was heated at reflux for 2 h. When completed, the mixture was cooled, and the obtained crystalline solid was filtered off, washed with propan-2-ol, and recrystallized from 1,4-dioxane to give the title compound **15** (white solid, yield 0.29 g, 46%, mp 170–171 °C).

^1^H NMR (400 MHz, DMSO-*d_6_*) δ 2.19 (s, 6H, 2 CH_3_), 2.50 (s, 3H, CH_3_, overlaps with the DMSO-*d_6_*); 2.68–2.81 (m, 2H, COCH_2_), 3.79–4.09 (m, 2H, NCH_2_), 4.43–4.53 (m, 1H, CH), 6.22 (s, 1H, H_Pyr_), 6.79 (d, *J* = 8.1 Hz, 1H, H_Ar_), 6.93 (d, *J* = 8.3 Hz, 1H, H_Ar_), 6.96 (s, 1H, H_Ar_); 9.32 (s, 1H, OH) ppm.

^13^C NMR (101 MHz, DMSO-*d_6_*) δ 13.54, 14.07 (2 CH_3 Pyr_), 19.91, 33.47, 36.69, 51.22 (COCH_2_, CH, NCH_2_), 111.50 (CH_Pyr_), 116.57, 124.97, 127.82, 128.39, 128.70, 143.81, 150.26, 152.06 (C_Ar_), 171.80, 172.61 (2 C=O) ppm.

IR (KBr), ν 3211 (OH); 1729, 1664 (2 C=O); 1512 (C=N) cm^−1^.

Calcd for C_17_H_19_N_3_O_3_, %: C 65.16; H 6.11; N 13.41. Found, %: C 65.21; H 6.11; N 13.46.

*N-(2,5-dimethyl-1H-pyrrol-1-yl)-1-(2-hydroxy-5-methylphenyl)-5-oxopyrrolidine-3-carboxamide (* **16** *)*

To a solution of hydrazide **4** (0.55 g, 2.2 mmol) in propan-2-ol (22 mL), hexane-2,5-dione (0.44 g, 3.9 mmol) and glacial acetic acid (3 drops) were added dropwise, and the mixture was heated at reflux for 4 h. Then, the reaction mixture was cooled, and the formed crystalline solid was filtered off, washed with propan-2-ol, and recrystallized from 1,4-dioxane to give the title compound **16** (brownish solid, yield 0.34 g, 48%, mp 208–1209 °C).

^1^H NMR (400 MHz, DMSO-*d_6_*) δ 1.97, 2.00 (2s, 6H, 2 CH_3_), 2.19 (s, 3H, NCCHCCH_3_), 2.61–2.78 (m, 2H, COCH_2_), 3.43–3.57 (m, 1H, CH), 3.77–3.87 (m, 1H, NCH_2_), 3.89–4.07 (m, 1H, NCH_2_), 5.65 (s, 2H, H_Pyrr_), 6.80 (d, *J* = 7.9 Hz, 1H, H_Ar_), 6.87–7.02 (m, 2H, H_Ar_), 9.35 (s, 1H, OH), 10.86 (s, 1H, NH) ppm.

^13^C NMR (101 MHz, DMSO-*d_6_*) δ 10.92, 10.96 (2 CH_3_), 19.93 (CH_3_), 33.82, 35.28, 51.41 (COCH_2_, CH, NCH_2_), 103.08 (CH_Pyrr_), 116.55, 124.99, 126.74, 126.76, 127.85, 128.46, 128.75, 150.32 (C_Ar_), 171.89, 171.99 (2 C=O) ppm.

IR (KBr), ν 3265, 3117 (OH, NH); 1667, 1608 (2 C=O) cm^−1^.

Calcd for C_18_H_21_N_3_O_3_, %: C 66.04; H 6.47; N 12.84. Found, %: C 66.11; H 6.50; N 12.79.

### 3.2. Determination of Antimicrobial Activity

#### 3.2.1. Preparation of Bacterial Inoculum

Clinically important reference bacterial strains were sourced from the American Type Culture Collection (ATCC). In this study, the Gram-positive bacteria *Staphylococcus aureus* subsp. *aureus* (ATCC 9144), *Listeria monocytogenes* (ATCC 35152), and *Bacillus cereus* (ATCC 11778), as well as the Gram-negative bacterium *Escherichia coli* (ATCC 8739), were used to evaluate the compounds in vitro.

Bacteria were cultured on Tryptone Soya Agar (OXOID, Hampshire, UK) 24 h prior to testing. A turbidity equivalent to 0.5 McFarland standard (10^8^ CFU/mL) was prepared from the cultured bacterial suspensions.

#### 3.2.2. Determination of Minimum Inhibitory Concentration (MIC)

The broth microdilution method [54] was used to study the antibacterial activity of the derivatives. Bacterial growth was assessed in 96-well microplates (OXOID, Hampshire, UK) in Muller–Hinton (MH) broth. Serial two-fold dilutions of the compounds were used to determine the MIC, ranging from 500 µg/mL to 0.244 µg/mL concentrations. The concentration of bacteria used for the study was 5 × 10^5^ CFU/mL. Each well contained 100 µL of the mixture (different concentrations of compounds and bacteria at 5 × 10^4^ CFU). The sterility of the medium, the growth of the tested bacteria, and the sensitivity to the antibiotics oxallin, ampicillin, and cefuroxime were also controlled. Microtitration plates with the test mixture were incubated at 37 °C for 24 h. The MIC was determined as the lowest concentration of the compound at which no bacterial growth (turbidity) could be seen in the plate wells. Cefuroxime was used as a control (C) for antibacterial activity screening. The experiment was performed in triplicate.

#### 3.2.3. Determination of Minimum Bactericidal Concentration (MBC)

The MBC was considered as the lowest concentration of the compound causing a ≥3 log_10_ reduction (≥99.9% kill) in the number (5 × 10^4^ CFU/100 µL) of bacteria [54]. Ten microliters of mixture was taken from the well in which the MIC value was determined and from up to three wells in which the concentration of the compound was 2, 4, and 8 times higher. The tested mixtures were spread on Mueller–Hinton agar. The growth of bacteria and number of colonies were evaluated after 24 h of incubation at 37 °C. The MBC was considered as the lowest concentration of a compound when bacteria did not grow or formed up to 5 colonies. The experiment was conducted in triplicate.

#### 3.2.4. Biofilm Assay

Biofilm formation was investigated using the tube adherence test, a qualitative assay originally described by Christensen et al. (1985) [14]. The bacterial strains included in the study were *Staphylococcus aureus* subsp. *aureus* (ATCC 9144), *Listeria monocytogenes* (ATCC 7644), *Bacillus cereus* (ATCC 11778), and the Gram-negative bacterium *Escherichia coli* (ATCC 8739). Following this assay, biofilm formation was confirmed for *S. aureus* and *E. coli*. Based on these findings and previous studies demonstrating antibacterial activity, two compounds, **11b** and **11d**, were selected to evaluate their capacity to disrupt biofilms formed by the Gram-positive coccus *S. aureus* and the Gram-negative rod *E. coli*.

A loopful of test bacteria was inoculated into 10 mL of Tryptic Soy Broth (Liofilchem, Roseto degli Abruzzi, Italy) containing 1% glucose in test tubes. The tubes were incubated for 24 h at 37 °C. After incubation, the tubes were decanted, washed with phosphate-buffered saline (PBS, pH 7.3), and dried.

The ability of compounds **11b** and **11d** to disrupt biofilms was evaluated at a concentration of 10.0 µg/mL. The compounds were added to the tubes and incubated for 1 h before staining. The tubes were then stained with 0.1% crystal violet. Excess stain was removed by washing with deionized water. The tubes were dried in an inverted position.

Biofilm formation was scored based on the results from the control strains. Bacteria were considered biofilm-producing when a visible layer of biofilm was observed on the walls of the tube. The scoring for biofilm formation was as follows: (1) negative, (2) weak positive, (3) moderate positive, and (4) strong positive. The experiment was conducted in triplicate [55].

## 4. Conclusions

In the current study, a library of 1-(2-hydroxy-5-methylphenyl)-5-oxopyrrolidine-3-carboxylic acid derivatives were obtained, characterized, and assessed for their antibacterial activity against *Staphylococcus aureus*, *Listeria monocytogenes*, *Bacillus cereus*, and *Escherichia coli* bacteria strains. In summary, hydrazones **11b** and **11d** demonstrate the highest antibacterial properties and show promise as potential biofilm-disrupting agents. The ability of compound **11b** to disrupt biofilms of both *S. aureus* and *E. coli* suggests that it could be a valuable candidate for further investigation. However, the differential activity of compound **11d** against *S. aureus* biofilms indicates that its mechanism of action may be species-specific, highlighting the complexity of biofilm-targeting strategies. Further studies examining the mechanism of action of these compounds and optimizing their concentrations could provide valuable insights into their potential as adjunct therapies for treating chronic infections caused by biofilm-forming pathogens.

## Figures and Tables

**Table 1:** Structures of the synthesized compounds and their yields.

Structure No.	Chemical Structure	Yield, %	Structure No.	Chemical Structure	Yield, %
**2**	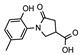	88	**12a**	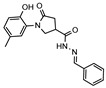	69
**3**	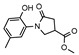	97	**12b**	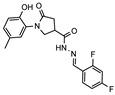	66
**4**	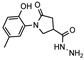	67	**12c**	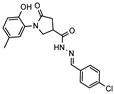	87
**5**	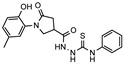	69	**12d**	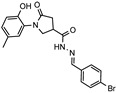	92
**6**	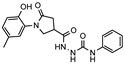	91	**12e**	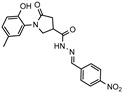	98
**7**	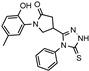	83	**12f**	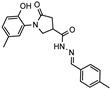	87
**8**	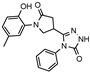	38	**12g**	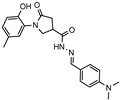	83
**9a**	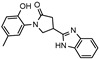	45	**12h**	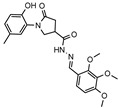	88
**9b**	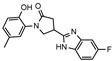	11	**12i**	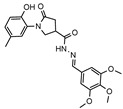	75
**9c**	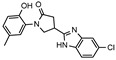	15	**12j**	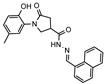	50
**9d**	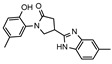	67	**13a**	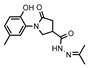	86
**10**	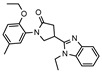	34	**13b**	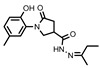	77
**11a**	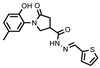	73	**13c**	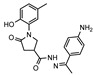	55
**11b**	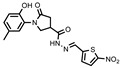	73	**14**	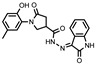	48
**11c**	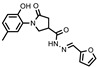	57	**15**	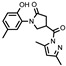	46
**11d**	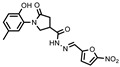	63	**16**	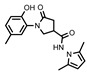	48

## Data Availability

Data are contained within the article.

## Supplementary Materials

The following supporting information can be downloaded at: https://www.mdpi.com/article/10.3390/molecules30122639/s1, Figure S1: **^1^**H NMR of compound **2**; Figure S2: **^13^**C NMR of compound **2**; Figure S3: **^1^**H NMR of compound **3**; Figure S4: **^13^**C NMR of compound **3**; Figure S5: **^1^**H NMR of compound **4**; Figure S6; **^13^**C NMR of compound **4**; Figure S7: **^1^**H NMR of compound **5**; Figure S8: **^13^**C NMR of compound **5**; Figure S9: **^1^**H NMR of compound **6**; Figure S10: **^13^**C NMR of compound **6**; Figure S11: **^1^**H NMR of compound **7**; Figure S12: **^13^**C NMR of compound **7**; Figure S13: **^1^**H NMR of compound **8**; Figure S14: **^13^**C NMR of compound **8**; Figure S15: **^1^**H NMR of compound **9a**; Figure S16: **^13^**C NMR of compound **9a**; Figure S17: **^1^**H NMR of compound **9b**; Figure S18: **^13^**C NMR of compound **9b**; Figure S19: **^1^**H NMR of compound **9c**; Figure S20: **^13^**C NMR of compound **9c**; Figure S21: **^1^**H NMR of compound **9d**; Figure S22; **^13^**C NMR of compound **89**; Figure S23; **^1^**H NMR of compound **10**; Figure S24: **^13^**C NMR of compound **10**; Figure S25: **^1^**H NMR of compound **11a**; Figure S26: **^13^**C NMR of compound **11a**; Figure S27: **^1^**H NMR of compound **11b**; Figure S28: **^13^**C NMR of compound **11b**; Figure S29: **^1^**H NMR of compound **11c**; Figure S30: **^13^**C NMR of compound **11c**; Figure S31: **^1^**H NMR of compound **11d**; Figure S32: **^13^**C NMR of compound **11d**; Figure S33: **^1^**H NMR of compound **12a**; Figure S34: **^13^**C NMR of compound **12a**; Figure S35: **^1^**H NMR of compound **12b**; Figure S36: **^13^**C NMR of compound **12b**; Figure S37: **^1^**H NMR of compound **12c**; Figure S38: **^13^**C NMR of compound **12c**; Figure S39: **^1^**H NMR of compound **12d**; Figure S40: **^13^**C NMR of compound **12d**; Figure S41: **^1^**H NMR of compound **12e**; Figure S42: **^13^**C NMR of compound **12e**; Figure S43: **^1^**H NMR of compound **12f**; Figure S44: **^13^**C NMR of compound **12f**; Figure S45: **^1^**H NMR of compound **12g**; Figure S46: **^13^**C NMR of compound **12g**; Figure S47: **^1^**H NMR of compound **12h**; Figure S48: **^13^**C NMR of compound **12h**; Figure S49: **^1^**H NMR of compound **12i**; Figure S50: **^13^**C NMR of compound **12i**; Figure S51: **^1^**H NMR of compound **12j**; Figure S52: **^13^**C NMR of compound **12j**; Figure S53: **^1^**H NMR of compound **13a**; Figure S54: **^13^**C NMR of compound **13a**; Figure S55: **^1^**H NMR of compound **13b**; Figure S56: **^13^**C NMR of compound **13b**; Figure S57: **^1^**H NMR of compound **13c**; Figure S58: **^13^**C NMR of compound **13c**; Figure S59: **^1^**H NMR of compound **14**; Figure S60: **^13^**C NMR of compound **14**; Figure S61: **^1^**H NMR of compound **15**; Figure S62: **^13^**C NMR of compound **15**; Figure S63: **^1^**H NMR of compound **16**; Figure S64: **^13^**C NMR of compound **16**; Figure S65: IR spectrum of compound **2**; Figure S66: IR spectrum of compound **3**; Figure S67: IR spectrum of compound **4**; Figure S68: IR spectrum of compound **5**; Figure S69: IR spectrum of compound **6**; Figure S70: IR spectrum of compound **7**; Figure S71: IR spectrum of compound **8**; Figure S72: IR spectrum of compound **9a**; Figure S73: IR spectrum of compound **9b**; Figure S74: IR spectrum of compound **9c**; Figure S75: IR spectrum of compound **9d**; Figure S76: IR spectrum of compound **10**; Figure S77: IR spectrum of compound **11a**; Figure S78: IR spectrum of compound **11b**; Figure S79: IR spectrum of compound **11c**; Figure S80: IR spectrum of compound **11d**; Figure S81: IR spectrum of compound **12a**; Figure S82: IR spectrum of compound **12b**; Figure S83: IR spectrum of compound **12c**; Figure S84: IR spectrum of compound **12d**; Figure S85: IR spectrum of compound **12e**; Figure S86: IR spectrum of compound **12f**; Figure S87: IR spectrum of compound **12g**; Figure S88: IR spectrum of compound **12h**; Figure S89: IR spectrum of compound **12i**; Figure S90: IR spectrum of compound **12j**; Figure S91: IR spectrum of compound **13a**; Figure S92: IR spectrum of compound **13b**; Figure S93: IR spectrum of compound **13c**; Figure S94: IR spectrum of compound **14**; Figure S95: IR spectrum of compound **15**; Figure S96: IR spectrum of compound **16**.

## Abbreviations

The following abbreviations are used in this manuscript:

O	Oxacillin
A	Ampicillin
C	Cefuroxime
MIC	Minimum Inhibitory Concentration
MBC	Minimum Bactericidal Concentration
*S. aureus*	*Staphylococcus aureus* subsp. *aureus*
