# Supplementary material for: Synthesis of 1-(2-Hydroxy-5-methylphenyl)-5-oxopyrrolidine-3-carboxylic Acid Derivatives as a Promising Scaffold Against Disease-Causing Bacteria Relevant to Public Health
Source: Molecules. 2025 Jun 18;30(12):2639. doi: 10.3390/molecules30122639 (PMC12196313; doi:10.3390/molecules30122639)
Supplement: Supplementary file 1 [file molecules-30-02639-s001.zip › molecules-3642506-supplementary.pdf]

Supplementary Materials

Synthesis of 1-(2-Hydroxy-5-methylphenyl)-5-oxopyrrolidine-3-carboxylic Acid Derivatives as a Promising Scaffold Against Disease-Causing Bacteria Relevant to Public Health

NMR Spectra (compounds 2–16, all in DMSO-d_6, Figures S1–S64)

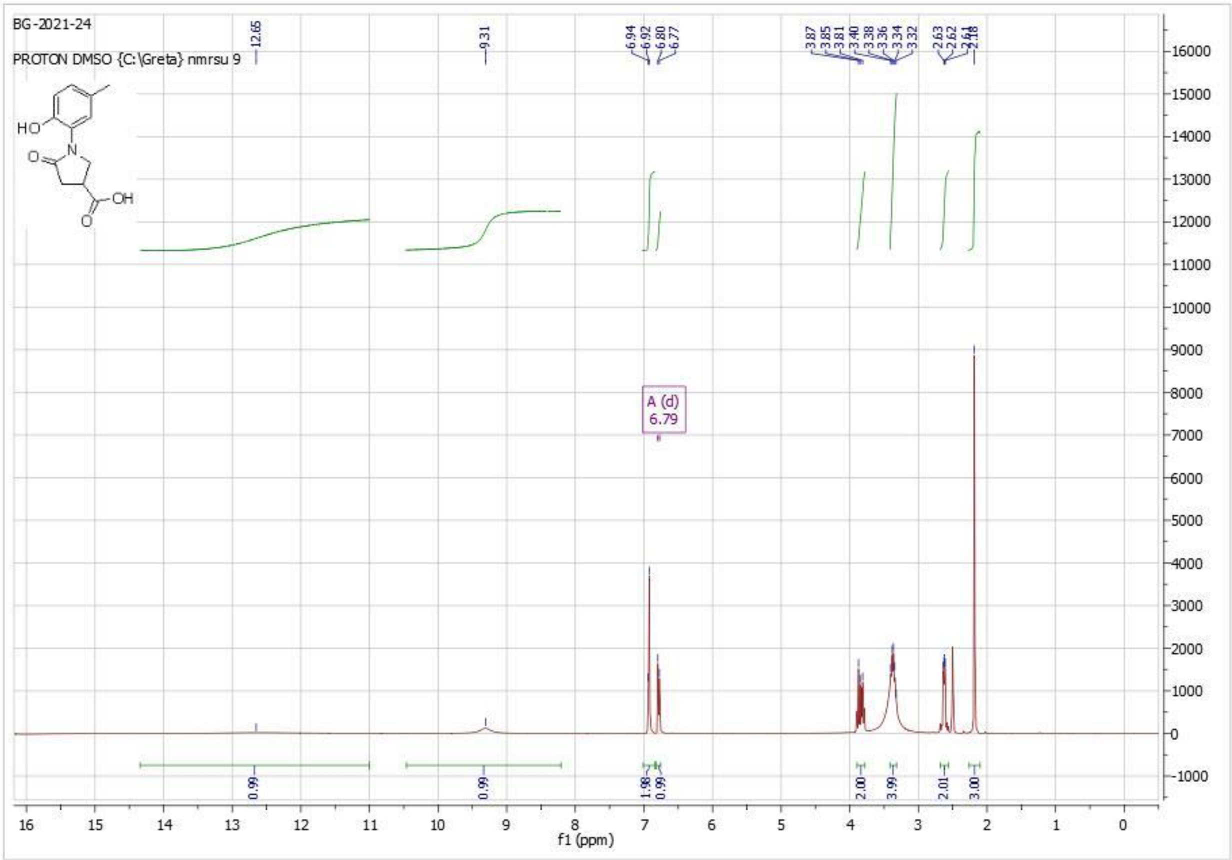

Figure S1. ^{1}H NMR of compound **2.**

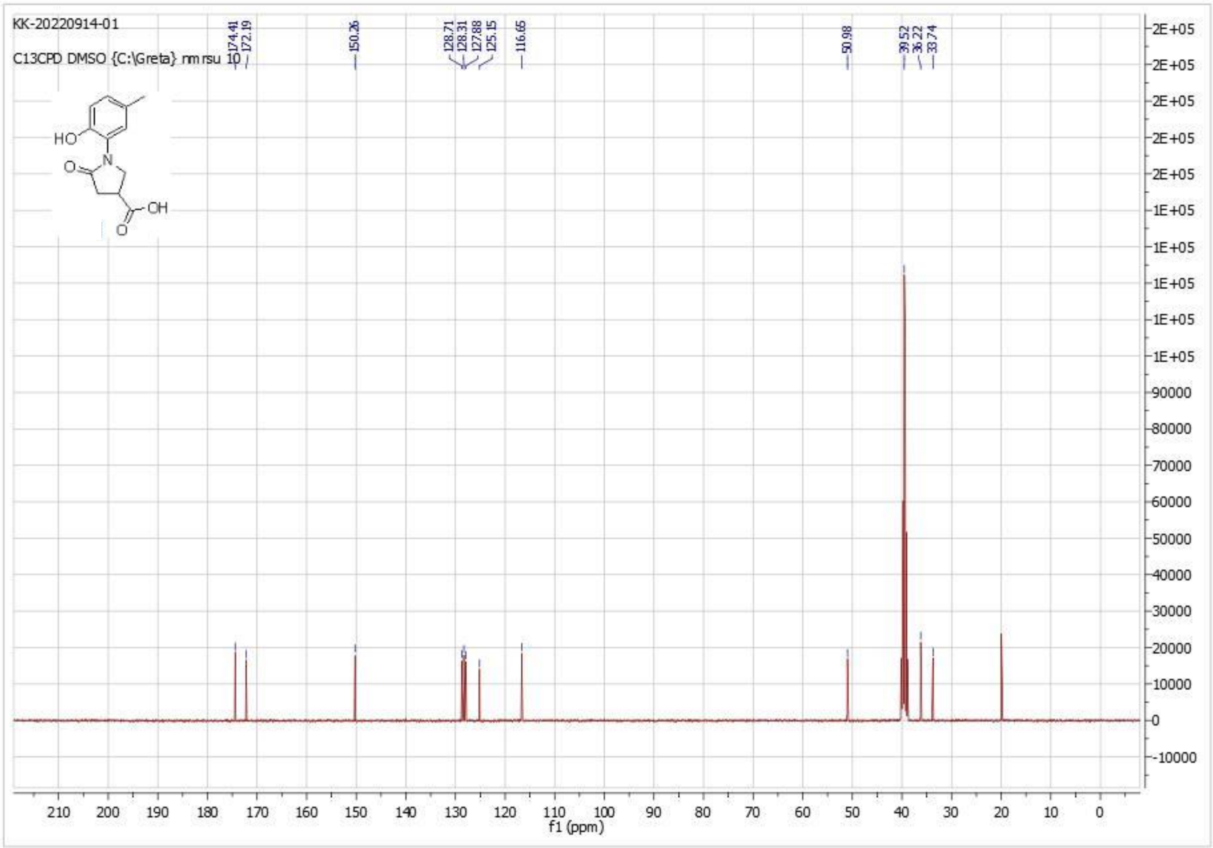

Figure S2. ^{13}C NMR of compound **2.**

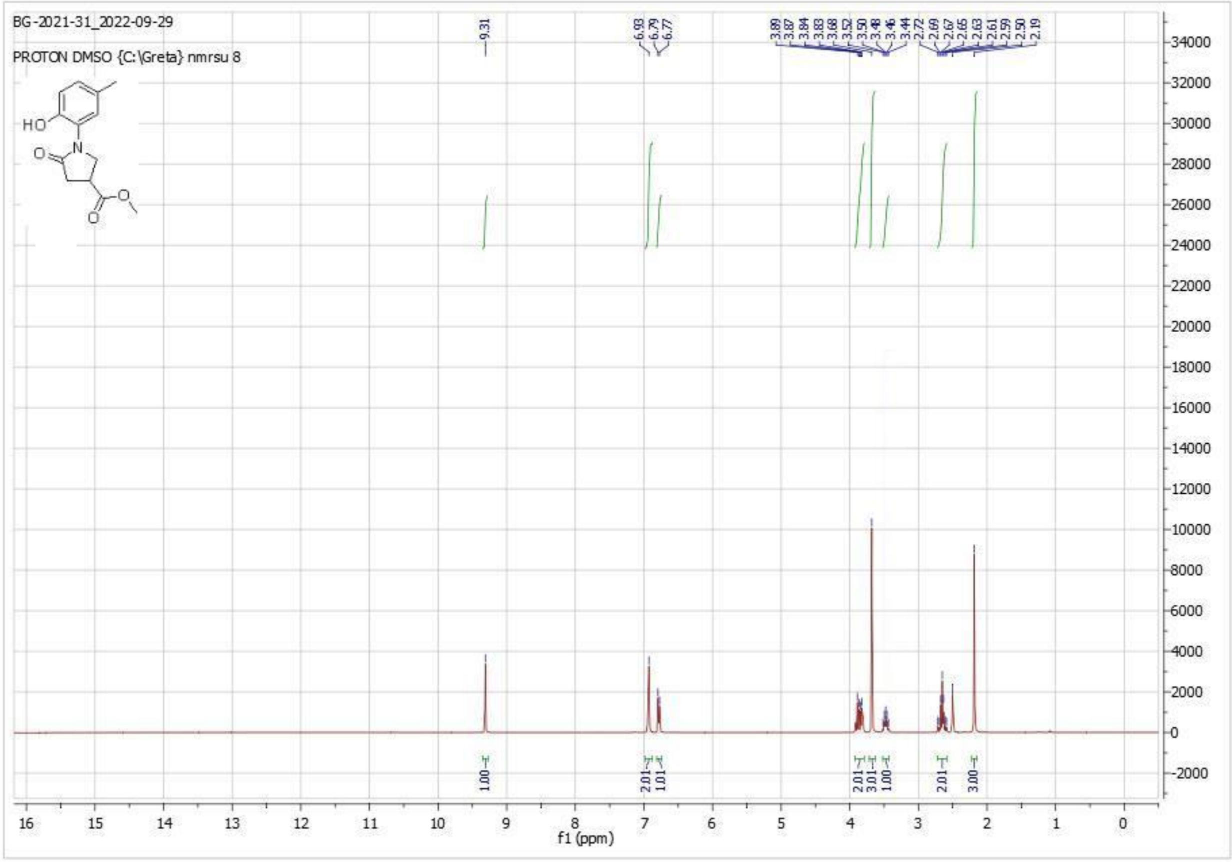

Figure S3. ^{1}H NMR of compound **3.**

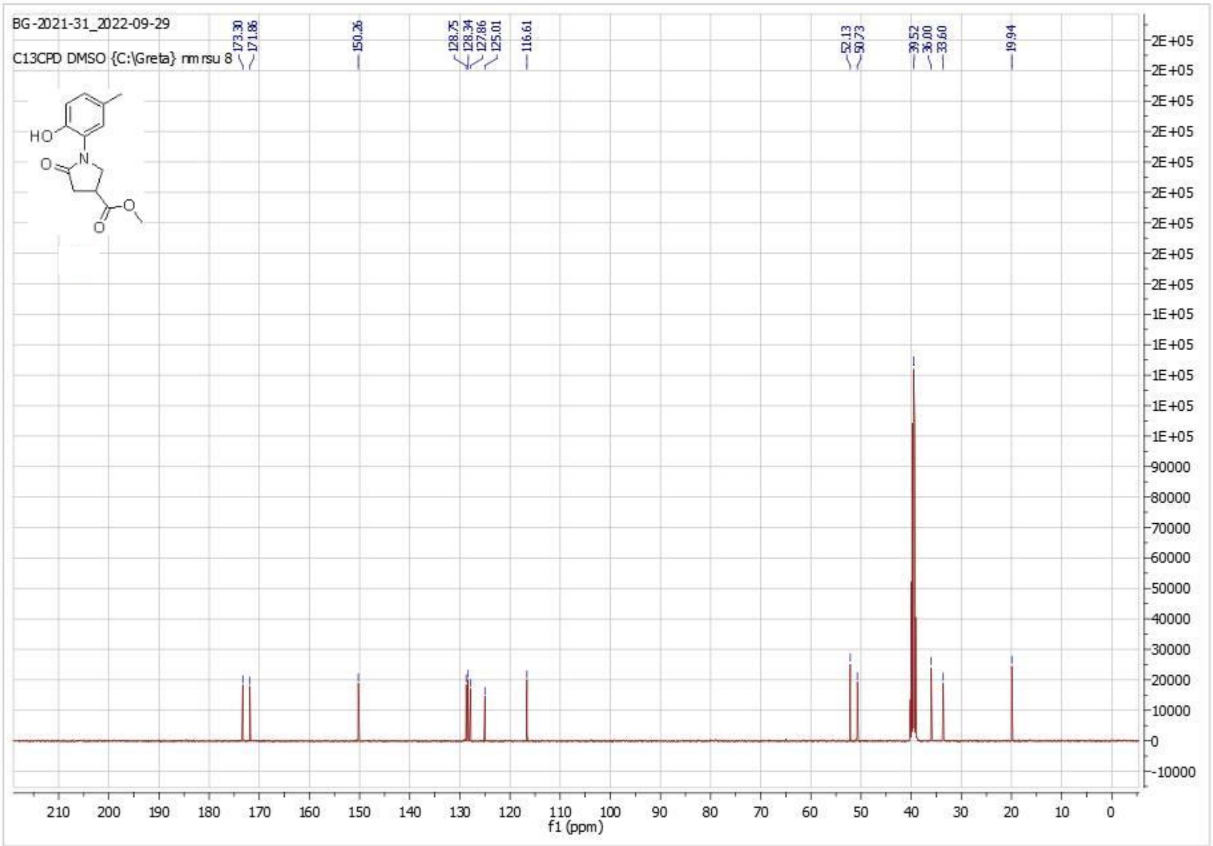

Figure S4. ^{13}C NMR of compound **3.**

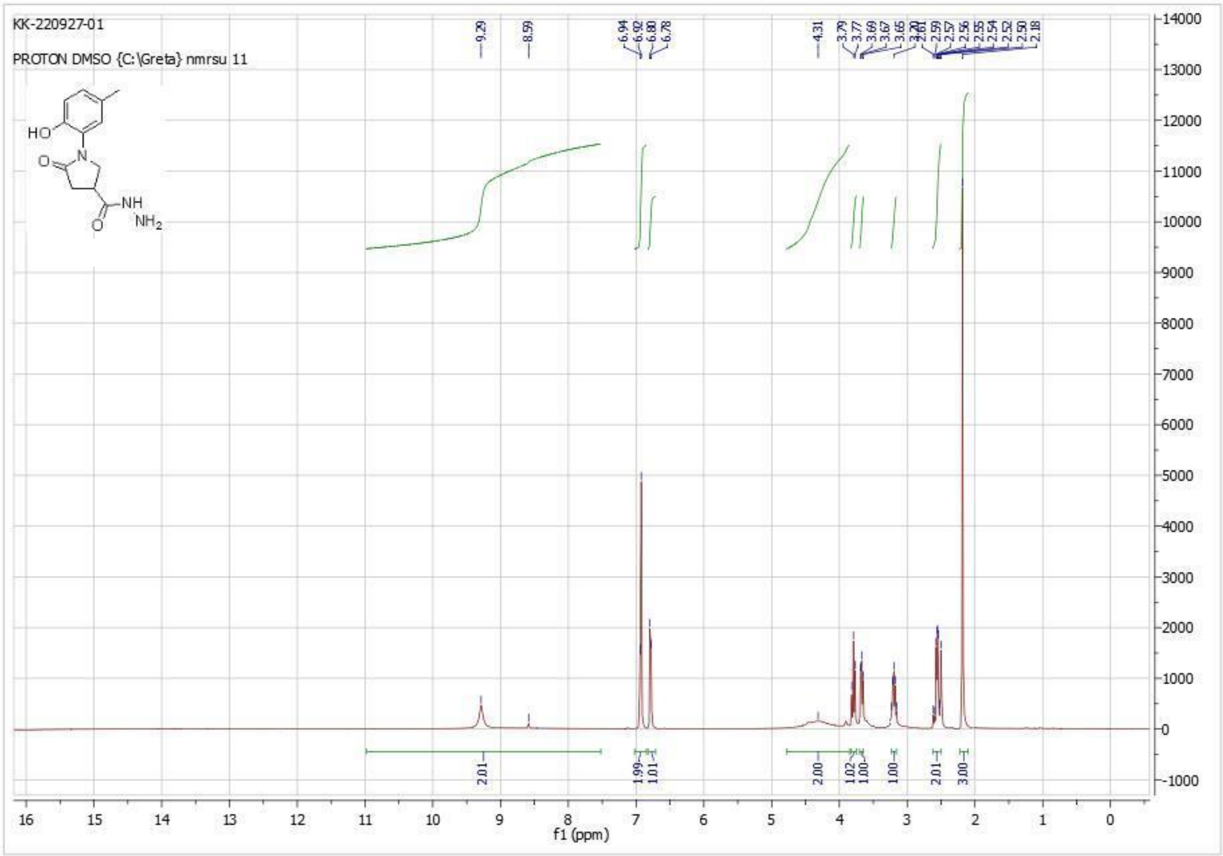

Figure S5. ^{1}H NMR of compound **4.**

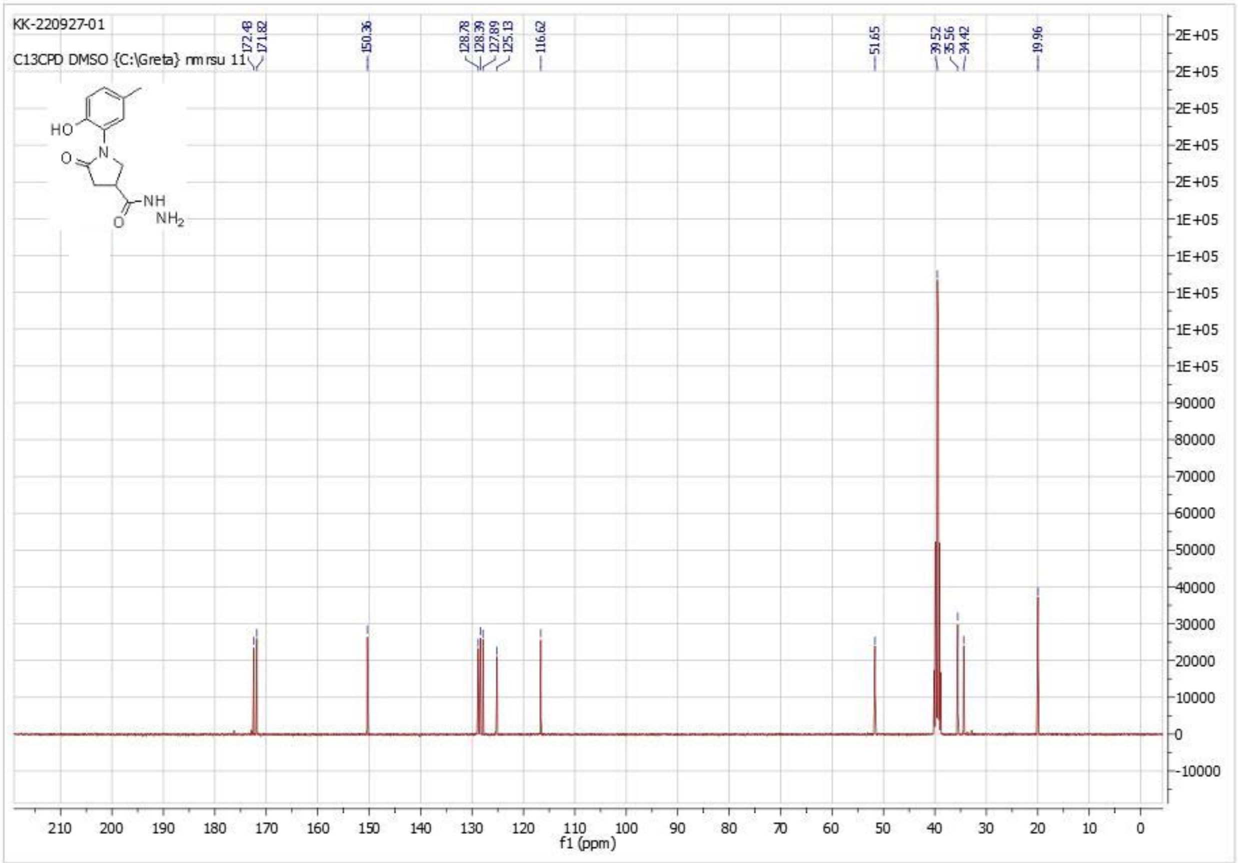

Figure S6. ^{13}C NMR of compound **4.**

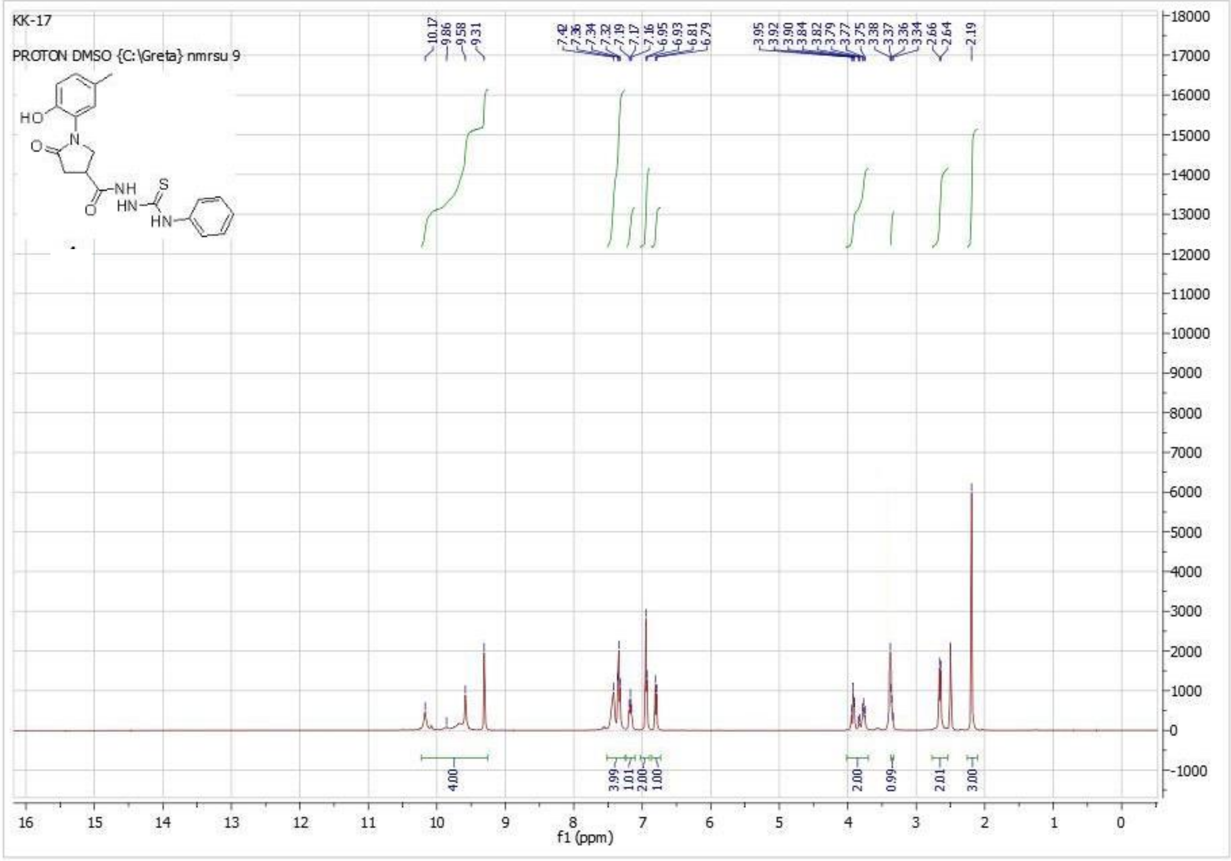

Figure S7. ^{1}H NMR of compound **5.**

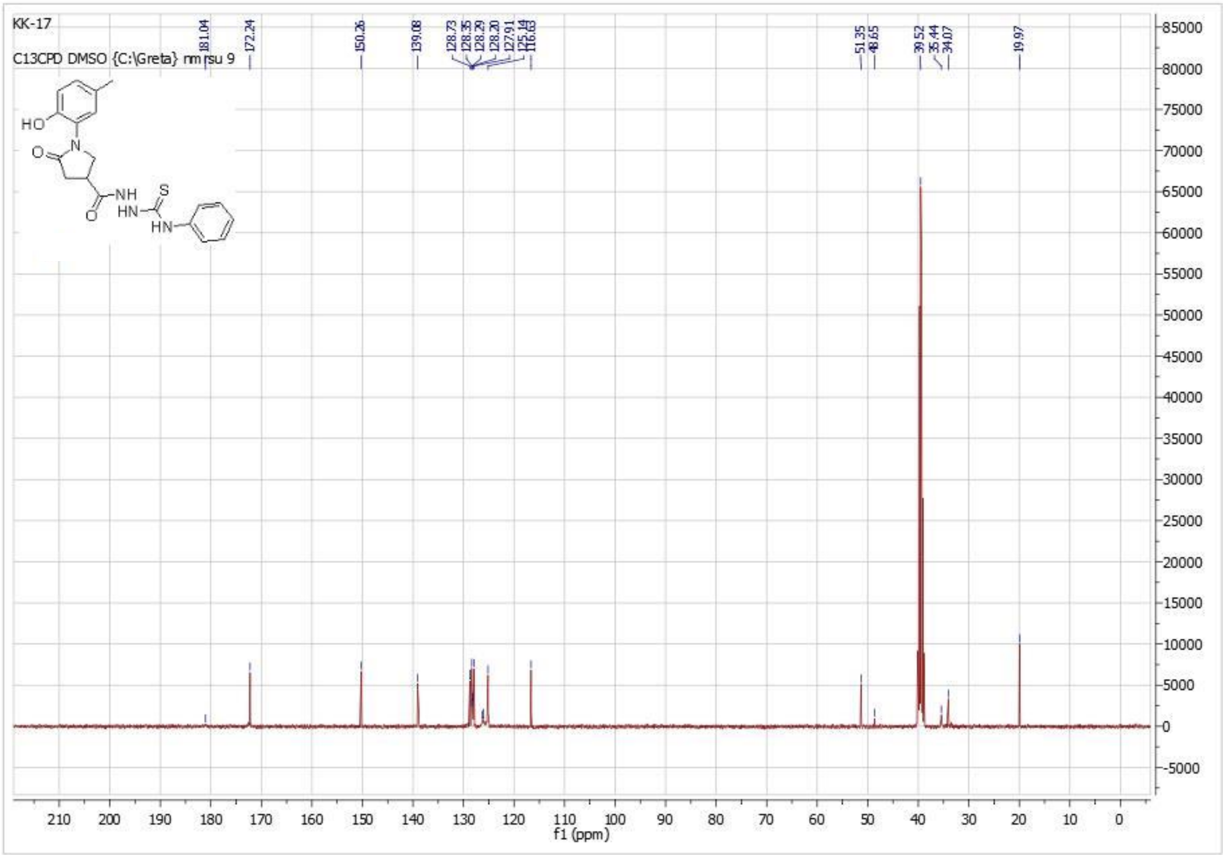

Figure S8. ^{13}C NMR of compound **5.**

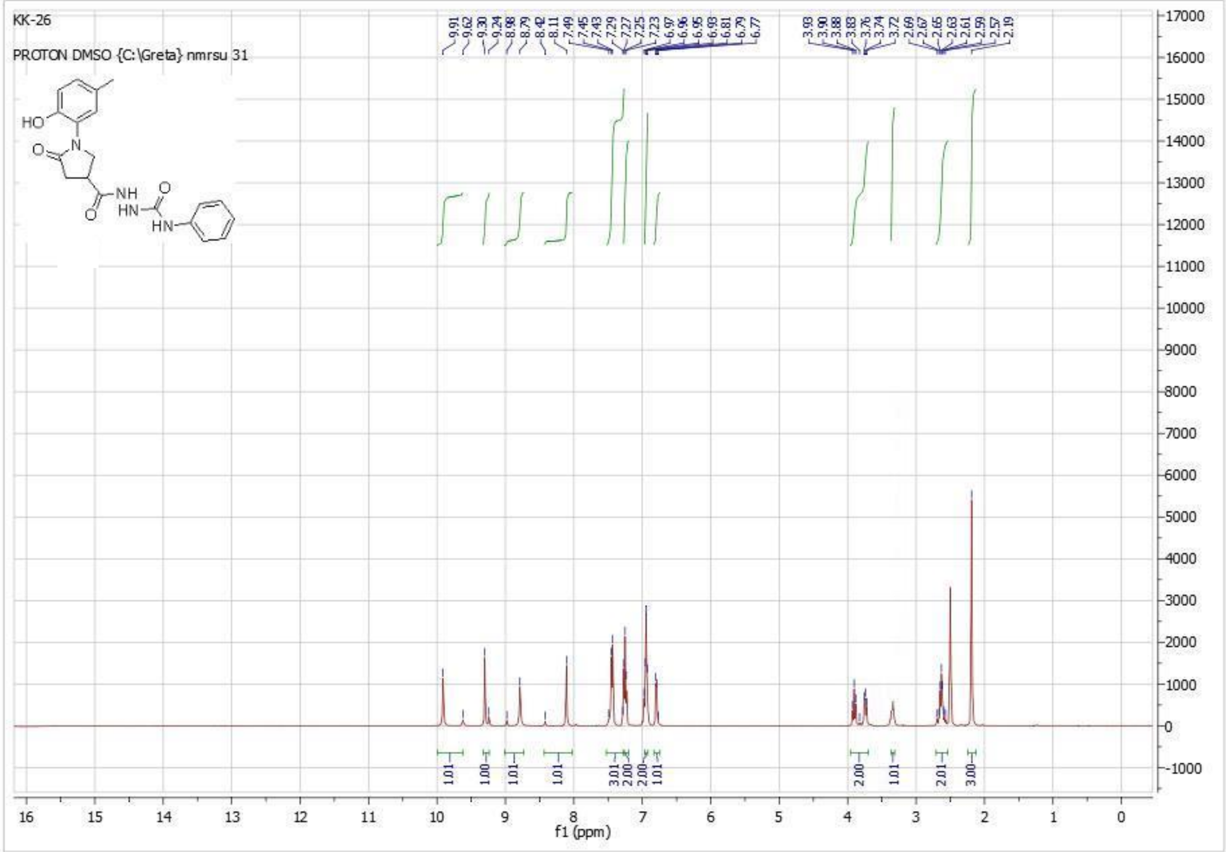

Figure S9. ^{1}H NMR of compound **6.**

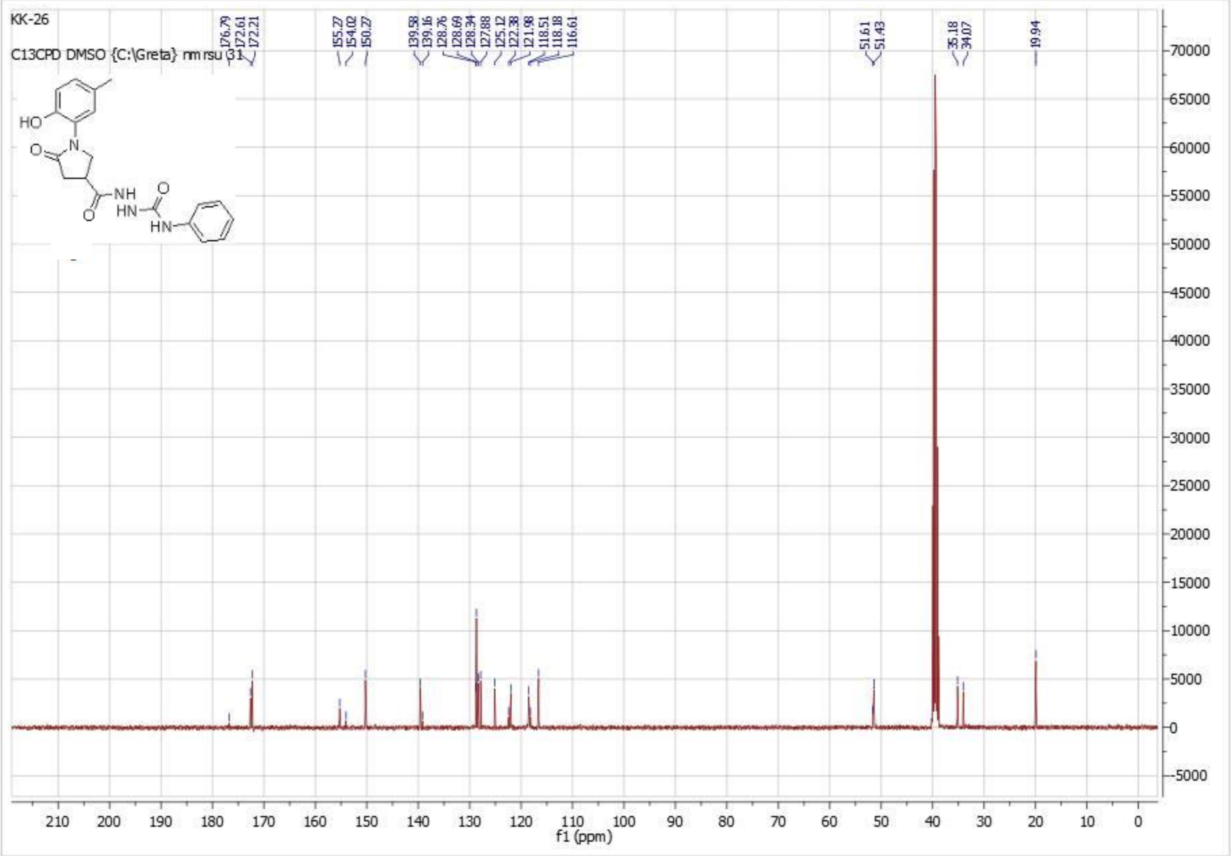

Figure S10. ^{13}C NMR of compound **6.**

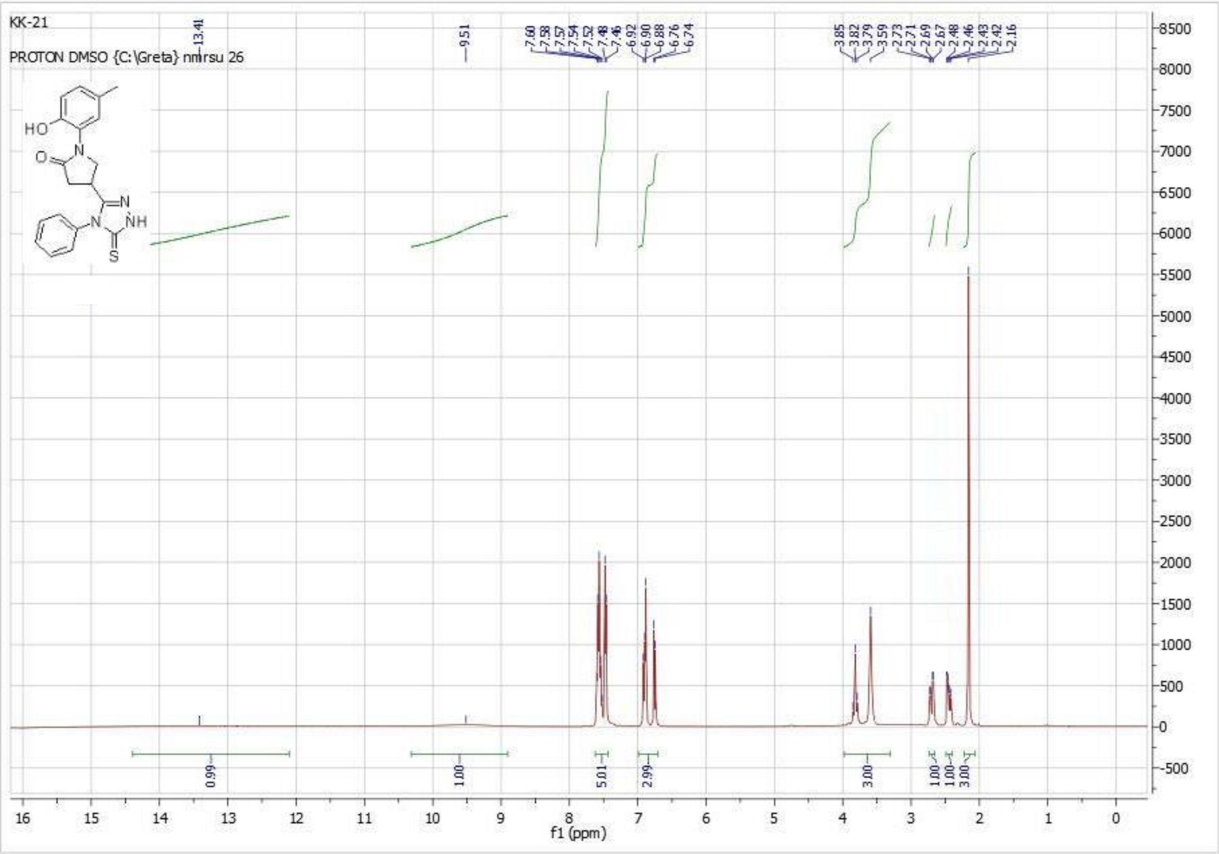

Figure S11. ^{1}H NMR of compound **7.**

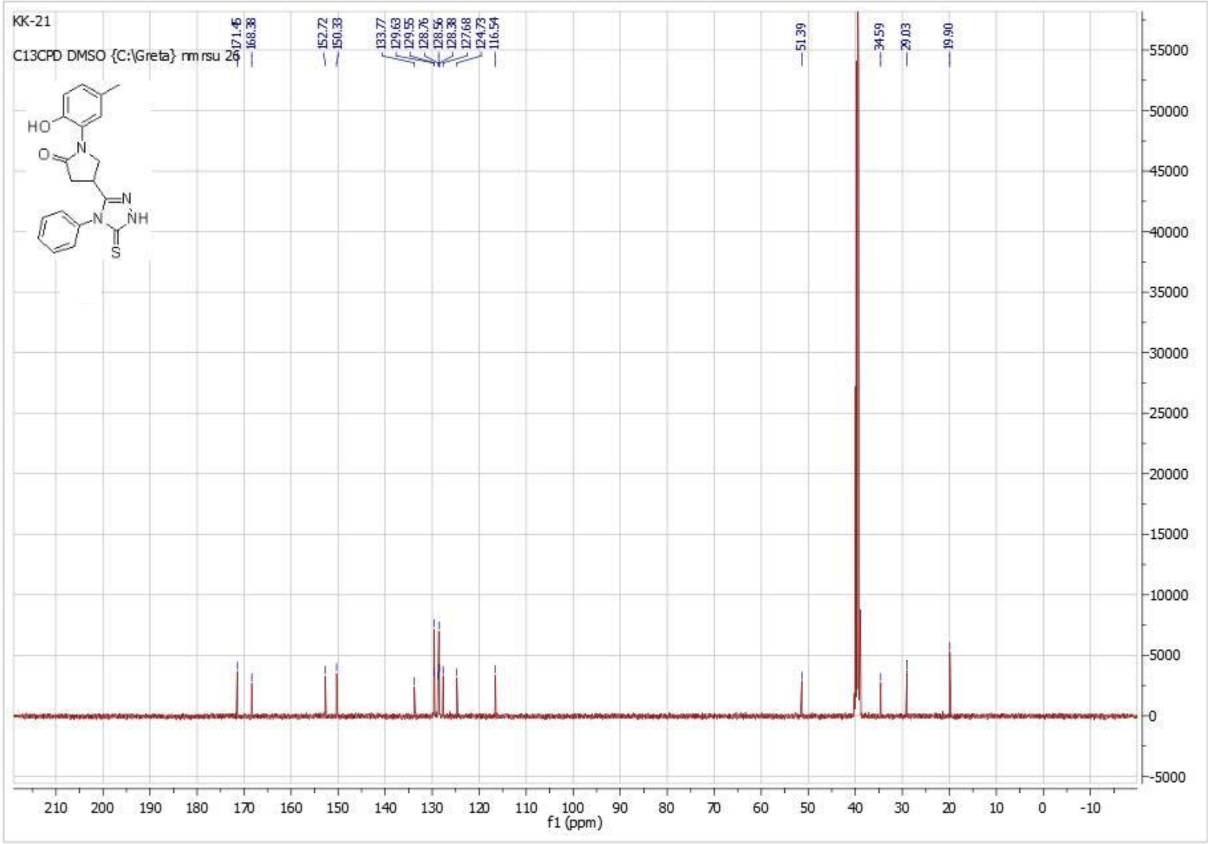

Figure S12. ^{13}C NMR of compound **7.**

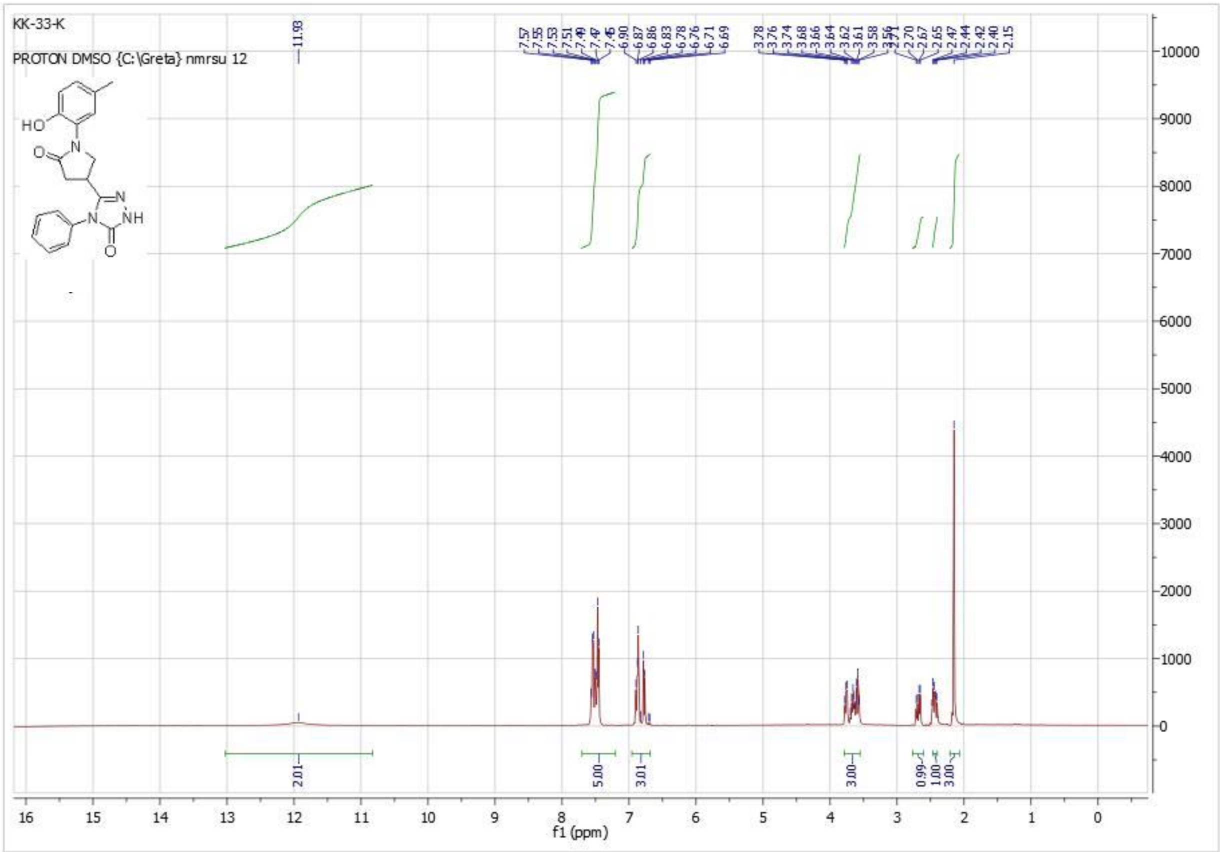

Figure S13. ^{1}H NMR of compound **8.**

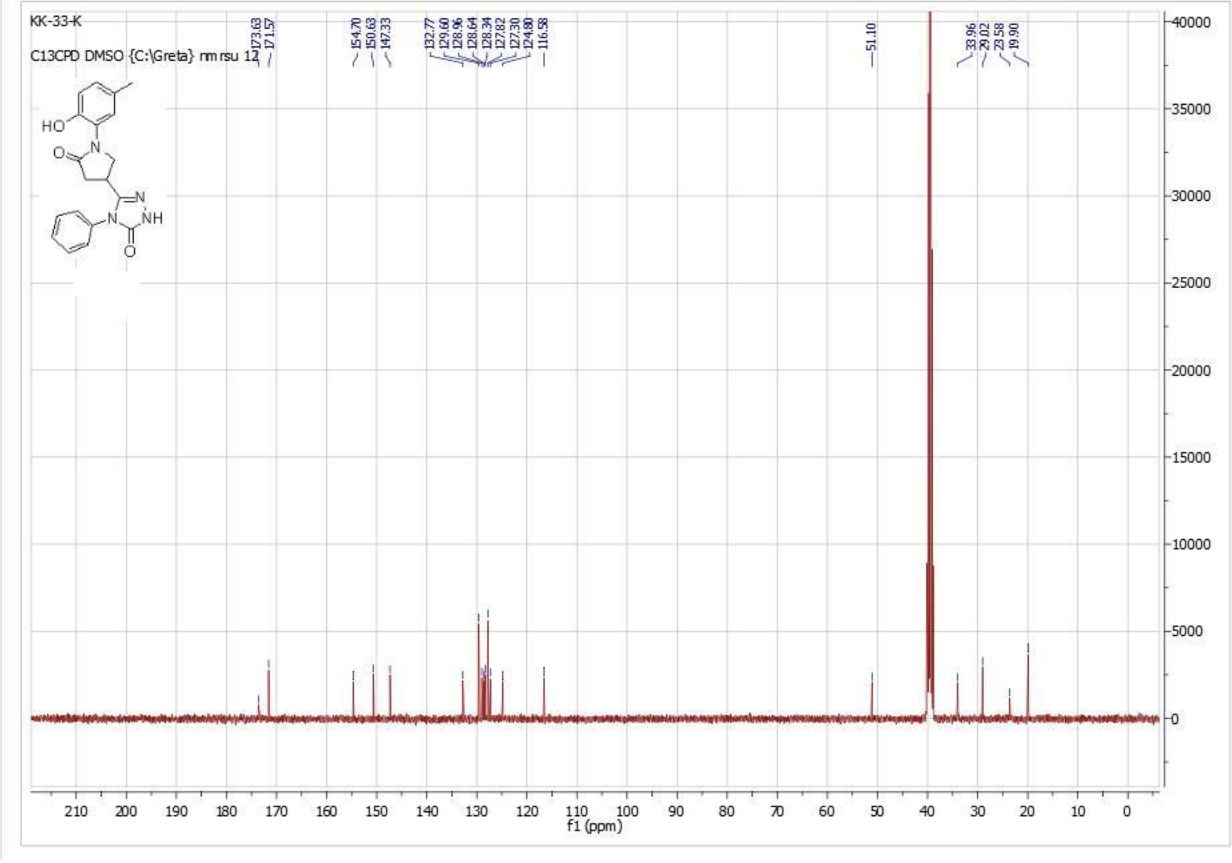

Figure S14. ^{13}C NMR of compound **8.**

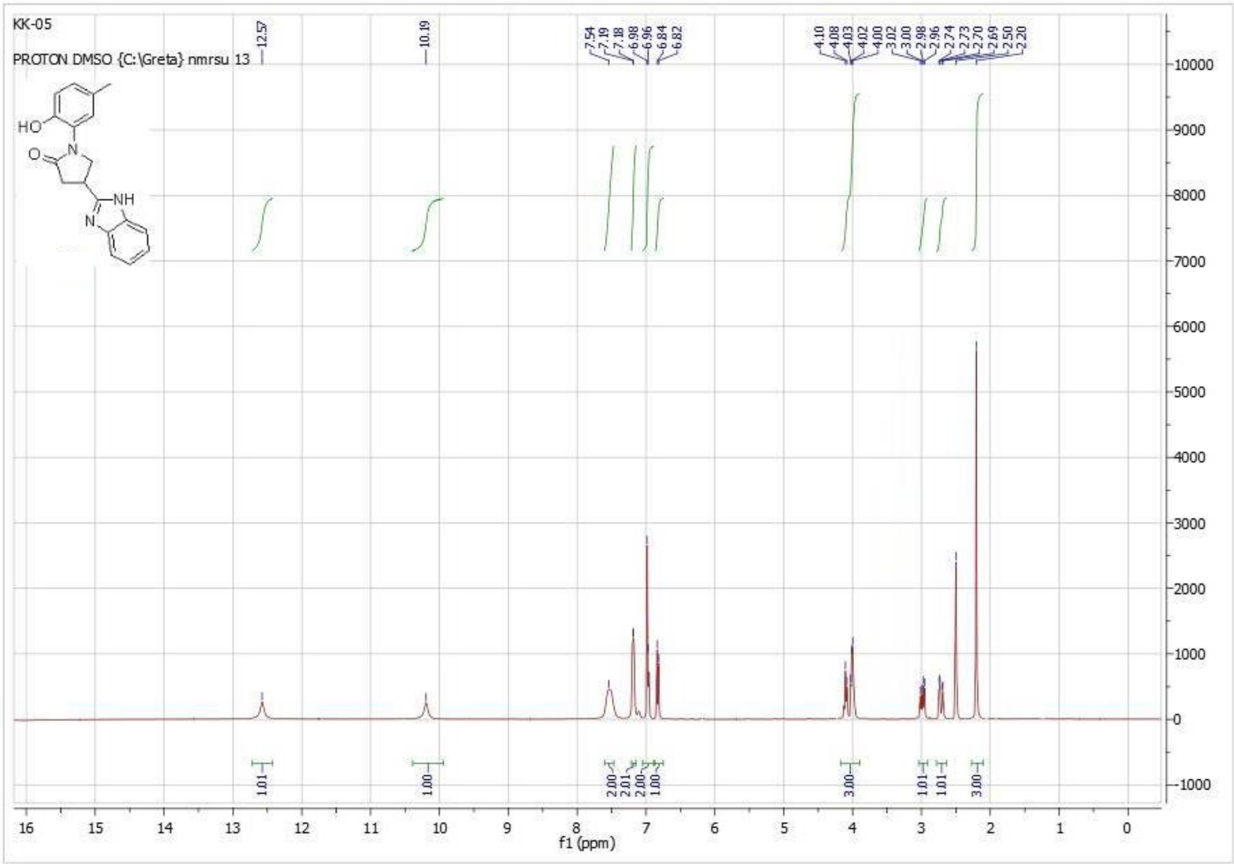

Figure S15. ^{1}H NMR of compound **9a.**

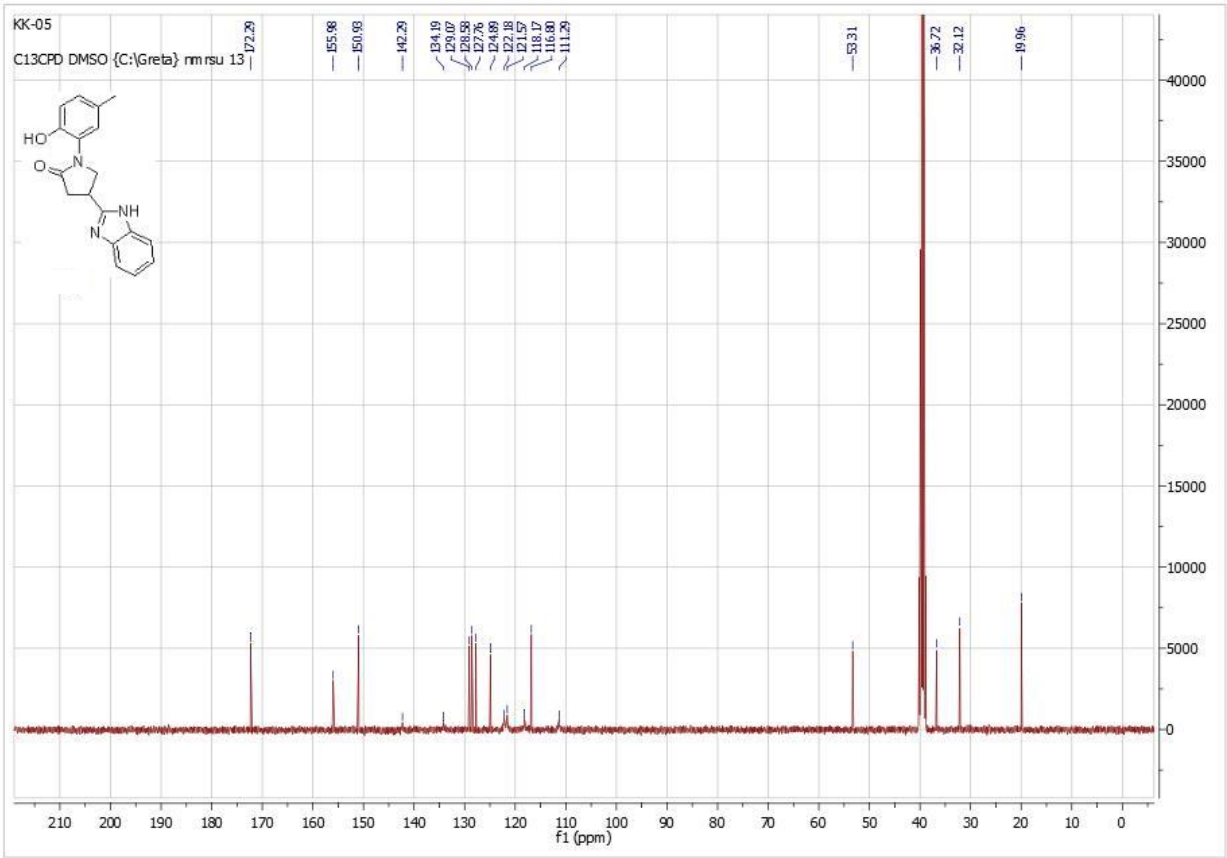

Figure S16. ^{13}C NMR of compound **9a.**

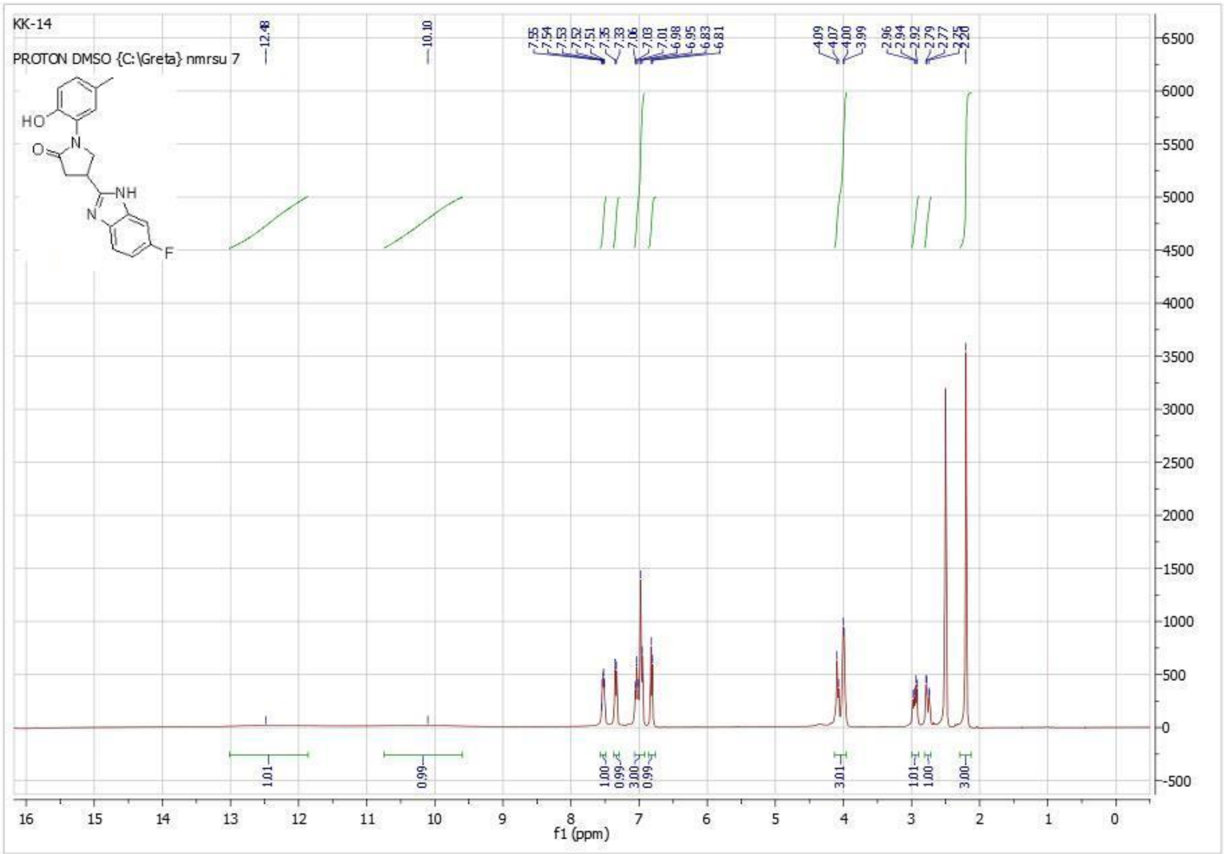

Figure S17. ^{1}H NMR of compound **9b.**

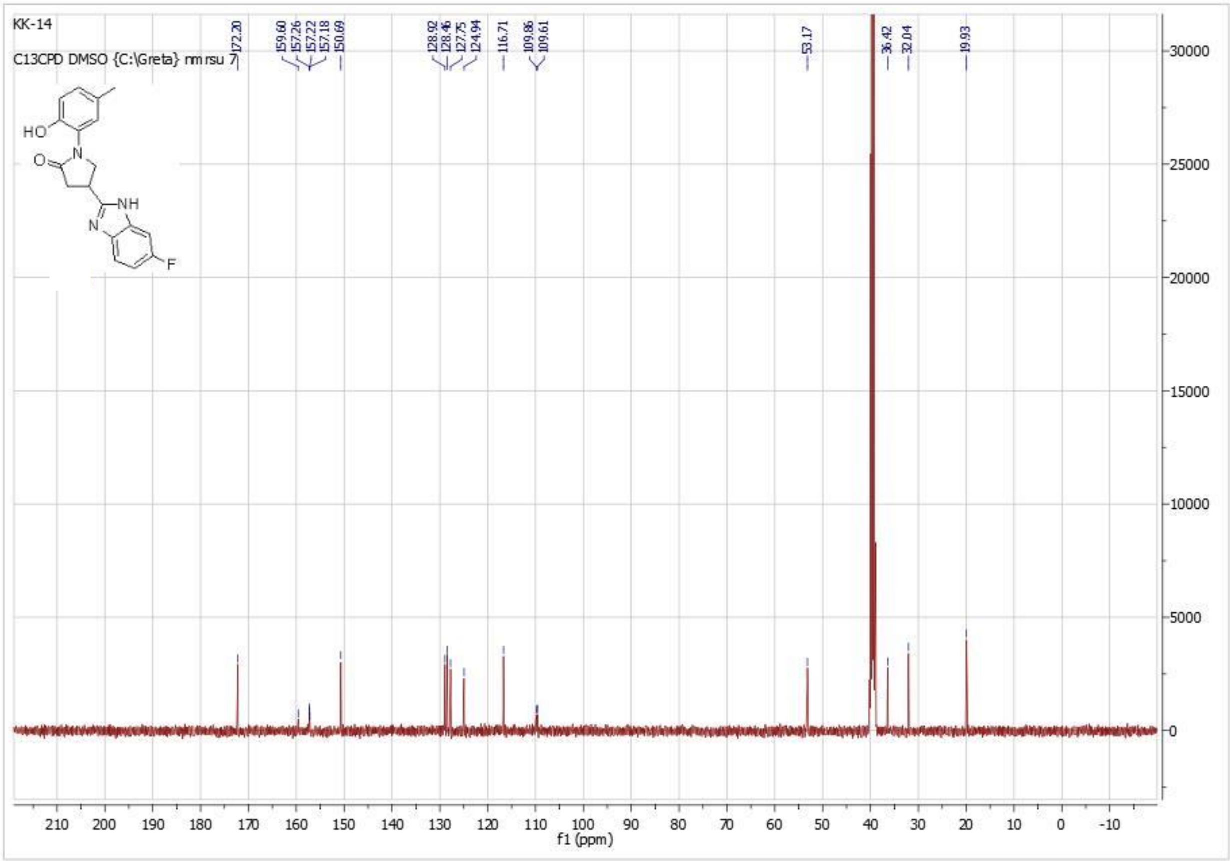

Figure S18. ^{13}C NMR of compound **9b.**

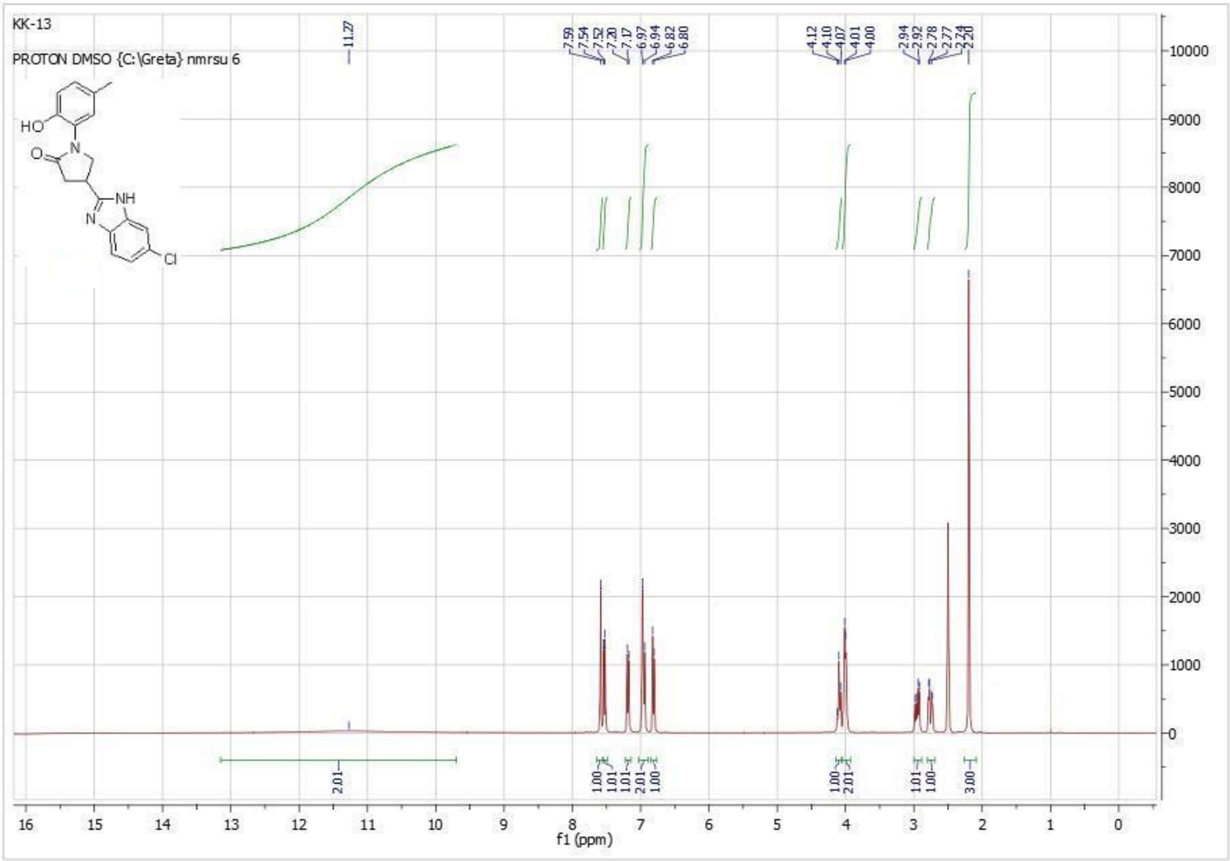

Figure S19. ¹H NMR of compound **9c.**

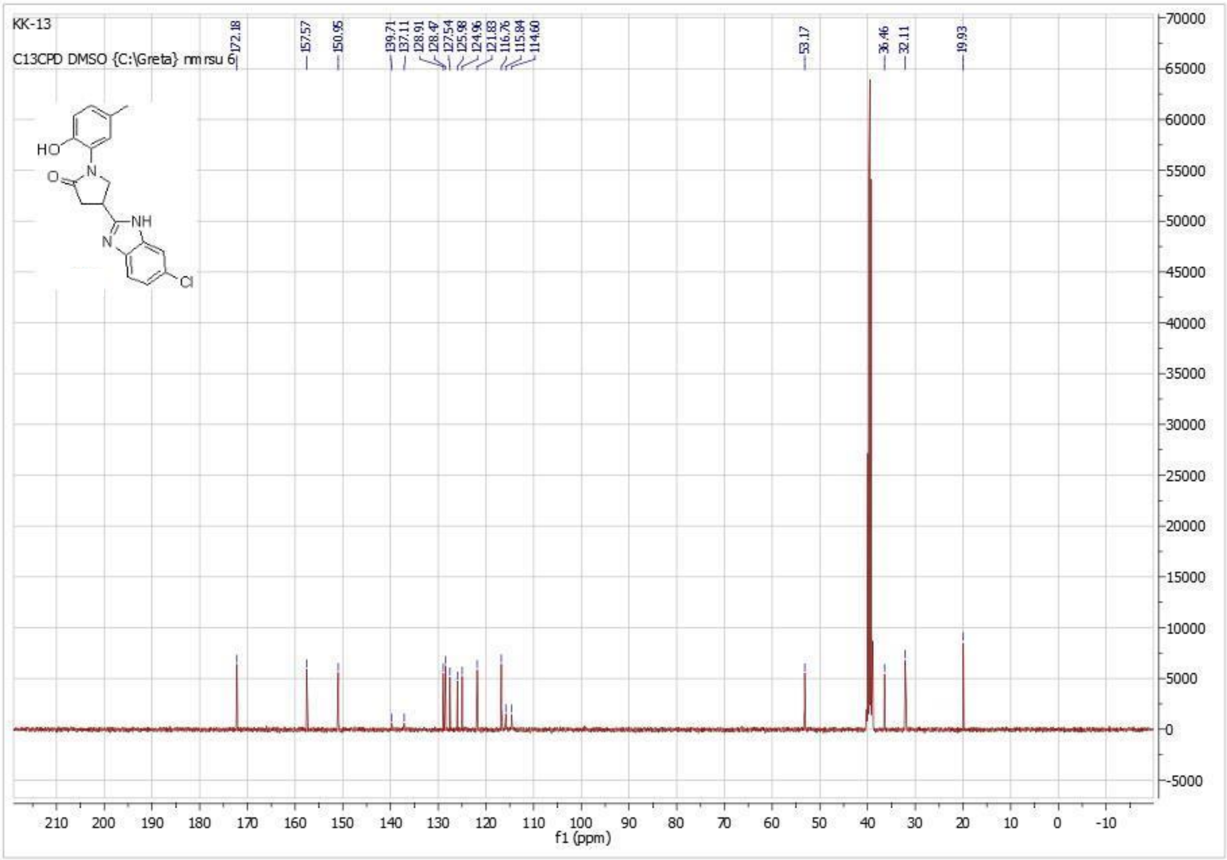

Figure S20. ¹³C NMR of compound **9c.**

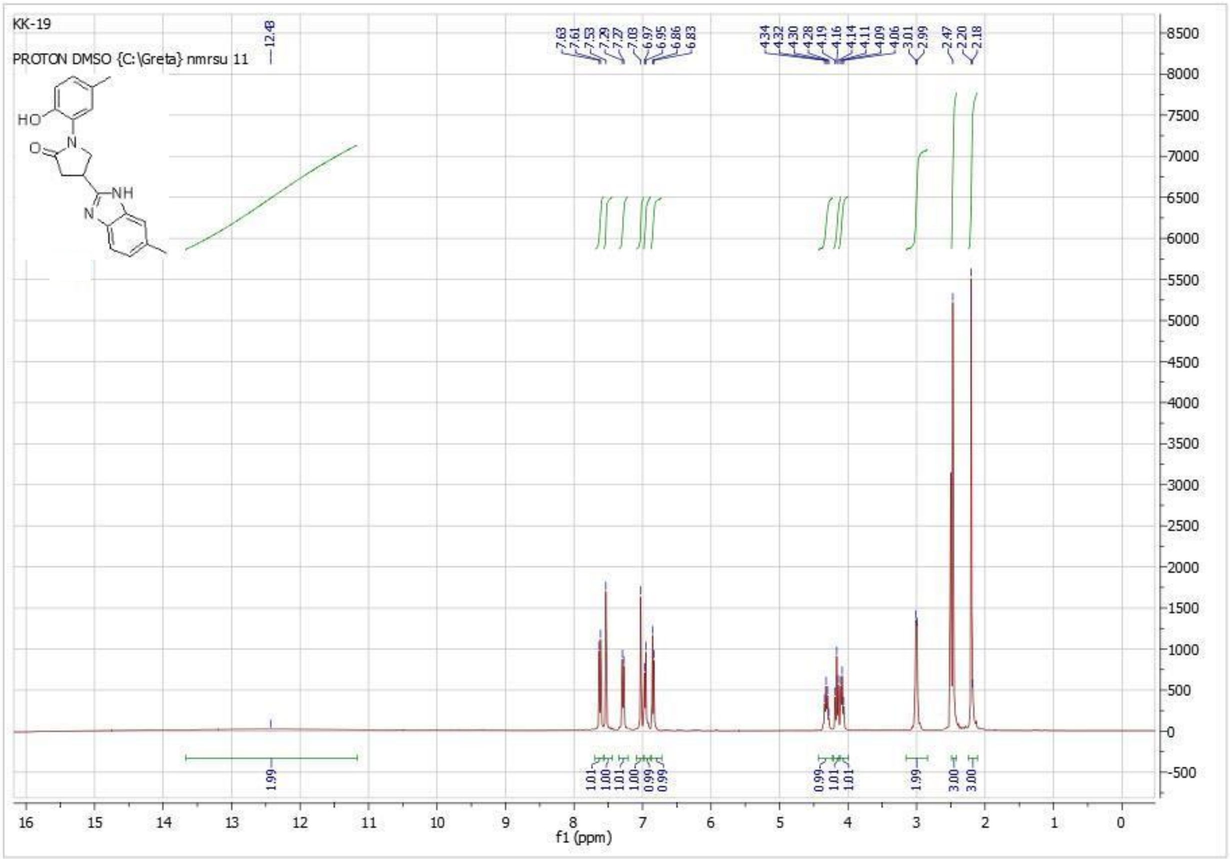

Figure S21. ^{1}H NMR of compound **9d.**

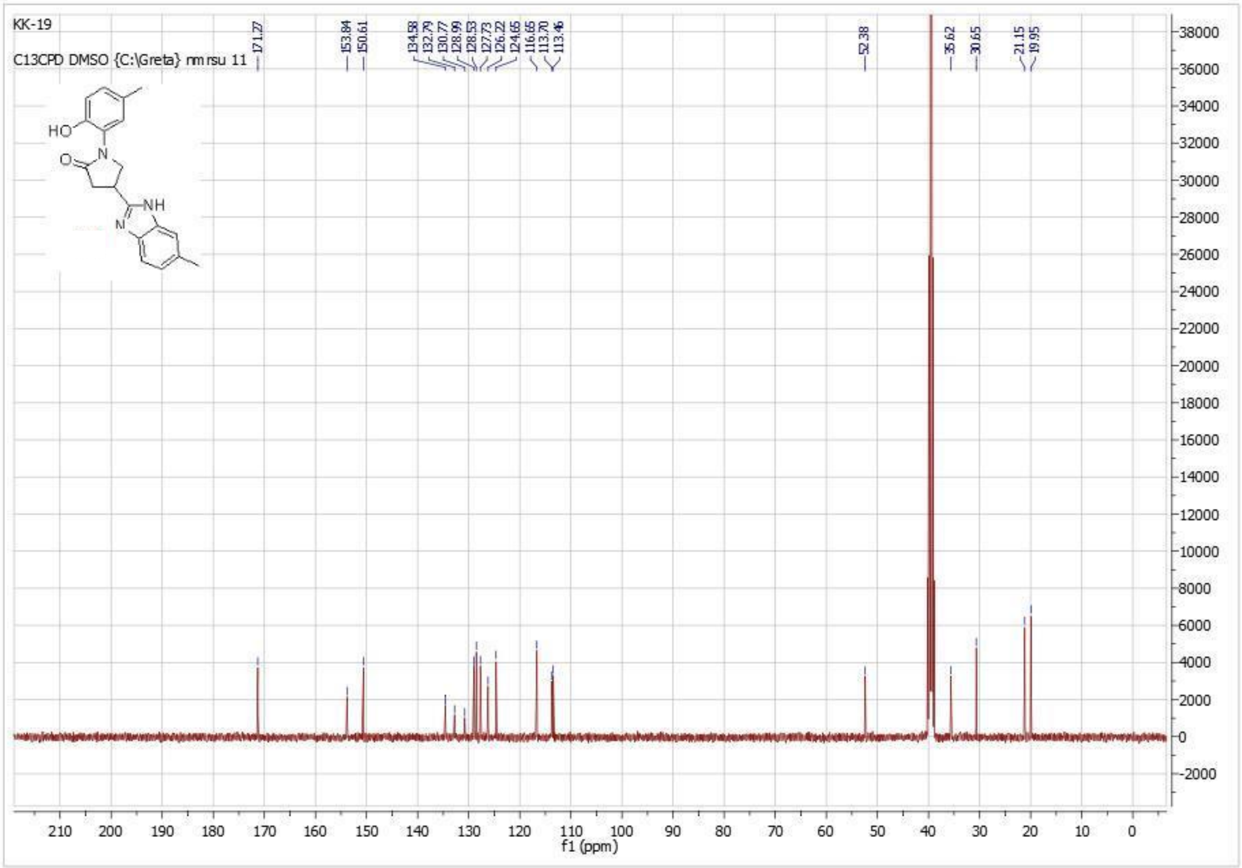

Figure S22. ^{13}C NMR of compound **9d.**

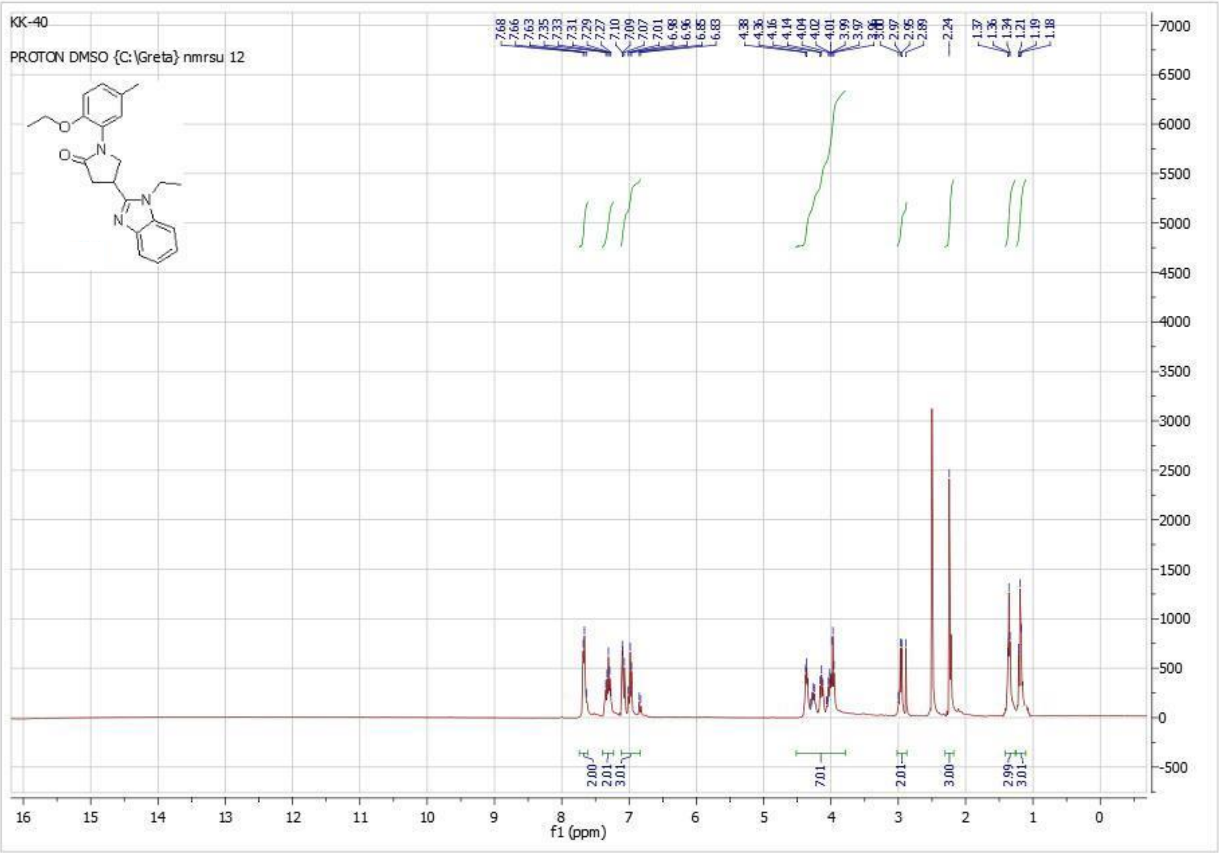

Figure S23. ^{1}H NMR of compound **10.**

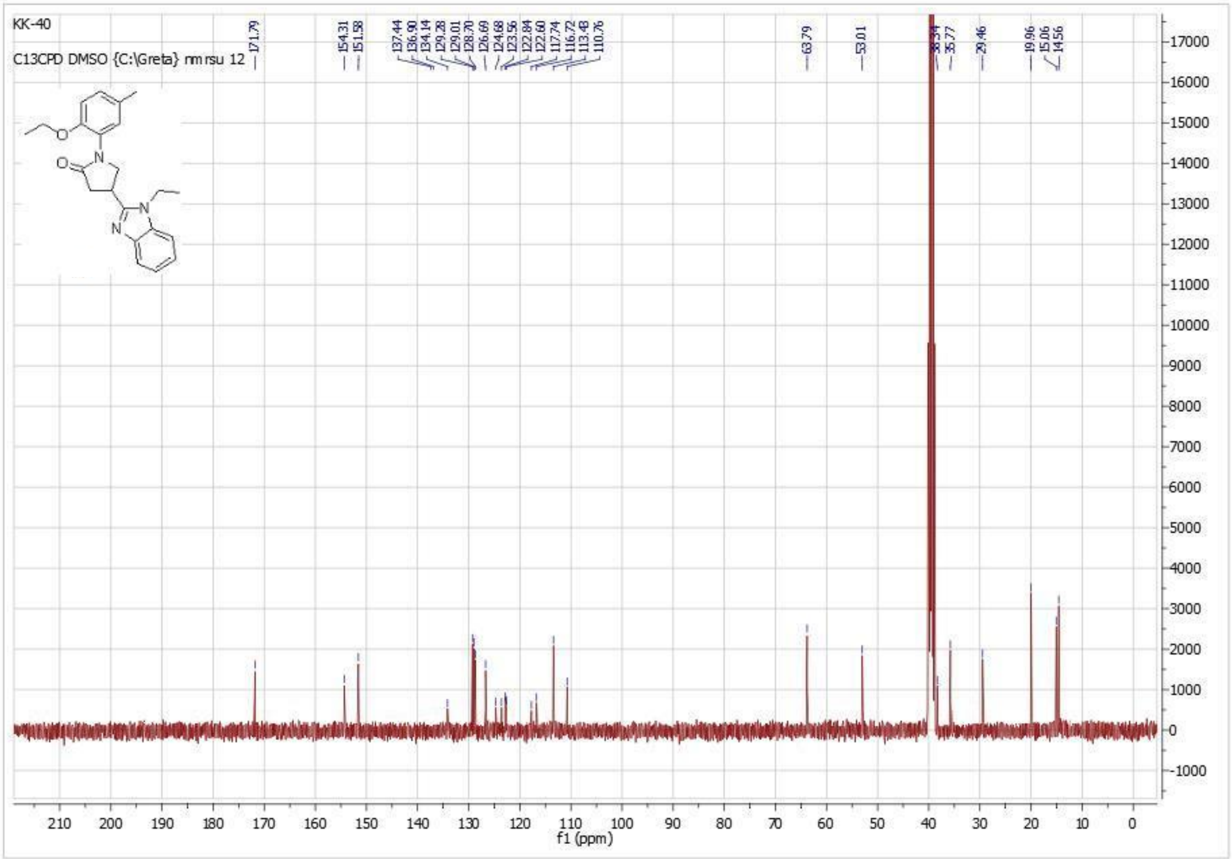

Figure S24. ^{13}C NMR of compound **10.**

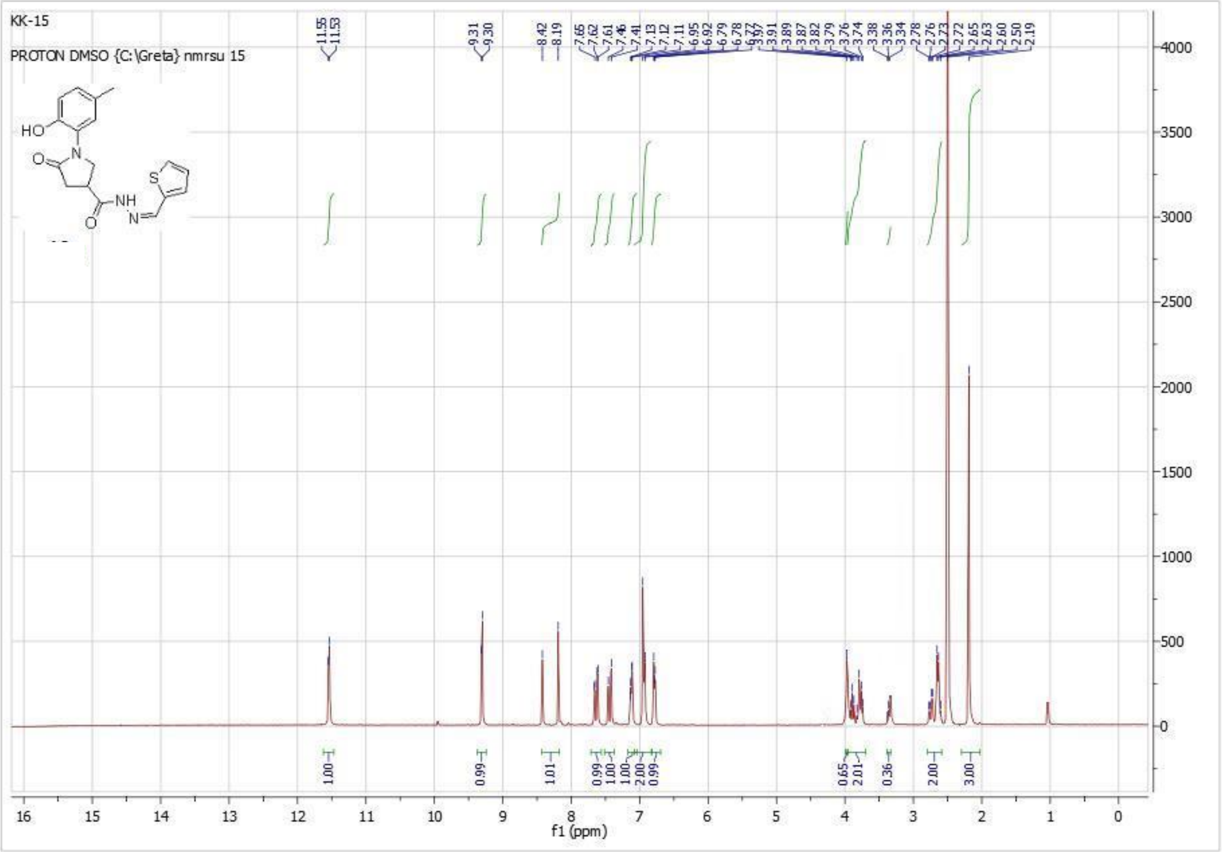

Figure S25. ^{1}H NMR of compound **11a.**

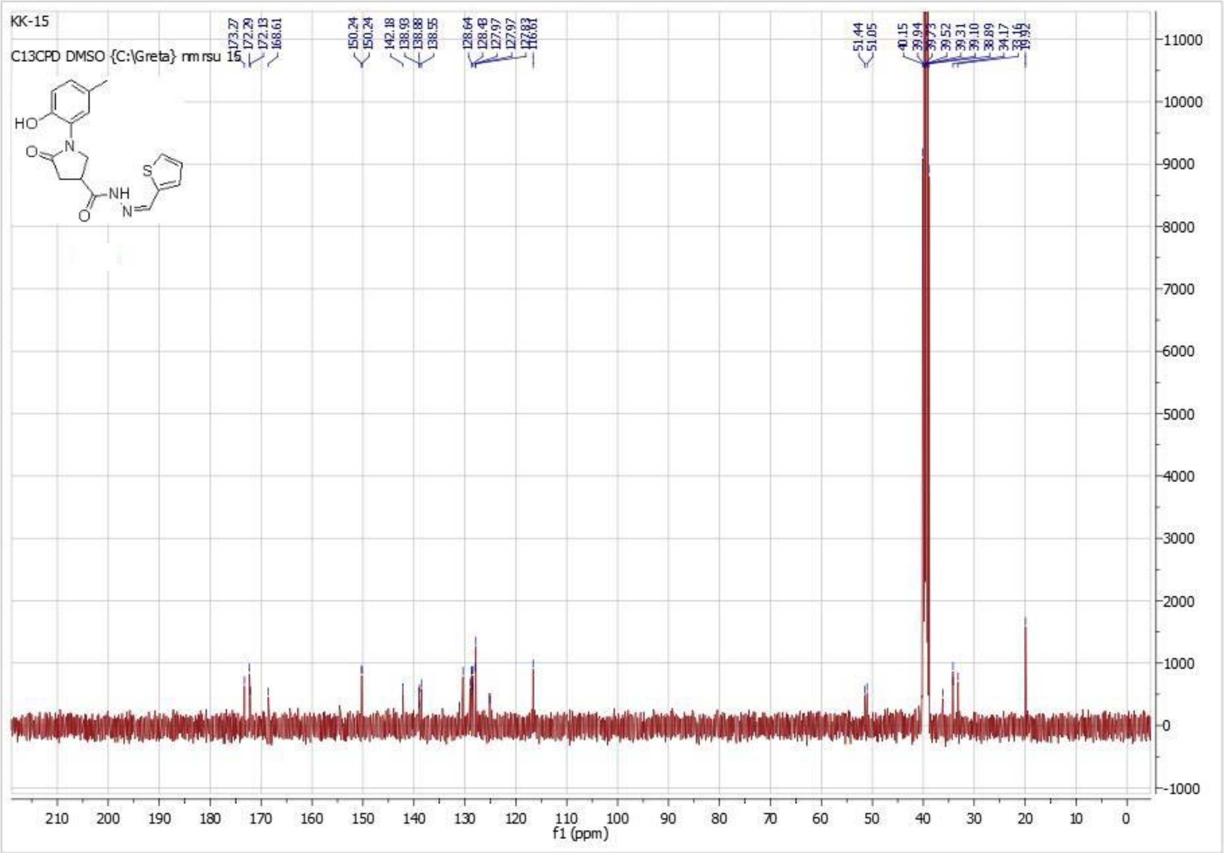

Figure S26. ^{13}C NMR of compound **11a.**

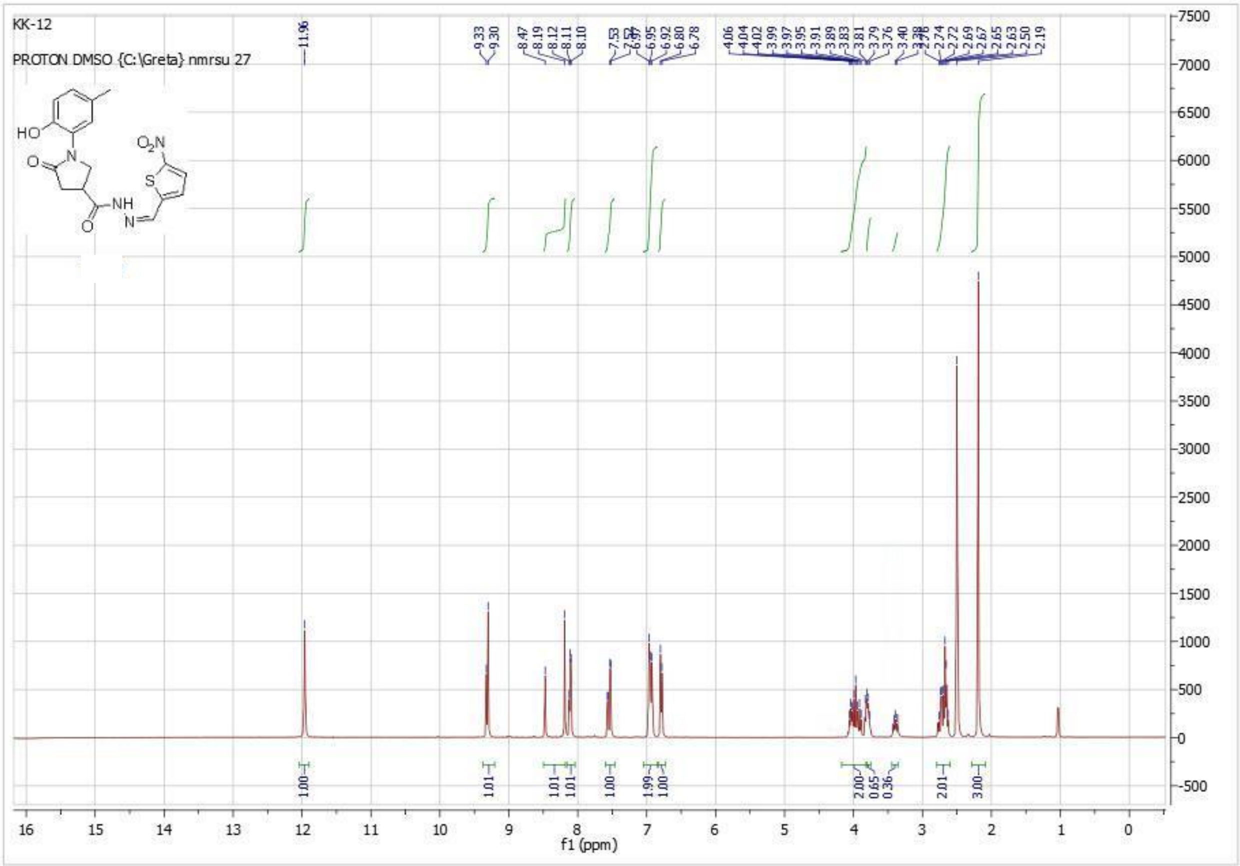

Figure S27. ^{1}H NMR of compound **11b.**

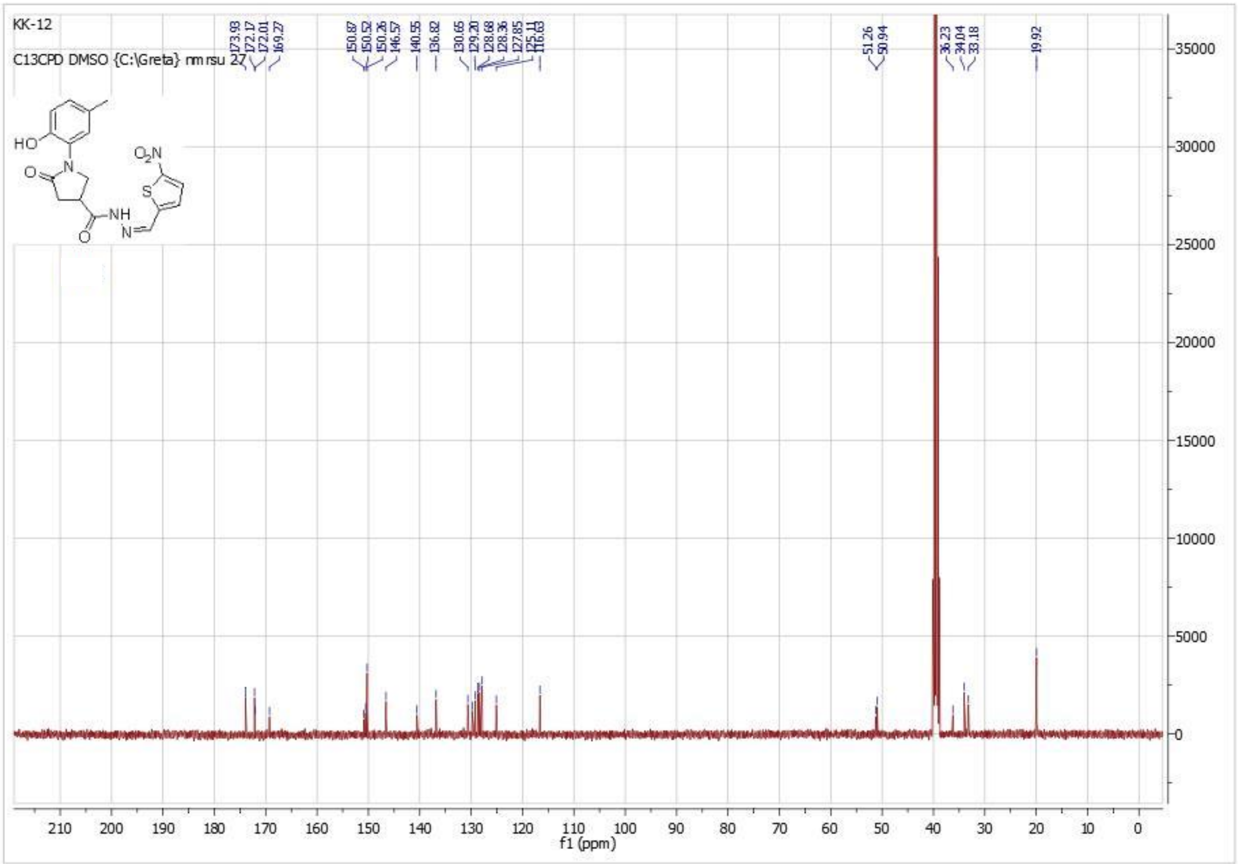

Figure S28. ^{13}C NMR of compound **11b.**

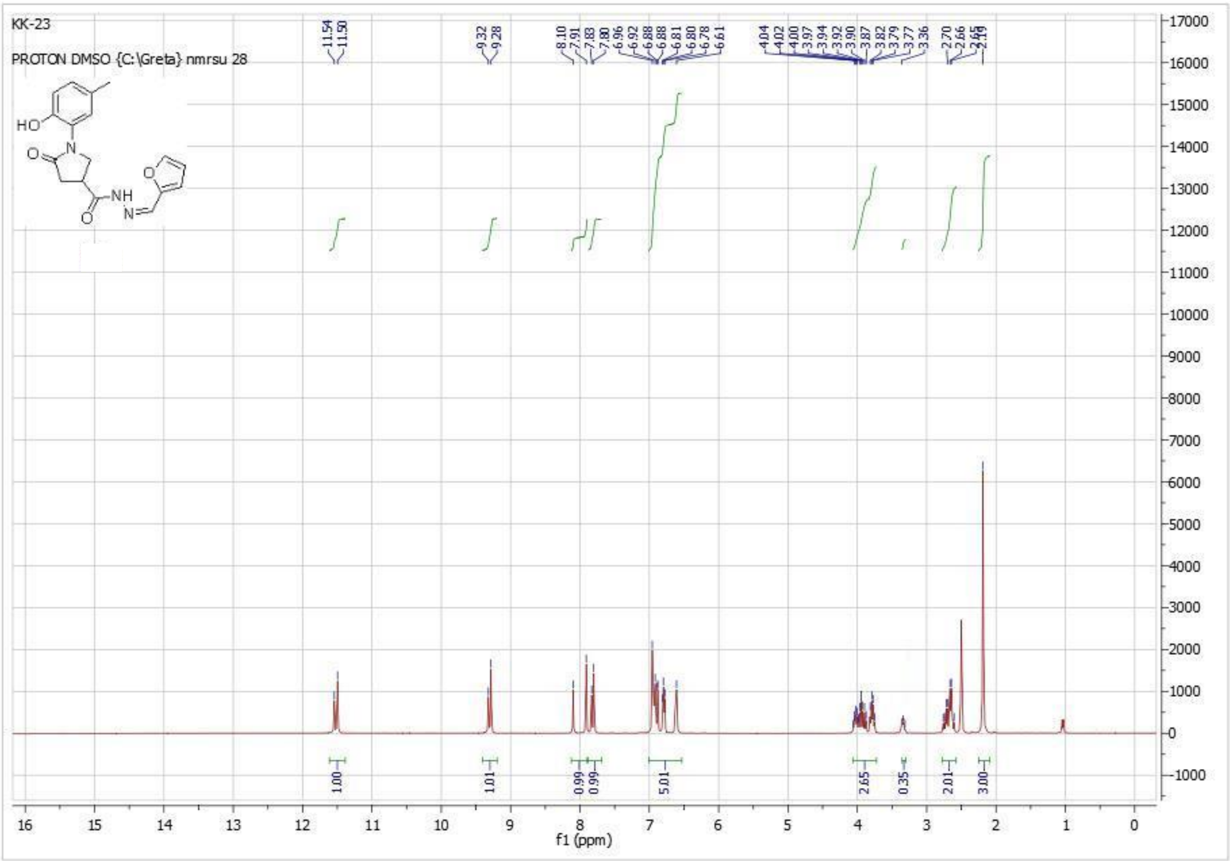

Figure S29. ^{1}H NMR of compound **11c.**

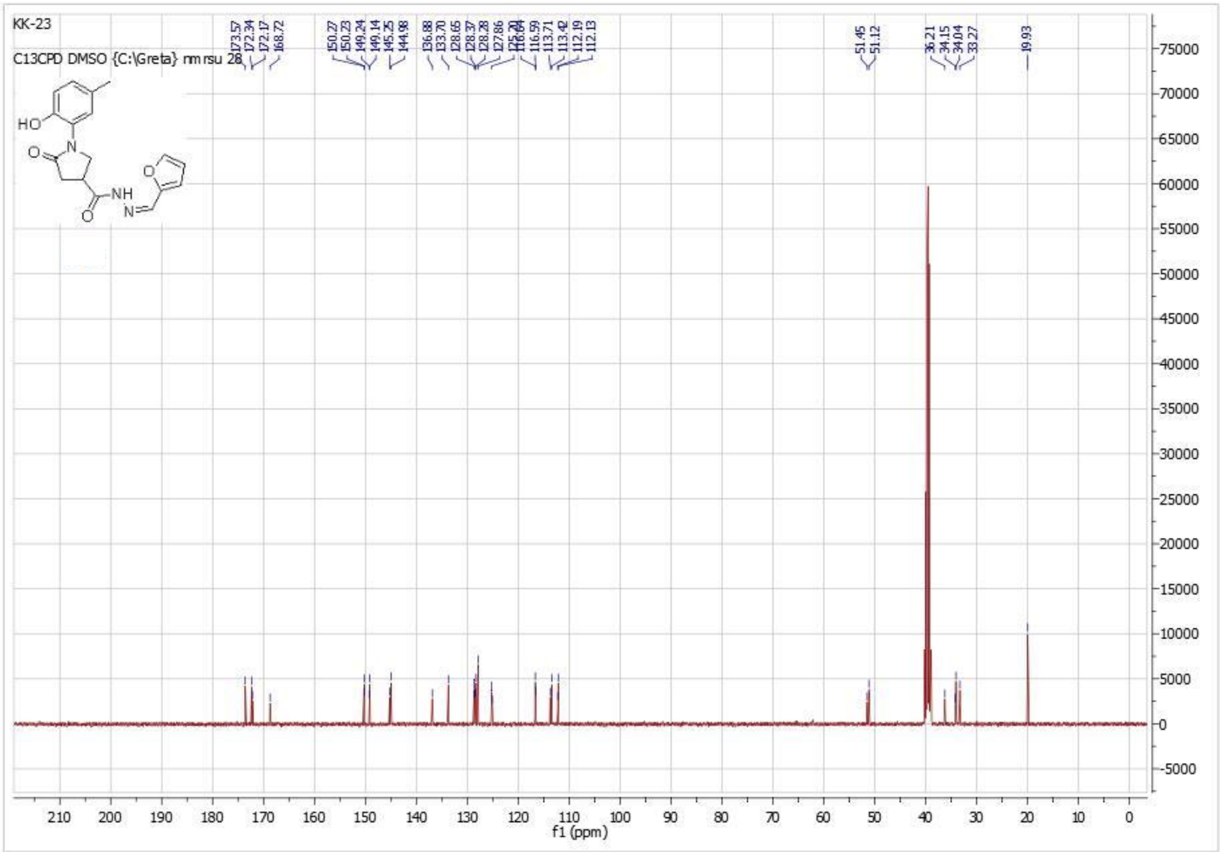

Figure S30. ^{13}C NMR of compound **11c.**

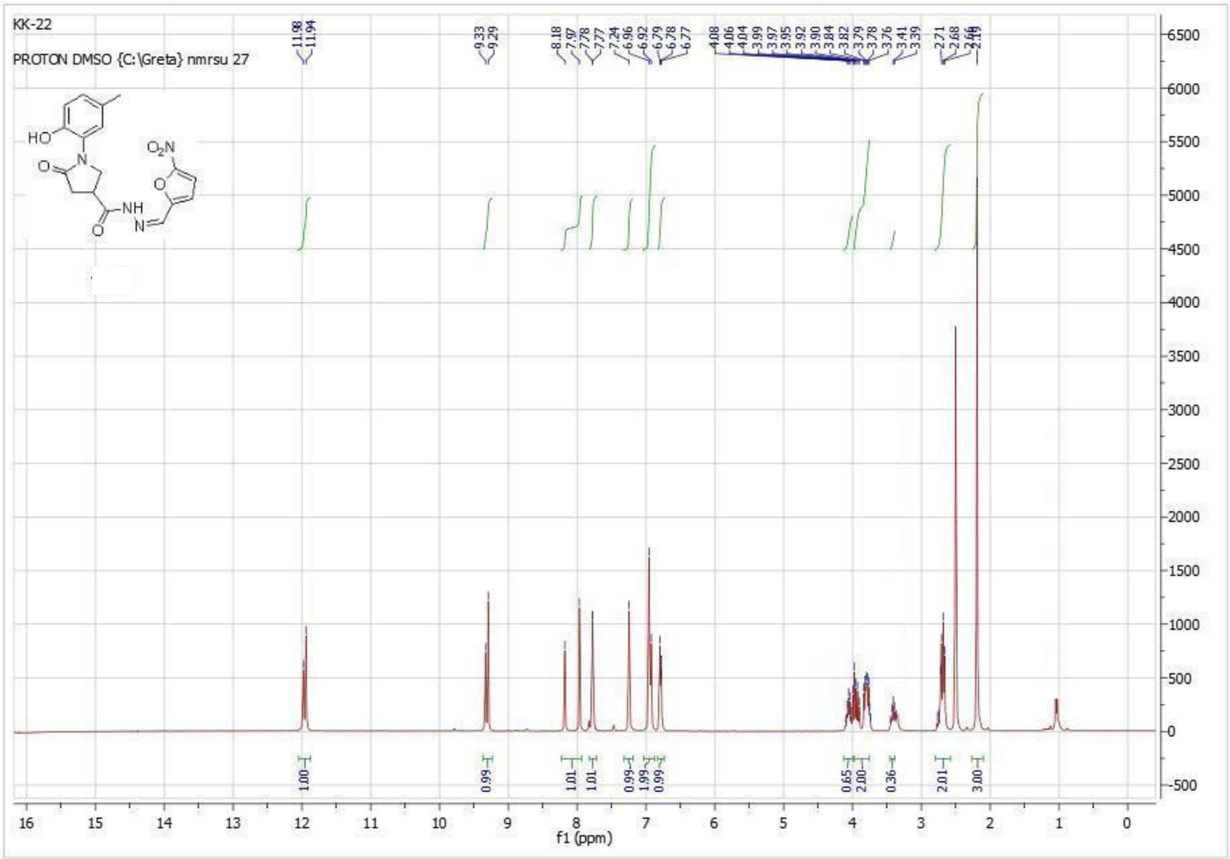

Figure S31. ^{1}H NMR of compound **11d.**

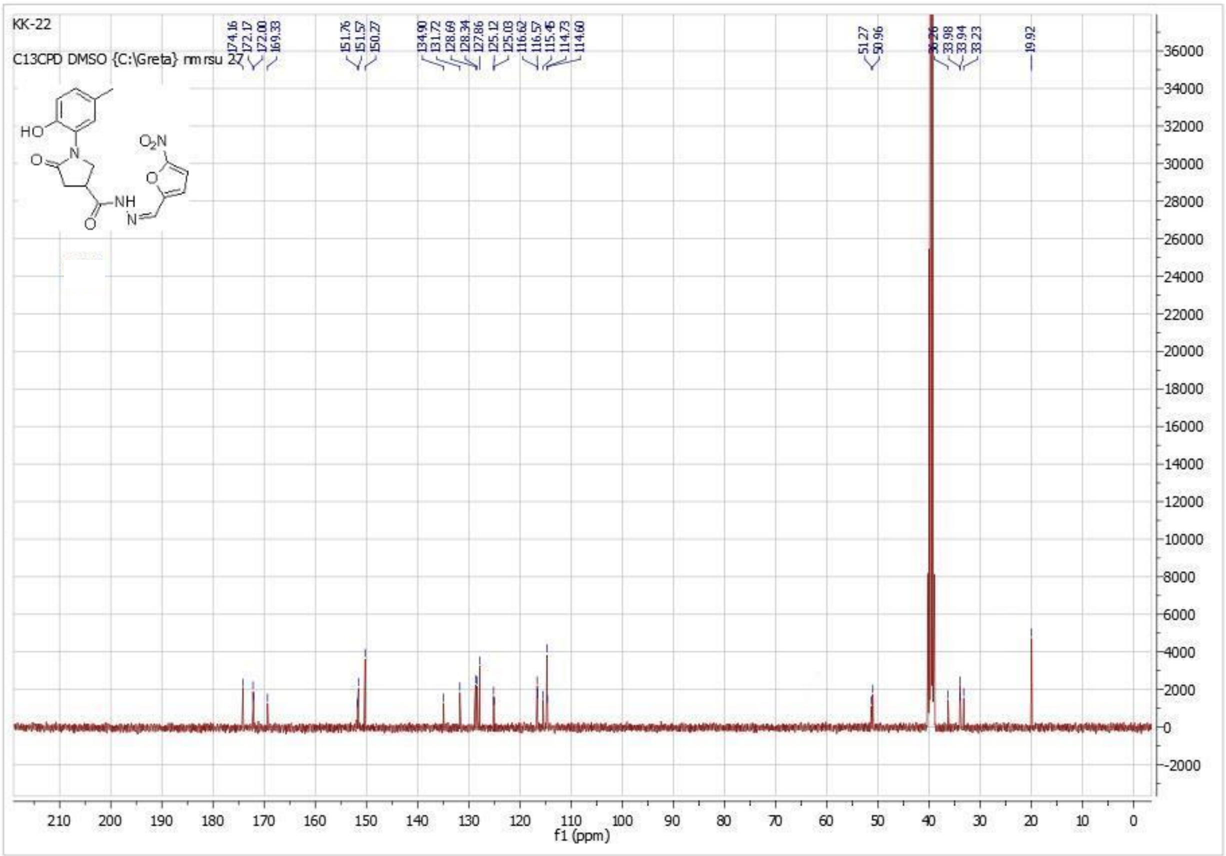

Figure S32. ^{13}C NMR of compound **11d.**

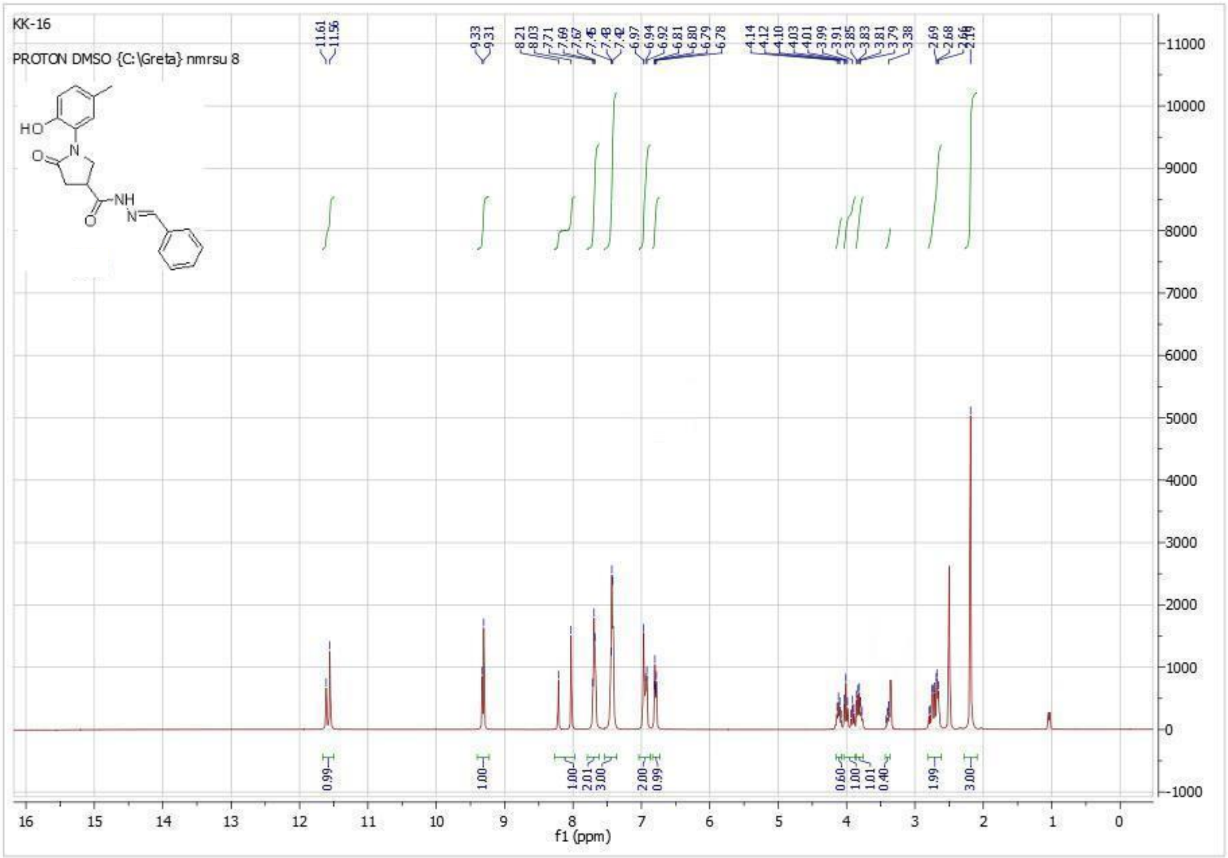

Figure S33. ^{1}H NMR of compound **12a.**

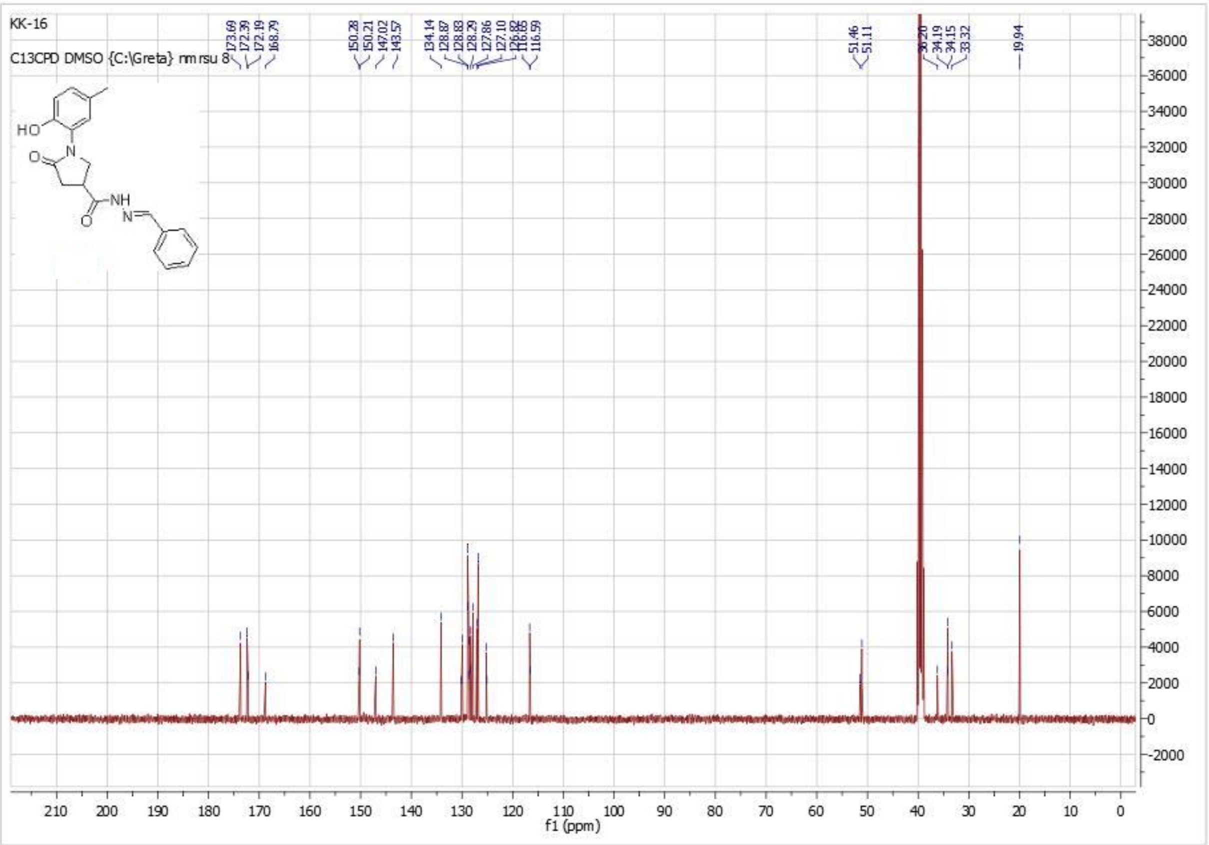

Figure S34. ^{13}C NMR of compound **12a**

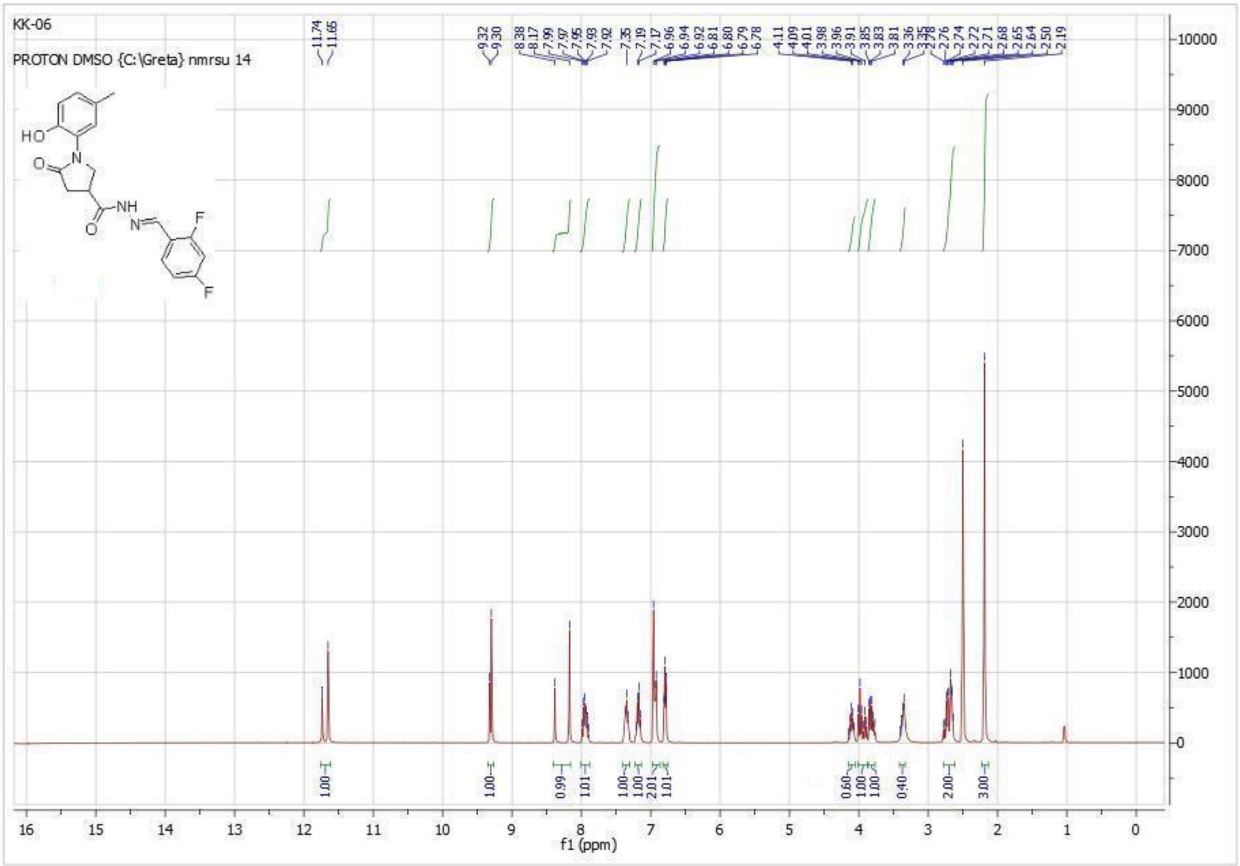

Figure S35. ^{1}H NMR of compound **12b.**

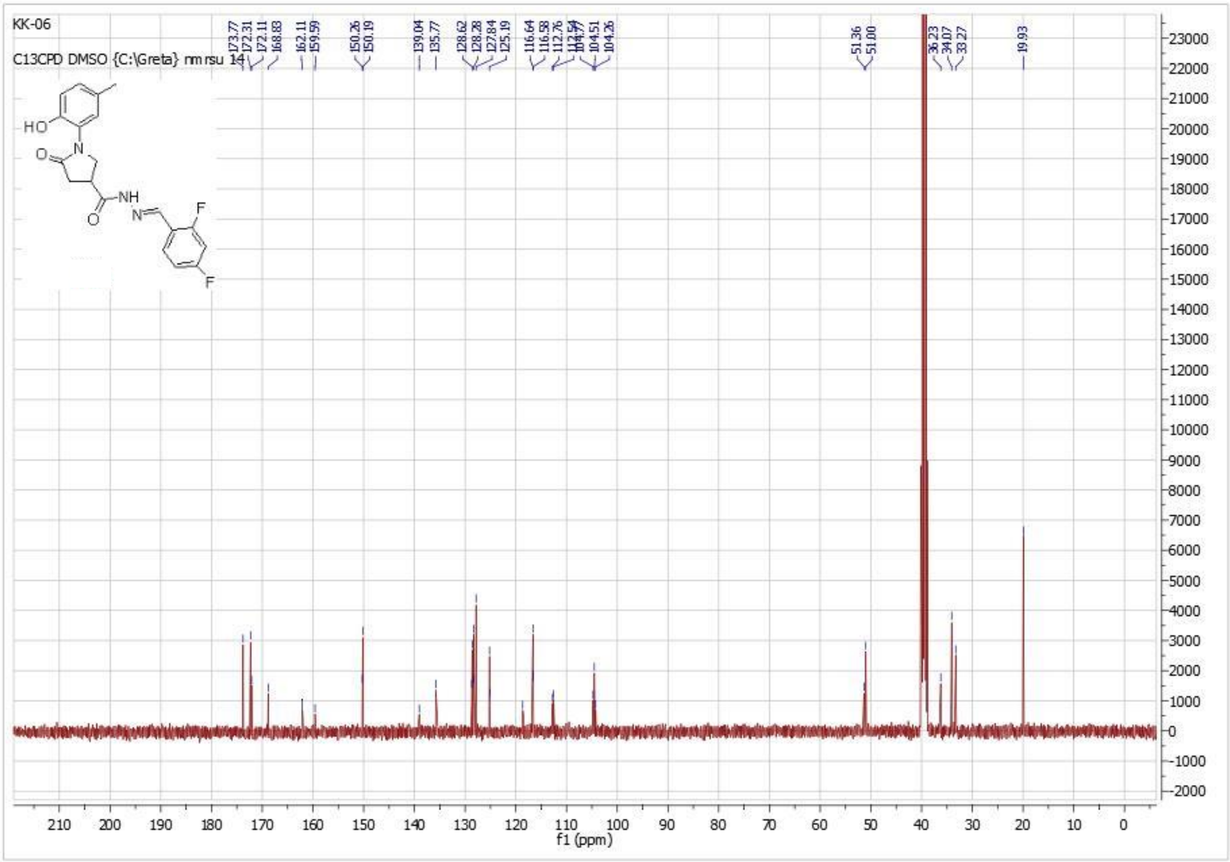

Figure S36. ^{13}C NMR of compound **12b.**

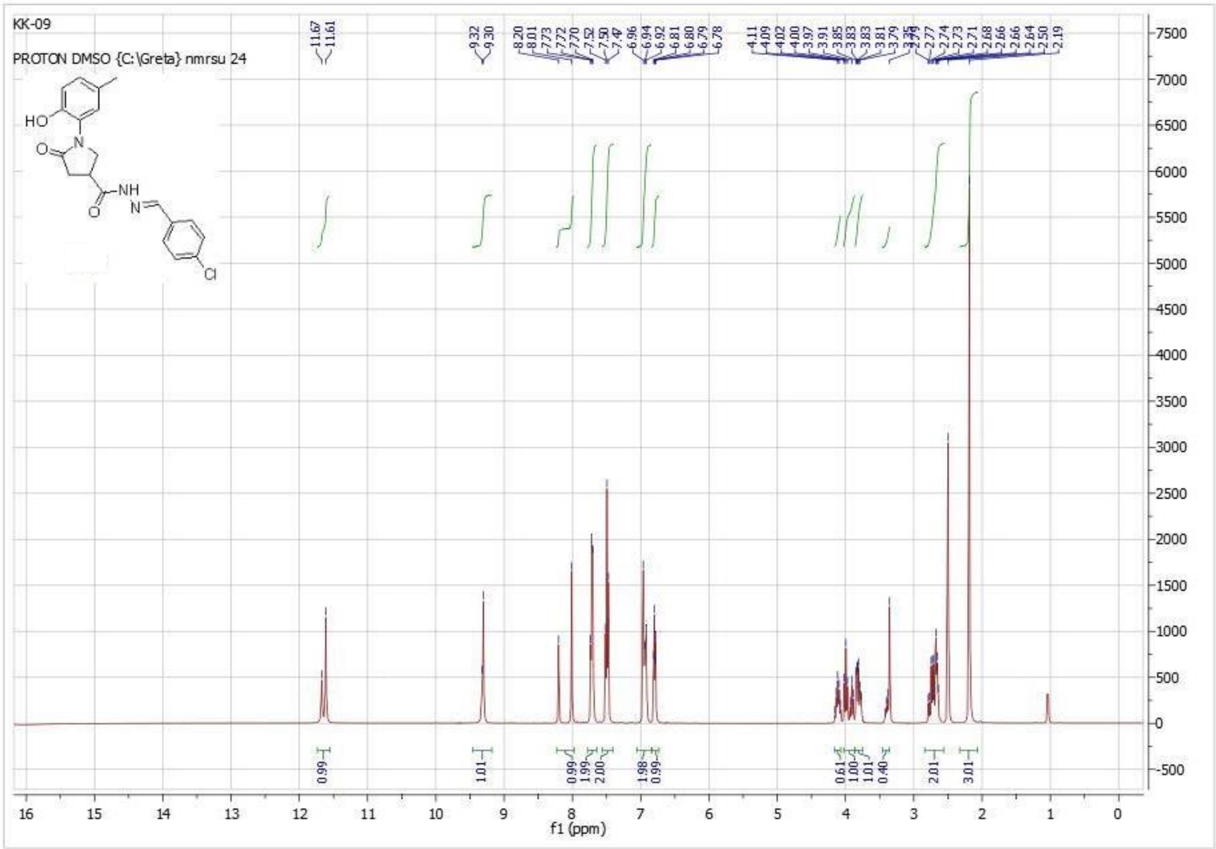

Figure S37. ^{1}H NMR of compound **12c.**

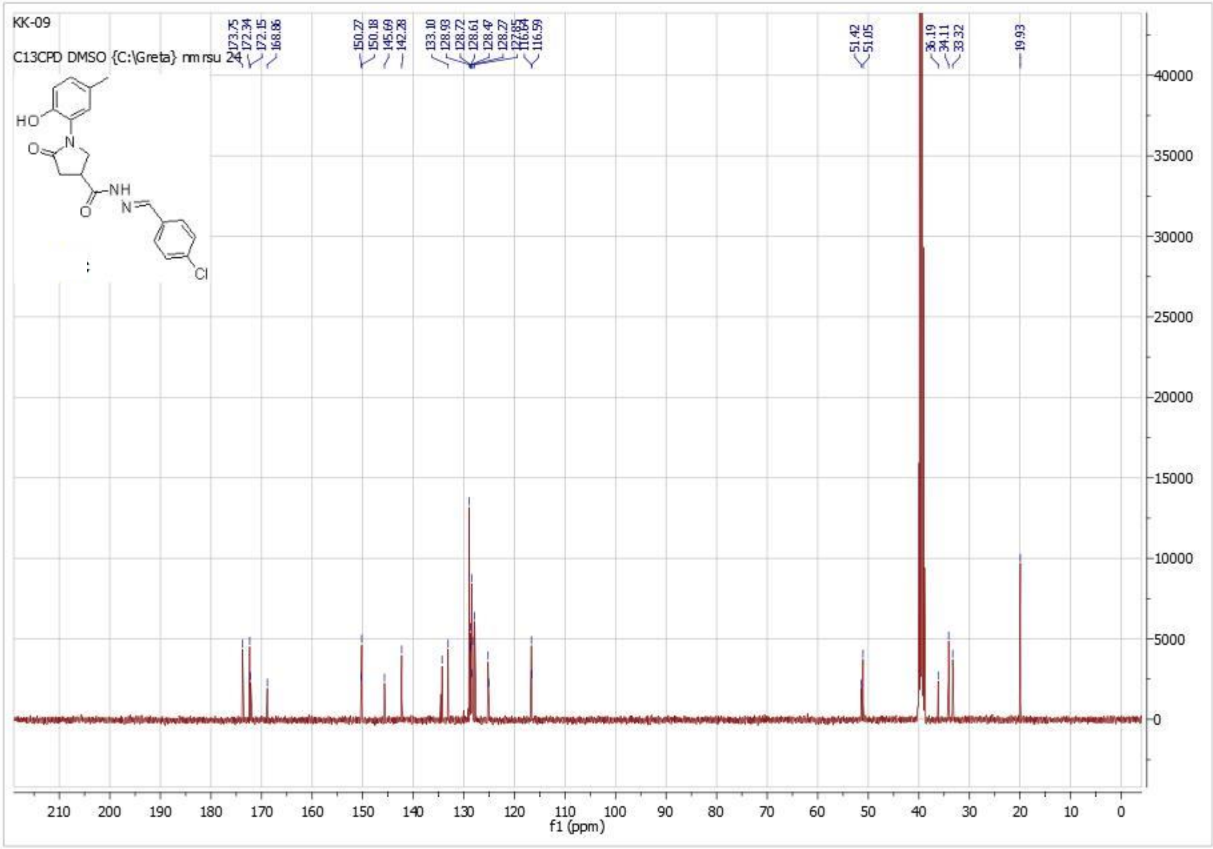

Figure S38. ^{13}C NMR of compound **12c.**

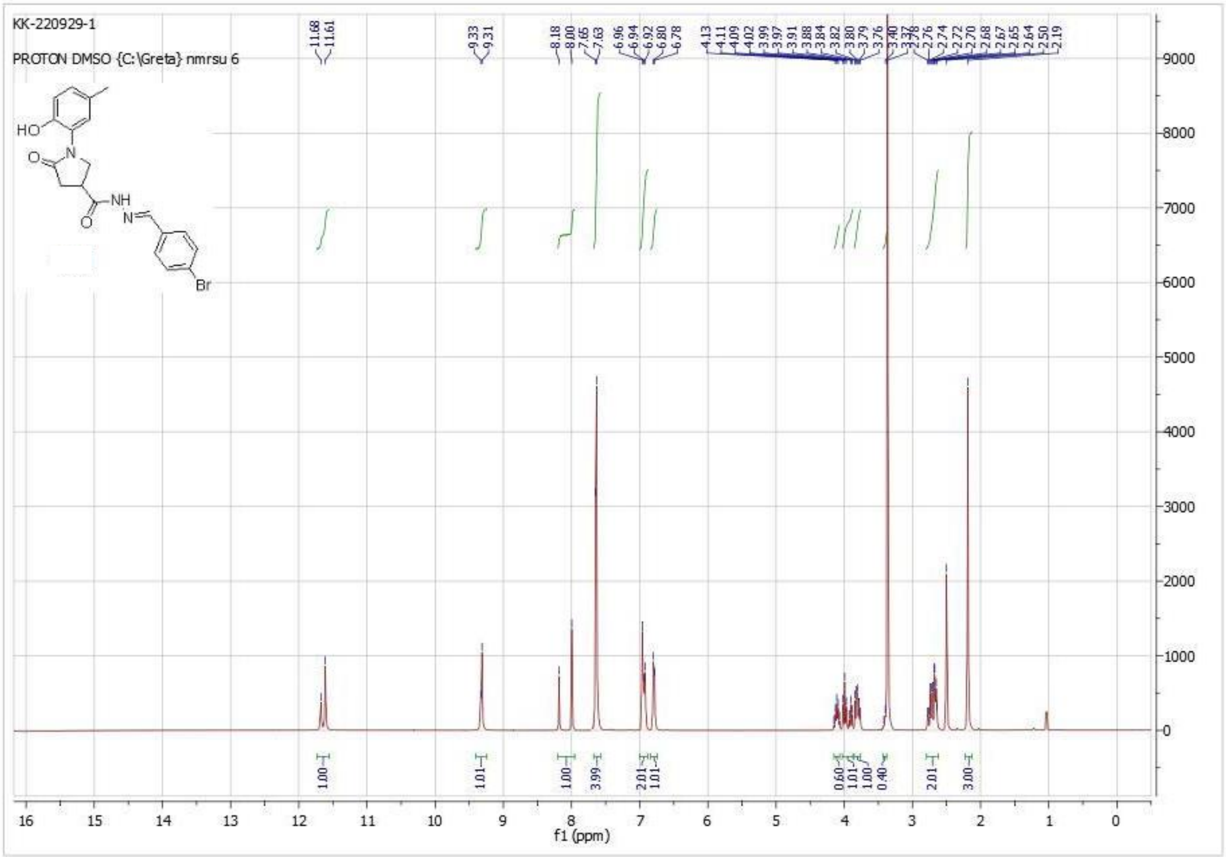

Figure S39. ^{1}H NMR of compound **12d.**

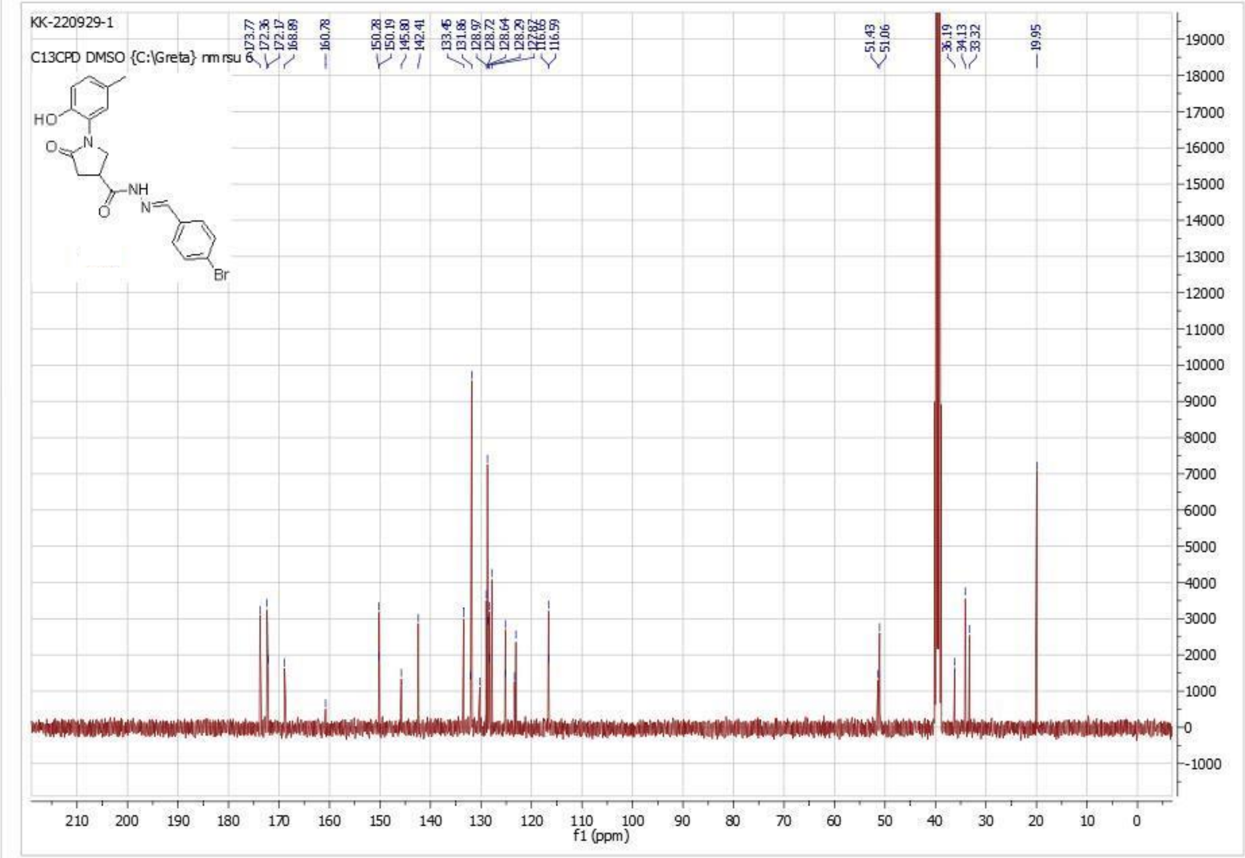

Figure S40. ^{13}C NMR of compound **12d.**

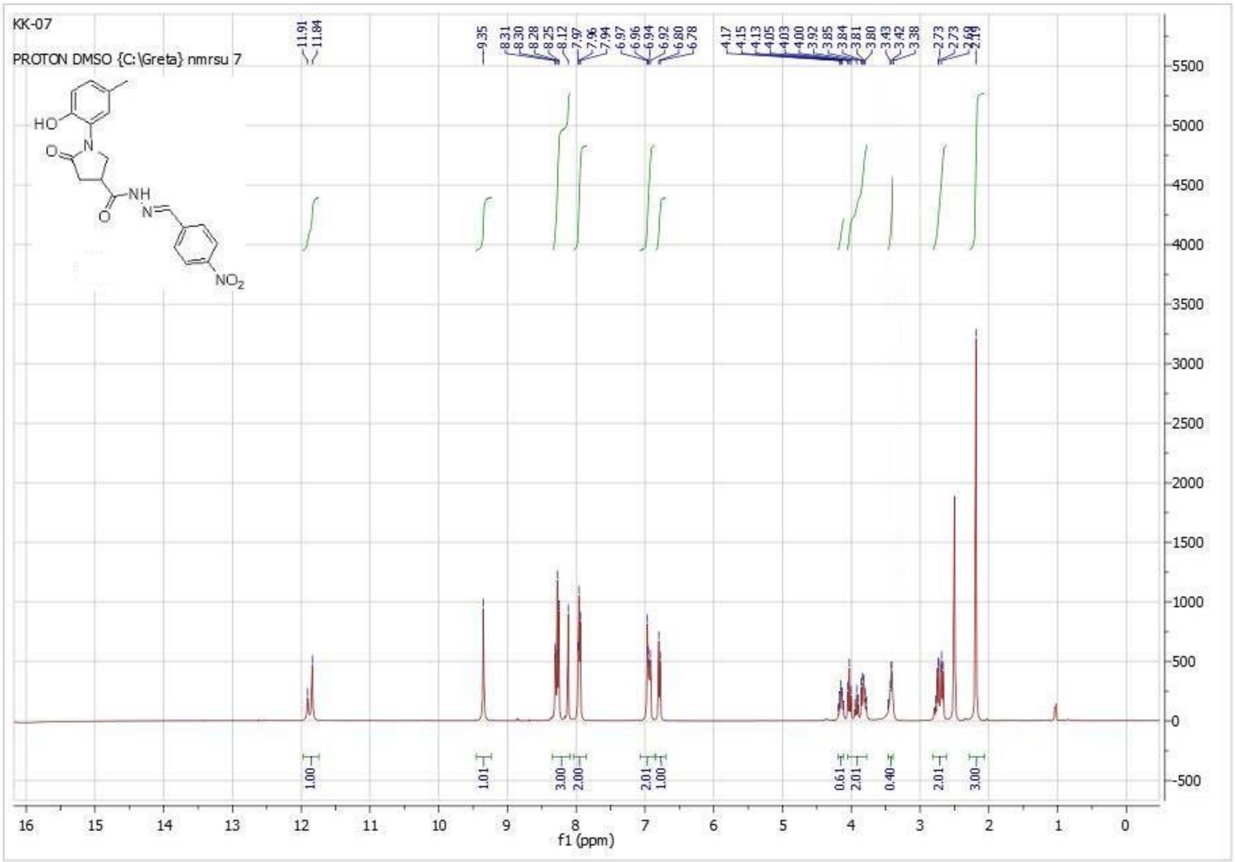

Figure S41. ^{1}H NMR of compound **12e.**

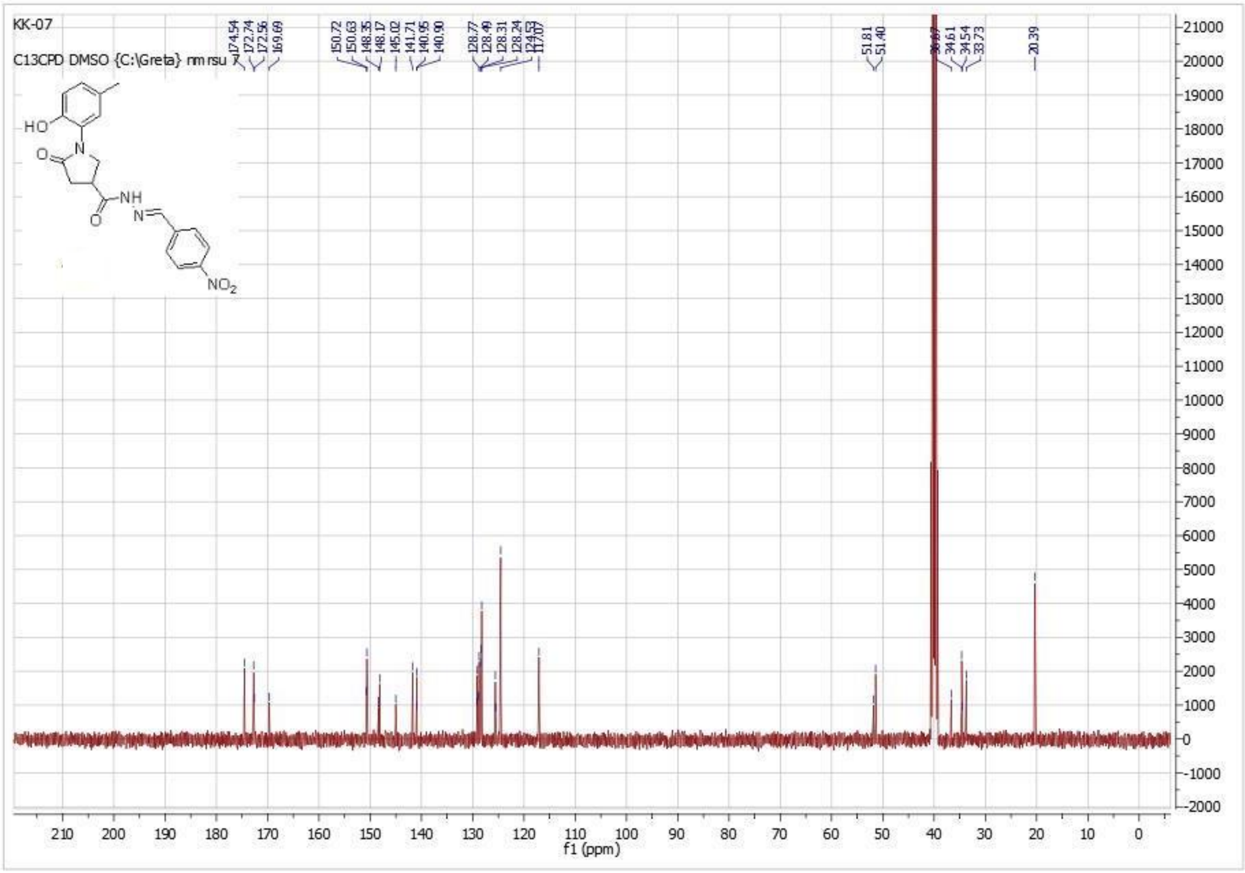

Figure S42. ^{13}C NMR of compound **12e.**

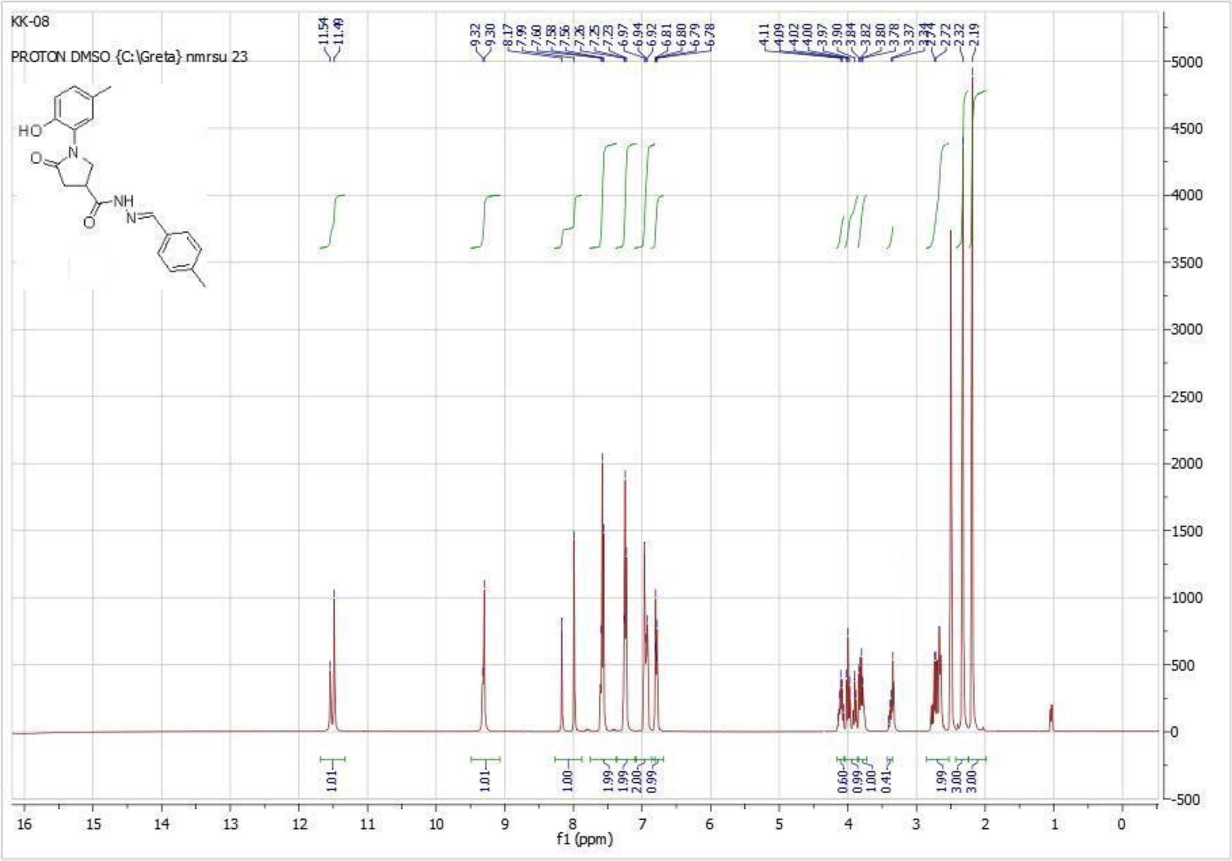

Figure S43. ^{1}H NMR of compound **12f.**

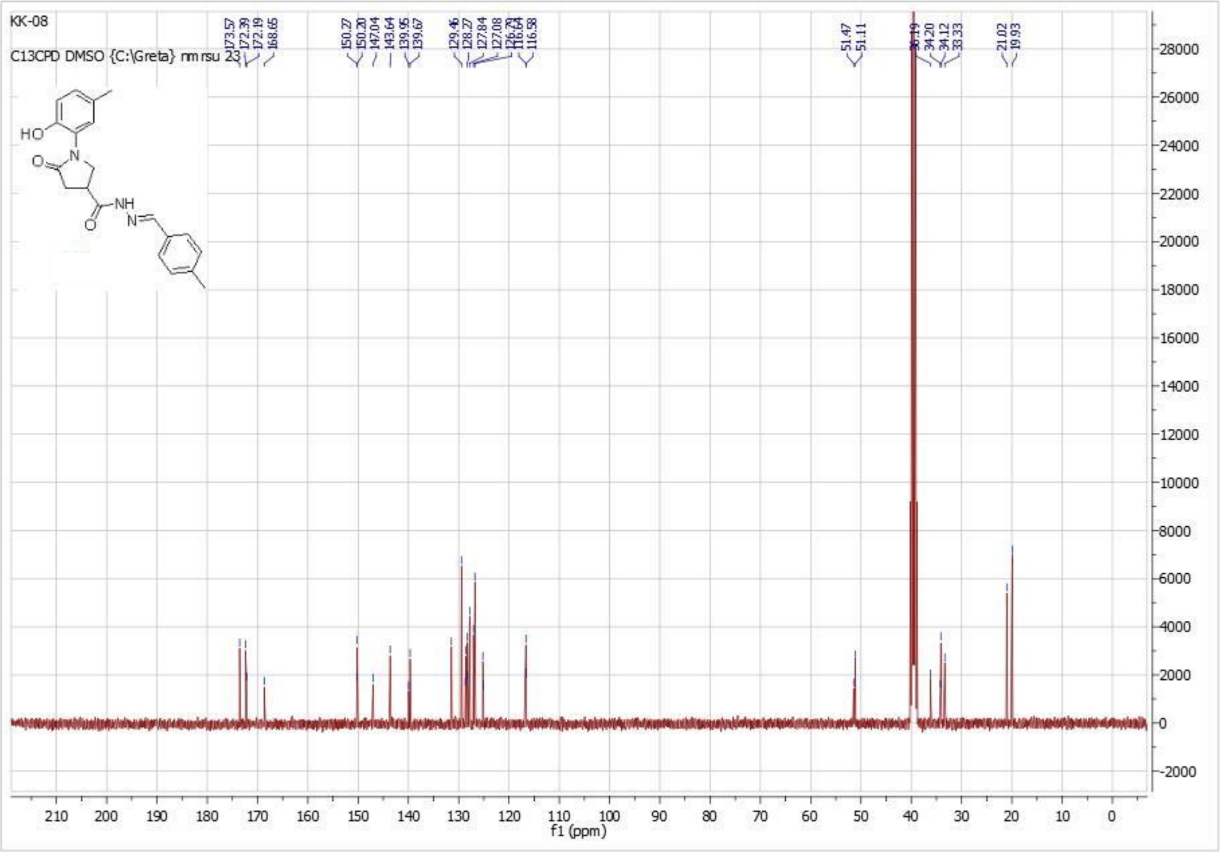

Figure S44. ^{13}C NMR of compound **12f.**

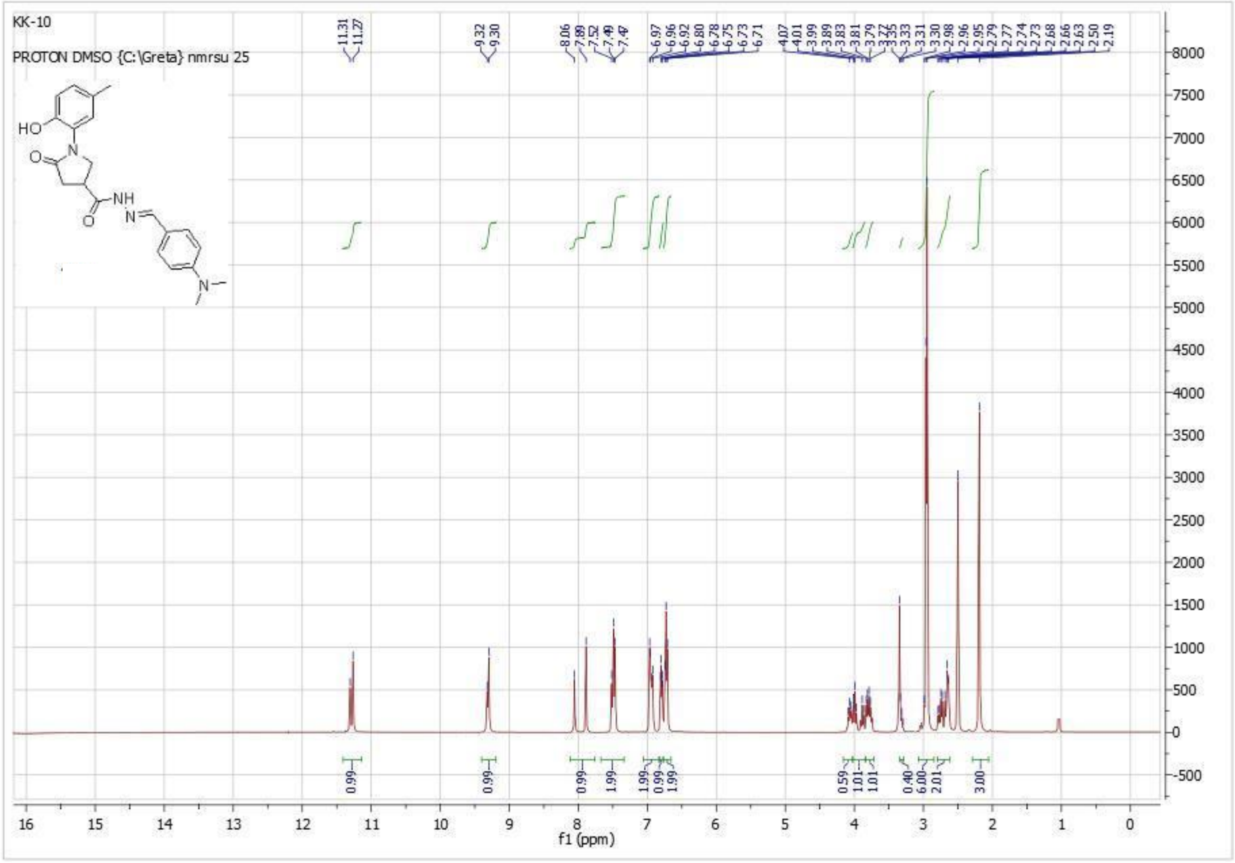

Figure S45. ^{1}H NMR of compound **12g.**

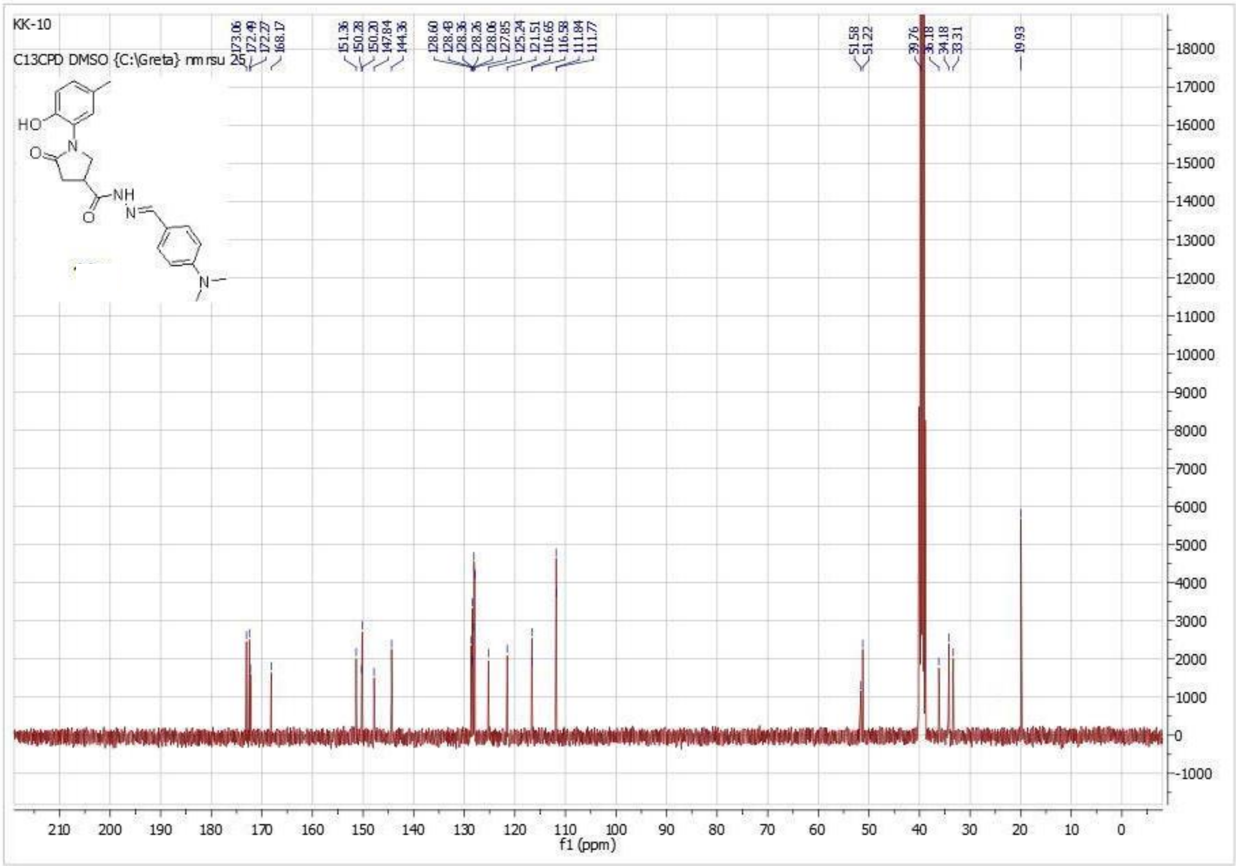

Figure S46. ^{13}C NMR of compound **12g.**

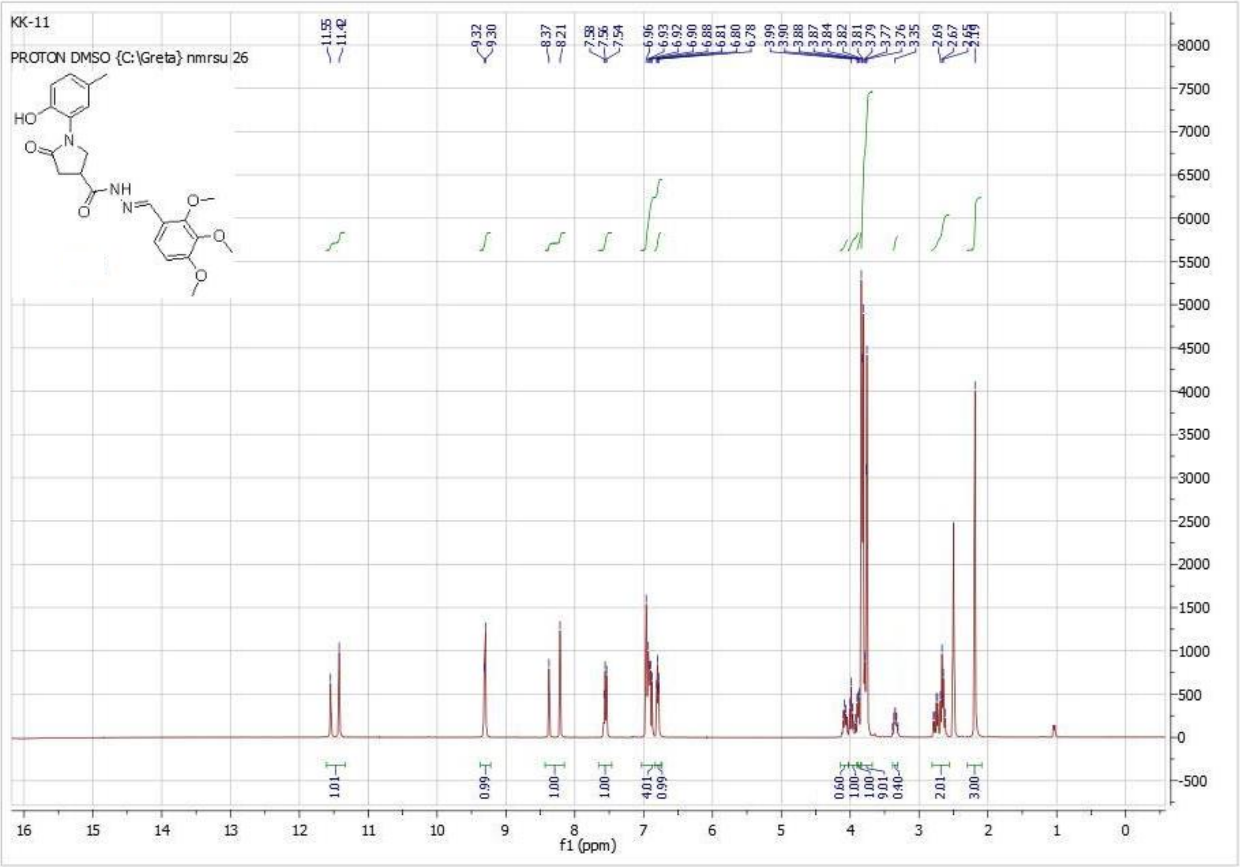

Figure S47. ^{1}H NMR of compound **12h.**

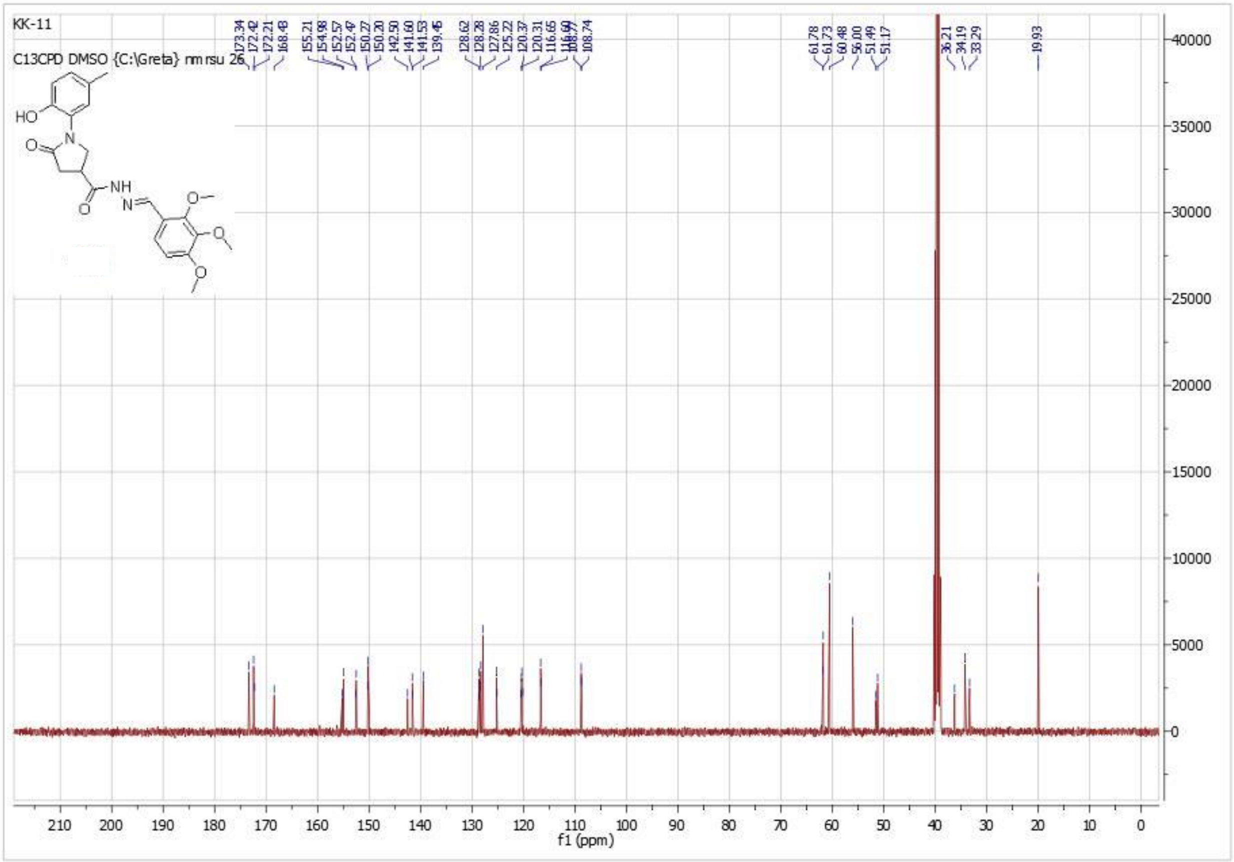

Figure S48. ^{13}C NMR of compound **12h.**

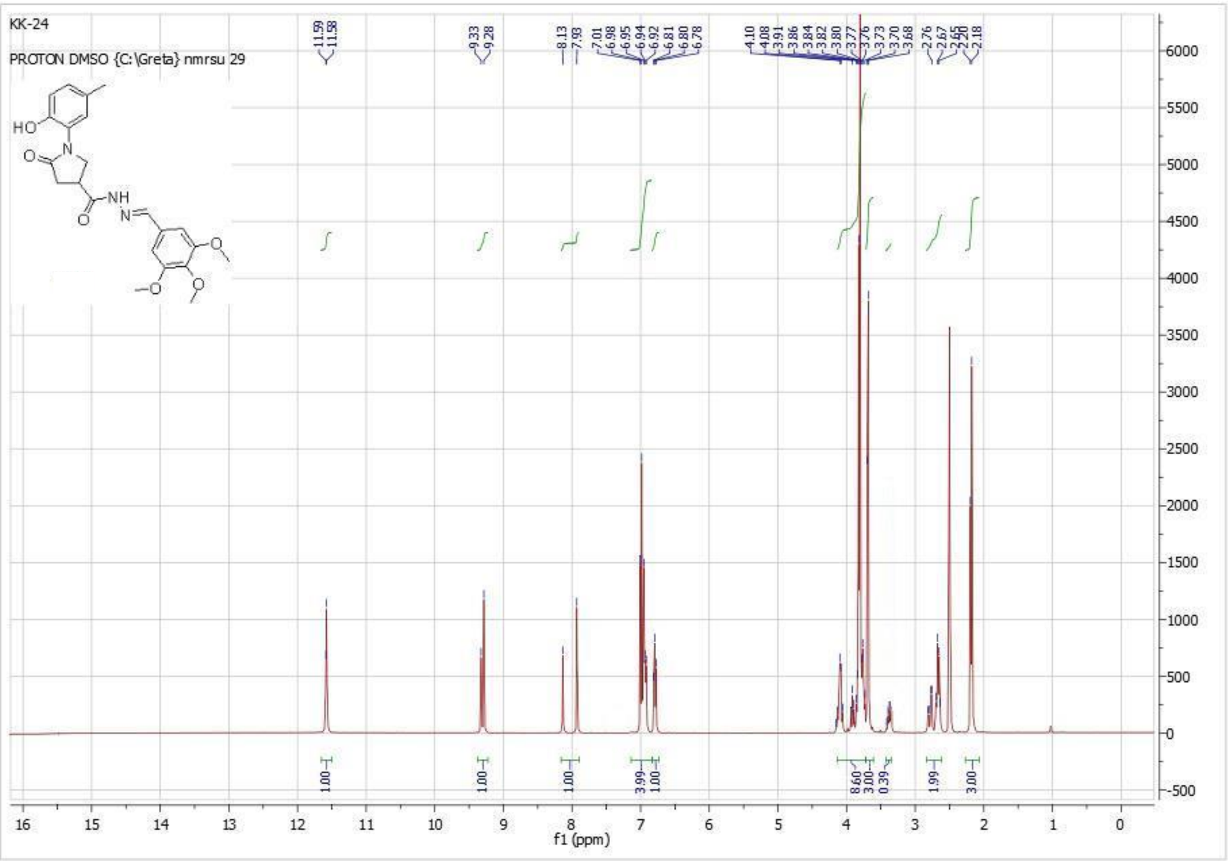

Figure S49. ^{1}H NMR of compound **12i.**

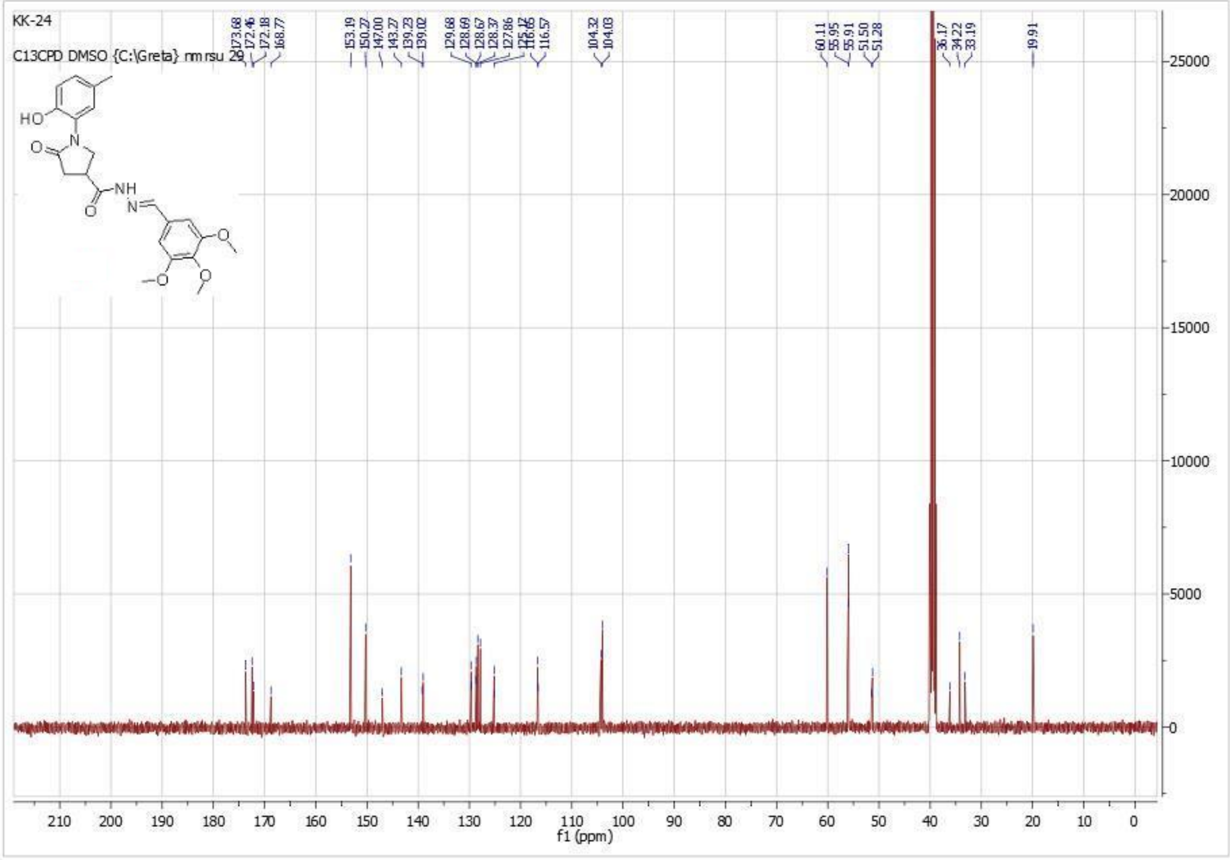

Figure S50. ^{13}C NMR of compound **12i.**

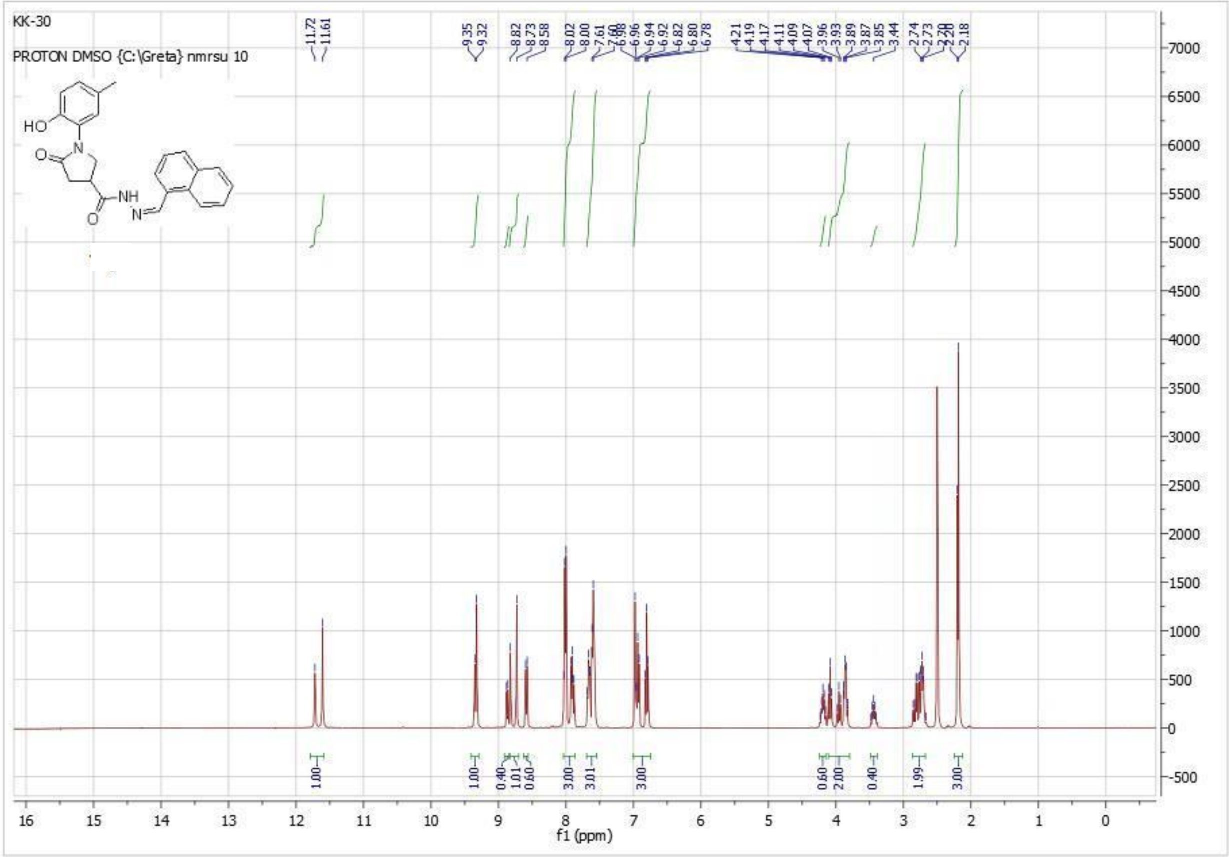

Figure S51. ^{1}H NMR of compound **12j.**

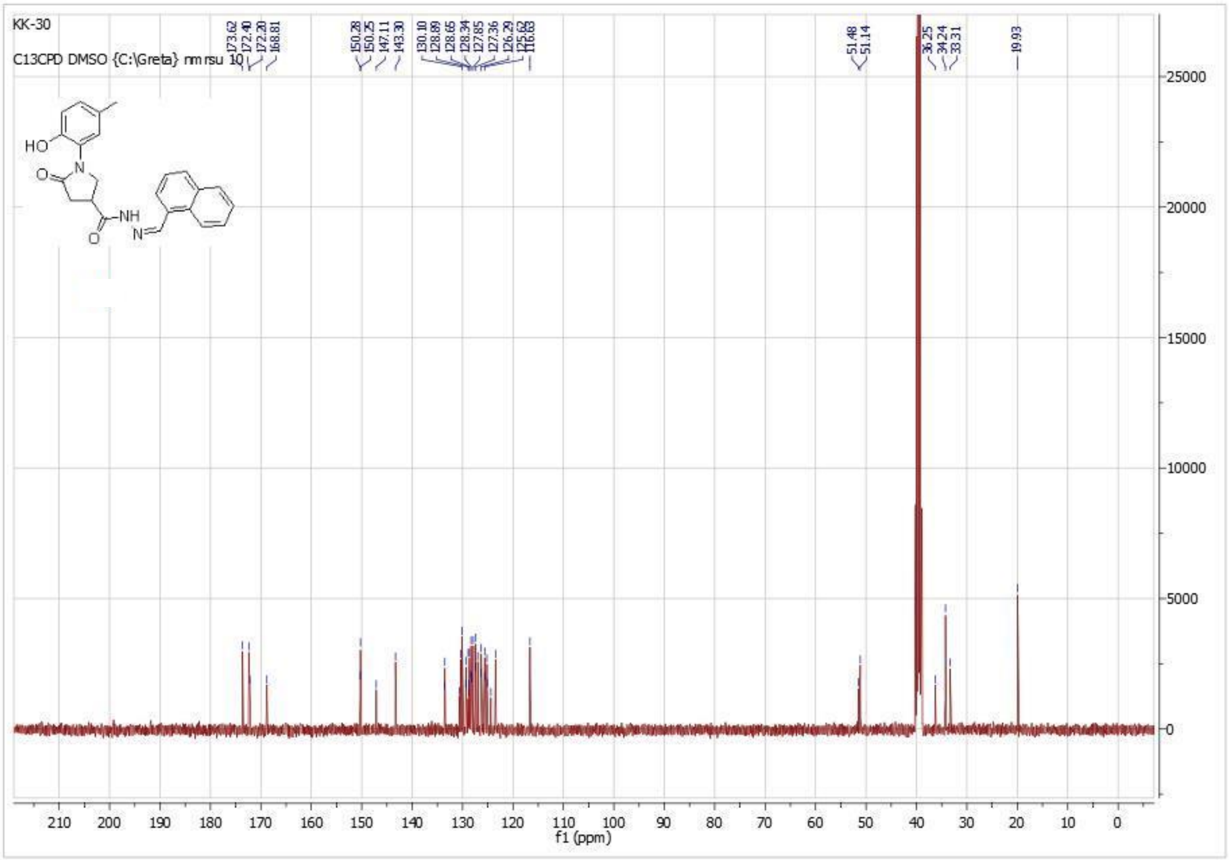

Figure S52. ^{13}C NMR of compound **12j.**

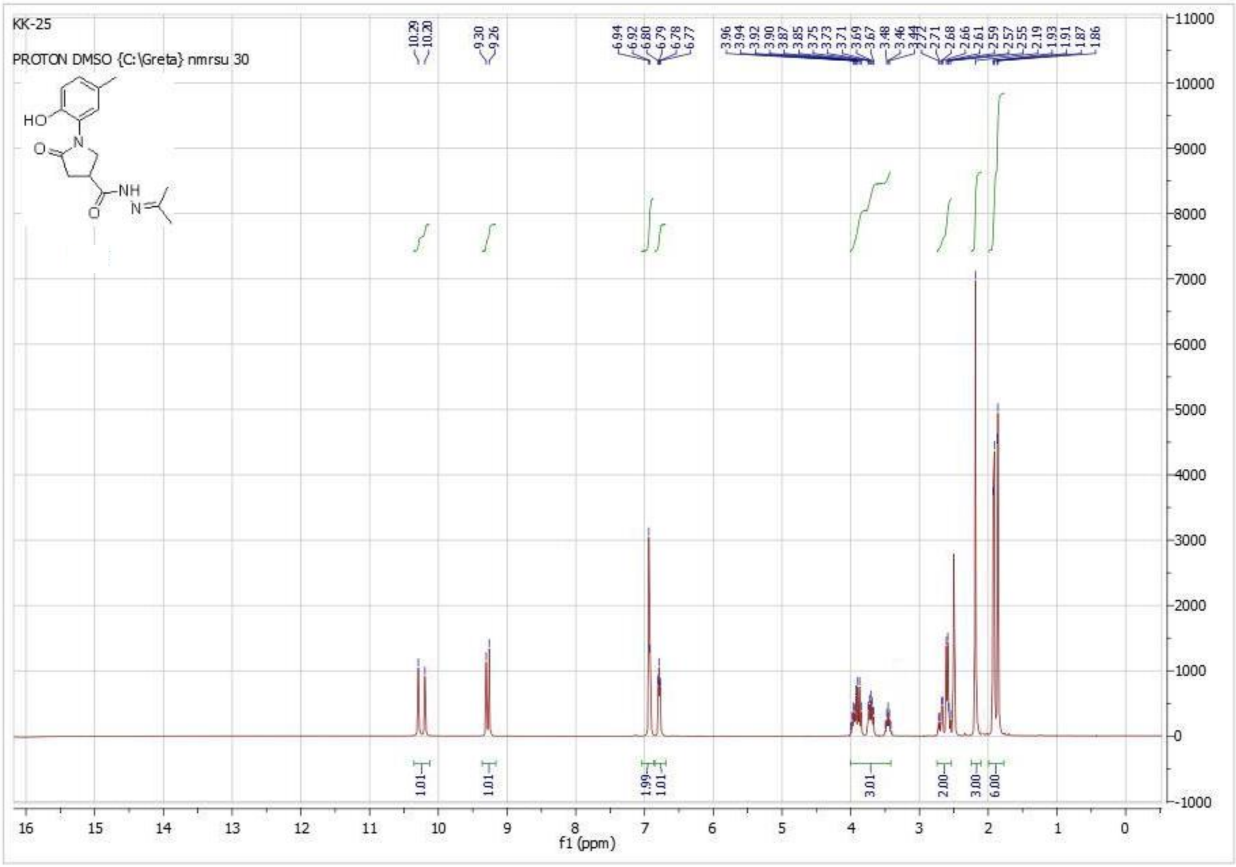

Figure S53. ^{1}H NMR of compound **13a.**

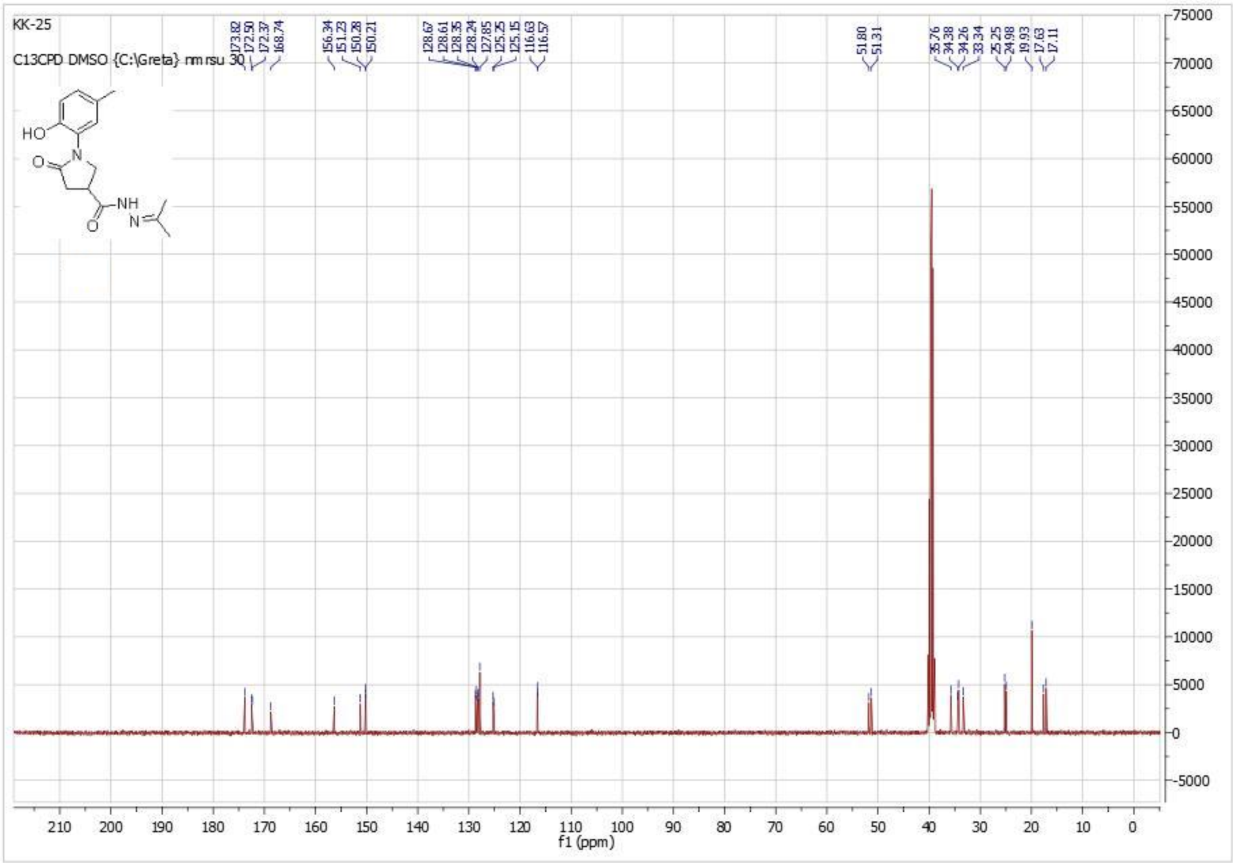

Figure S54. ^{13}C NMR of compound **13a.**

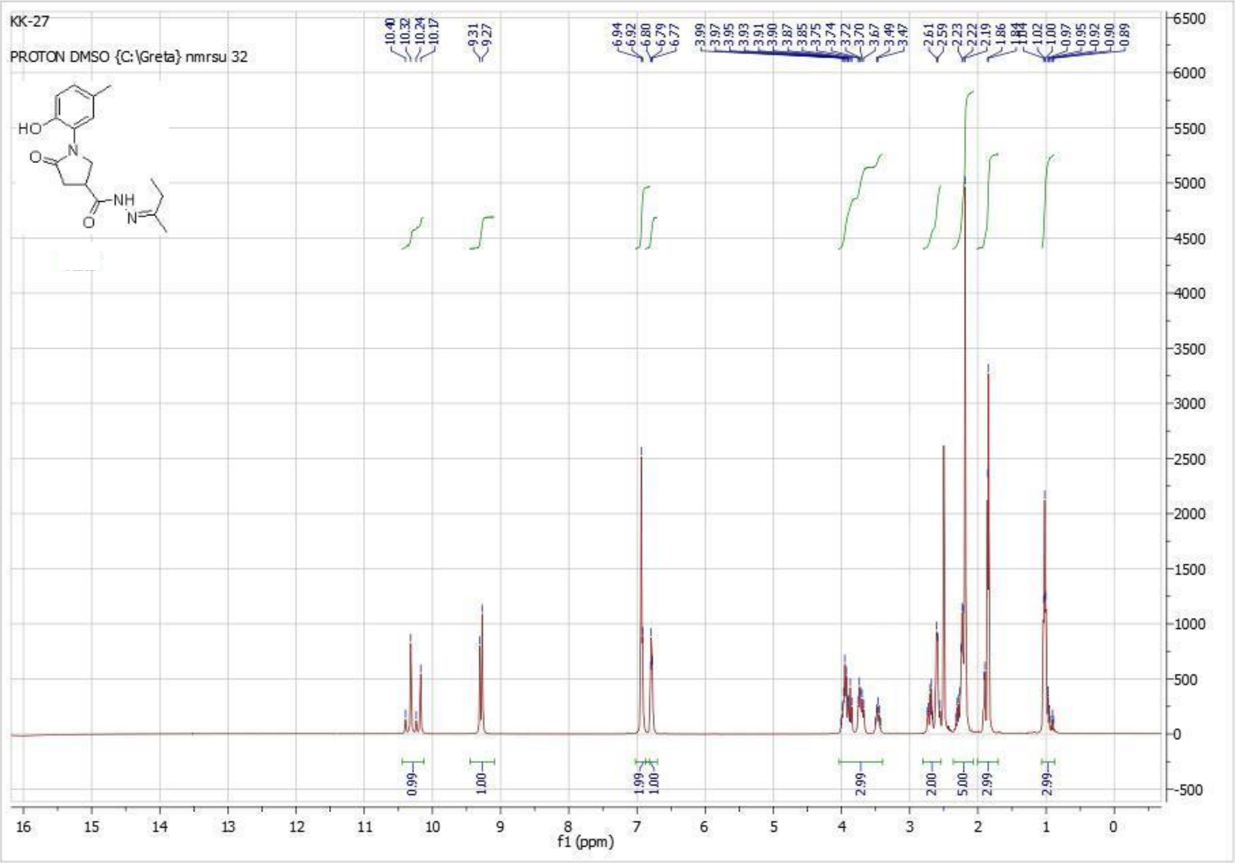

Figure S55. ^{1}H NMR of compound **13b.**

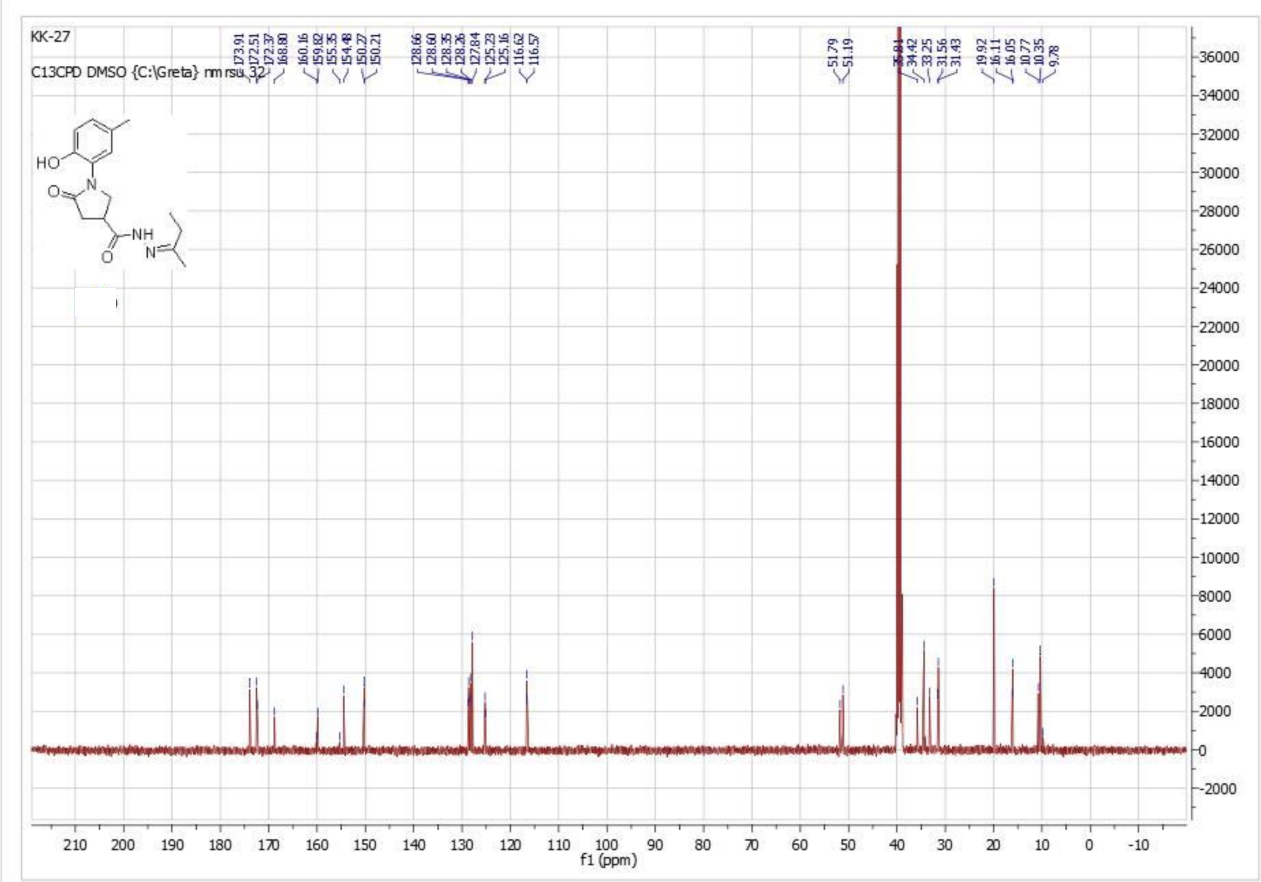

Figure S56. ^{13}C NMR of compound **13b.**

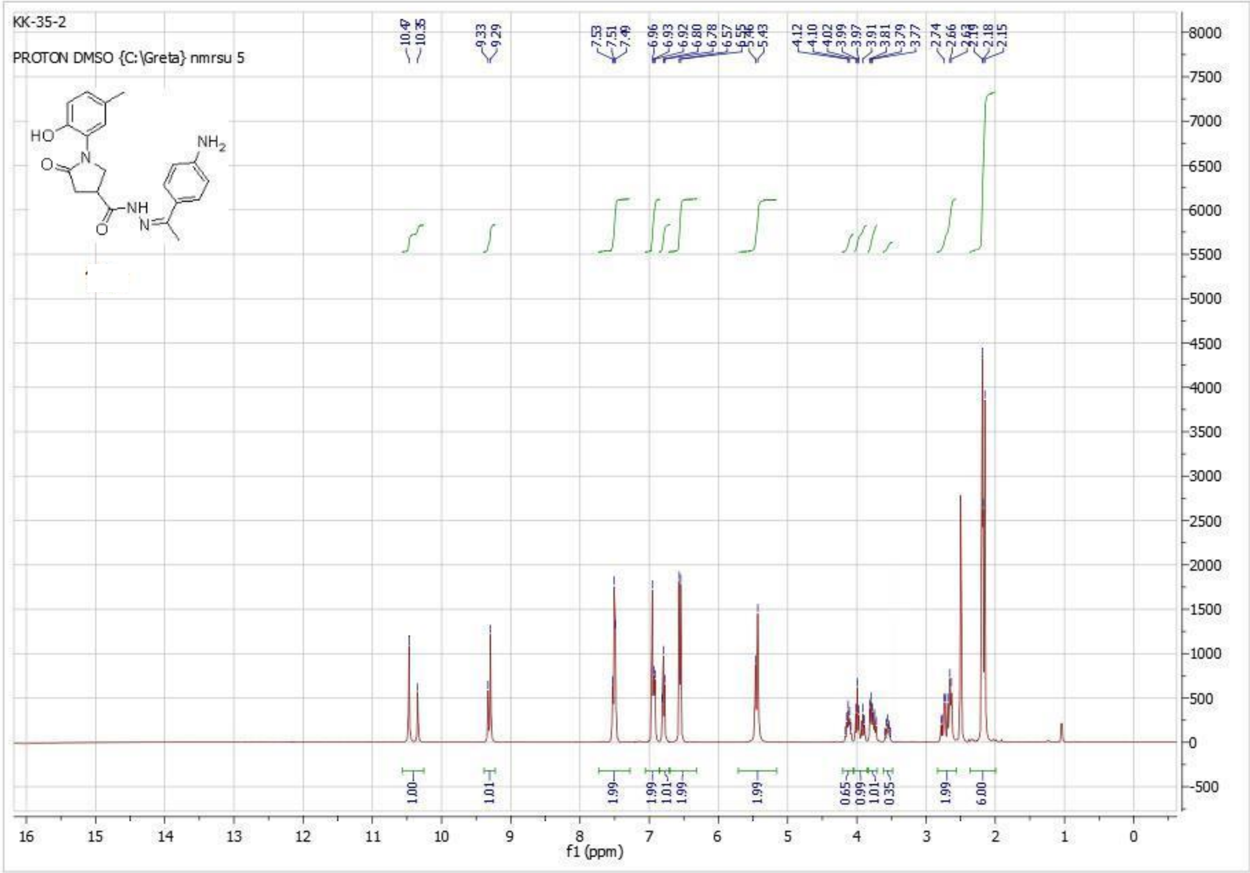

Figure S57. ^{1}H NMR of compound **13c.**

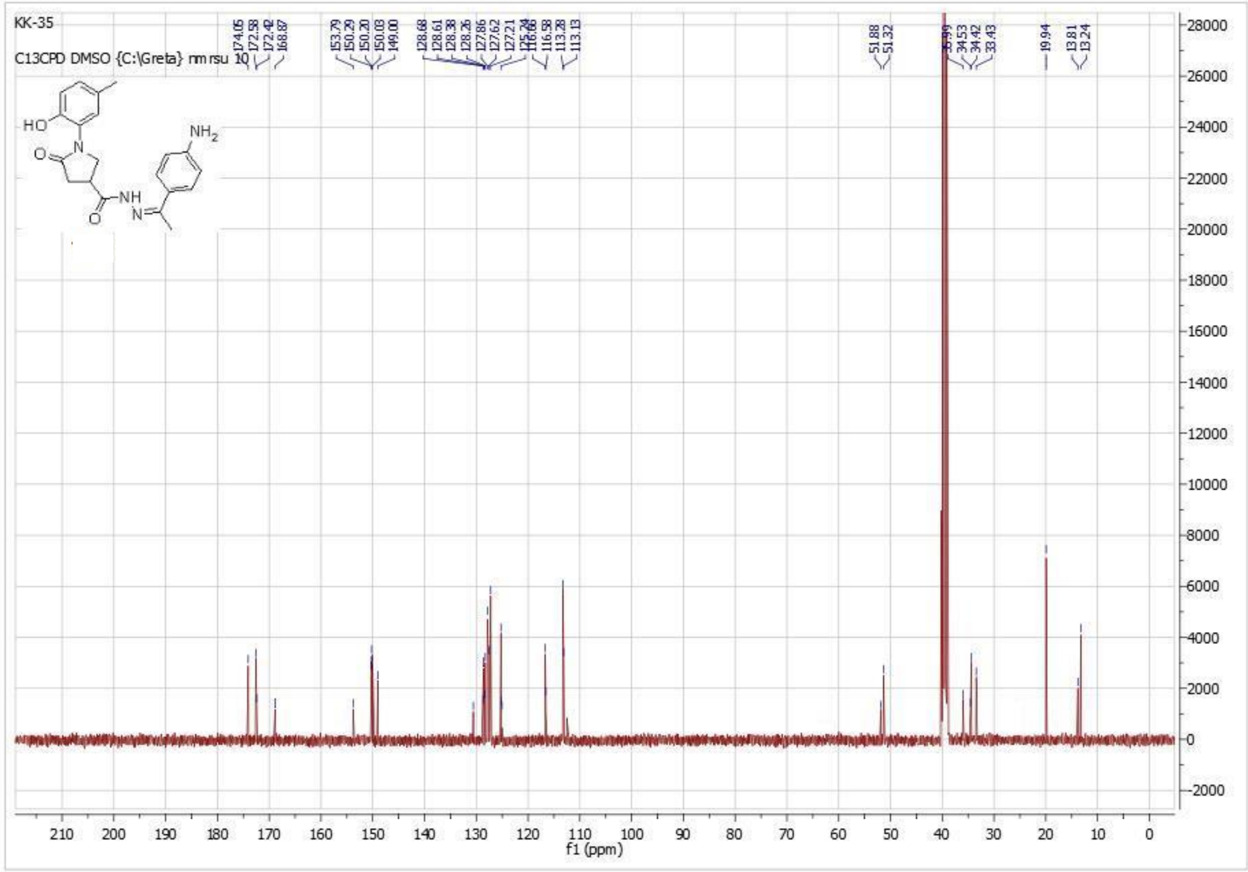

Figure S58. ^{13}C NMR of compound **13c.**

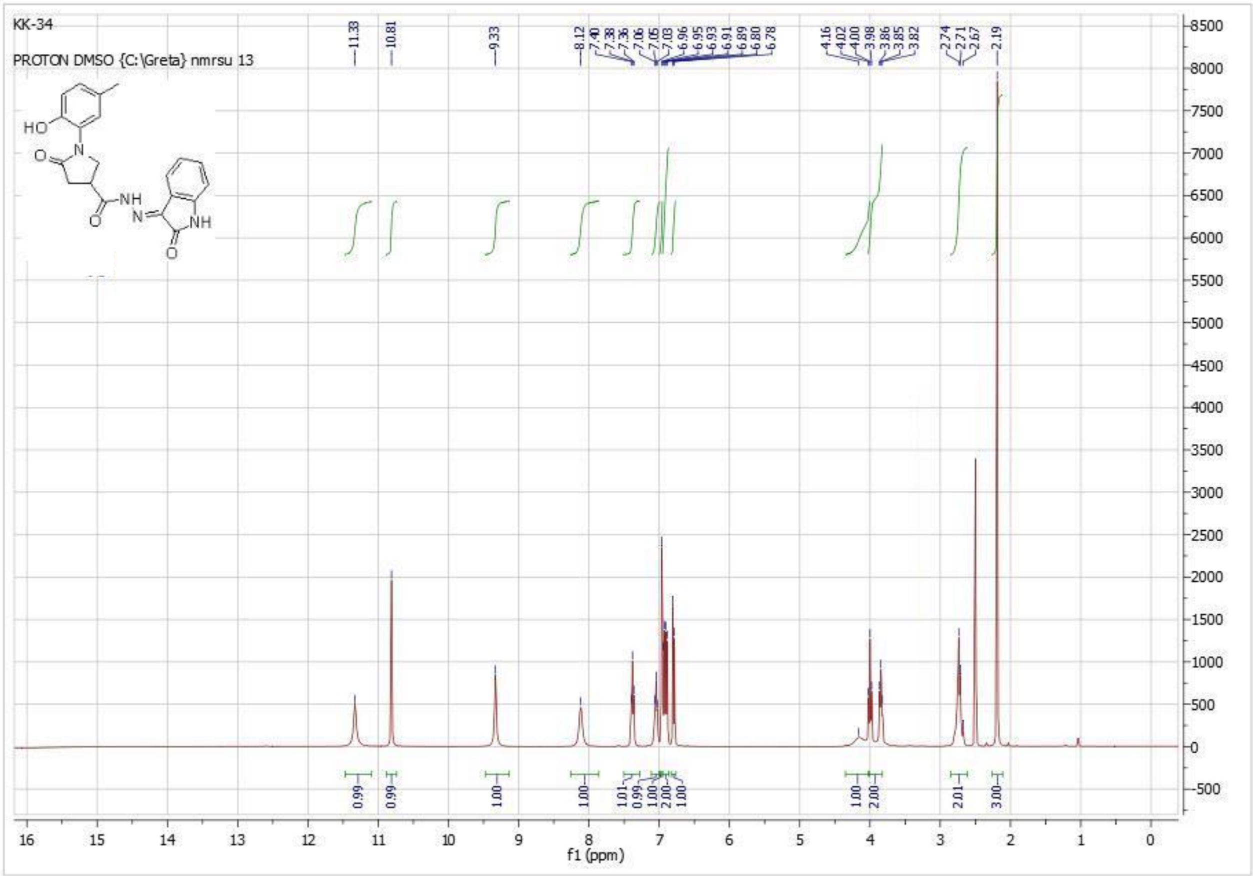

Figure S59. ^{1}H NMR of compound **14.**

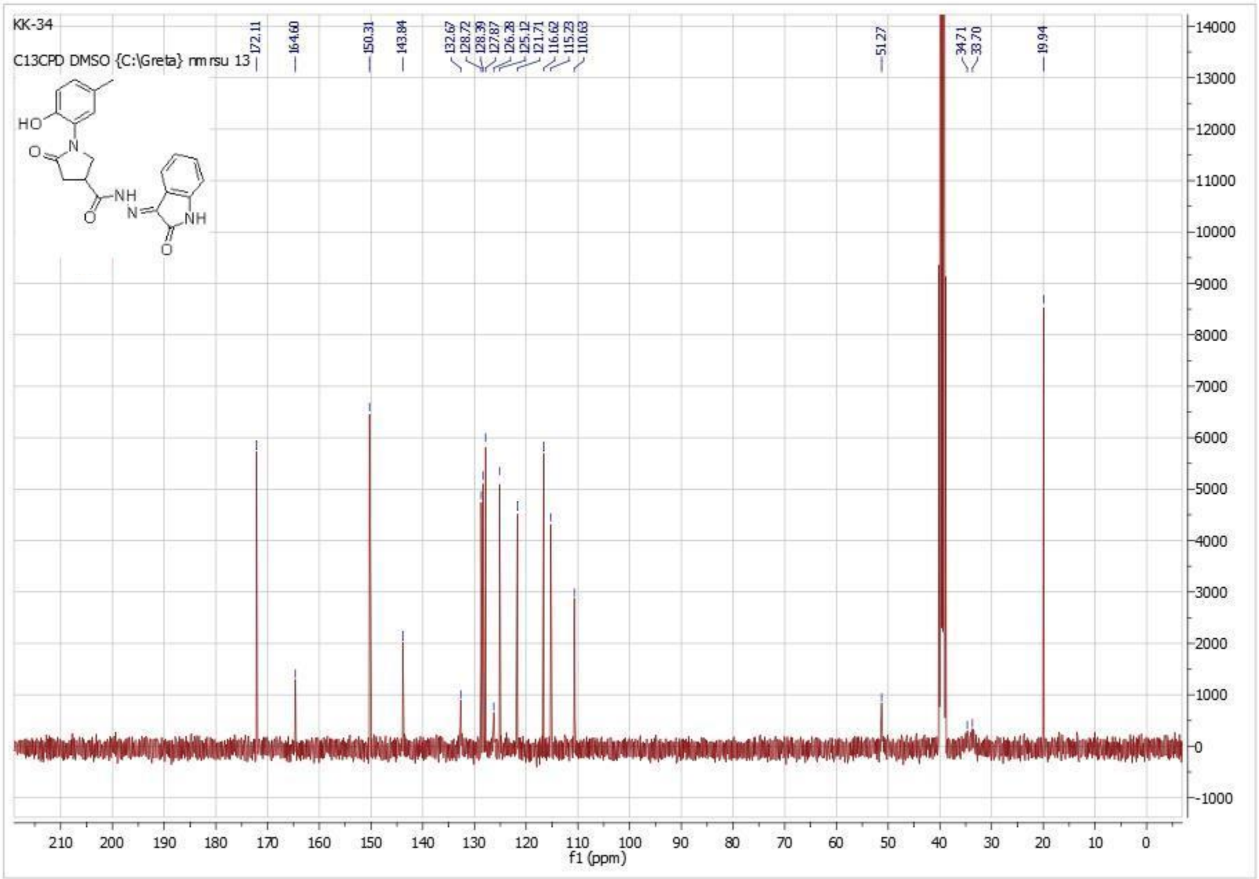

Figure S60. ^{13}C NMR of compound **14.**

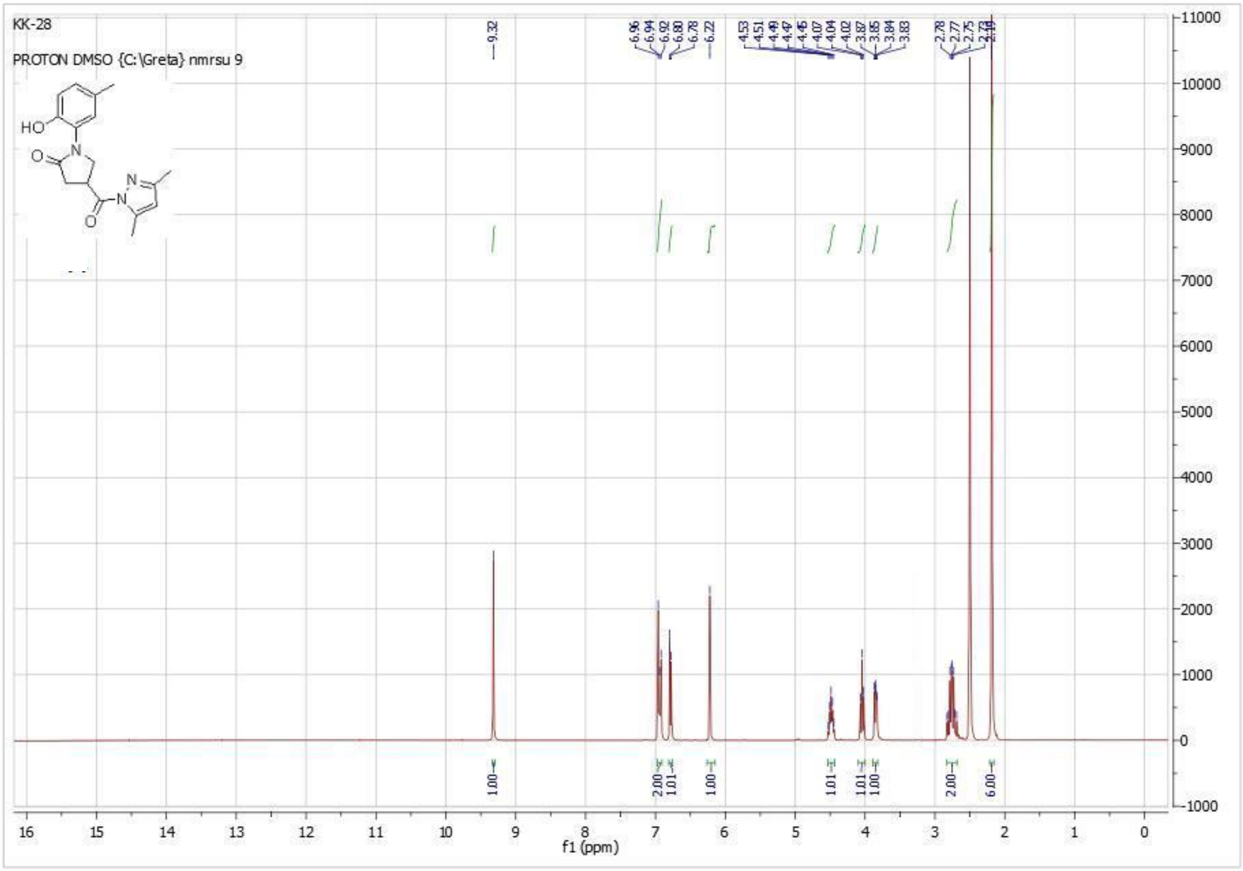

Figure S61. ^{1}H NMR of compound **15.**

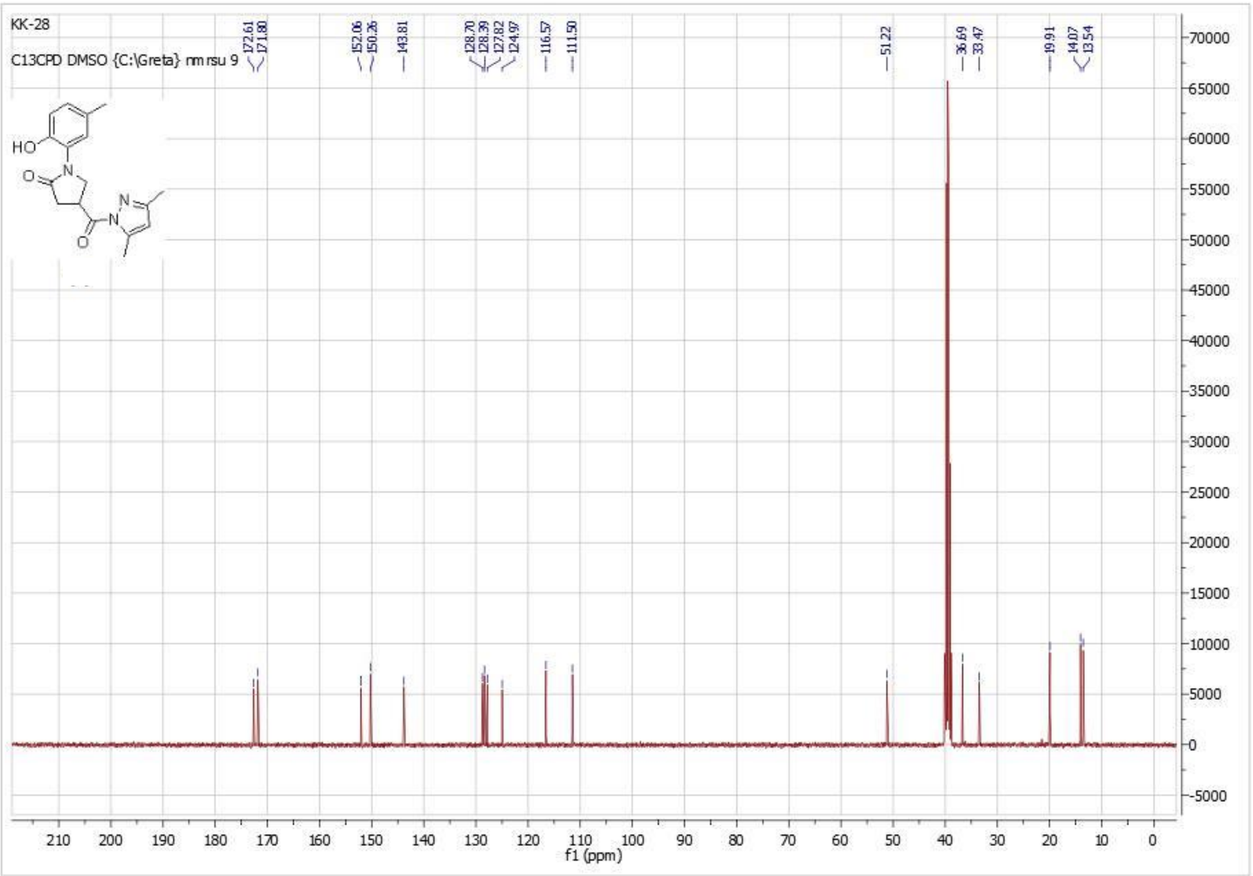

Figure S62. ^{13}C NMR of compound **15.**

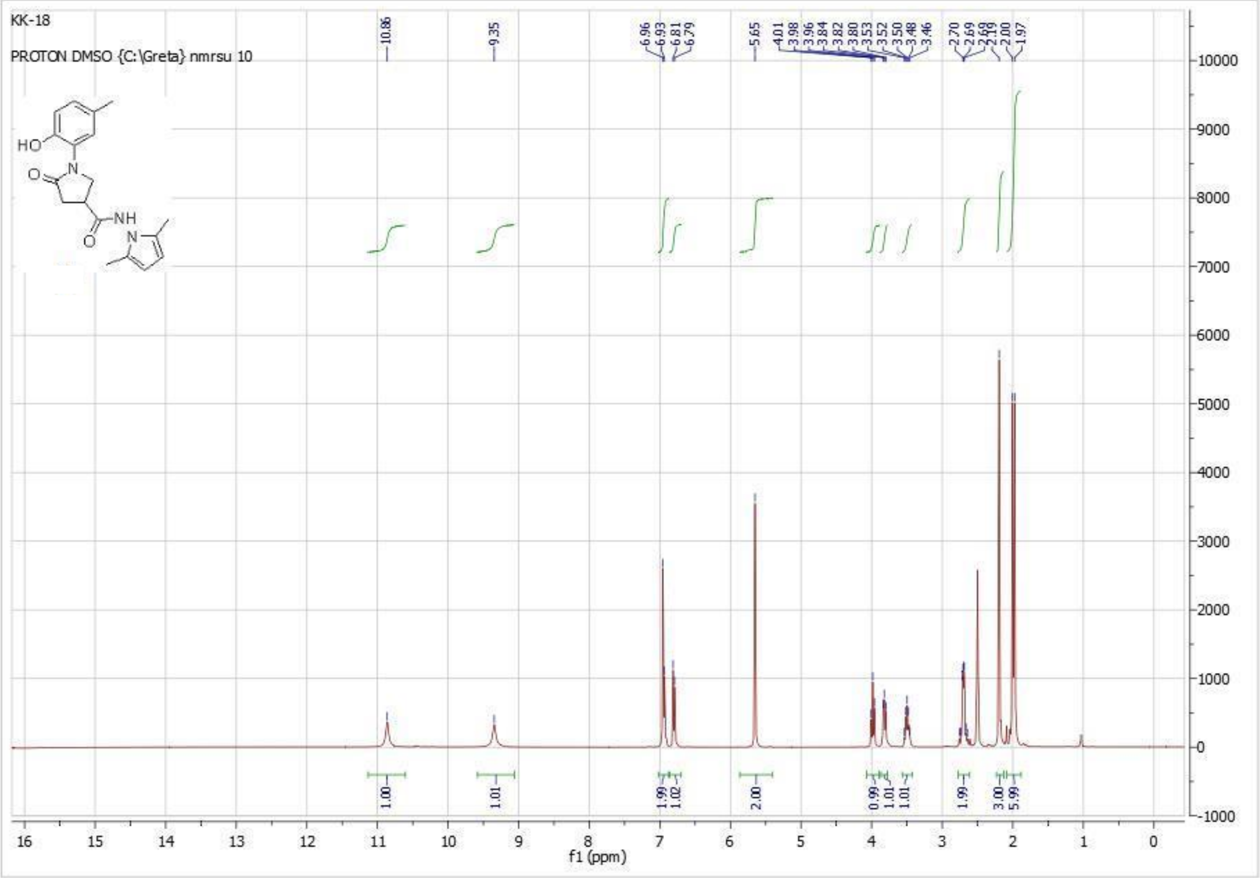

Figure S63. ^{1}H NMR of compound **16.**

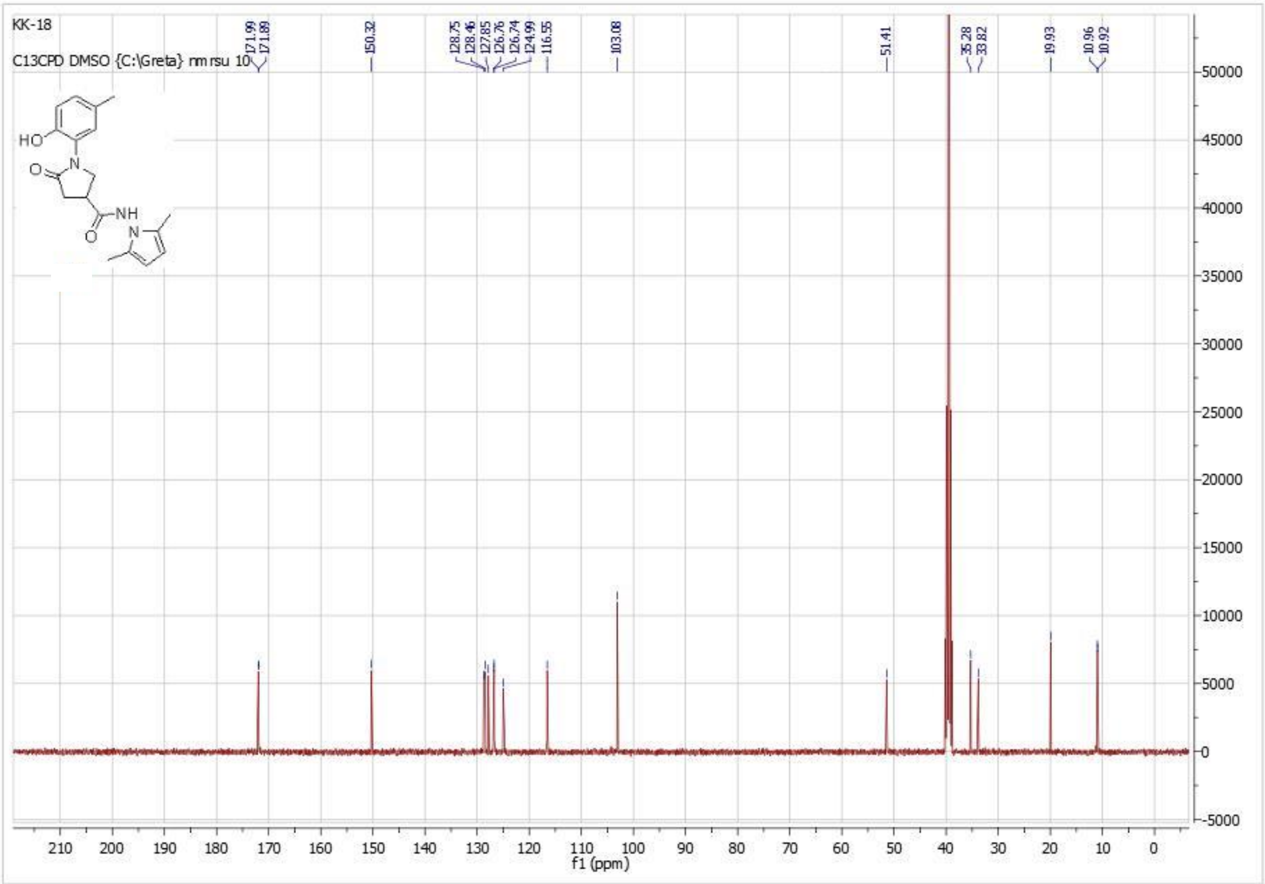

Figure S64. ^{13}C NMR of compound **16.**

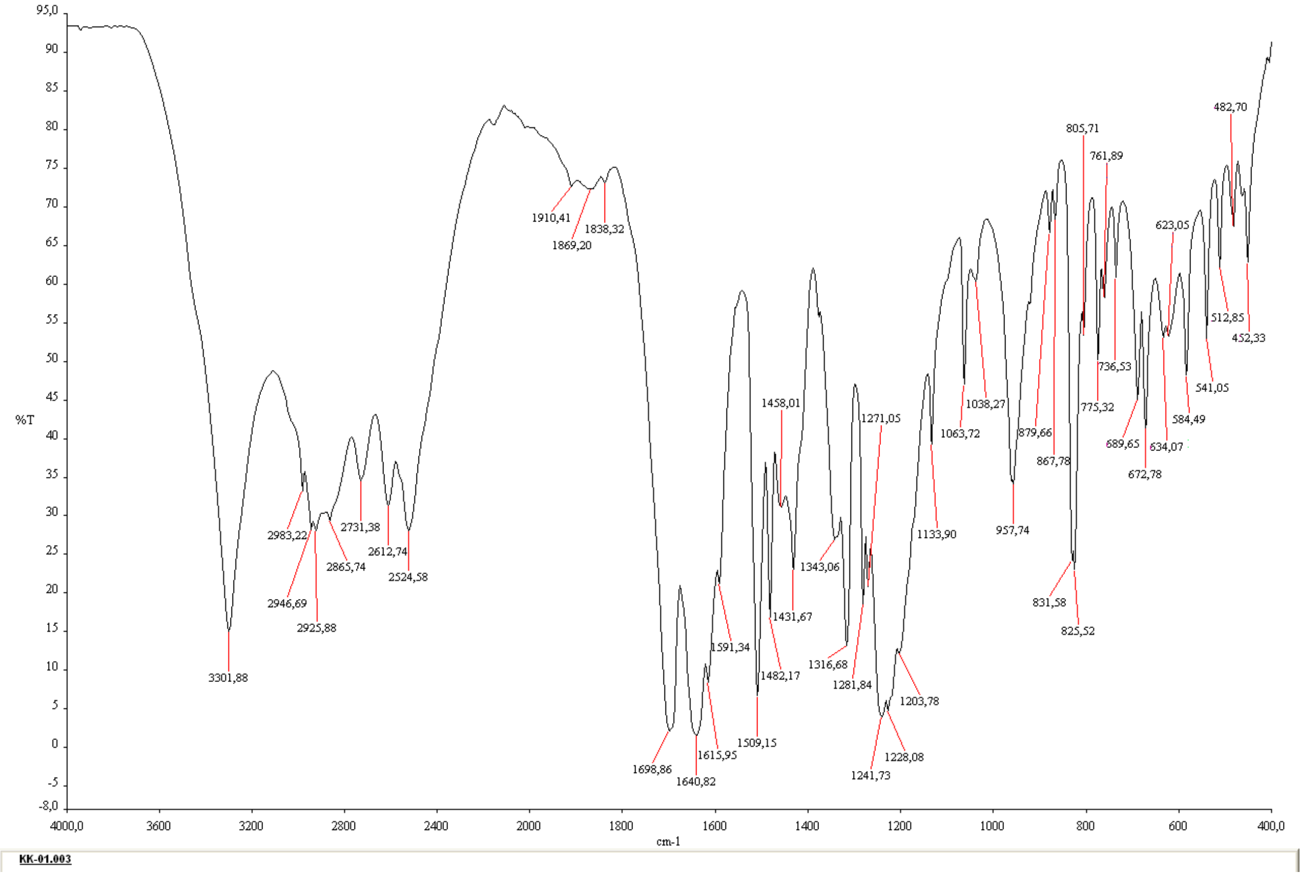

Figure S65. IR spectrum of compound **2**.

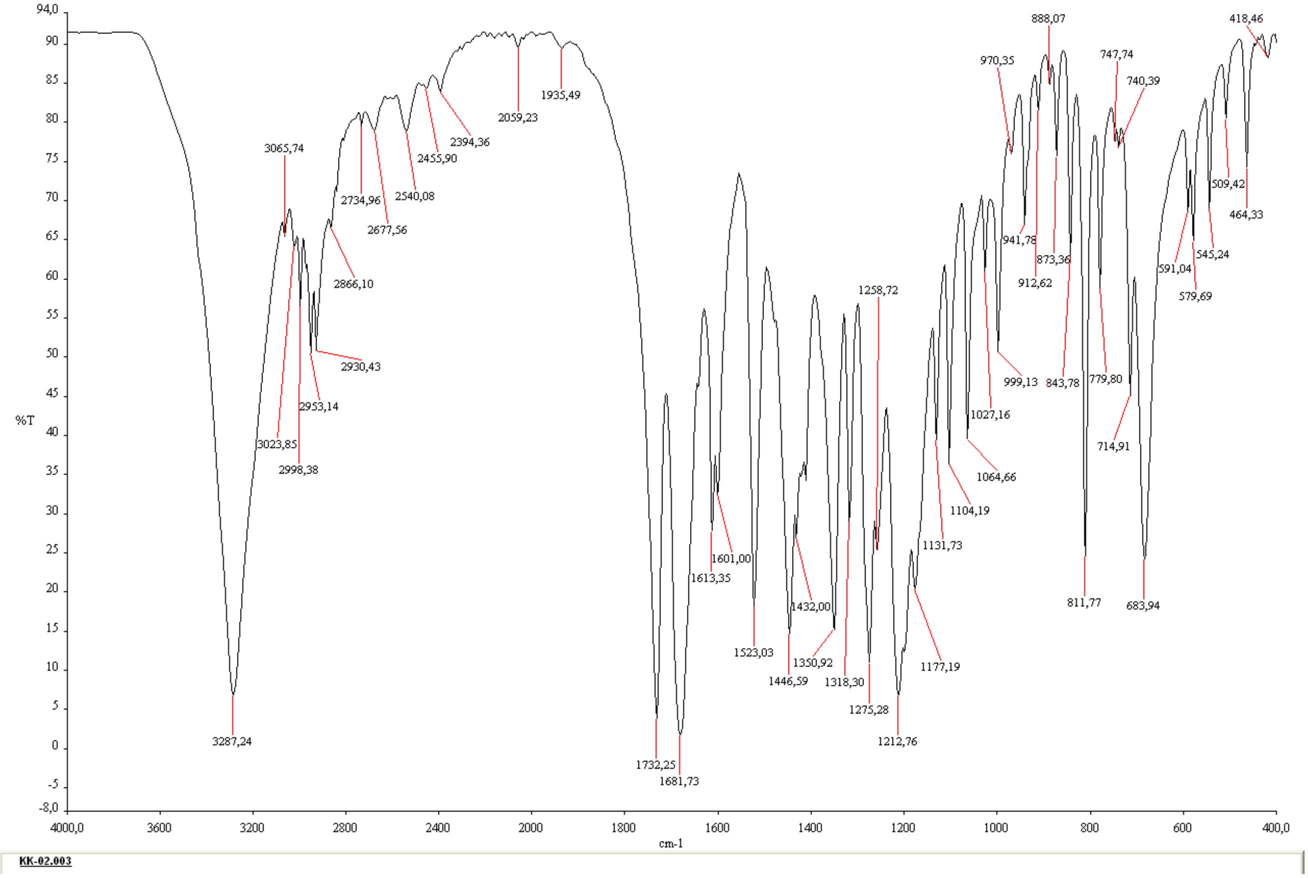

Figure S66. IR spectrum of compound **3**.

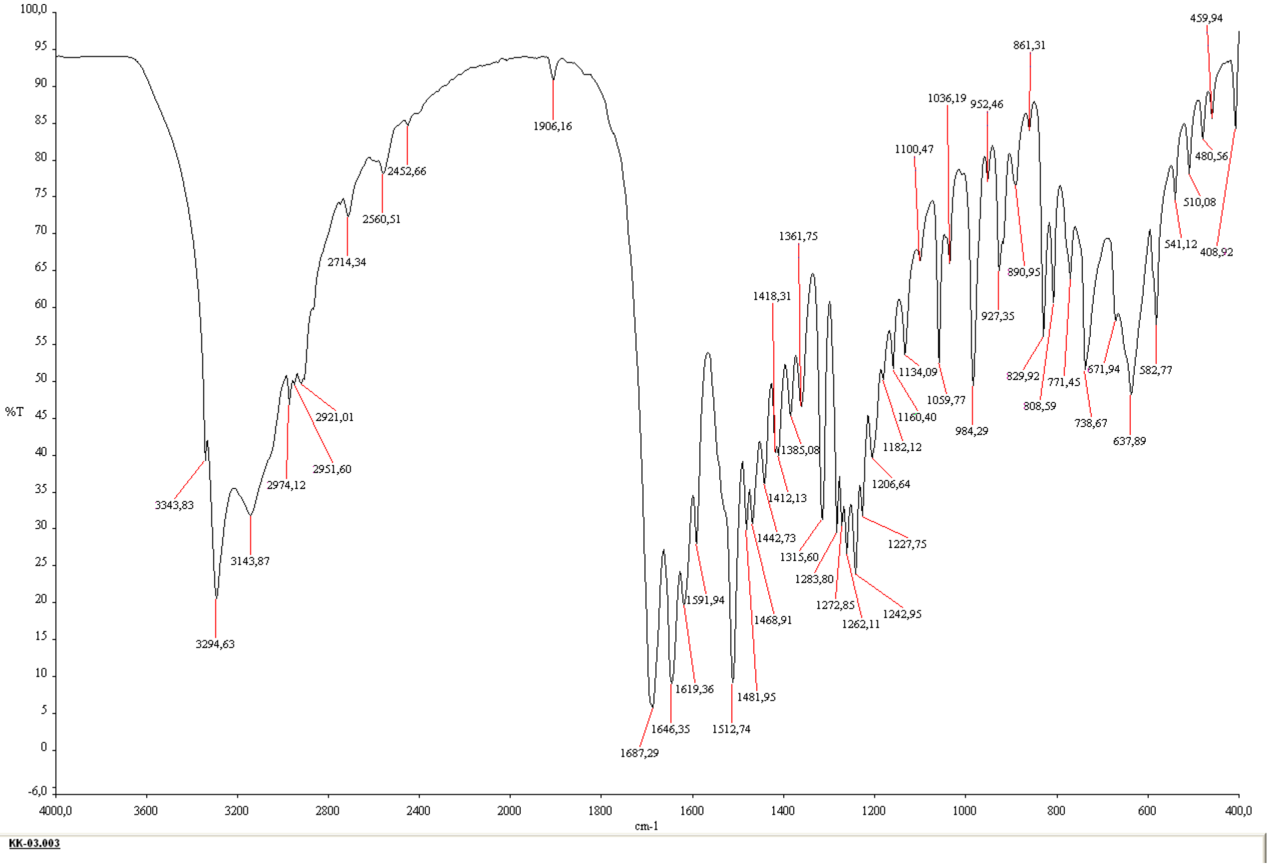

Figure S67. IR spectrum of compound **4**.

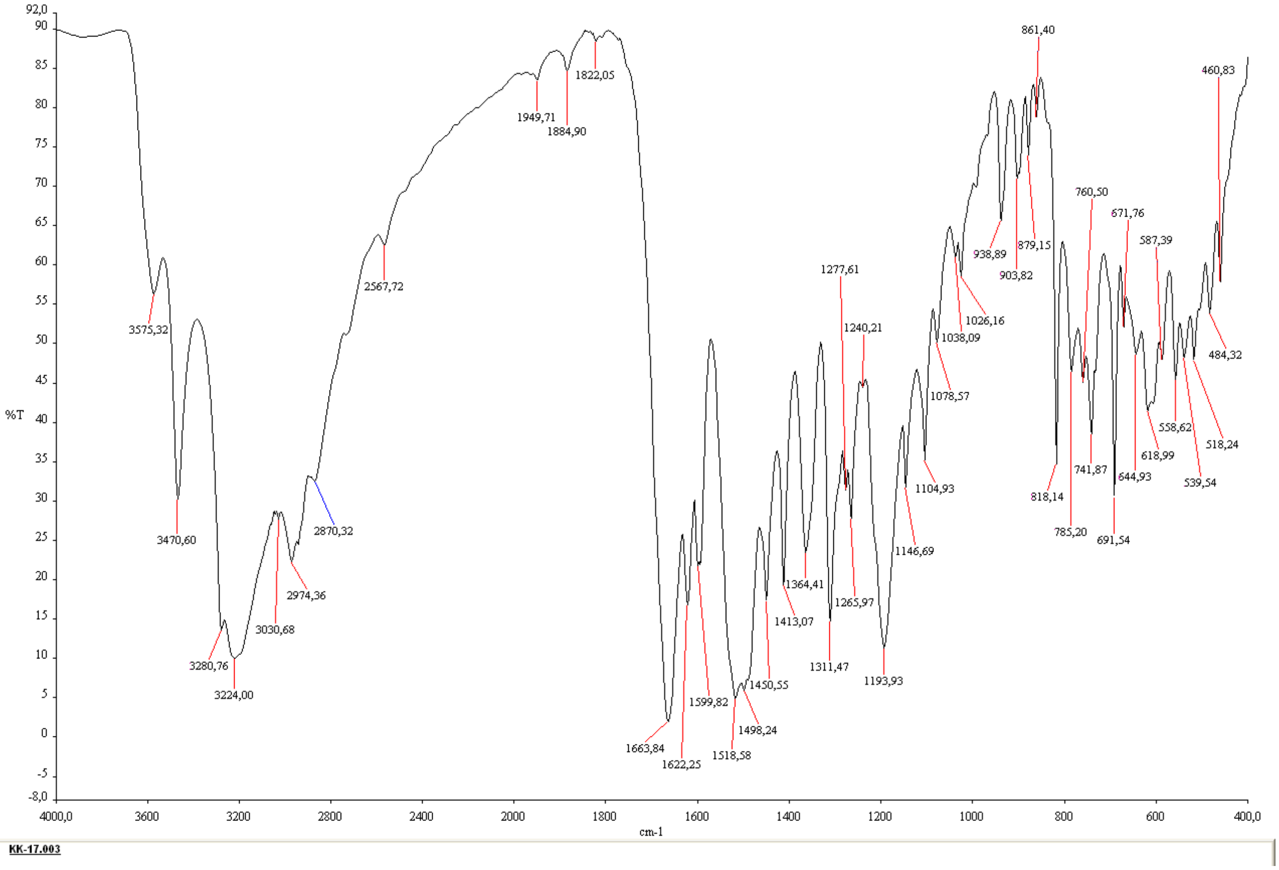

Figure S68. IR spectrum of compound **5**.

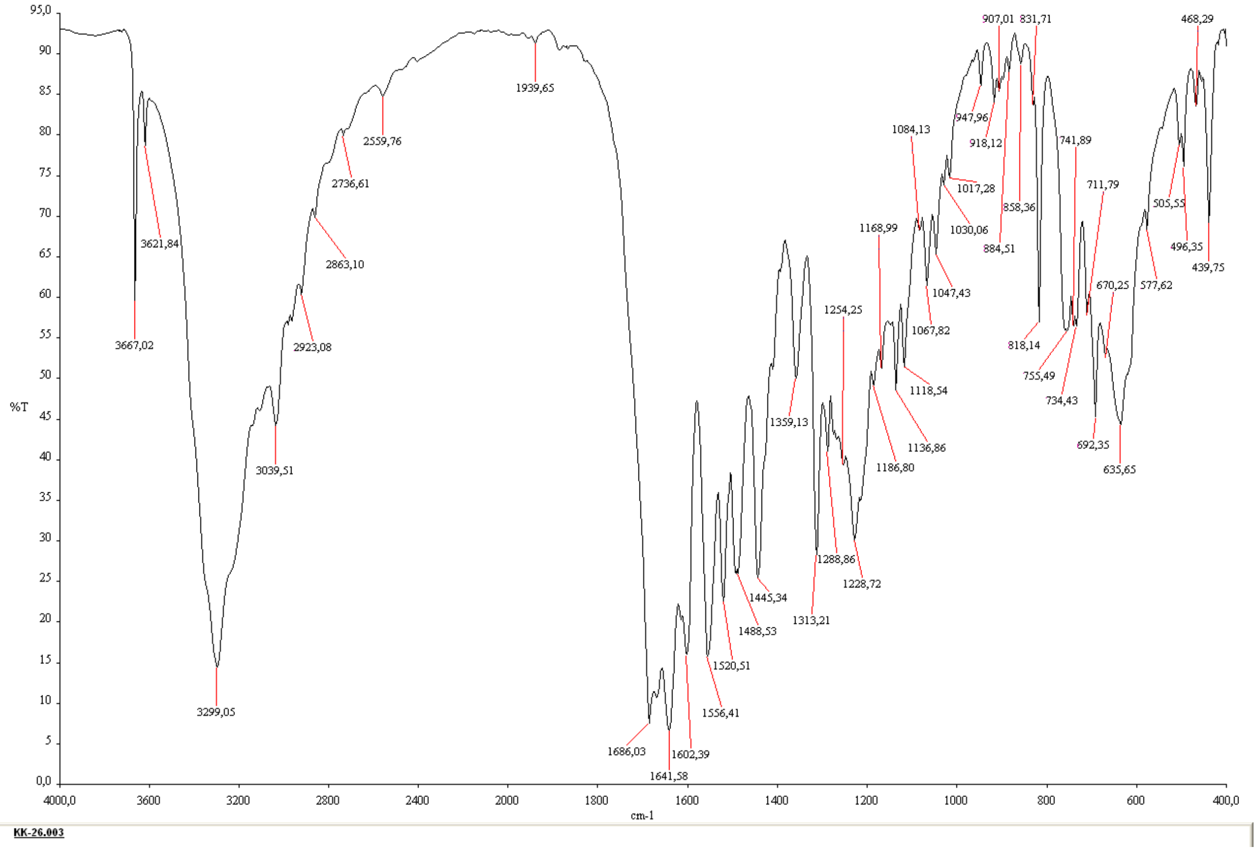

Figure S69. IR spectrum of compound **6**.

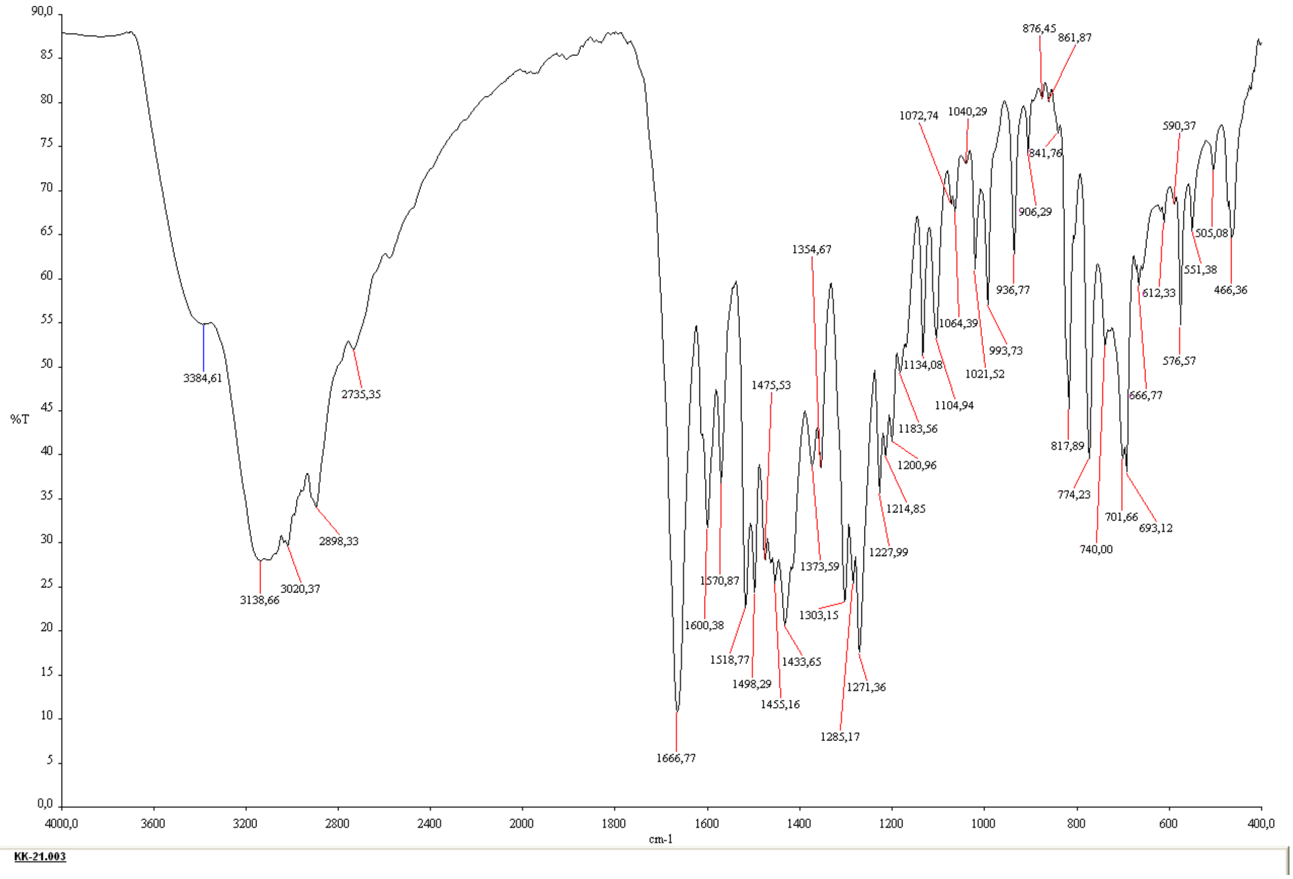

Figure S70. IR spectrum of compound **7**.

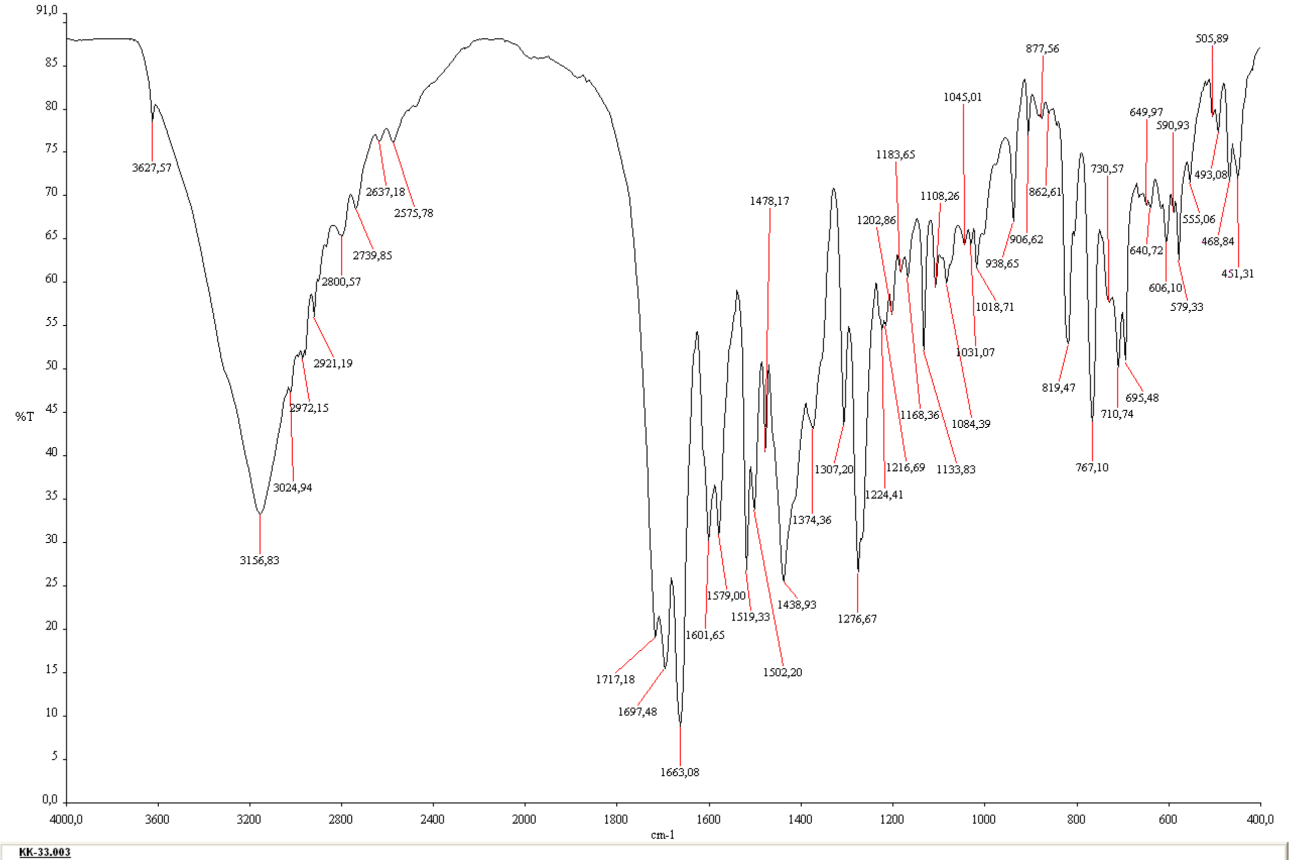

Figure S71. IR spectrum of compound **8**.

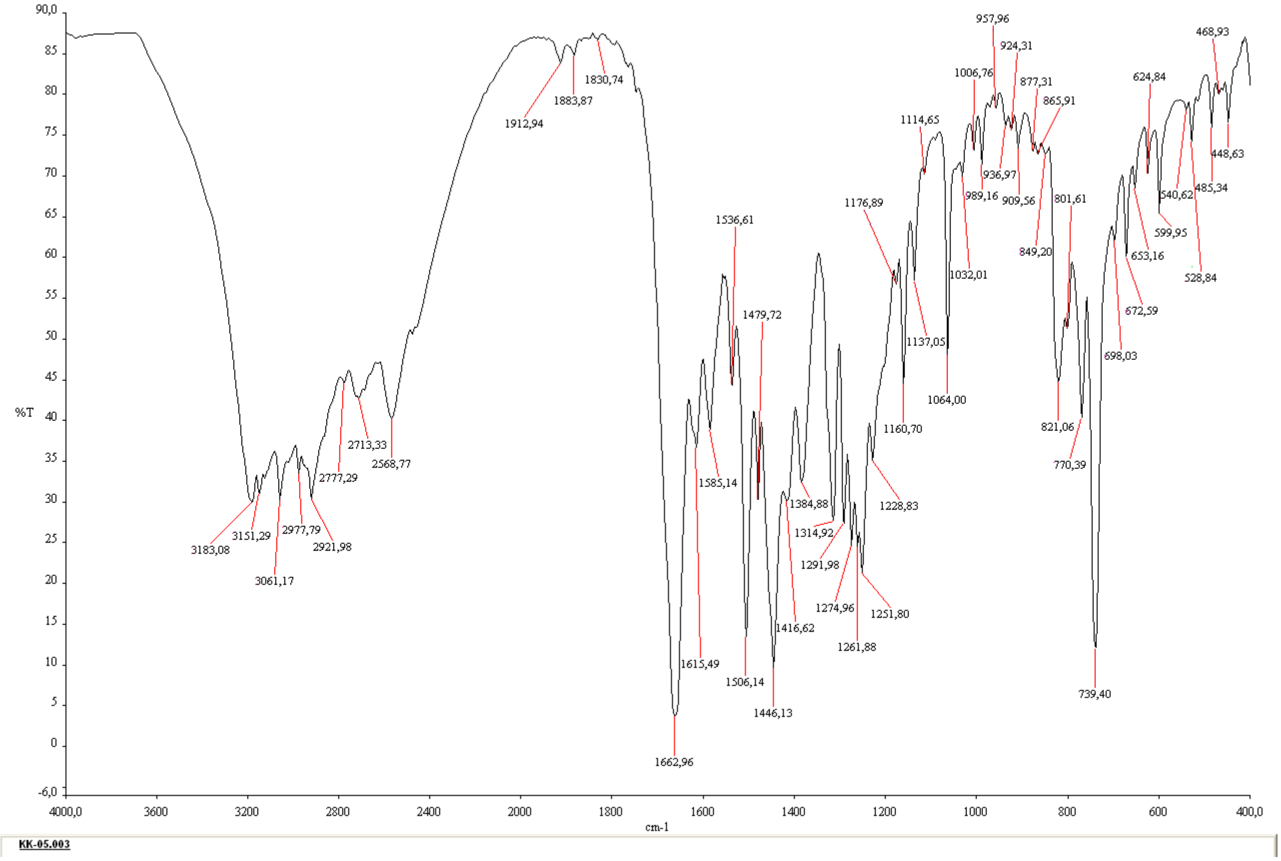

Figure S72. IR spectrum of compound **9a**.

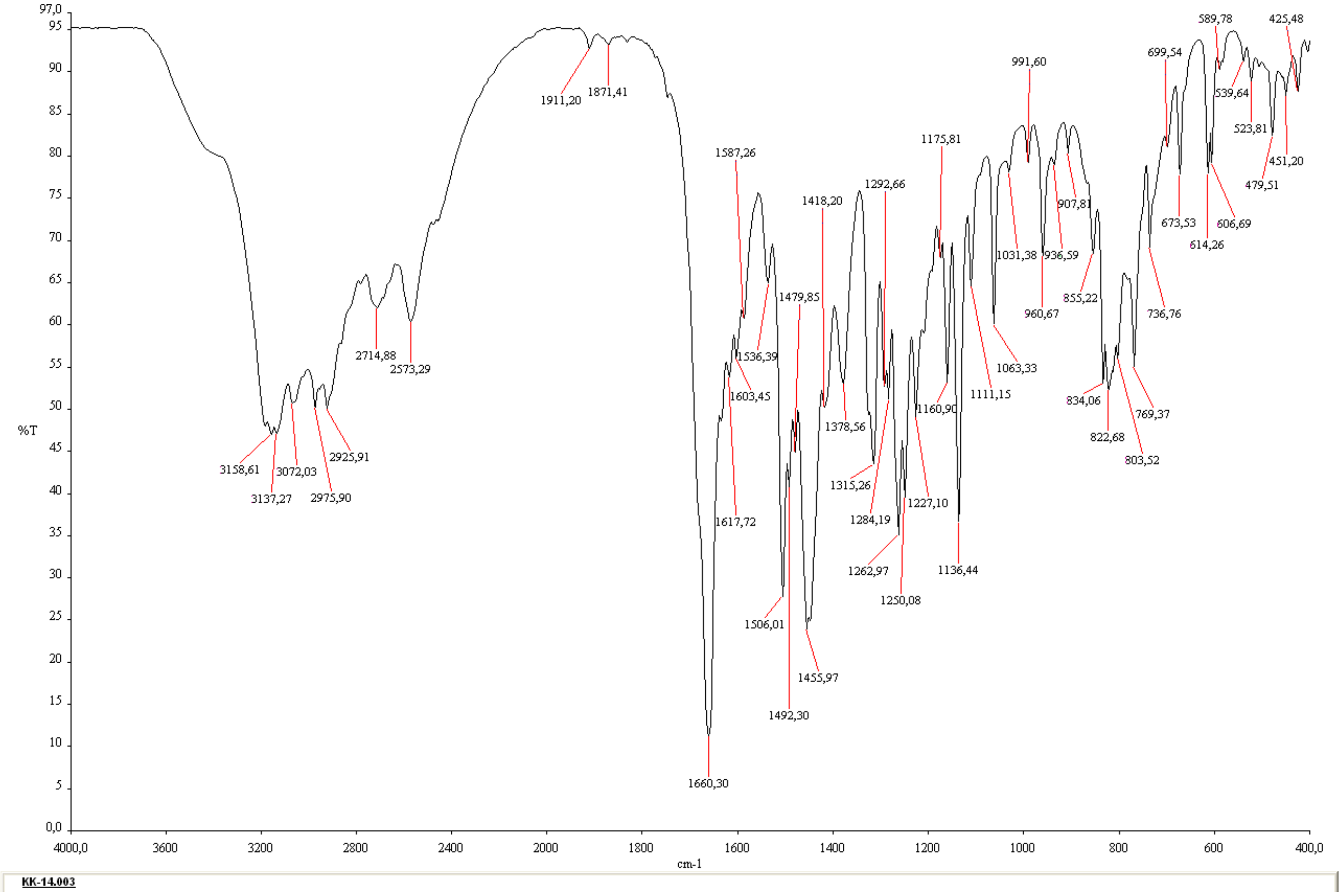

Figure S73. IR spectrum of compound **9b**.

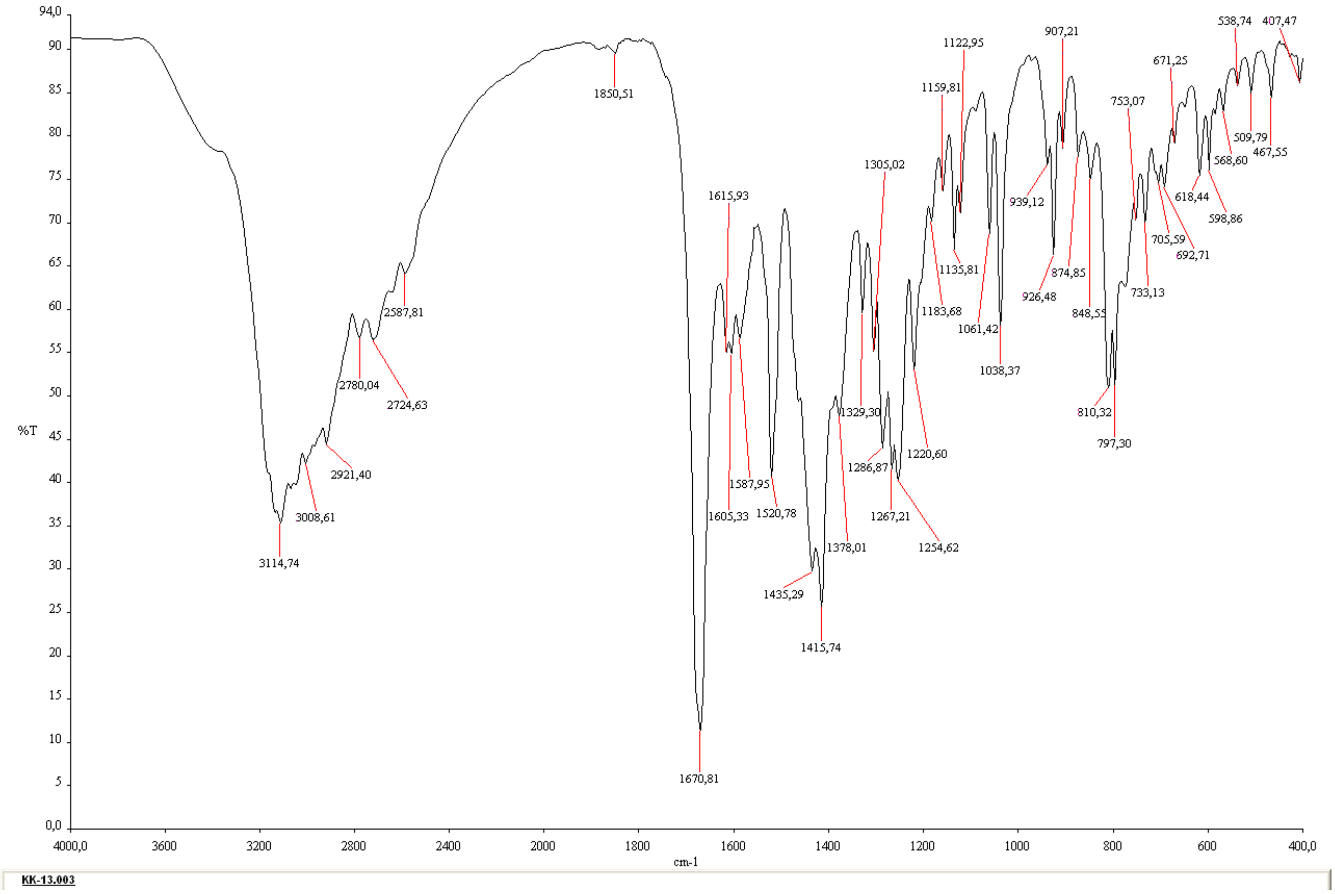

Figure S74. IR spectrum of compound **9c**.

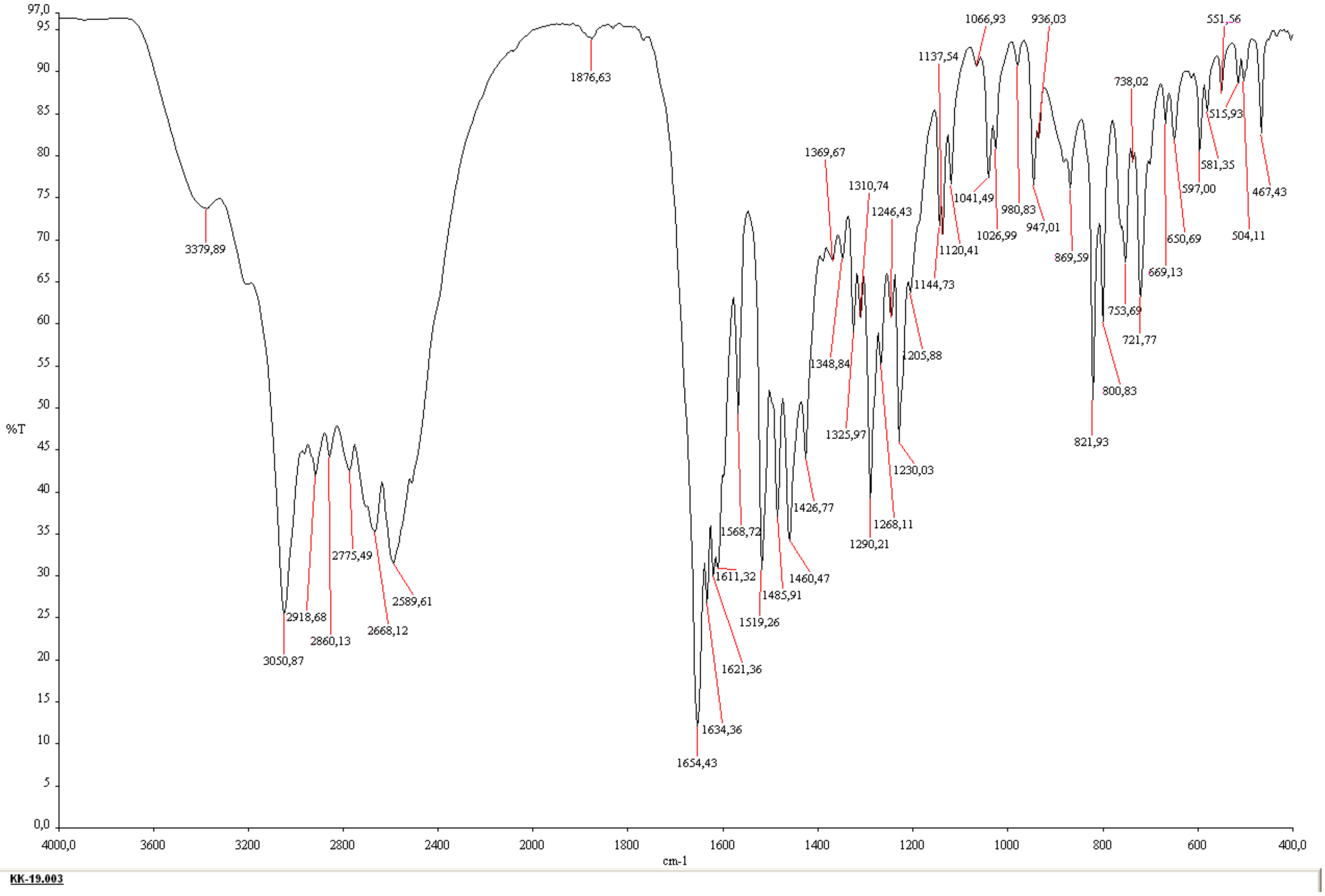

Figure S75. IR spectrum of compound **9d**.

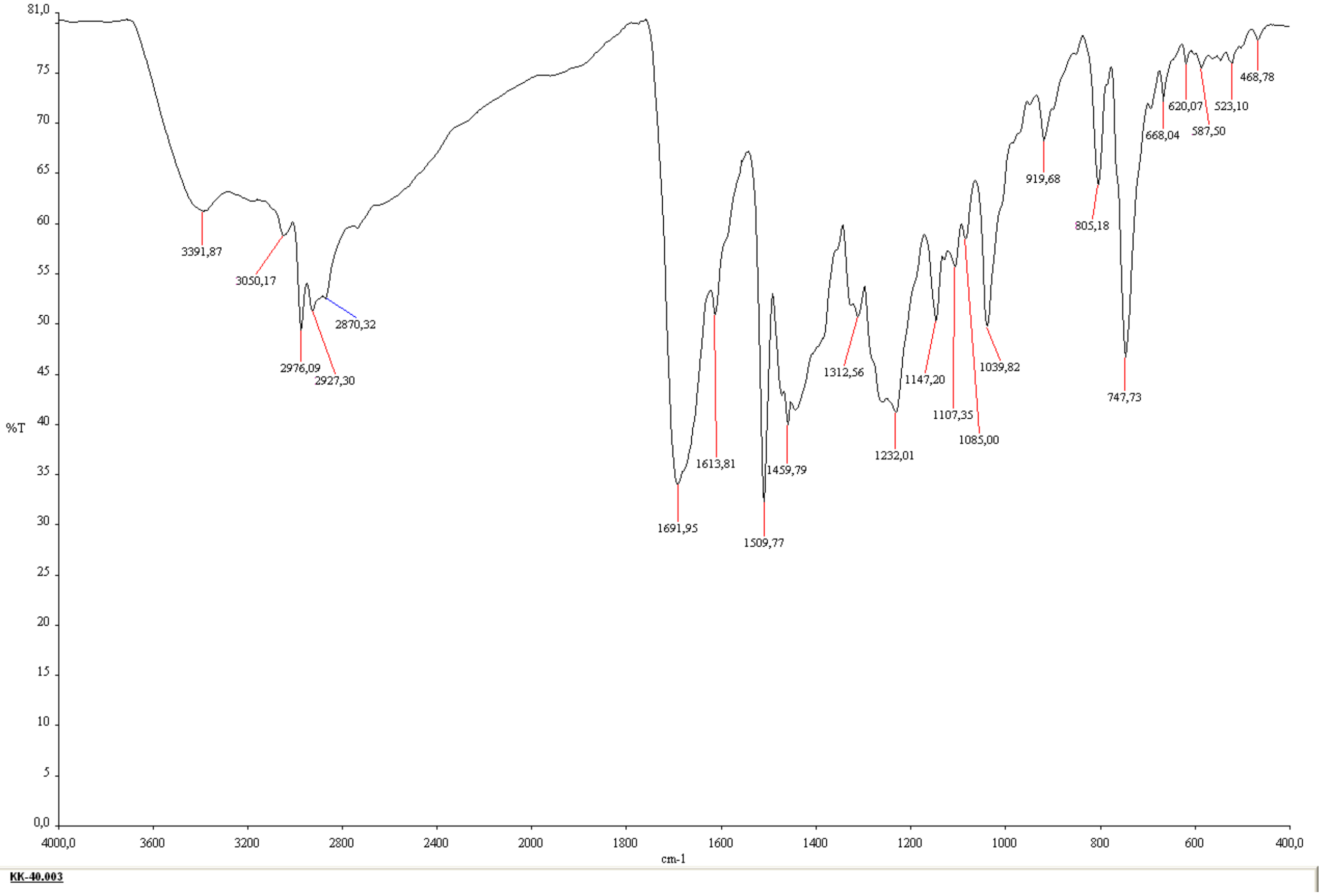

Figure S76. IR spectrum of compound **10**.

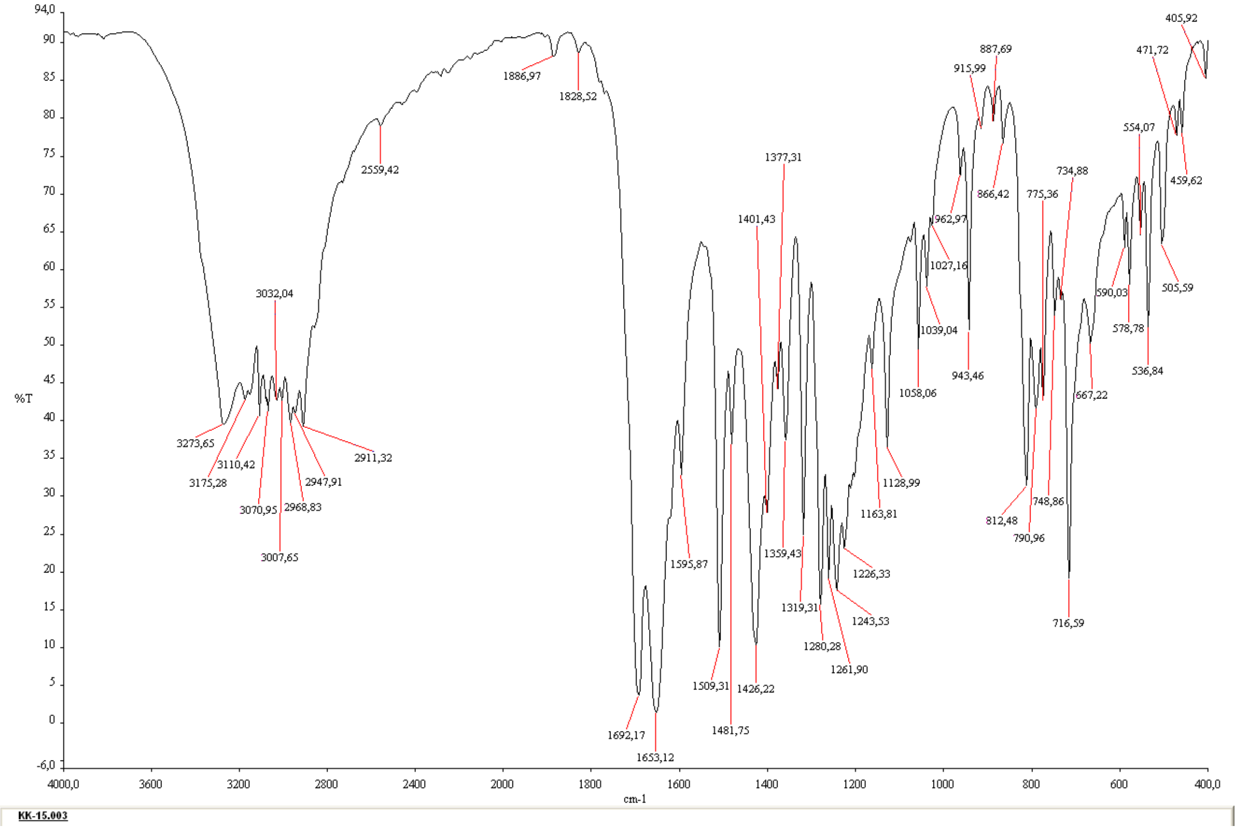

Figure S77. IR spectrum of compound **11a**.

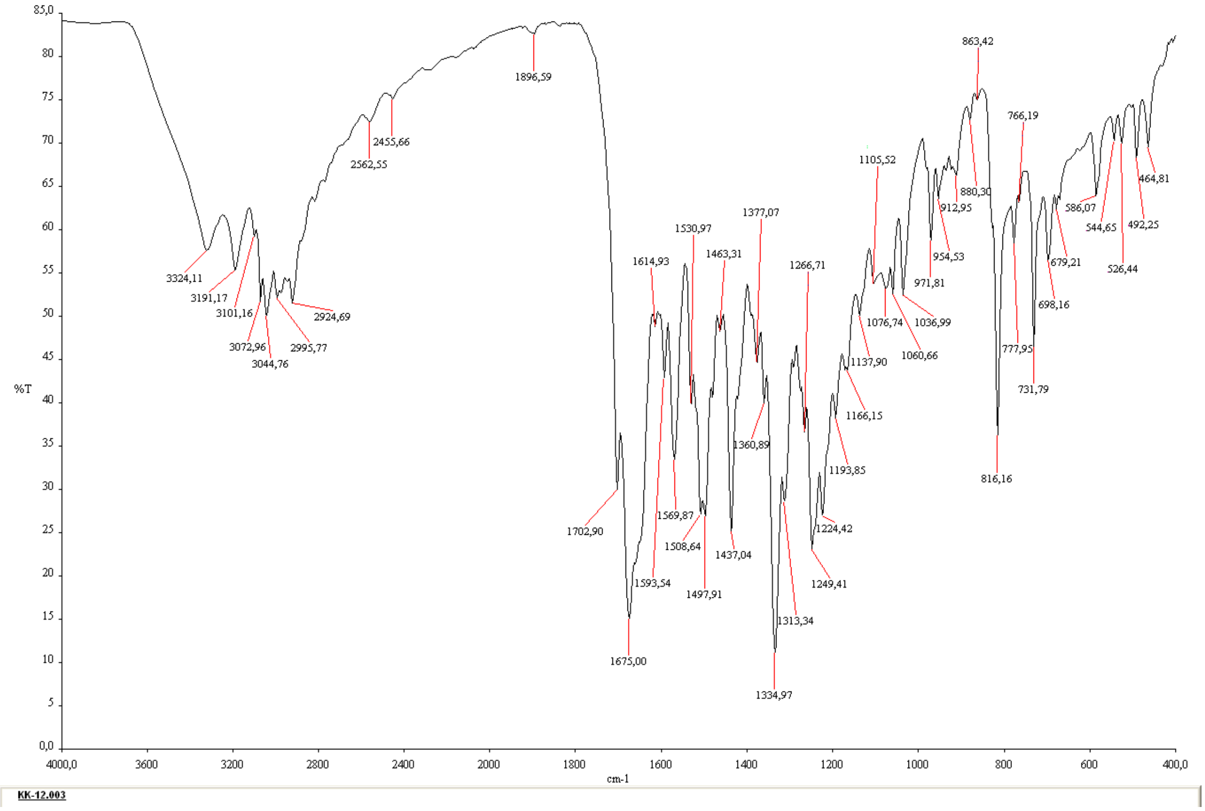

Figure S78. IR spectrum of compound **11b**.

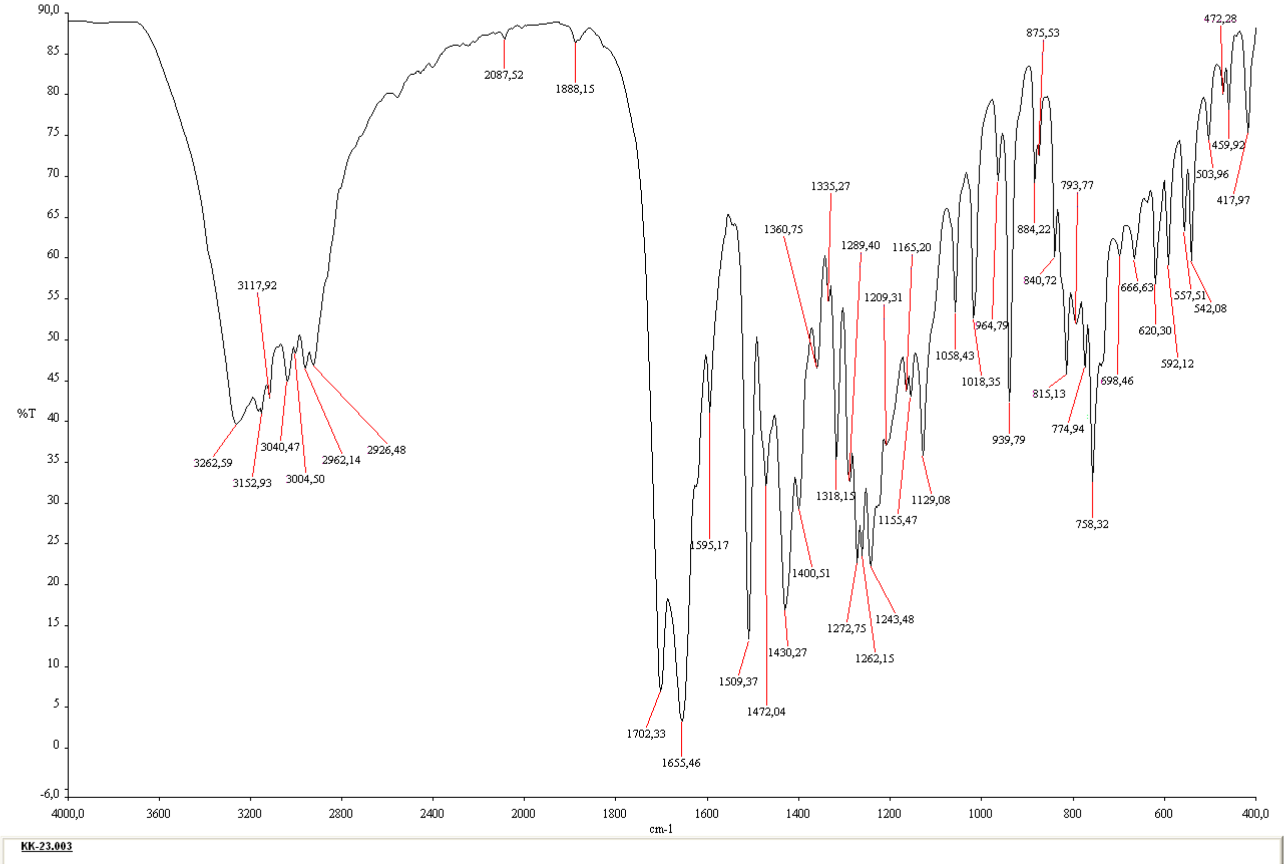

Figure S79. IR spectrum of compound **11c**.

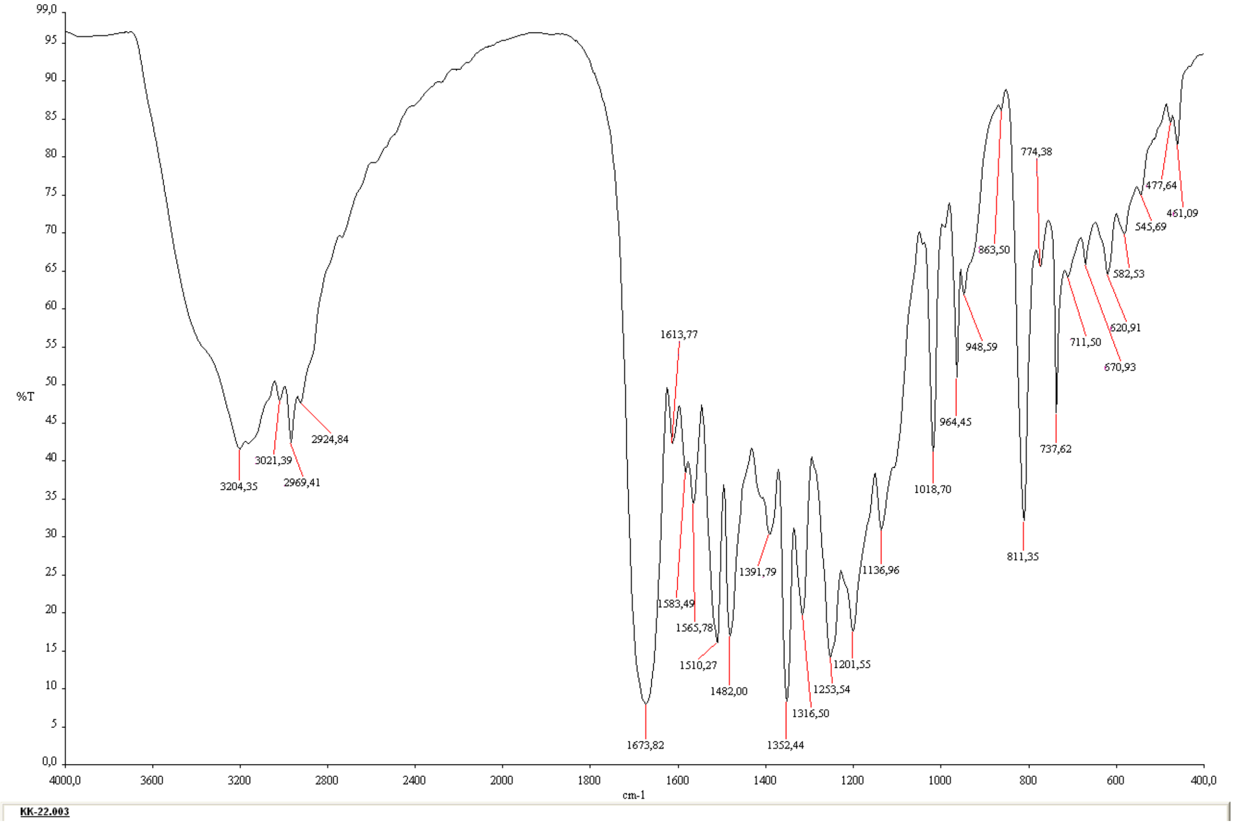

Figure S80. IR spectrum of compound **11d**.

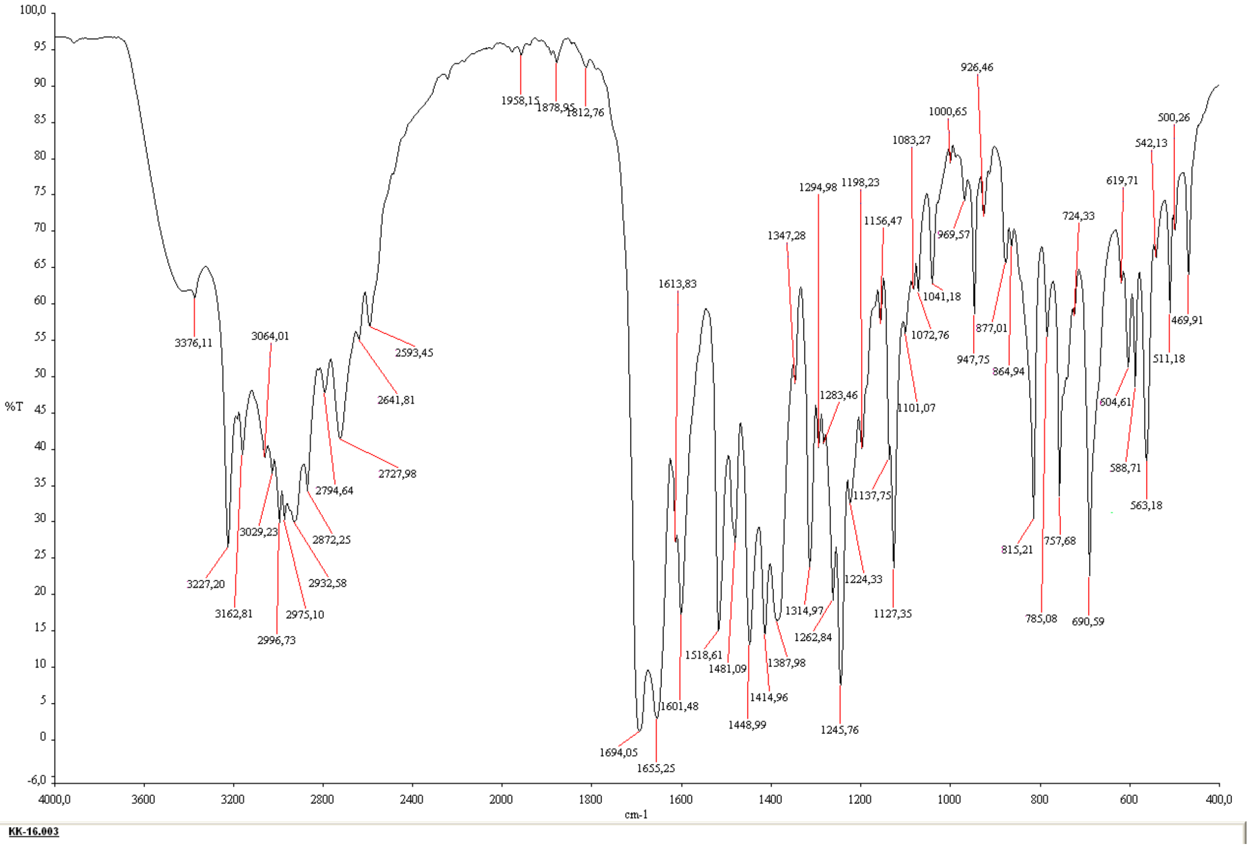

Figure S81. IR spectrum of compound **12a**.

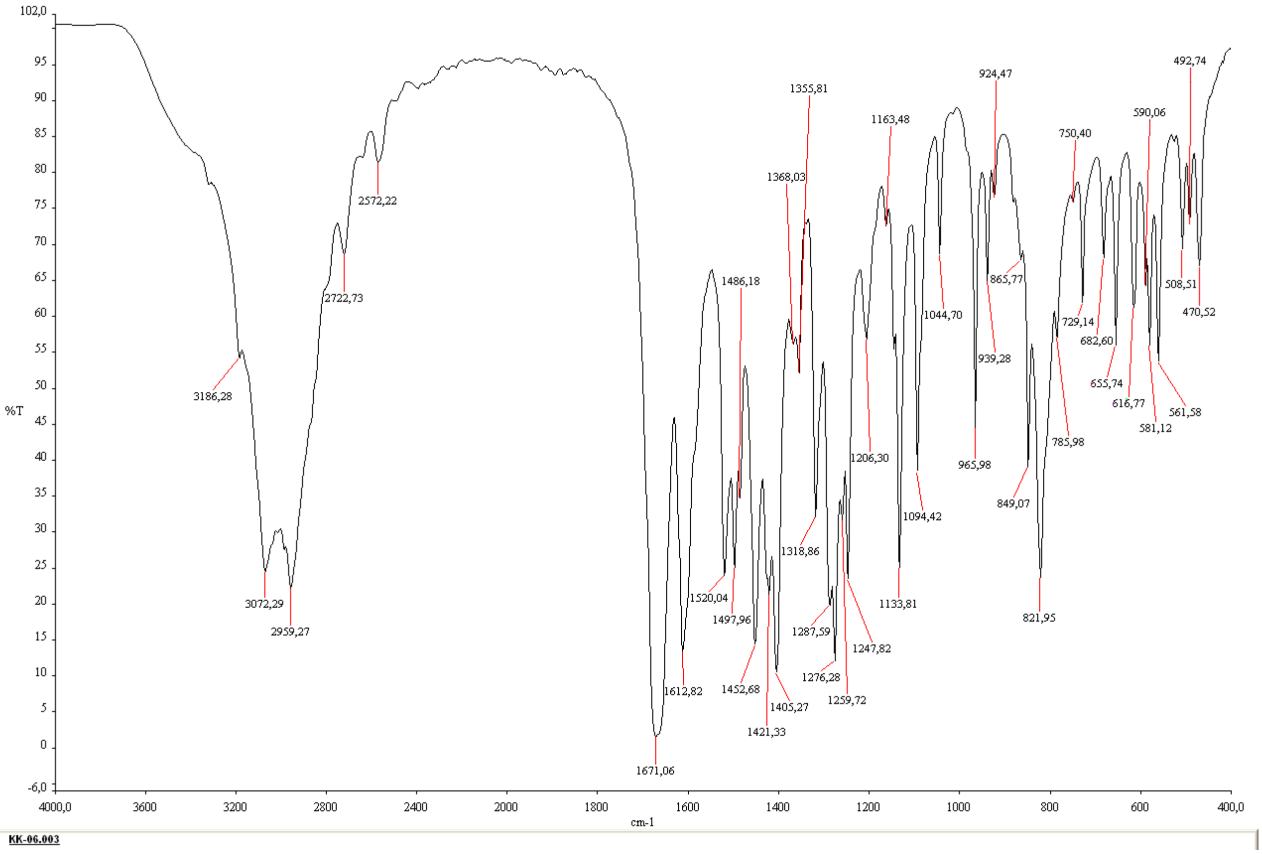

Figure S82. IR spectrum of compound **12b**.

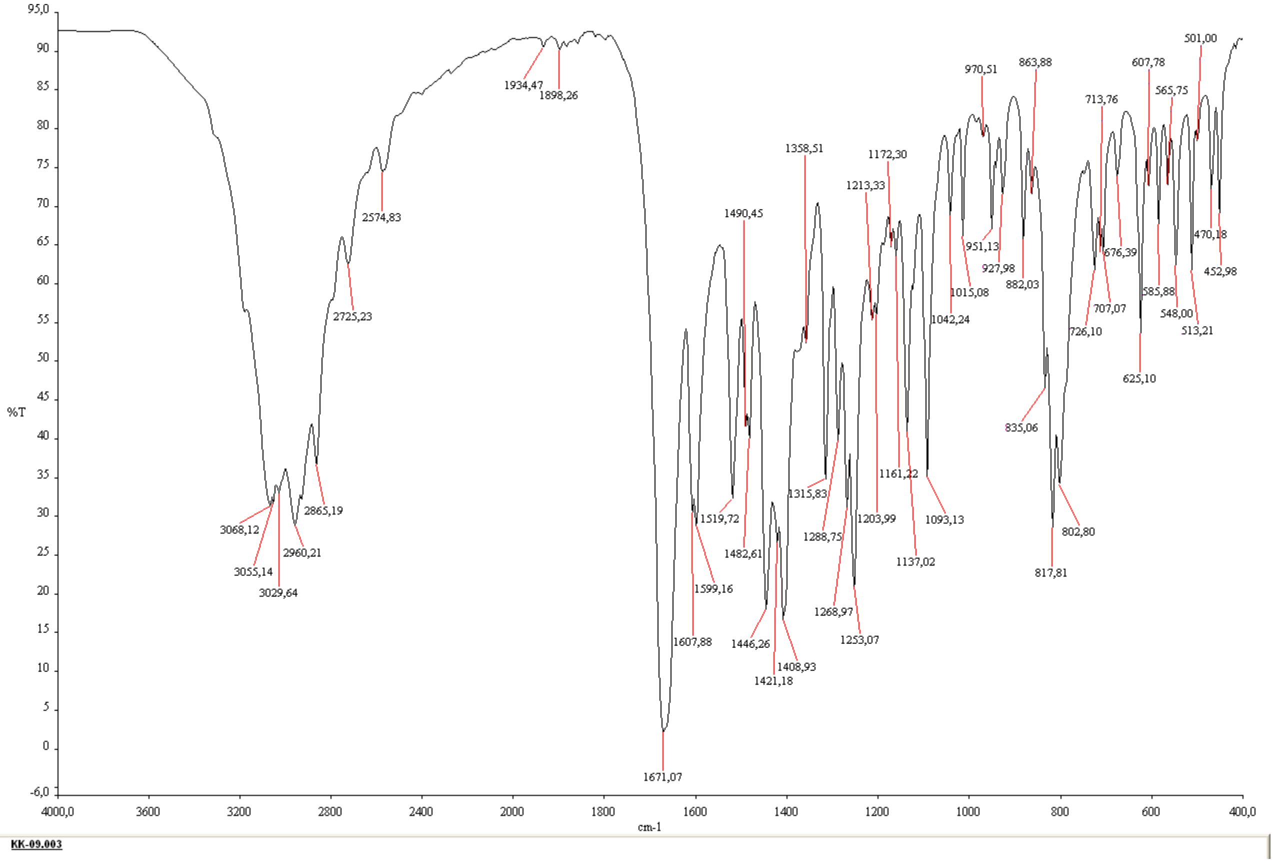

Figure S83. IR spectrum of compound **12c**.

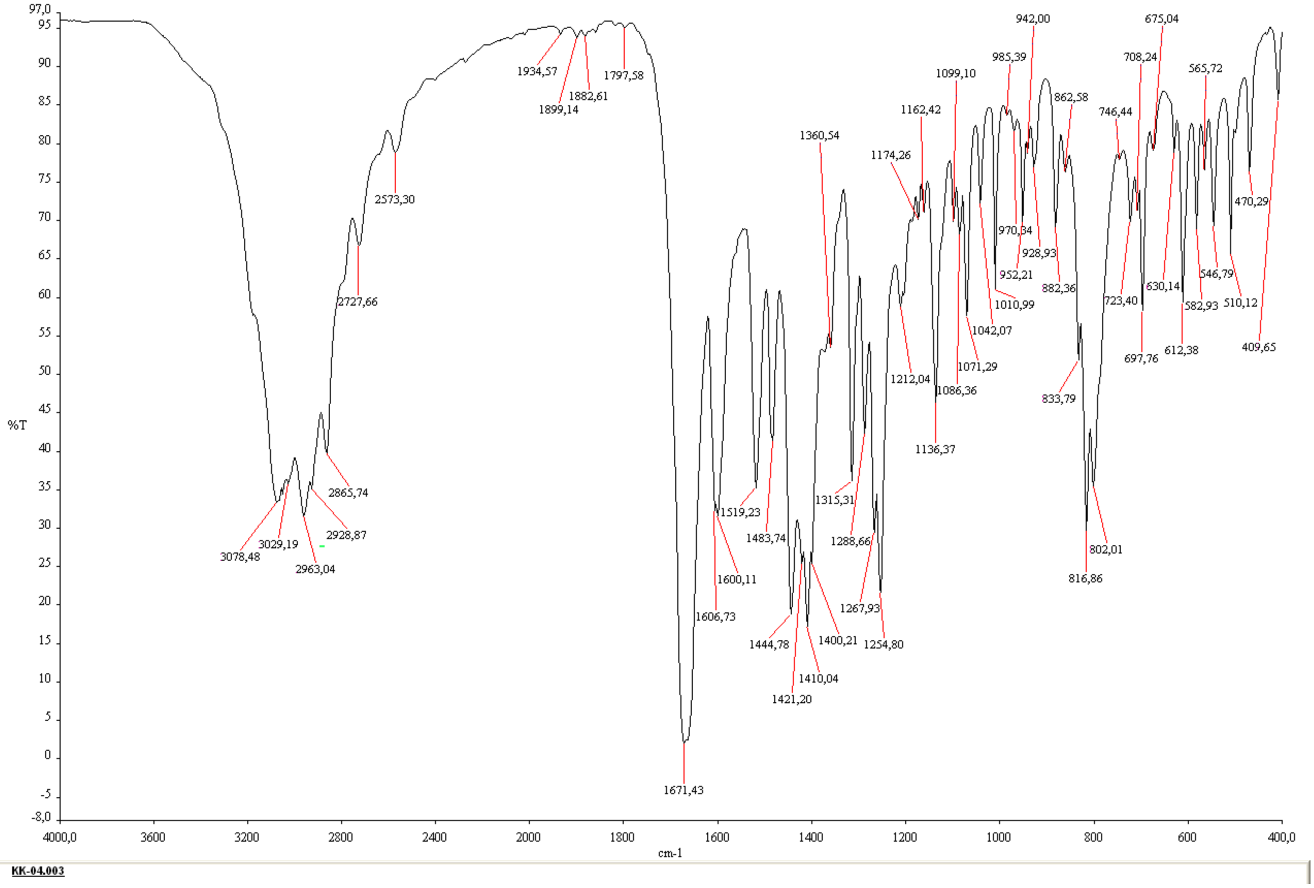

Figure S84. IR spectrum of compound **12d**.

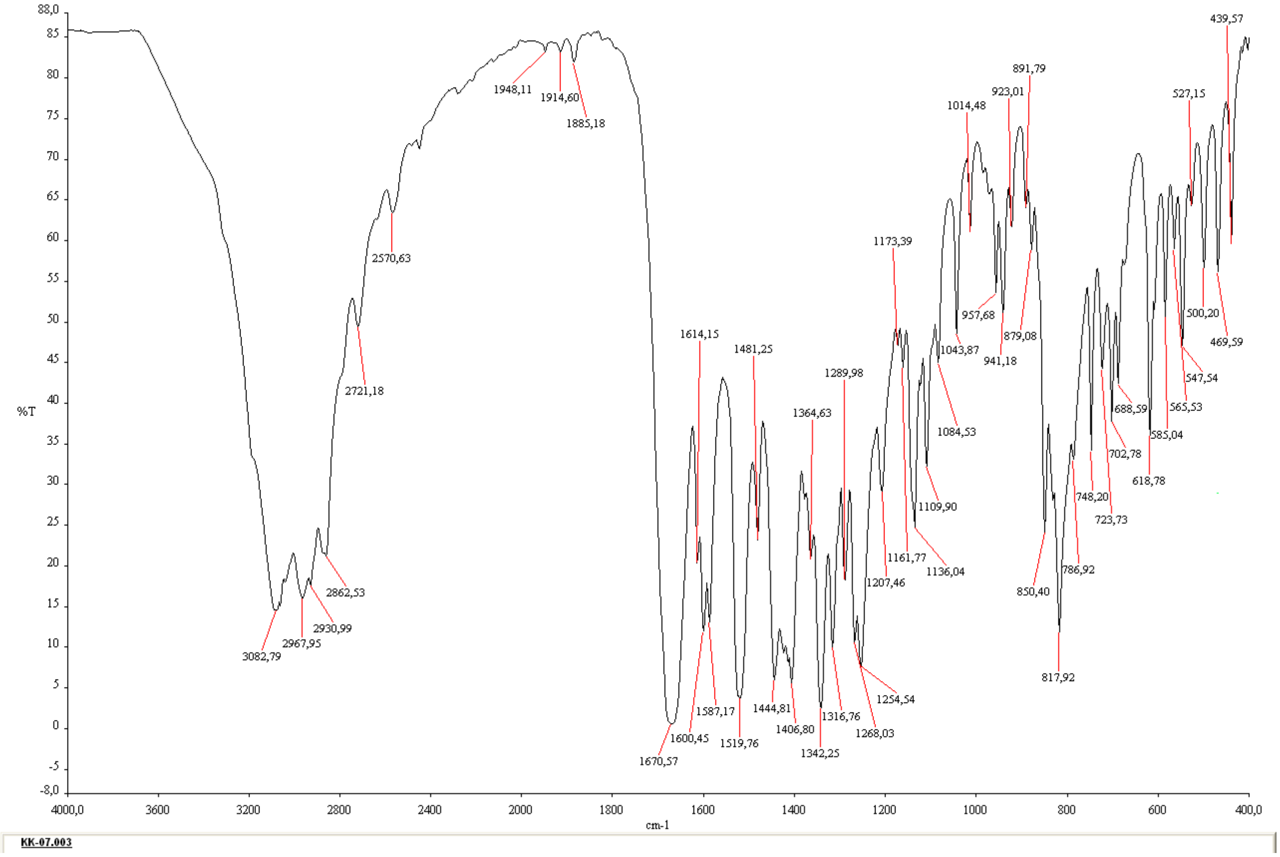

Figure S85. IR spectrum of compound **12e**.

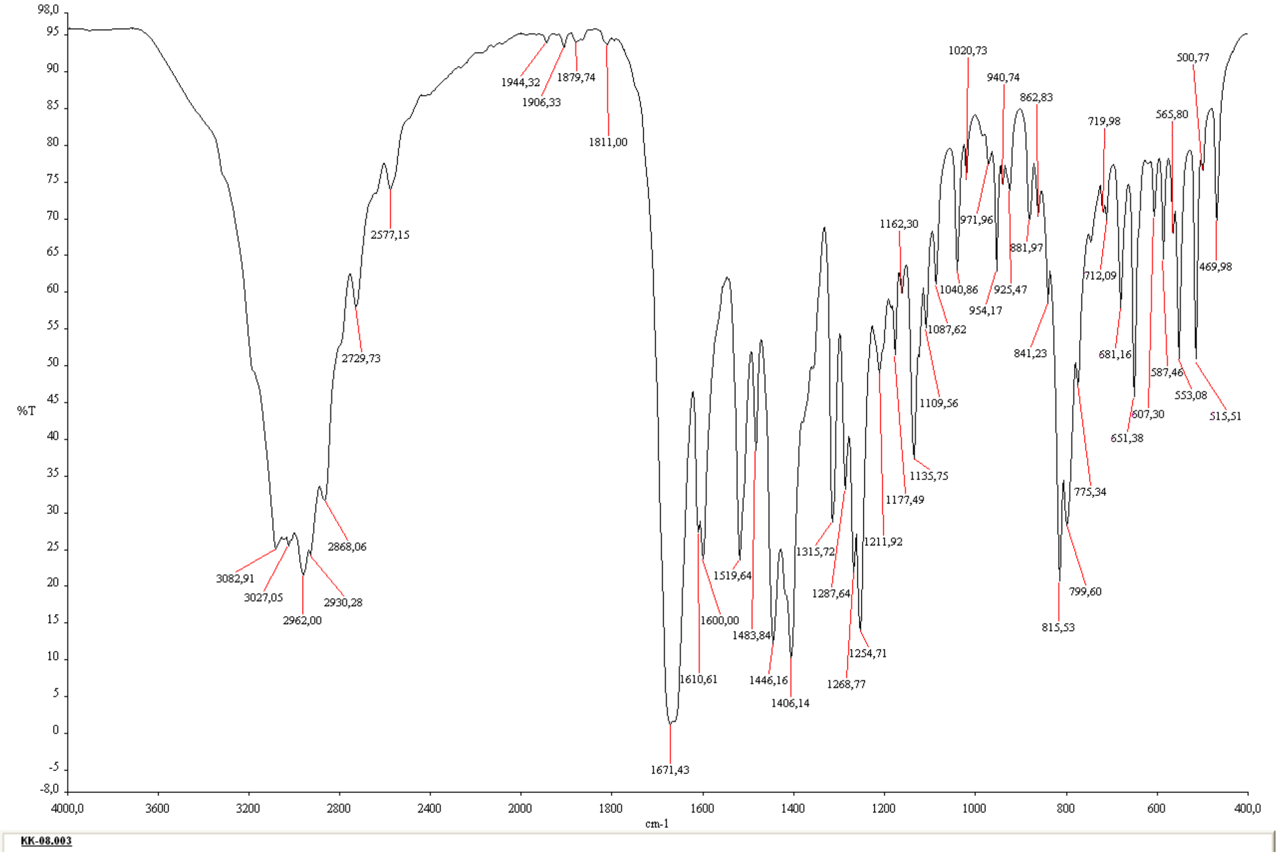

Figure S86. IR spectrum of compound **12f**.

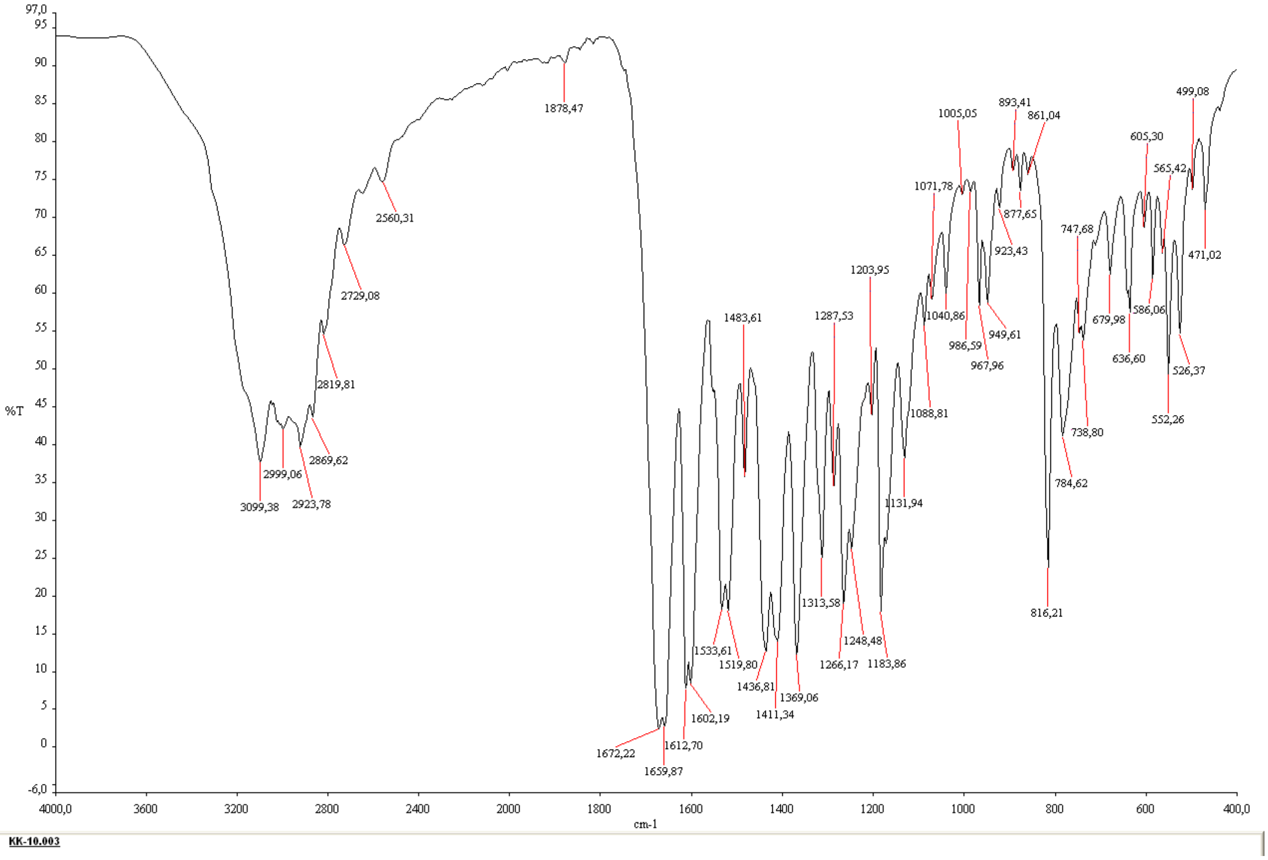

Figure S87. IR spectrum of compound **12g**.

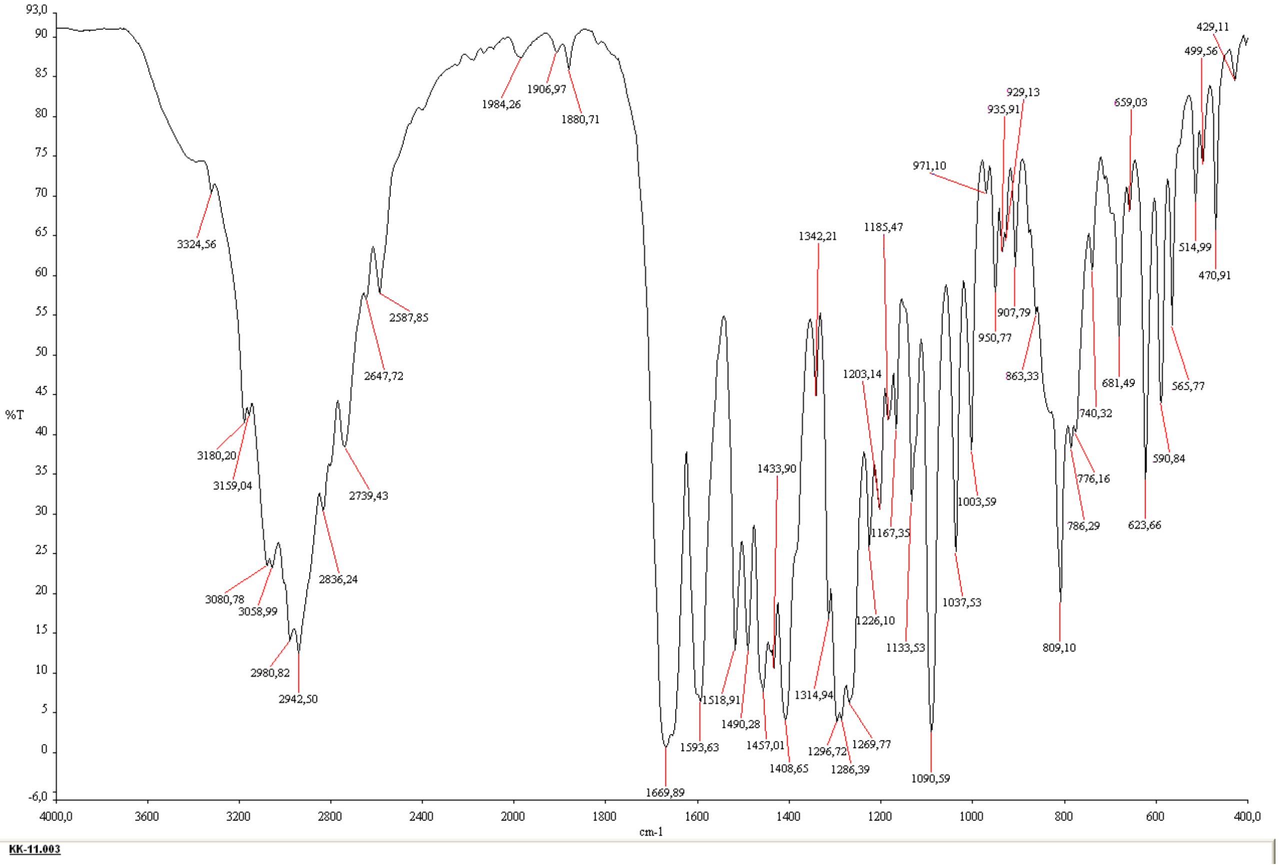

Figure S88. IR spectrum of compound **12h**.

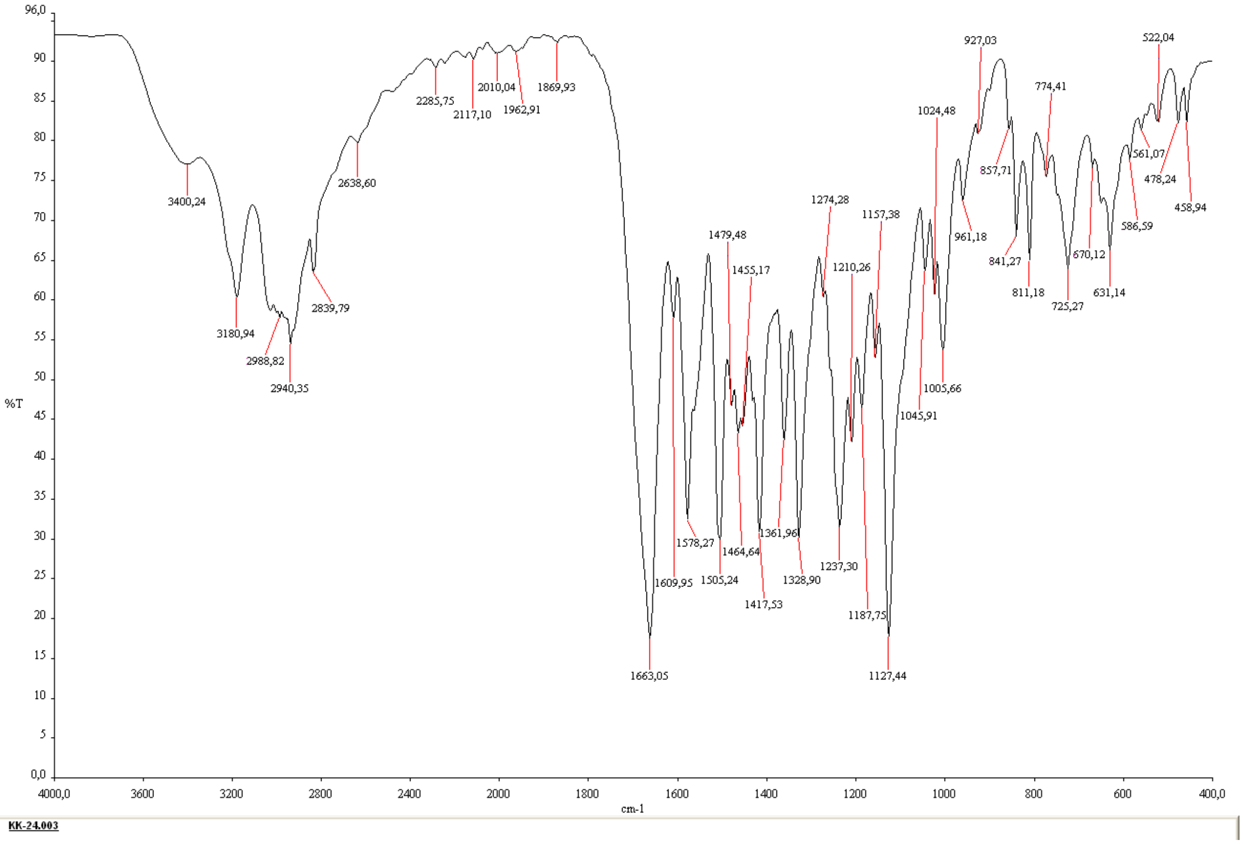

Figure S89. IR spectrum of compound **12i**.

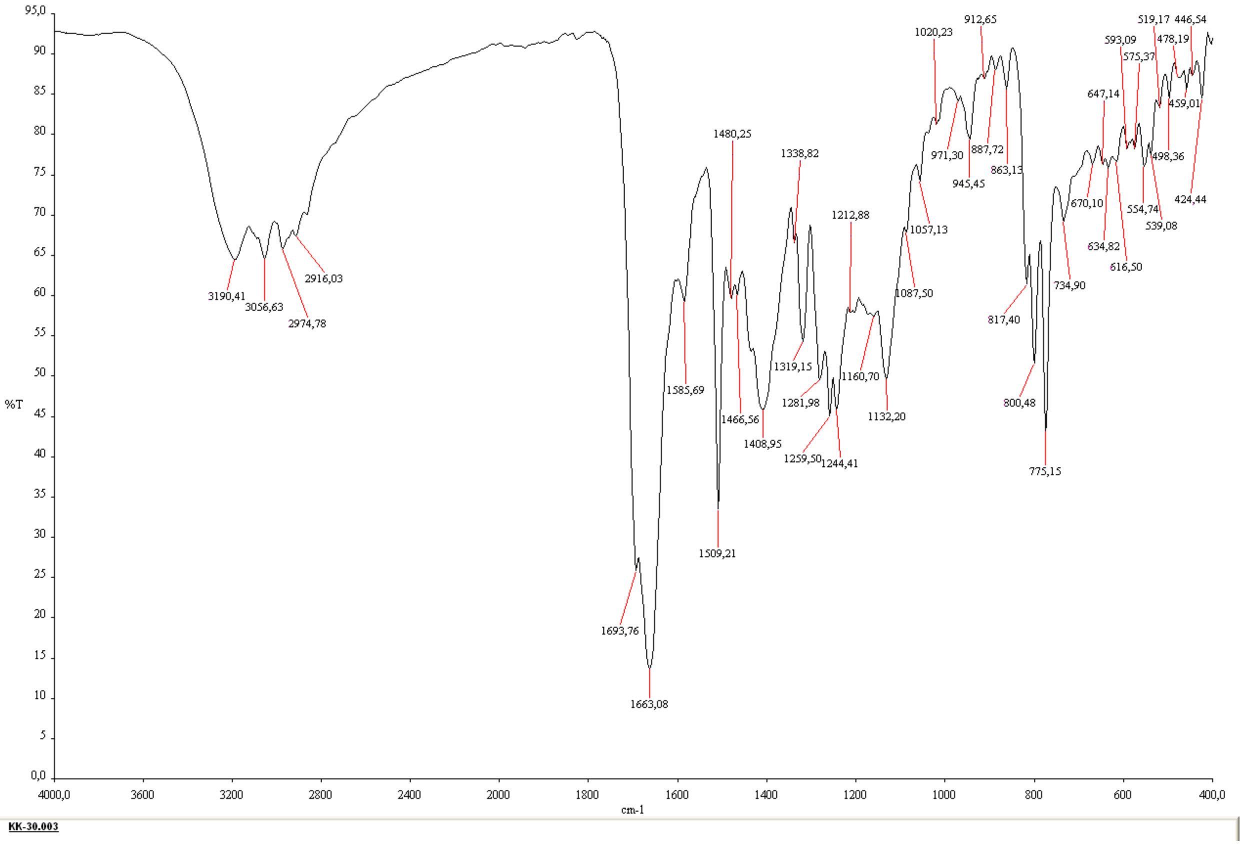

Figure S90. IR spectrum of compound **12j**.

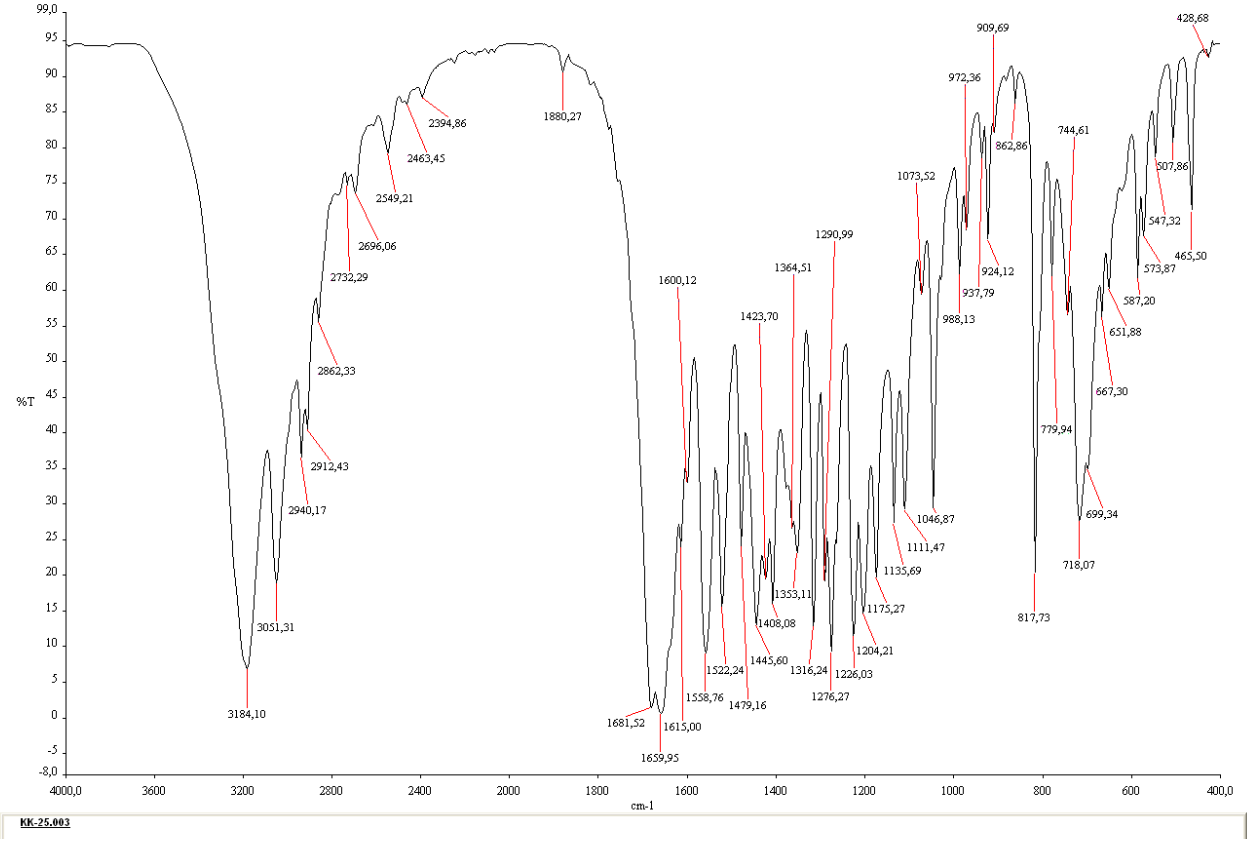

Figure S91. IR spectrum of compound **13a**.

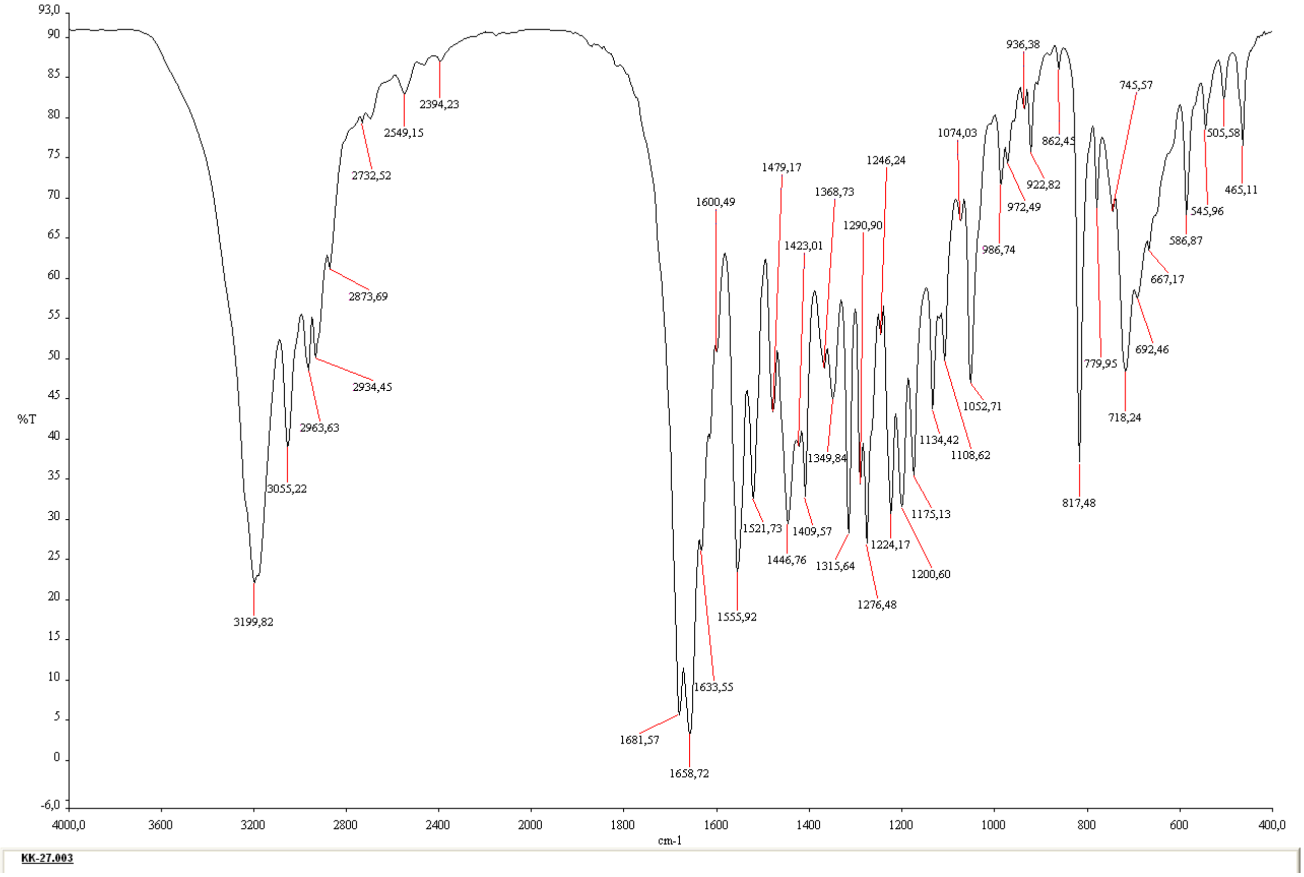

Figure S92. IR spectrum of compound **13b**.

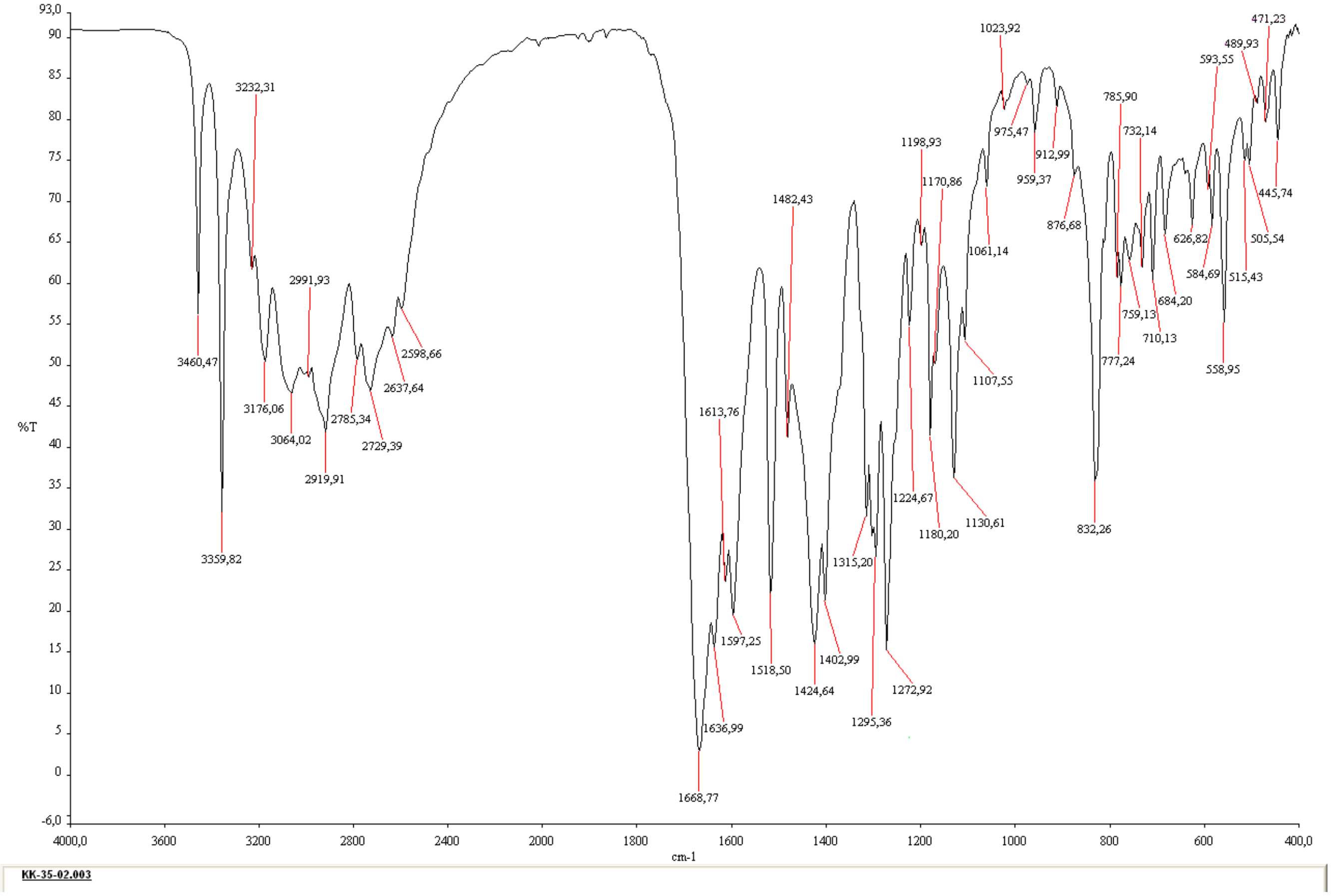

Figure S93. IR spectrum of compound **13c**.

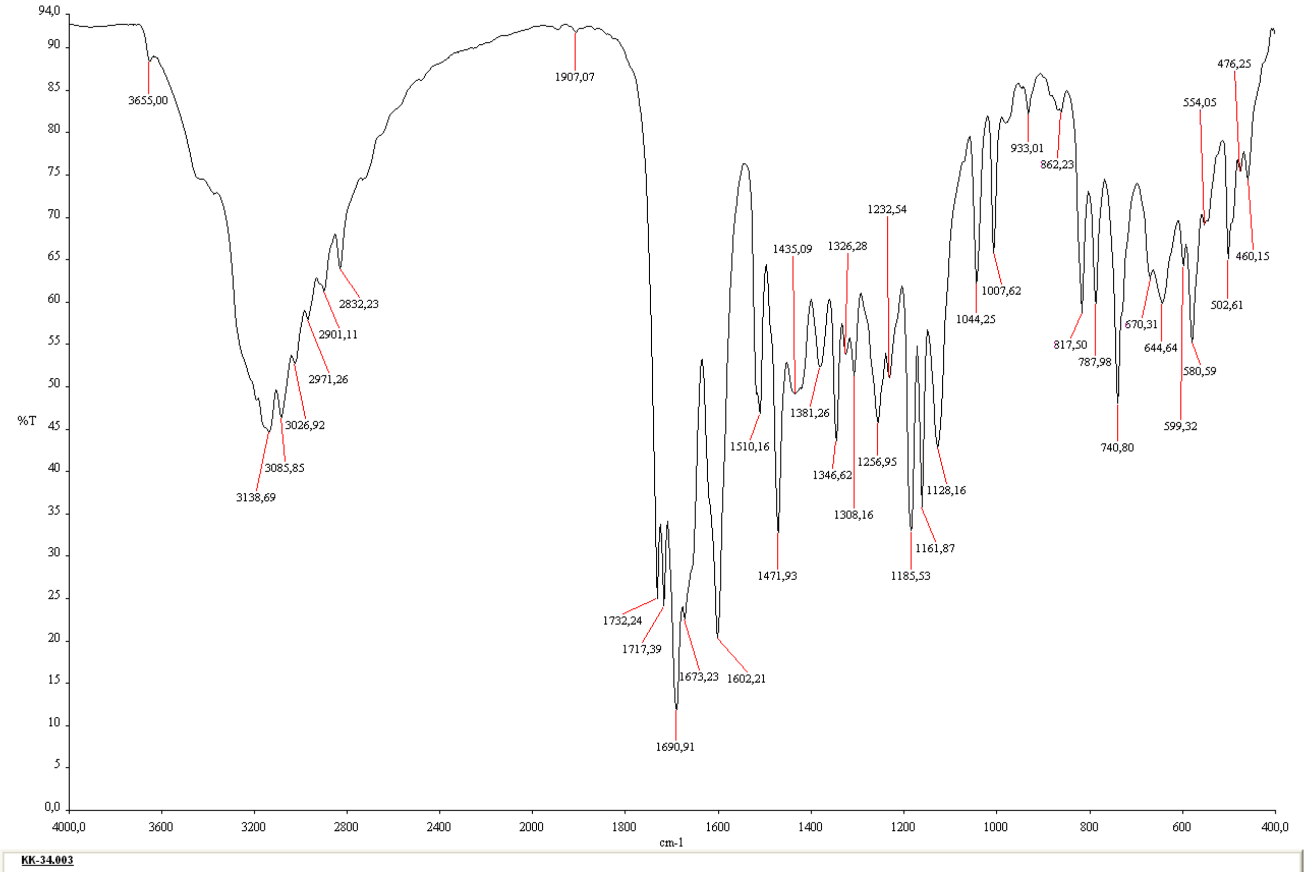

Figure S94. IR spectrum of compound **14**.

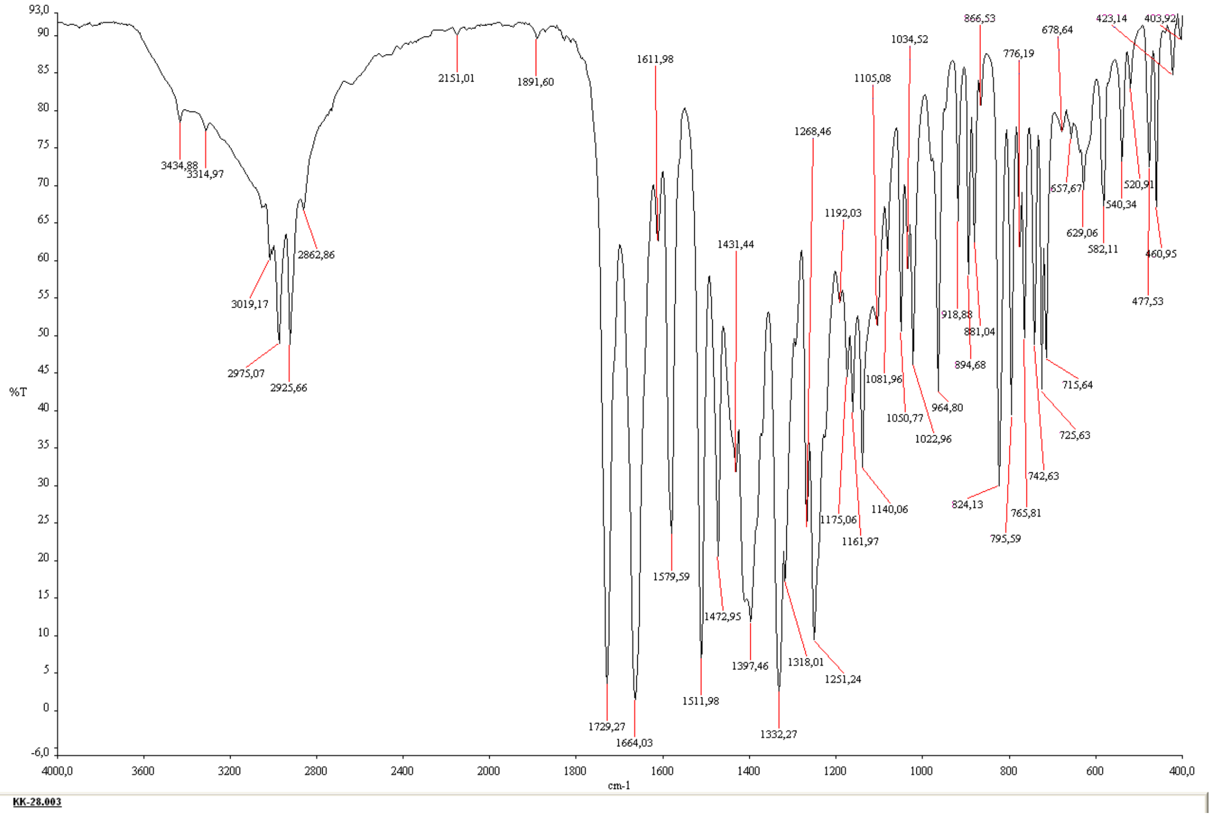

Figure S95. IR spectrum of compound **15**.

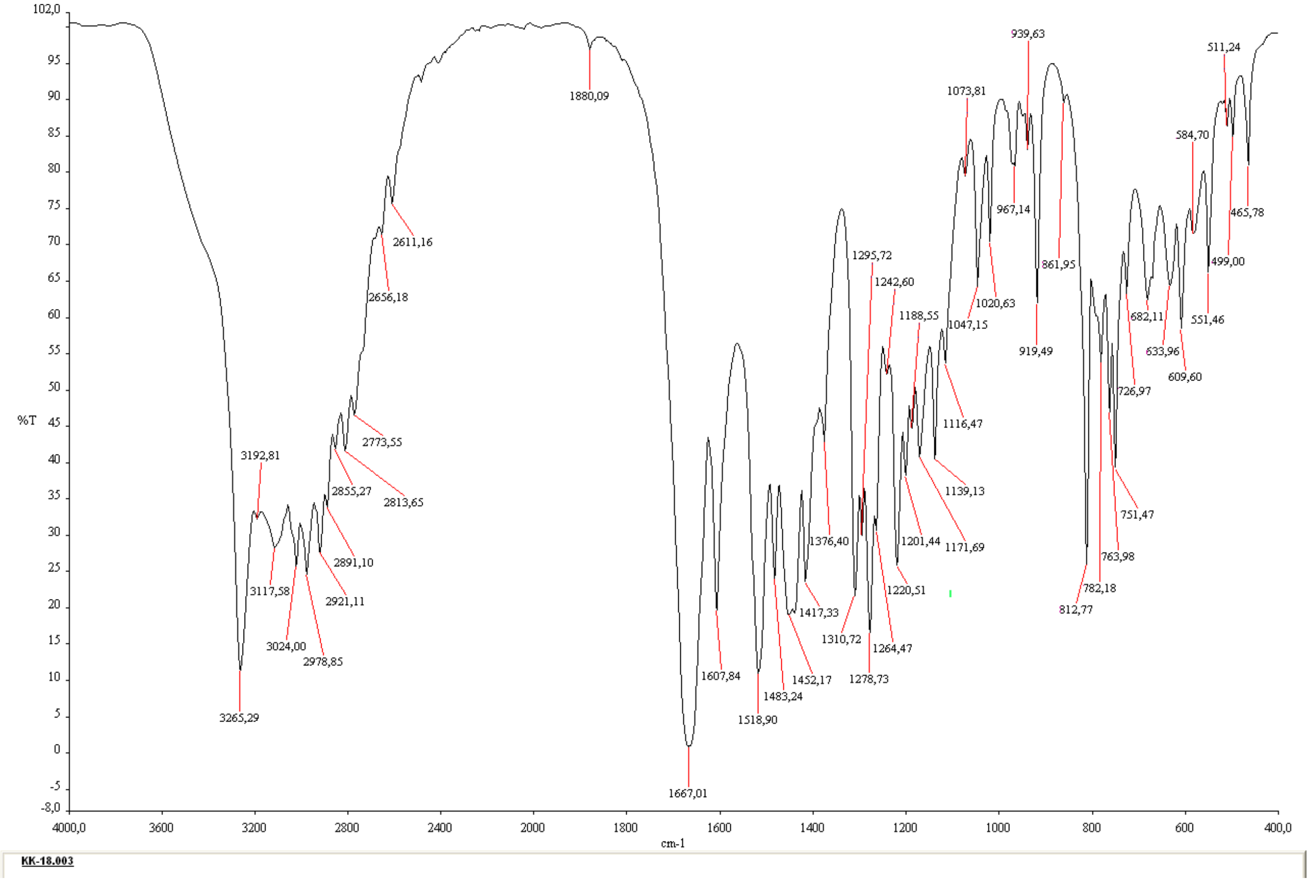

Figure S96. IR spectrum of compound **16**.
